# Supplementary material for: Paramagnetic encoding of molecules
Source: Nat Commun. 2022 Jun 8;13:3179. doi: 10.1038/s41467-022-30811-9 (PMC9177614; doi:10.1038/s41467-022-30811-9)
Supplement: Supplementary file 1 — Supplementary Information [file 41467_2022_30811_MOESM1_ESM.pdf]

Supplementary Information for

Paramagnetic encoding of molecules

Jan Kretschmer,[1,2] Tomáš David,[1] Martin Dračínský,[1] Ondřej Socha,[1] Daniel Jirák,[3,4] Martin Vít,[3,5] Radek Jurok,[6,7] Martin Kuchař,[6,8] Ivana Císařová,[9] Miloslav Polasek[1*]

[1] Institute of Organic Chemistry and Biochemistry of the CAS, Flemingovo náměstí 542/2, 160 00 Prague 6, Czech Republic.

[2] Department of Organic Chemistry, Faculty of Science, Charles University in Prague, Hlavova 2030/8, 128 43, Prague 2, Czech Republic.

[3] MR Unit, Department of Radiodiagnostic and Interventional Radiology, Institute for Clinical and Experimental Medicine, Vídeňská 1958/9, 140 21, Prague 4, Czech Republic.

[4] Institute of Biophysics and Informatics, First Faculty of Medicine, Charles University in Prague, Salmovská 1, 120 00, Prague 2, Czech Republic.

[5] Faculty of Mechatronics Informatics and Interdisciplinary studies, Technical University of Liberec, Hálkova 917/6, 460 01, Liberec, Czech Republic.

[6] Forensic Laboratory of Biologically Active Substances, Department of Chemistry of Natural Compounds, University of Chemistry and Technology Prague, Technická 5, 166 28, Prague 6, Czech Republic.

[7] Department of Organic Chemistry, University of Chemistry and Technology Prague, Technická 5, 166 28, Prague 6, Czech Republic.

[8] Department of Experimental Neurobiology, National Institute of Mental Health, Topolová 748, 250 67, Klecany, Czech Republic.

[9] Department of Inorganic Chemistry, Faculty of Science, Charles University in Prague, Hlavova 2030/8, 128 43, Prague 2, Czech Republic.

[*] E-mail: **miloslav.polasek@uochb.cas.cz**

Table of content

Supplementary Methods 5
Supplementary Fig. 1. Crystallographic details of [**Dy**(**L**[1])]·3.5H_2O 7
Supplementary Fig. 2. Coordination environment in [**Dy**(**L**[1])]·3.5H_2O 8
Supplementary Fig. 3. Crystal packing of [**Dy**(**L**[1])]·3.5H_2O 9
Supplementary Fig. 4. Suggested mechanism of epoxide opening leading to **6** and **6*** 10
Supplementary Fig. 5. Crystallographic details of [**Dy**(**L**[2])]·3H_2O 11
Supplementary Fig. 6. Coordination environment in [**Dy**(**L**[2])]·3H_2O 12
Supplementary Fig. 7. Crystal packing of [**Dy**(**L**[2])]·3H_2O 13
Supplementary Fig. 8. Suggested mechanism that allows [**M**(**L**[1])] to undergo amide coupling 14
Supplementary Fig. 9. ^{19}F NMR spectroscopy of **M**[1]**M**[2]**-TP1** statistical mixtures 15
Supplementary Fig. 10. Temperature effect on ^{19}F NMR signals of **DyHo-TP1** 16
Supplementary Fig. 11. ^{19}F NMR spectroscopy of **TP1** and **TP2** encoded with Dy^{3+}/Ho^{3+} ions........ 17
Supplementary Fig. 12. Synthesis of control **M**[1]**M**[2]**-TP3** based on **DOTA-K** ligand 18
Supplementary Fig. 13. ^{19}F NMR spectroscopy of **TP1** and **TP3** encoded with Dy^{3+}/Ho^{3+} ions........ 19
Supplementary Fig. 14. Fitting (two independent limited dataset) of **M**[1]**M**[2]**-TP1** ^{19}F NMR shifts 20
Supplementary Fig. 15. Fitting (full dataset) of **M**[1]**M**[2]**-TP1** ^{19}F NMR shifts 21
Supplementary Fig. 16. Comparison of pseudocontact paramagnetic shifts with Bleaney constants 22
Supplementary Fig. 17. Simulated ^{19}F NMR shifts of **M**[1]**M**[2]**-TP1** compounds 23
Supplementary Fig. 18. Fitting (reduced dataset) of **M**[1]**M**[2]**-TP1** ^{19}F NMR shifts 24
Supplementary Fig. 19. Decoding of **M**[1]**M**[2]**-TP1** sequences 25
Supplementary Fig. 20. ^{19}F NMR spectroscopy of **TP1** encoded with Tb^{3+}/Dy^{3+}/Ho^{3+}/Yb^{3+} ions 26
Supplementary Fig. 21. Signal-to-noise ^{19}F NMR measurements of **TP1** with Dy^{3+}/Ho^{3+} ions 27

*Synthesis of **L**[1] and **L**[2] building blocks:*

Supplementary Fig. 22. Synthesis of epoxide intermediates **5** and **5*** (scheme) 28
Supplementary Fig. 23. Synthesis of intermediate **1** 29
Supplementary Fig. 24. One-pot synthesis of intermediate **4** 30
Supplementary Fig. 25. Synthesis of intermediates **5** and **5*** 31
Supplementary Fig. 26. Synthesis of building blocks **L**[1], **L**[2] and their protected variants (scheme) 32
Supplementary Fig. 27. Synthesis of intermediate **6** 33
Supplementary Fig. 28. Synthesis of intermediate **7** 34
Supplementary Fig. 29. Synthesis of building block ***t*Bu$_3$L**[1] 35
Supplementary Fig. 30. Synthesis of building block **L**[1] 36
Supplementary Fig. 31. Synthesis of building block **Fmoc-L**[1] 37
Supplementary Fig. 32. Synthesis of intermediate **8** 38
Supplementary Fig. 33. Synthesis of building block **Ac-Fmoc-Me$_3$L**[1] 39
Supplementary Fig. 34. Synthesis of intermediate **7*** 40
Supplementary Fig. 35. Synthesis of building block ***t*Bu$_3$L**[2] 41
Supplementary Fig. 36. Synthesis of building block **L**[2] 42

*Synthesis of **M**[1]**M**[2]**-TP1** tripeptides based on **L**[1] architecture*:

Supplementary Fig. 37. Synthesis of partially protected tripeptide intermediate **II** (scheme) 43
Supplementary Fig. 38. Synthesis of intermediate **I** 44
Supplementary Fig. 39. Synthesis of intermediate **II** 45

Table of content

Supplementary Fig. 40. Synthesis of **M^1M^2-TP1** via post-synthetic complexation (scheme) 46
Supplementary Fig. 41. Synthesis of tripeptide **TP1** 47
Supplementary Fig. 42. Post-synthetis of **M^1M^2-TP1** statistical mixtures 48
Supplementary Fig. 43. Synthesis of **M^1M^2-TP1** with Tb^{3+}/Dy^{3+}/Ho^{3+}/Yb^{3+} ions (scheme) 49
Supplementary Fig. 44. Synthesis of intermediate **Tb-TP1** 50
Supplementary Fig. 45. Synthesis of intermediate **Dy-TP1** 51
Supplementary Fig. 46. Synthesis of intermediate **Ho-TP1** 52
Supplementary Fig. 47. Synthesis of intermediate **Yb-TP1** 53
Supplementary Fig. 48. Synthesis of **TbTb-TP1** 54
Supplementary Fig. 49. Synthesis of **DyTb-TP1** 55
Supplementary Fig. 50. Synthesis of **HoTb-TP1** 56
Supplementary Fig. 51. Synthesis of **YbTb-TP1** 57
Supplementary Fig. 52. Synthesis of **TbDy-TP1** 58
Supplementary Fig. 53. Synthesis of **DyDy-TP1** 59
Supplementary Fig. 54. Synthesis of **HoDy-TP1** 60
Supplementary Fig. 55. Synthesis of **YbDy-TP1** 61
Supplementary Fig. 56. Synthesis of **TbHo-TP1** 62
Supplementary Fig. 57. Synthesis of **DyHo-TP1** 63
Supplementary Fig. 58. Synthesis of **HoHo-TP1** 64
Supplementary Fig. 59. Synthesis of **YbHo-TP1** 65
Supplementary Fig. 60. Synthesis of **TbYb-TP1** 66
Supplementary Fig. 61. Synthesis of **DyYb-TP1** 67
Supplementary Fig. 62. Synthesis of **HoYb-TP1** 68
Supplementary Fig. 63. Synthesis of **YbYb-TP1** 69
Supplementary Fig. 64. Alternative synthesis of **M^1M^2-TP1** with Dy^{3+}/Ho^{3+} ions (scheme) 70
Supplementary Fig. 65. Synthesis of building block [**Dy**(**L^1**)] and [**Dy**(**L^1**)]·3.5H_2O single crystals 71
Supplementary Fig. 66. Synthesis of building block [**Ho**(**L^1**)] 72
Supplementary Fig. 67. Synthesis of intermediate **Dy-III** 73
Supplementary Fig. 68. Synthesis of intermediate **Ho-III** 74
Supplementary Fig. 69. Synthesis of intermediate **Dy-TP1** 75
Supplementary Fig. 70. Synthesis of intermediate **Ho-TPI** 76
Supplementary Fig. 71. Synthesis of **DyDy-TP1** 77
Supplementary Fig. 72. Synthesis of **HoDy-TP1** 78
Supplementary Fig. 73. Synthesis of **TmDy-TP1** 79
Supplementary Fig. 74. Synthesis of **DyHo-TP1** 80
Supplementary Fig. 75. Synthesis of **HoHo-TP1** 81
Supplementary Fig. 76. Synthesis of **DyHo-TP1** and **HoDy-TP1** using metal blocks (scheme) 82
Supplementary Fig. 77. Synthesis of building block [**Dy**(**Fmoc-L^1**)] 83
Supplementary Fig. 78. Synthesis of building block [**Ho**(**Fmoc-L^1**)] 84
Supplementary Fig. 79. Synthesis of intermediate **Dy-IV** 85
Supplementary Fig. 80. Synthesis of intermediate **Ho-IV** 86
Supplementary Fig. 81. Synthesis of intermediate **Dy-V** 87
Supplementary Fig. 82. Synthesis of intermediate **Ho-V** 88

Table of content

Supplementary Fig. 83. Synthesis of intermediate **Fmoc-HoDy-TP1** 89
Supplementary Fig. 84. Synthesis of intermediate **Fmoc-DyHo-TP1** 90
Supplementary Fig. 85. Synthesis of **HoDy-TP1** 91
Supplementary Fig. 86. Synthesis of **DyHo-TP1** 92

*Synthesis of **M^1M^2-TP2** tripeptides based on mixed **L^1/L^2** architecture:*

Supplementary Fig. 87. Synthesis of **M^1M^2-TP2** with Dy^{3+}/Ho^{3+} ions (scheme) 93
Supplementary Fig. 88. Synthesis of intermediate **I*** 94
Supplementary Fig. 89. Synthesis of intermediate **II*** 95
Supplementary Fig. 90. Synthesis of intermediate **Dy-TP2** 96
Supplementary Fig. 91. Synthesis of intermediate **Ho-TP2** 97
Supplementary Fig. 92. Synthesis of **DyDy-TP2** 98
Supplementary Fig. 93. Synthesis of **HoDy-TP2** 99
Supplementary Fig. 94. Synthesis of **DyHo-TP2** 100
Supplementary Fig. 95. Synthesis of **HoHo-TP2** 101
Supplementary Fig. 96. Synthesis of [**Dy**(**L^2**)] (scheme) 102
Supplementary Fig. 97. Synthesis of building block [**Dy**(**L^2**)] and [**Dy**(**L^2**)]$\cdot 3H_2O$ single crystals 103

*Synthesis of **DOTA-K** building blocks:*

Supplementary Fig. 98. Synthesis of building block **Fmoc-Me_3DOTA-K** (scheme) 104
Supplementary Fig. 99. Synthesis of intermediate **9** 105
Supplementary Fig. 100. Synthesis of intermediate **10** 106
Supplementary Fig. 101. One-pot synthesis of intermediate **13** 107
Supplementary Fig. 102. Synthesis of building block **Fmoc-Me_3DOTA-K** 108
Supplementary Fig. 103. Synthesis of building block **Phe**{***p*-CF_3**}**-*t*Bu_3DOTA-K** (scheme) 109
Supplementary Fig. 104. Synthesis of intermediate **14** 110
Supplementary Fig. 105. Synthesis of intermediate **15** 111
Supplementary Fig. 106. Synthesis of intermediate **16** 112
Supplementary Fig. 107. Synthesis of intermediate **17** 113
Supplementary Fig. 108. Synthesis of building block **Phe**{***p*-CF_3**}**-*t*Bu_3DOTA-K** 114

*Synthesis of **M^1M^2-TP3** control tripeptides based on **DOTA-K** architecture:*

Supplementary Fig. 109. Synthesis of **HoDy-TP3** and **DyDy-TP3** (scheme) 115
Supplementary Fig. 110. Synthesis of intermediate **VI** 116
Supplementary Fig. 111. Synthesis of intermediate **Dy-TP3** 117
Supplementary Fig. 112. Synthesis of intermediate **Ho-TP3** 118
Supplementary Fig. 113. Synthesis of **DyDy-TP3** 119
Supplementary Fig. 114. Synthesis of **HoDy-TP3** 120
Supplementary Fig. 115. Synthesis of **DyHo-TP3** 121
Supplementary Fig. 116. Synthesis of **HoHo-TP3** 122

Supplementary References 123

Supplementary Methods

General: All chemicals were purchased from *Sigma-Aldrich* and *Fluorochem*. Dry solvents were purchased from *Acros Organics*. Deuterated solvents were purchased from *Eurisotop*. Throughout the text, all use of H_2O means Milli-Q water (18.2 mΩ·cm) and all use of M/Ln means M^{3+}/Ln^{3+}. Following abbreviations are used throughtout the text: EIC (extracted ion chromatogram); TEA (Et_3N), FA (HCOOH), TFA (CF_3COOH), PYAOP ((*7*-azabenzotriazol-*1*-yloxy)tripyrrolidinophosphonium hexafluorophosphate), DMAP (*4*-dimethylaminopyridine), MOPS (*3*-(*N*-morpholino)propanesulfonic acid), SNR (signal-to-noise ratio).

NMR spectroscopy: ^1H, ^{13}C and ^{19}F NMR spectra were recorded on Avance III™ HD 400 MHz spectrometer (*Bruker*, 400.1 MHz for ^1H, 100.6 MHz for ^{13}C) system equipped with either broad-band Prodigy cryo-probe with ATM module (5 mm CPBBO BB-$^1H/^{19}F/D$ Z-GRD) or inversion broadband probe with ATM module (5 mm PA BBI ^1H/D-BB Z-GRD), Avance III™ HD 500 MHz spectrometer (*Bruker*, 500.0 MHz for ^1H, 125.7 MHz for ^{13}C, 470.4 MHz for ^{19}F) equipped with broad-band cryo-probe with ATM module (5 mm CPBBO BB-$^1H/^{19}F/^{15}N/D$ Z-GRD) or Avance III™ HD 600 MHz spectrometer (*Bruker*, 600.1 MHz for ^1H, 150.9 MHz for ^{13}C) equipped with inverse triple resonance cryo-probe with ATM module (5 mm CPTCI $^1H/^{13}C/^{15}N/D$ Z-GRD). The temperature of the measurement is given for each experiment. Chemical shifts are in ppm, coupling constants in Hz. ^{19}F NMR spectra were referenced to TFA ($\delta_F = -76.55$ ppm). Signals of cyclen macrocycle are abbreviated *mc*. Integrals in showcased NMR spectra were rounded to integers for clarity reasons (obscured signals that cannot be exactly integrated are colored grey). Signals arising from two rotamers (if resolved) are listed together, separated by semicolon.

Liquid chromatography: LC-MS experiments were performed on 1260 Infinity II (*Agilent*) equipped with Luna® Omega Polar column (5 µm, 100 Å, 150 × 4.6 mm) using H_2O–MeCN gradients (1 mL min^{-1} flow rate) with FA (0.1%) as an additive. Three methods were used: **Method 1** (0 min → 1 min 5% MeCN; 1 min → 10 min 5% → 100% MeCN); **Method 2** (0 min → 3 min 0% MeCN; 3 min → 10 min 0% → 50% MeCN); **Method 3** (0 min → 0.5 min 5% MeCN; 0.5 min → 5 min 5% → 100% MeCN; 5 min → 5.5 min 100% MeCN). **Method 4** (0 min → 3 min 5% MeCN; 3 min → 4 min 5% → 40% MeCN; 4 min → 10 min 40% → 75% MeCN). Preparative HPLC experiments were performed on 1260 Infinity II (*Agilent*) equipped with YMC-Actus Triart C18 column (5 µm, 100 Å, 250 × 20.0 mm) using H_2O–MeCN gradients (20 mL min^{-1} flow rate) with either FA (0.1%) or TFA (0.1%) as an additive. Normal phase flash chromatography (SiO_2, 40–60 µm, 60 Å) was performed on Combi*Flash*® NEXTGEN 300+ system from *Teledyne ISCO*. Reverse phase flash chromatography was performed with RediSep® R_f Gold C18 Aq column (*Teledyne ISCO*, 150 g) using H_2O–MeCN gradient system with TFA (0.1 %) as an additive.

High-resolution mass spectra: HRMS (with ESI ionization) were recorded on an *Agilent* 5975C MSD Quadrupol, Q-Tof micro from *Waters* or LTQ Orbitrap XL from *Thermo Fisher Scientific*.

Supplementary methods (continuation)

Elemental analysis: CHN elemental analysis was performed on PE 2400 Series II CHN Analyzer from *Perkin Elmer*. Fluorine elemental analysis was performed by initial combustion of the sample in quartz vessel, followed by adsorption of resulting HF in H_2O and determining its concentration by potentiometry using fluoride ion selective electrode. Lanthanide content was determined by ICP-AES (SPECTRO Arcos from *SPECTRO Analytical Instruments*). All EA data are presented in calcd. (found) format.

X-Ray diffraction: The diffraction experiment for crystal structure determination was performed on D8 VENTURE Kappa Duo (Bruker) with PHOTONIII detector by IµS micro-focus sealed tube with MoKα (0.71073 Å) radiation at a temperature 120(2) K. The structure was solved by intrinsic phasing (XT)[1] and refined by full matrix least squares based on F^2 (SHELXL2018).[2] The hydrogen atoms on carbon were fixed into idealized positions (riding model) and assigned temperature factors $H_{iso}(H) = 1.2\ U_{eq}$ (pivot atom). In the case of [**Dy**(**L^1**)]·3.5H_2O, the hydrogen atoms in –NH and –OH moieties were found on difference Fourier maps and refined under rigid body assumption with assigned temperature factors $H_{iso}(H) = 1.2\ U_{eq}$ (pivot atom). In the case of [**Dy**(**L^2**)]·3H_2O, some hydrogen atoms in –NH and –OH moieties were found on difference Fourier maps and others were calculated into positions most suitable for the formation of hydrogen bonds. X-ray crystallographic data have been deposited with the Cambridge Crystallographic Data Centre (CCDC) under deposition number **2072548** for [**Dy**(**L^1**)]·3.5H_2O and **2143481** for [**Dy**(**L^2**)]·3H_2O. Both can be obtained free of charge from the Centre via its website (*www.ccdc.cam.ac.uk/getstructures*).

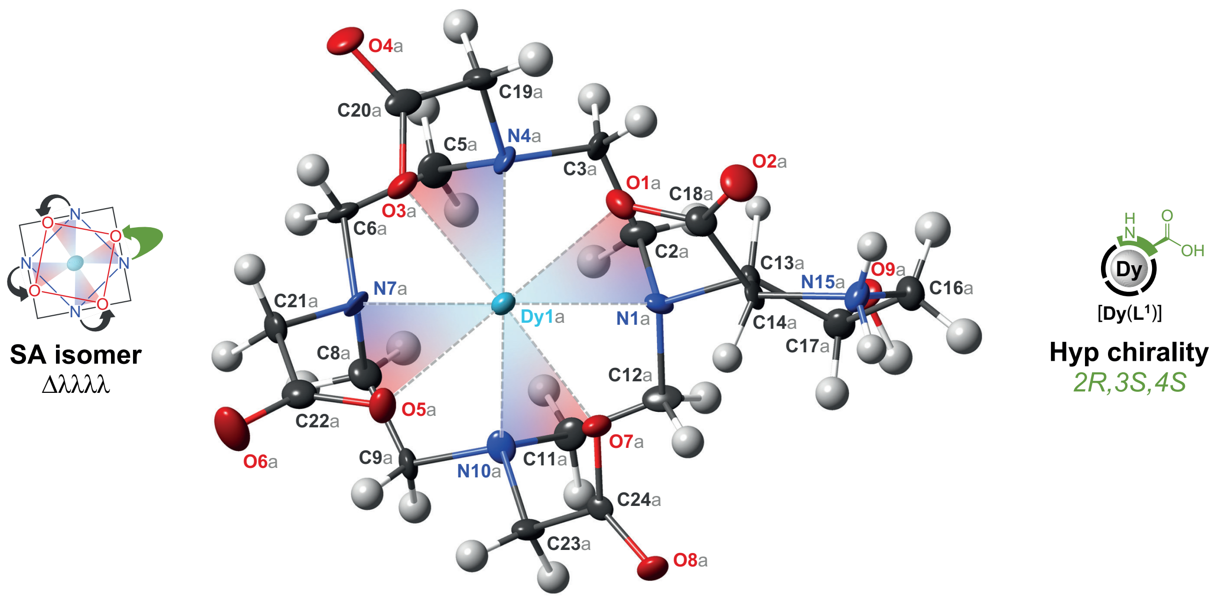

Supplementary Fig. 1. Crystallographic details of [Dy(L1)]·3.5H$_2$O. Molecular structure of one of the two zwitterionic units [**Dy**(**L**1)] found in the crystal structure of [**Dy**(**L**1)]·3.5H$_2$O (view from the top) shows *2R,3S,4S* configuration of the proline ring (Hyp) and the chelate adopts Δλλλλ square antiprismatic (SA) conformation. Thermal ellipsoids were set at 50% probability. Hydrogen atom labels were omitted for clarity reason. The presented crystal was refined as pseudomerohedric twin with twin matrix −1 0 0; 0 −1 0; 1 0 1 and volume ration of two domains 0.04619. However, crystals of [**Dy**(**L**1)]·3.5H$_2$O exhibit far more complicated diffraction pattern. Four crystals from different crystallizations were tested on diffractometer all featuring sets of additional diffractions with more or less observable diffuse scattering. Modulation or extensive twinning were considered, but no better model, than presented one, was found. In all measured crystals, the final difference Fourier map showed two high maxima of positive electron density at the same positions. These positions are too close to macrocyclic ring to have chemical meaning and could be ascribed to complicated real structure of the crystals: either stacking fault of infinite chains of Dy-complex, or extensive twinning. Crystal data for [**Dy**(**L**1)]·3.5H$_2$O: M_R = 698.0; Monoclinic, $P2_1$ (No 4), a = 13.6315 (5) Å, b = 8.0202 (2) Å, c = 24.0534 (8) Å, β = 105.875 (1)°, V = 2529.40 (14) Å^3, Z = 4, D_x = 1.833 mg m^{-3}. Needle, colourless of dimensions 0.47 × 0.07 × 0.02 mm, multi-scan absorption correction (μ = 3.03 mm^{-1}) T_{min} = 0.77, T_{max} = 0.93; a total of 33017 measured reflections (θ_{max} = 27.5°), from which 11534 were unique (R_{int} = 0.029) and 11288 observed according to the $I > 2\sigma(I)$ criterion. The refinement converged (Δ/σ_{max} = 0.002) to R = 0.039 for observed reflections and w$R(F^2)$ = 0.100, GOF = 1.10 for 677 parameters and all 11534 reflections. The final difference map displayed no peaks of chemical significance ($\Delta\rho_{max}$ = 7.00, $\Delta\rho_{min}$ −1.12 e.Å^{-3}). The determination of absolute structure was based on anomalous scattering of Dysprosium atom. Absolute structure parameter: −0.010 (4).[3]

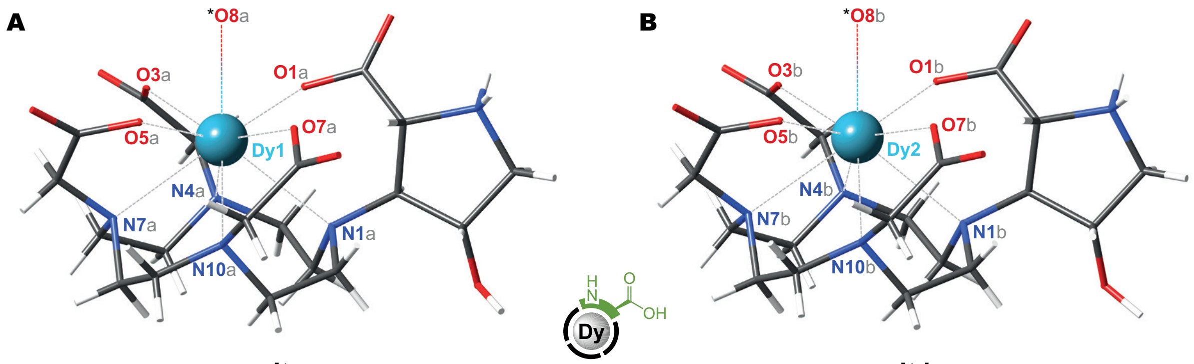

unit a	[Dy(L¹)]	unit b
**coordination bond lengths**	***difference***	**coordination bond lengths**
N1a -----Dy1 = 2.825 Å	*0.035%*	N1b -----Dy2 = 2.824 Å
N4a -----Dy1 = 2.645 Å	*0.000%*	N4b -----Dy2 = 2.645 Å
N7a -----Dy1 = 2.680 Å	*0.261%*	N7b -----Dy2 = 2.687 Å
N10a -----Dy1 = 2.642 Å	*0.113%*	N10b -----Dy2 = 2.645 Å
O1a -----Dy1 = 2.354 Å	*0.842%*	O1b -----Dy2 = 2.374 Å
O3a -----Dy1 = 2.321 Å	*0.302%*	O3b -----Dy2 = 2.314 Å
O5a -----Dy1 = 2.372 Å	*0.670%*	O5b -----Dy2 = 2.388 Å
O7a -----Dy1 = 2.312 Å	*0.865%*	O7b -----Dy2 = 2.292 Å
*O8a -----Dy1 = 2.372 Å	*0.970%*	O8b -----Dy2 = 2.349 Å
**horizontal donor atom distance**	***difference***	**horizontal donor atom distance**
N7a ⟷ N1a = 4.363 Å	*0.138%*	N7b ⟷ N1b = 4.357 Å
N10a ⟷ N4a = 4.125 Å	*0.024%*	N10b ⟷ N4b = 4.126 Å
O5a ⟷ O1a = 4.492 Å	*0.553%*	O5b ⟷ O1b = 4.517 Å
O7a ⟷ O3a = 4.449 Å	*0.607%*	O7b ⟷ O3b = 4.422 Å
**vertical donor atom distance**	***difference***	**vertical donor atom distance**
N1a ⟷ O1a = 2.942 Å	*1.242%*	N1b ⟷ O1b = 2.979 Å
N4a ⟷ O3a = 2.736 Å	*0.183%*	N4b ⟷ O3b = 2.731 Å
N7a ⟷ O5a = 2.765 Å	*0.434%*	N7b ⟷ O5b = 2.753 Å
N10a ⟷ O7a = 2.705 Å	*0.515%*	N10b ⟷ O7b = 2.719 Å
**diagonal donor atom distance**	***difference***	**diagonal donor atom distance**
N1a ⟷ O5a = 4.871 Å	*0.287%*	N1b ⟷ O5b = 4.885 Å
N4a ⟷ O7a = 4.663 Å	*0.236%*	N4b ⟷ O7b = 4.652 Å
N7a ⟷ O1a = 4.746 Å	*0.919%*	N7b ⟷ O1b = 4.790 Å
N10a ⟷ O3a = 4.621 Å	*0.390%*	N10b ⟷ O3b = 4.603 Å

Supplementary Fig. 2. Coordination environment in [Dy(L¹)]·3.5H$_2$O. The structure features two symmetrically independent units (**A**, unit a; **B**, unit b) that fit one on other almost perfectly with maximal difference of corresponding atoms 0.275 Å. The ligand is coordinated to the central Dy^{III} cation with 4 ring nitrogen donors and 4 carboxylate donors. The capping position is occupied by a carbonyl donor from neighbouring unit (denoted by asterisk) resulting in 9 coordinate chelate. Selected coordination parameters for both units are listed below their structures. Labels of atoms not involed in coordination to Dy^{III} cation were omitted for clarity reason.

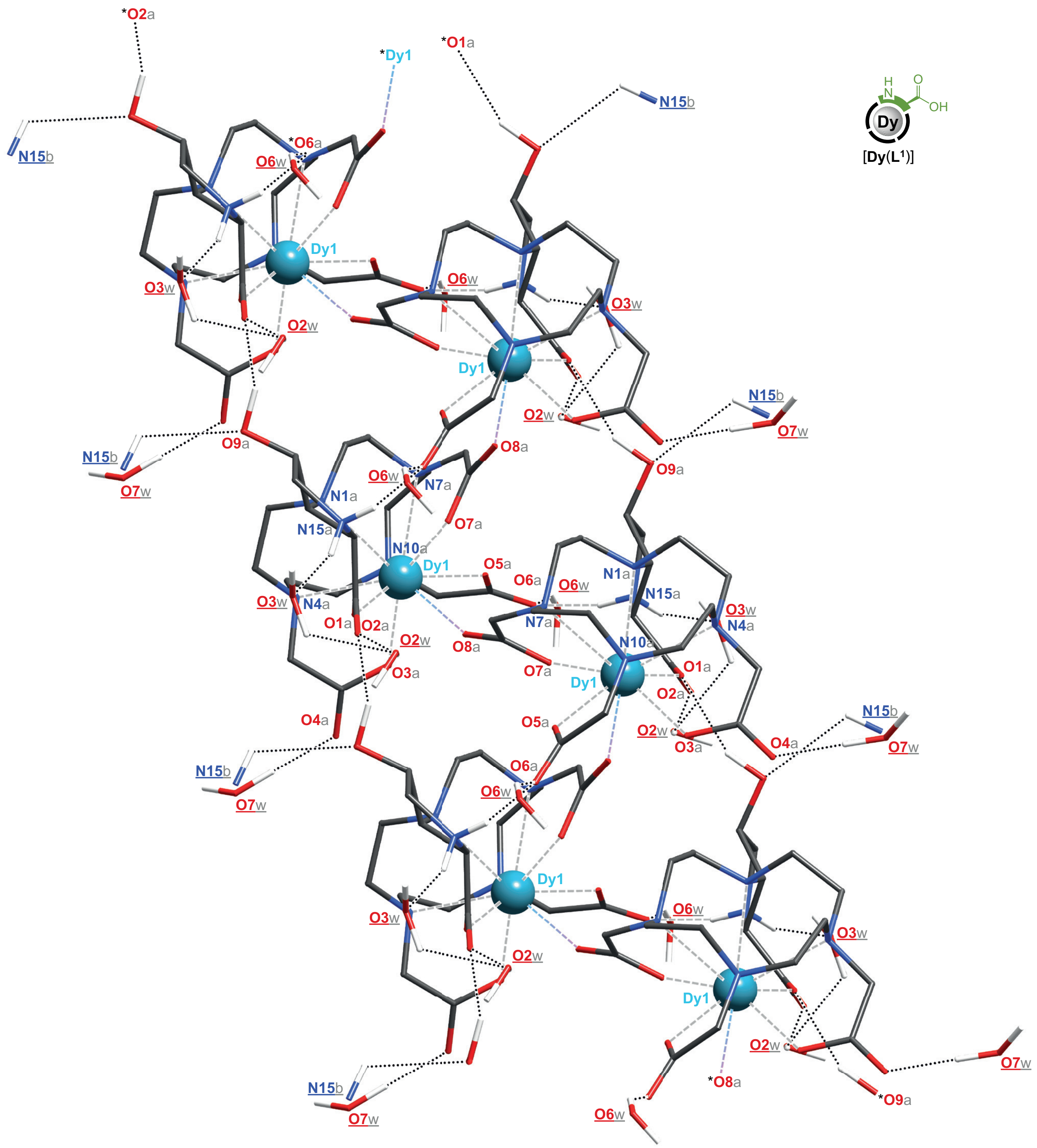

N15b—H⋯O9a hydrogen bond:	N15a—H⋯O6a hydrogen bond:	O9a—H⋯O1a hydrogen bond:	O9a—H⋯O2a hydrogen bond:
N15b ↔ H = 0.91 Å	N15a ↔ H = 0.91 Å	O9a ↔ H = 1.06 Å	O9a ↔ H = 1.06 Å
O9a ↔ H = 2.54 Å	O6a ↔ H = 1.85 Å	O1a ↔ H = 2.59 Å	O2a ↔ H = 1.90 Å
N15b ↔ O9a = 3.08 Å	N15a ↔ O6a = 2.74 Å	O9a ↔ O1a = 3.37 Å	O9a ↔ O2a = 2.88 Å
∢N15b–H⋯O9a = 118 °	∢N15a–H⋯O6a = 166 °	∢O9a–H⋯O1a = 131 °	∢O9a–H⋯O2a = 154 °

Supplementary Fig. 3. Crystal packing found in [Dy(L¹)]·3.5H₂O. One of the two coordination helixes stabilized by intermolecular coordination bond (*O8a---Dy1) and by intermolecular hydrogen bonds (dotted) directly between the units (O9a–H⋯O2a; O9a–H⋯O1a; N15a–H⋯O6a). The two helixes (each consists of symmetrically independent repeating units) are propagating along 2-fold screw axis and are stacked into parallel layers connected by hydrogen bond (N15b–H⋯O9a). Out of 7 water molecules in the structure, only 4 directly bound to displayed helix are shown (underlined). Parameters of selected hydrogen bonds are listed below. Carbon-bound hydrogens and some of the atom labels were omitted for clarity reason.

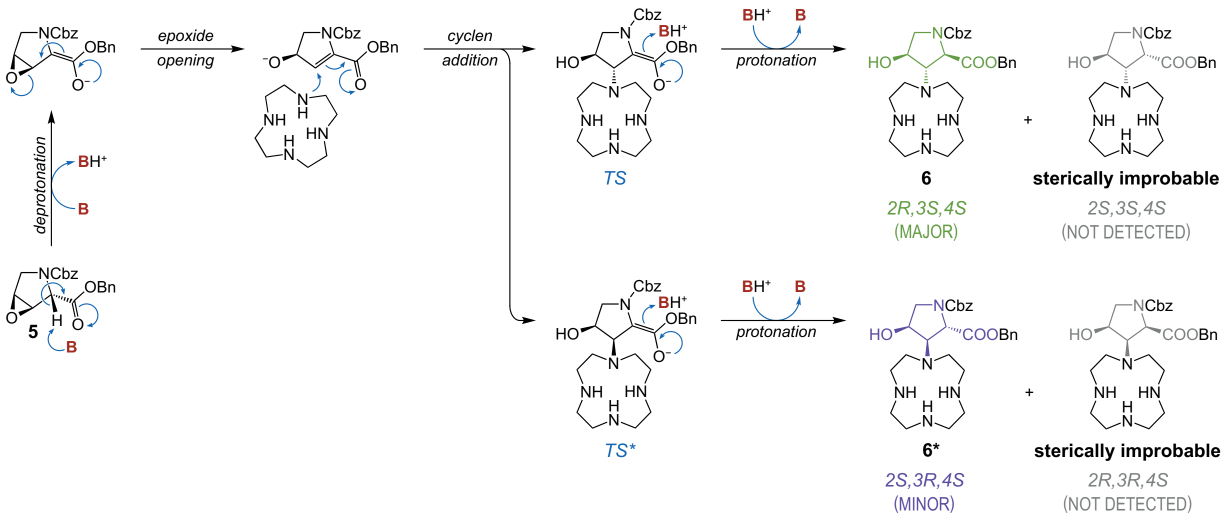

Supplementary Fig. 4. Suggested mechanism of formal ring-opening of epoxide 5 by cyclen leading to compounds 6 and 6*. Cyclen (as well as its derivatives formed during the course of the reaction) are bases strong enough to deprotonate epoxide **5** in position *2*. Intramolecular epoxide opening could then take place, allowing cyclen addition to the newly formed double bond (resulting in two transition states – *TS* and *TS**, differing in orientation of the cyclen moiety in respect to the *4S*-hydroxyl group). Subsequent protonation in the positions *2* leads to the formation of **6** and **6*** (with carboxylate and cyclen moieties pointing in opposite direction), while the two other possible products (with carboxylate and cyclen moieties pointing in the same direction) are sterically improbable.

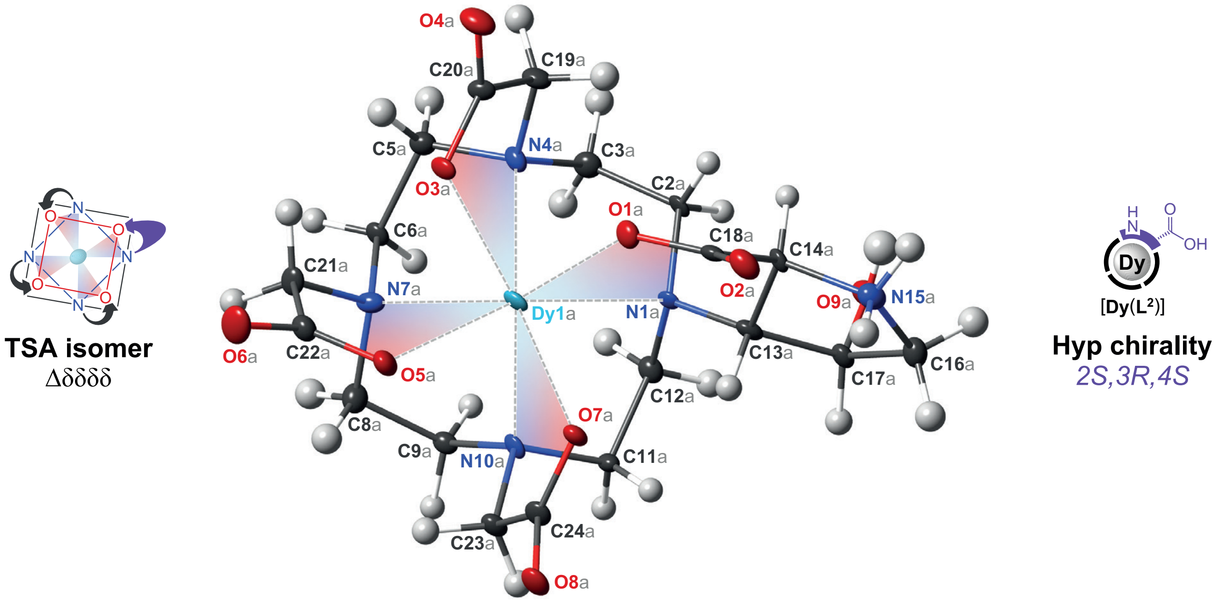

Supplementary Fig. 5. Crystallographic details of [Dy(L²)]·3H₂O. Molecular structure of one of the two zwitterionic units [**Dy**(**L**²)] found in the crystal structure of [**Dy**(**L**²)]·3H₂O (view from the top) shows *2S,3R,4S* configuration of the proline ring (Hyp) and the chelate adopts Δδδδδ twisted square antiprismatic (TSA) conformation. Thermal ellipsoids were set at 50% probability. Hydrogen atom labels were omitted for clarity reason. Altoghether nine crystals of [**Dy**(**L**²)]·3H₂O were measured, differing by a condition of crystallization, temperature of measurements and setting of diffraction experiment. All of them exhibited problematic diffraction patters, with the broad profile of diffractions along *c** reciprocal vector in most cases accompanied by multiple twinning. Since the compound crystalized in very thin plates perpendicular to *c* lattice vector, there is high probability of stacking faults of these thin layers. The real structure leads to errors in the final difference Fourier map exhibiting high maxima of residual electron density. Their positions are too close to macrocyclic ring to have chemical meaning. Crystal data for [**Dy**(**L**²)]·3H₂O: M_R = 689.0; Monoclinic, $P2_1$ (No 4), a = 7.6808 (4) Å, b = 9.1878 (5) Å, c = 34.176 (2) Å, β = 93.688 (2)°, V = 2406.8 (2) Å^3, Z = 4, D_x = 1.902 mg m^{-3}. Plate, colourless of dimensions 0.16 × 0.13 × 0.03 mm, multi-scan absorption correction (μ = 3.18 mm^{-1}) T_{min} = 0.73, T_{max} = 0.93; a total of 40689 measured reflections (θ_{max} = 27.5°), from which 10954 were unique (R_{int} = 0.039) and 10178 observed according to the $I > 2\sigma(I)$ criterion. The refinement converged (Δ/σ_{max} = 0.001) to R = 0.055 for observed reflections and w$R(F^2)$ = 0.119, GOF = 1.26 for 667 parameters and all 10954 reflections. The final difference map displayed no peaks of chemical significance ($\Delta\rho_{max}$ = 3.85, $\Delta\rho_{min}$ −2.66 e.Å^{-3}). The determination of absolute structure was based on anomalous scattering of Dysprosium atom. Absolute structure parameter: −0.010 (8).[3]

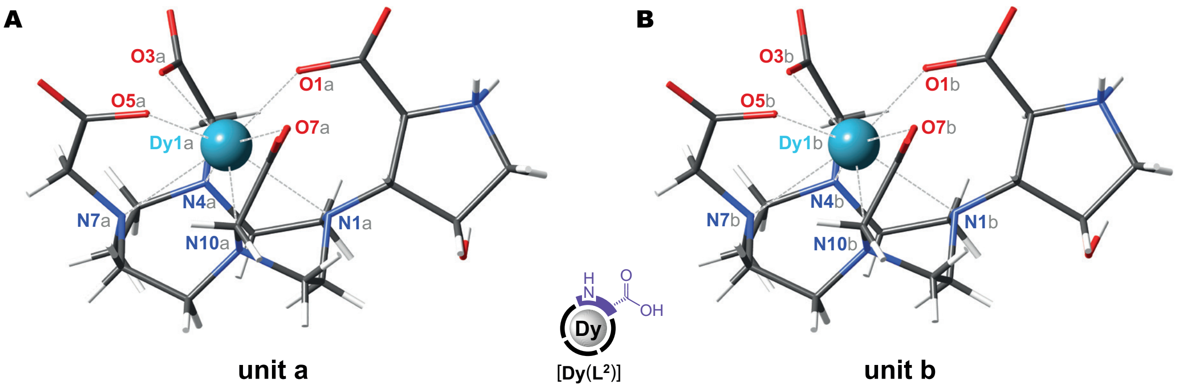

coordination bond lengths (unit a)	*difference*	coordination bond lengths (unit b)
N1a ----- Dy1a = 2.667 Å	*0.450%*	N1b ----- Dy1b = 2.655 Å
N4a ----- Dy1a = 2.544 Å	*0.039%*	N4b ----- Dy1b = 2.543 Å
N7a ----- Dy1a = 2.566 Å	*0.543%*	N7b ----- Dy1b = 2.580 Å
N10a ----- Dy1a = 2.555 Å	*1.122%*	N10b ----- Dy1b = 2.584 Å
O1a ----- Dy1a = 2.243 Å	*0.708%*	O1b ----- Dy1b = 2.259 Å
O3a ----- Dy1a = 2.330 Å	*0.086%*	O3b ----- Dy1b = 2.328 Å
O5a ----- Dy1a = 2.306 Å	*0.434%*	O5b ----- Dy1b = 2.296 Å
O7a ----- Dy1a = 2.352 Å	*0.808%*	O7b ----- Dy1b = 2.333 Å

horizontal donor atom distance (unit a)	*difference*	horizontal donor atom distance (unit b)
N7a ⟷ N1a = 4.262 Å	*0.164%*	N7b ⟷ N1b = 4.255 Å
N10a ⟷ N4a = 4.100 Å	*0.413%*	N10b ⟷ N4b = 4.117 Å
O5a ⟷ O1a = 3.937 Å	*0.025%*	O5b ⟷ O1b = 3.936 Å
O7a ⟷ O3a = 4.260 Å	*0.446%*	O7b ⟷ O3b = 4.241 Å

vertical donor atom distance (unit a)	*difference*	vertical donor atom distance (unit b)
N1a ⟷ O1a = 2.985 Å	*0.632%*	N1b ⟷ O1b = 3.004 Å
N4a ⟷ O3a = 2.644 Å	*1.159%*	N4b ⟷ O3b = 2.675 Å
N7a ⟷ O5a = 2.678 Å	*0.075%*	N7b ⟷ O5b = 2.680 Å
N10a ⟷ O7a = 2.684 Å	*0.074%*	N10b ⟷ O7b = 2.686 Å

diagonal donor atom distance (unit a)	*difference*	diagonal donor atom distance (unit b)
N1a ⟷ O5a = 4.871 Å	*0.452%*	N1b ⟷ O5b = 4.849 Å
N4a ⟷ O7a = 4.798 Å	*0.563%*	N4b ⟷ O7b = 4.771 Å
N7a ⟷ O1a = 4.700 Å	*0.655%*	N7b ⟷ O1b = 4.731 Å
N10a ⟷ O3a = 4.736 Å	*0.462%*	N10b ⟷ O3b = 4.758 Å

Supplementary Fig. 6. Coordination environment in [Dy(L²)]·3H₂O. The structure features two symmetrically independent units (**A**, unit a; **B**, unit b) that fit one on other almost perfectly with maximal difference of corresponding non-hydrogen atoms 0.082 Å. The ligand is coordinated to the central Dy^{III} cation with 4 ring nitrogen donors and 4 carboxylate donors forming 8 coordinate chelate. Selected coordination parameters for both units are listed below their structures. Labels of atoms not involed in coordination to Dy^{III} cation were omitted for clarity reason.

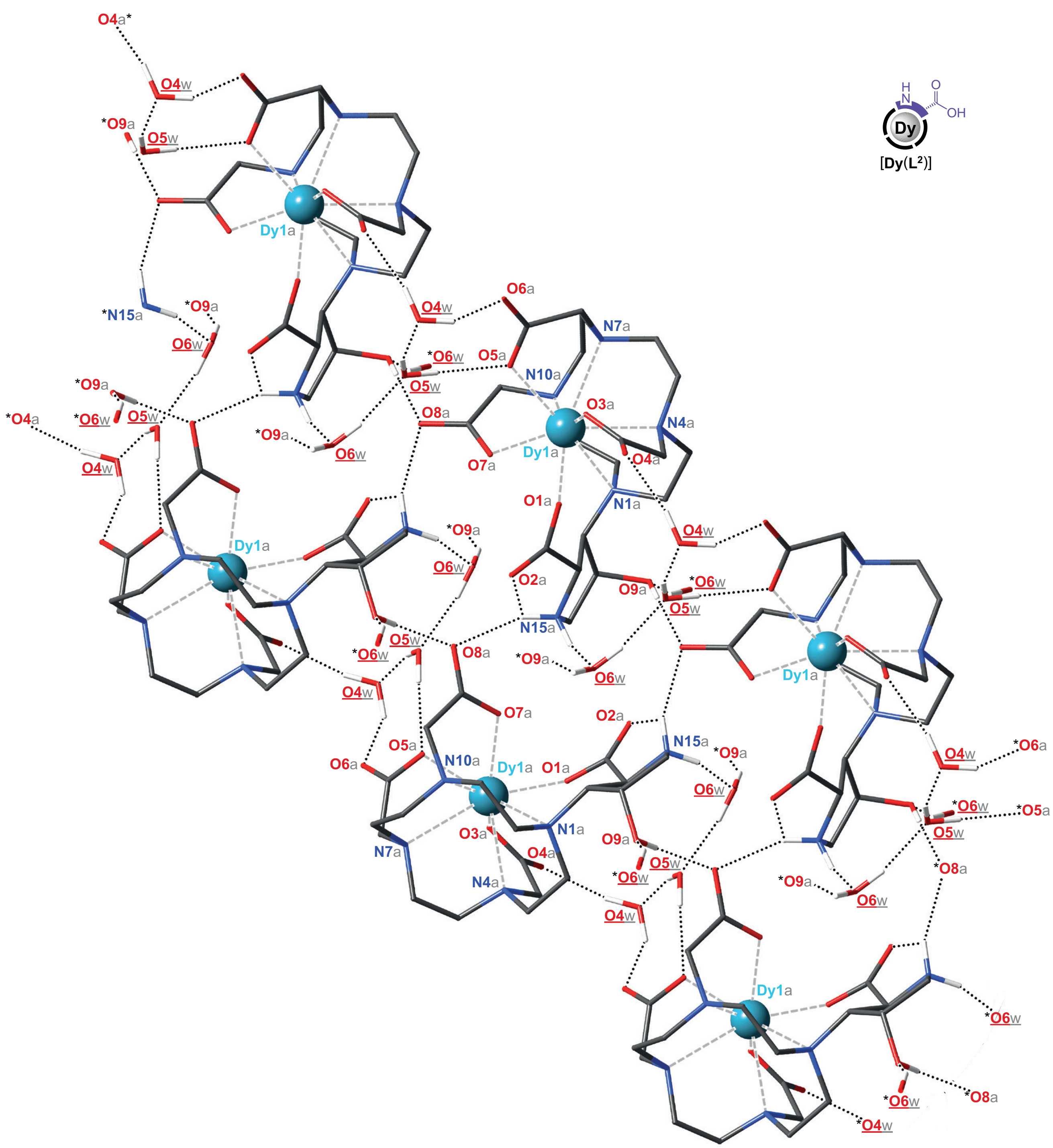

N15a—H·····O2a hydrogen bond:	N15a—H·····O9a hydrogen bond:	O9a—H·····O8a hydrogen bond:	O6w—H·····O9a hydrogen bond:
N15a ↔ H = 0.91 Å	N15a ↔ H = 0.91 Å	O9a ↔ H = 0.77 Å	O6w ↔ H = 1.05 Å
O2a ↔ H = 2.28 Å	O9a ↔ H = 2.03 Å	O8a ↔ H = 1.99 Å	O6w ↔ H = 1.87 Å
N15a ↔ O2a = 2.65 Å	N15a ↔ O9a = 2.88 Å	O9a ↔ O8a = 2.74 Å	O6w ↔ O9a = 2.87 Å
∡N15a–H···O2a = 104 °	∡N15a–H···O9a = 156 °	∡O9a–H···O8a = 167 °	∡O6w–H···O9a = 159 °

Supplementary Fig. 7. Crystal packing found in $[Dy(L^2)]\cdot 3H_2O$. One of the two columns stabilized by intermolecular hydrogen bonds between the units (O9a–H···O8a; N15a–H···O9a), intramolecular hydrogen bond (N15a–H···O2a) and by a network of interconnected water molecules. The two columns (each consists of symmetrically independent repeating units) are propagating along 2-fold screw axis and are stacked into parallel layers without apparent interaction. Out of 6 water molecules in the structure, only 3 directly bound to the displayed column are shown (underlined). Parameters of selected hydrogen bonds are listed below. Carbon-bound hydrogens and some of the atom labels were omitted for clarity reason.

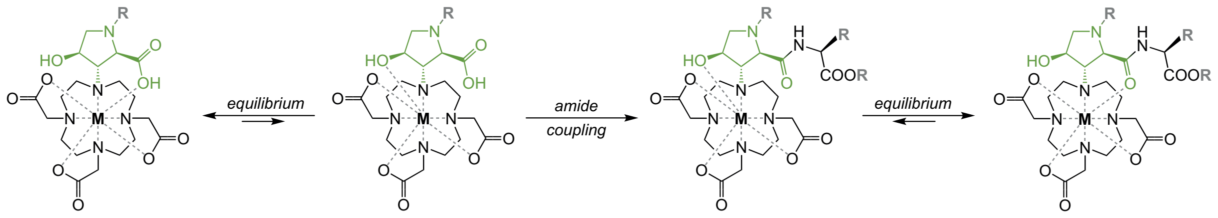

A B C D

Supplementary Fig. 8. Suggested mechanism that allows $[M(L^1)]$ building blocks to be directly incorporated into peptides via amide coupling. **A**, Expected structure of the major $[M(L^1)]$ isomer present in solution, with Hyp moiety coordinated to the central metal ion through *2R*-carboxyl rather then *4S*-hydroxyl group. **B**, Temporary de-coordination of *2R*-carboxyl group as a consequence of *4S*-hydroxyl coordination. **C**, In contrast to the coordination-protected (and hence unreactive) acetate arms, the momentarily available *2R*-carboxyl group on the Hyp moiety can undergo amide coupling. **D**, Rearrangement of the newly formed peptide product back to the thermodynamically favorable coordination state.

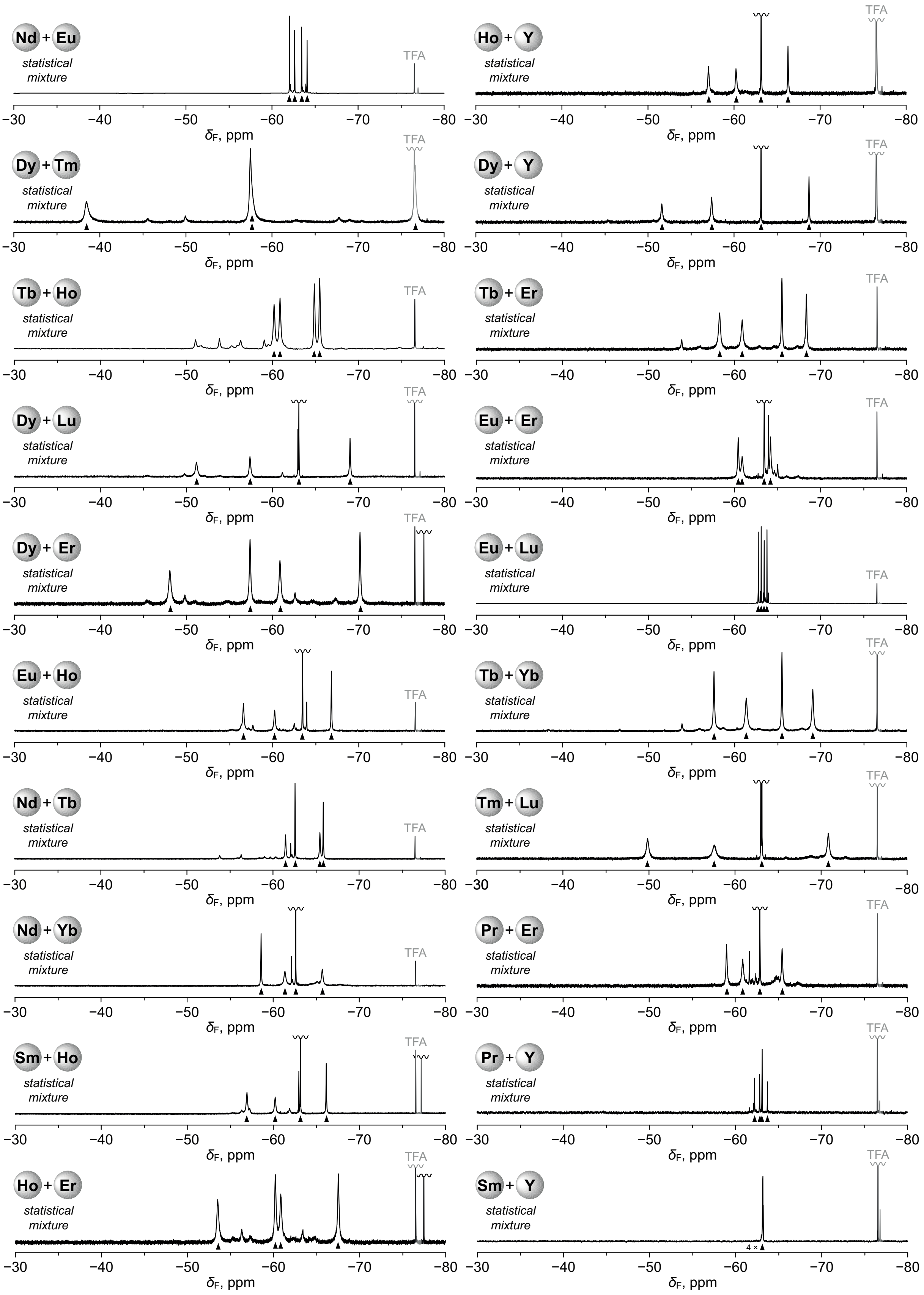

Supplementary Fig. 9. ^{19}F NMR spectroscopy (aq. MOPS/NaOH buffer, pH = 7, external D_2O for frequency lock, 470.4 MHz, T = 298.1 K) of M^1M^2-TP1 compounds prepared as statistical mixtures (in addition to Fig. 6A). The mixtures were prepared by one pot complexation of M^1 and M^2 by **TP1** and were measured without purification. Four major **M^1M^2-TP1** products are marked with triangles. Signal of TFA (trifluoroacetate; grey) originated from **TP1** purification was used as a reference (−76.55 ppm). Charges of Ln^{3+} ions were omitted for clarity reason.

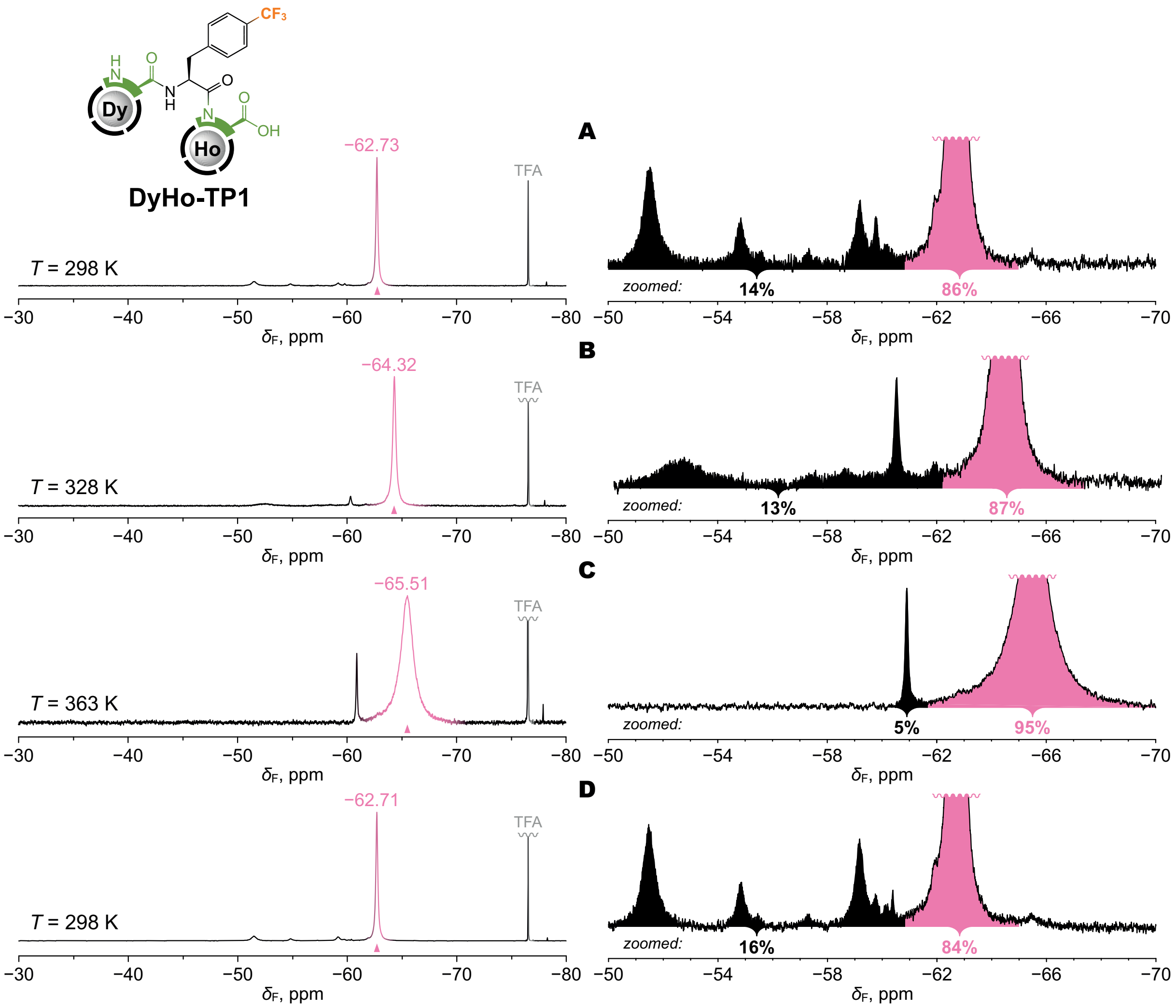

Supplementary Fig. 10. Temperature dependence of ^{19}F NMR signals (aq. MOPS/NaOH buffer, pH = 7, external D_2O for frequency lock, 470.4 MHz, *T* = 298.1 K) of DyHo-TP1 (coalescence). Compound **DyHo-TP1** was prepared by controlled synthesis, purified by preparative HPLC, and subjected to NMR measurement at increasing temperatures, before returning to the starting temperature. Left column shows full spectra, with the peak of the major species highlighted in pink colour and marked with a triangle. Right column shows spectra zoomed at the minor peaks including relative integration values in %. Charges of Ln^{3+} ions were omitted for clarity reasons. **A**, *T* = 298 K (25 °C), several minor peaks are visible. **B**, *T* = 328 K (55 °C), some minor peaks coalesced. **C**, *T* = 363 K (90 °C), all but one minor peak coalesced with the major peak. **D**, repeated measurement at *T* = 298 K (25 °C) shows recovery of the initially present minor peaks.

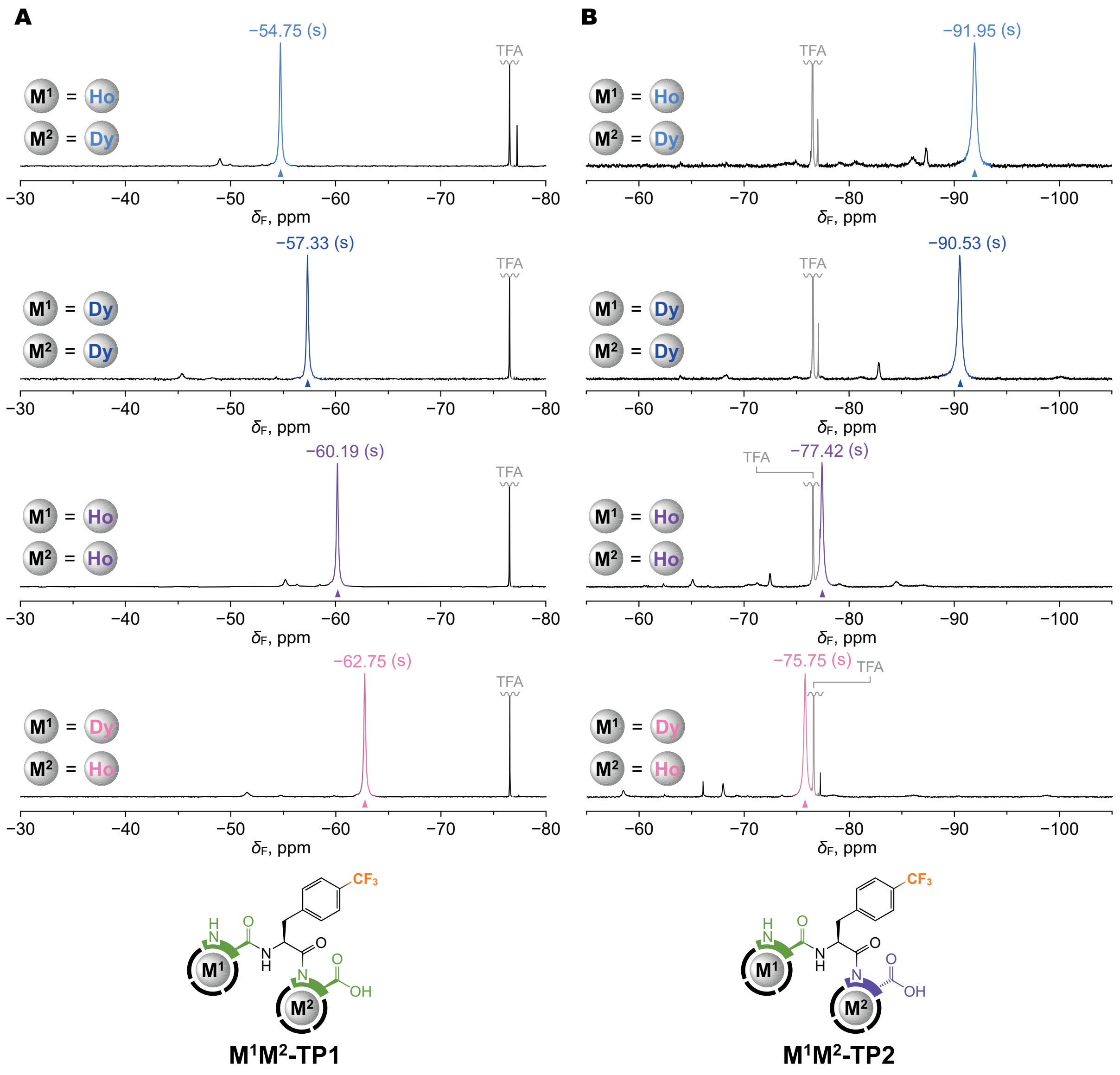

Supplementary Fig. 11. ^{19}**F NMR spectroscopy (aq. MOPS/NaOH buffer, pH = 7, external D_2O for frequency lock, 470.4 MHz, T = 298.1 K) comparison of compounds encoded with Dy^{3+}/Ho^{3+} ions. A**, **M^1M^2-TP1** based on **L^1** architecture. **B**, **M^1M^2-TP2** based on mixed **L^1/L^2** architecture. Major products are colored and marked with triangles. Charges of Ln^{3+} ions were omitted for clarity reasons.

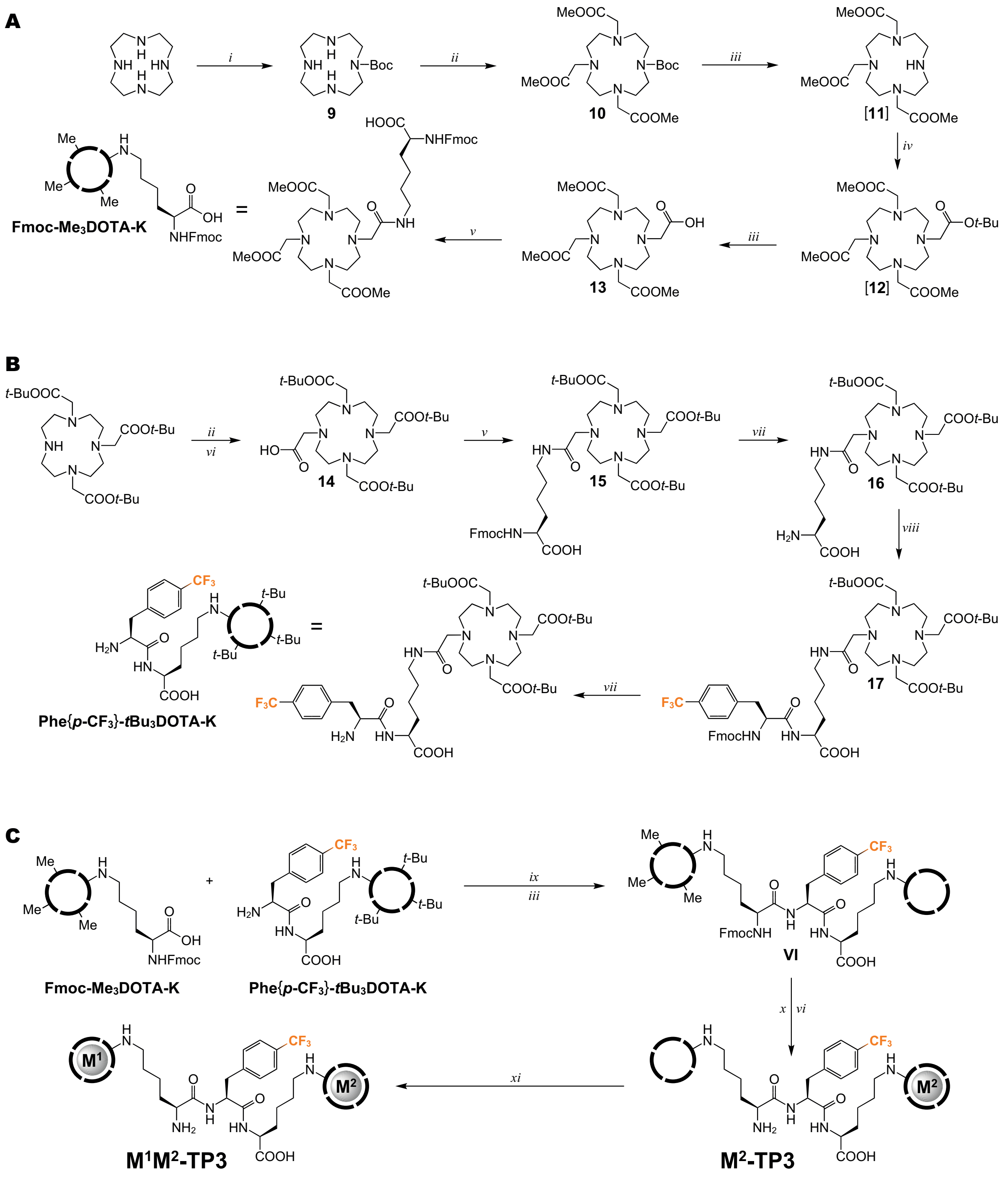

Supplementary Fig. 12. Synthesis of control tripeptides M¹M²-TP3 based on DOTA-K ligand (overview). **A**, Synthesis of building block **Fmoc-Me₃DOTA-K**. **B**, Synthesis of building block **Phe{p-CF₃}-*t*Bu₃DOTA-K**. **C**, Synthesis of **M¹M²-TP3** tripeptides. ***Conditions:*** (*i*) *tert*-butyl (4-nitrophenyl) carbonate, DCM; (*ii*) MeO_2CCH_2Br, K_2CO_3, MeCN; (*iii*) TFA; (*iv*) *t*-BuO_2CCH_2Br, K_2CO_3, MeCN; (*v*) H-Lys(Fmoc)-OH, PyAOP, DIPEA, DMSO; (*vi*) LiOH, MeOH, H_2O; (*vii*) DBU, DMF; (*viii*) Fmoc-Phe{*p*-CF_3}-OH, PyAOP, DIPEA, DMSO; (*ix*) PyAOP, DIPEA, DMSO; (*x*) M^2Cl_3, aq. MOPS/NaOH (pH 7.0); (*xi*) M^1Cl_3, aq. MOPS/NaOH (pH 7.0). Intermediates in brackets were not isolated.

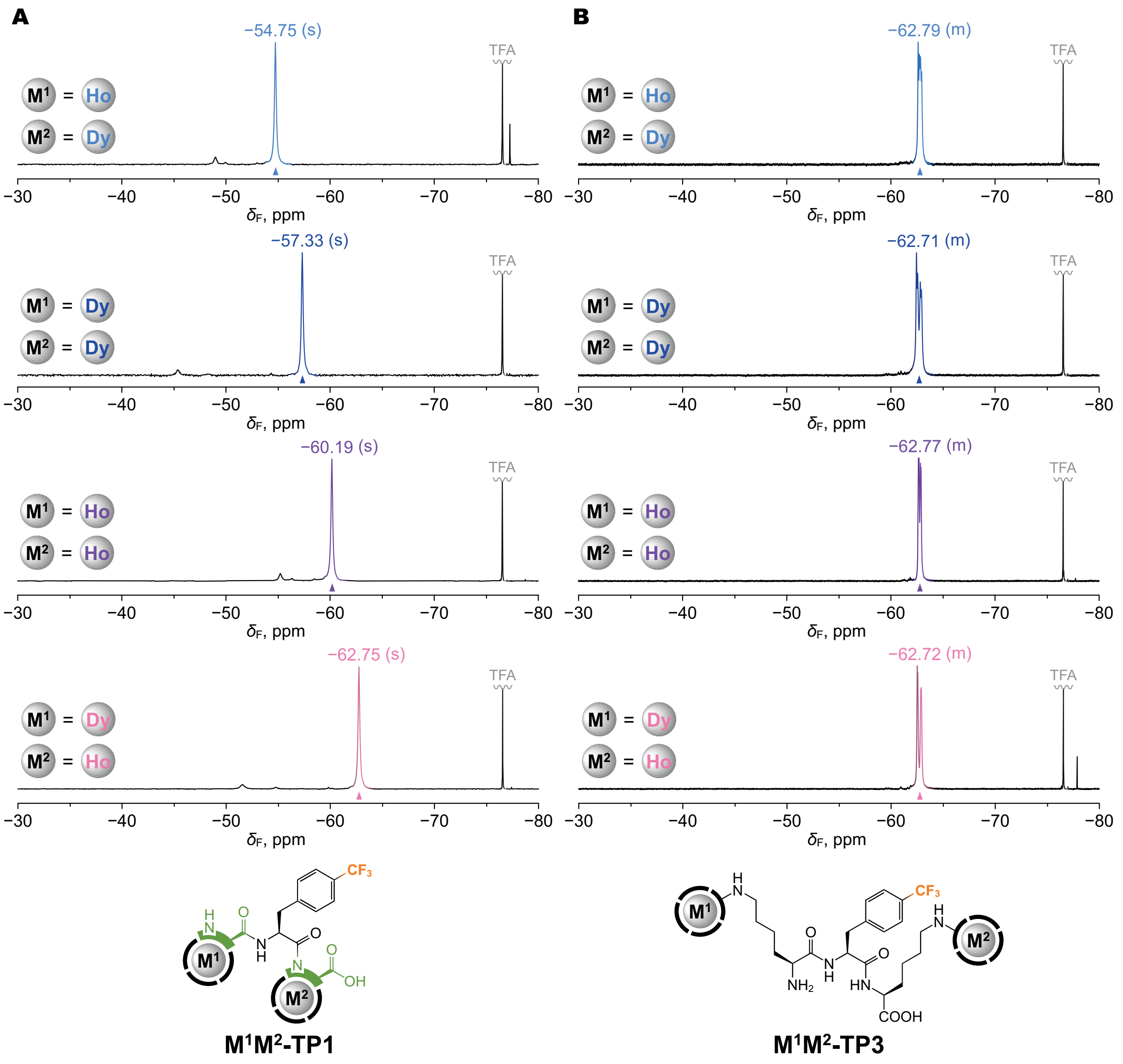

Supplementary Fig. 13. ^{19}**F NMR spectroscopy (aq. MOPS/NaOH buffer, pH = 7, external D$_2$O for frequency lock, 470.4 MHz, T = 298.1 K) comparison of compounds encoded with Dy^{3+}/Ho^{3+} ions. A**, **M^{1}M^2-TP1** based on **L^1** architecture. **B**, **M^{1}M^2-TP3** based on **DOTA-K** architecture. Major products are colored and marked with triangles. Charges of Ln^{3+} ions were omitted for clarity reasons.

A

M¹M²-TP1 system		¹⁹F chemical shift, ppm		
metals	M¹M²	δ_F	$^{sim}\delta_F$	difference
Dy + Ho statistical mixture	HoDy	−54.69	−54.75	0.06
	DyDy	−57.30	−57.30	0.00
	HoHo	−60.14	−60.17	0.03
	DyHo	−62.71	−62.72	0.01
Dy + Y statistical mixture	YDy	−51.61	−51.64	0.03
	DyDy	−57.40	−57.30	−0.10
	YY	−63.14	−63.12	−0.02
	DyY	−68.71	−68.79	0.08
Ho + Y statistical mixture	YHo	−57.04	−57.06	0.02
	HoHo	−60.24	−60.17	−0.07
	YY	−63.15	−63.12	−0.03
	HoY	−66.27	−66.24	−0.03

B

M¹M²-TP1 system		¹⁹F chemical shift, ppm		
metals	M¹M²	δ_F	$^{sim}\delta_F$	difference
Dy + Tm statistical mixture	TmDy	−38.16	−38.12	−0.04
	DyDy	−57.28	−57.32	0.04
	TmTm	−57.88	−57.74	−0.14
	DyTm	−76.96	−76.94	−0.02
Dy + Lu statistical mixture	LuDy	−51.18	−51.26	0.08
	DyDy	−57.40	−57.32	−0.08
	LuLu	−63.07	−63.04	−0.03
	DyLu	−69.03	−69.09	0.06
Tm + Lu statistical mixture	TmLu	−49.86	−49.90	0.04
	TmTm	−57.61	−57.74	0.13
	LuLu	−63.11	−63.04	−0.07
	LuTm	−70.86	−70.88	0.02

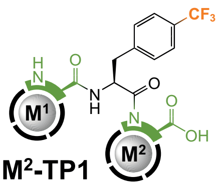

M¹M²-TP1

C

dataset	Dy/Ho, Dy/Y, Ho/Y			Dy/Tm, Dy/Lu, Tm/Lu		
¹⁹F chemical shift contributions	Dy^{3+}	Ho^{3+}	Y^{3+}	Dy^{3+}	Tm^{3+}	Lu^{3+}
δ_p^1 (paramagnetic contribution of M¹)	−5.666	−3.116	0	−6.057	13.139	0
δ_p^2 (paramagnetic contribution of M²)	11.484	6.064	0	11.777	−7.845	0
δ_d (const. diamagnetic contribution)	−63.122			−63.038		

Supplementary Fig. 14. Fitting results (two independent limited datasets) of experimental ¹⁹F NMR shifts of M¹M²-TP1 compounds. A, Dataset comprised of Dy/Ho, Dy/Y and Ho/Y statistical mixtures showing list of experimental chemical shifts (δ_F; colored Dy/Ho obtained from spectra in Fig. 6A, rest obtained from Supplementary Fig. 9) and calculated chemical shifts ($^{sim}\delta_F$) and their differences resulting from the fit. **B**, Dataset comprised of Dy/Tm, Dy/Lu and Tm/Lu statistical mixtures showing list of experimental chemical shifts (δ_F; all obtained from spectra in Supplementary Fig. 9) and calculated chemical shifts ($^{sim}\delta_F$) and their differences resulting from the fit. Charges of Ln^{3+} ions were omitted for clarity reasons. **C**, Fitted values of individual δ_p^1, δ_p^2 and δ_d parameters (in ppm) that make the overall shift ($^{sim}\delta_F = \delta_p^1 + \delta_p^2 + \delta_d$).

M^1M^2-TP1 system		^{19}F chemical shift, ppm		
metals	M^1M^2	δ_F	$^{sim}\delta_F$	difference
Dy + Ho statistical mixture	HoDy	−54.69	−54.66	−0.03
	DyDy	−57.30	−57.32	0.02
	HoHo	−60.14	−60.17	0.03
	DyHo	−62.71	−62.83	0.12
Dy + Y statistical mixture	YDy	−51.61	−51.48	−0.13
	DyDy	−57.40	−57.32	−0.08
	YY	−63.14	−63.08	−0.06
	DyY	−68.71	−68.92	0.21
Ho + Y statistical mixture	YHo	−57.04	−56.99	−0.05
	HoHo	−60.24	−60.17	−0.07
	YY	−63.15	−63.08	−0.07
	HoY	−66.27	−66.26	−0.01
Dy + Tm statistical mixture	TmDy	−38.16	−38.22	0.06
	DyDy	−57.28	−57.32	0.04
	TmTm	−57.88	−57.74	−0.14
	DyTm	−76.96	−76.84	−0.12
Dy + Lu statistical mixture	LuDy	−51.18	−51.48	0.30
	DyDy	−57.40	−57.32	−0.08
	LuLu	−63.07	−63.08	0.01
	DyLu	−69.03	−68.92	−0.11
Tm + Lu statistical mixture	TmLu	−49.86	−49.82	−0.04
	TmTm	−57.61	−57.74	0.13
	LuLu	−63.11	−63.08	−0.03
	LuTm	−70.86	−71.00	0.14
Tb + Ho statistical mixture	HoHo	−60.19	−60.17	−0.02
	TbHo	−60.88	−60.83	−0.05
	HoTb	−64.87	−64.77	−0.10
	TbTb	−65.49	−65.43	−0.06
Nd + Tb statistical mixture	NdTb	−61.50	−61.84	0.34
	NdNd	−62.60	−62.58	−0.02
	TbTb	−65.49	−65.43	−0.06
	TbNd	−65.88	−66.18	0.30
Nd + Eu statistical mixture	EuNd	−62.04	−62.02	−0.02
	NdNd	−62.63	−62.58	−0.05
	EuEu	−63.45	−63.45	0.00
	NdEu	−64.08	−64.01	−0.07

M^1M^2-TP1 system		^{19}F chemical shift, ppm		
metals	M^1M^2	δ_F	$^{sim}\delta_F$	difference
Tb + Yb statistical mixture	YbTb	−57.59	−57.68	0.09
	YbYb	−61.34	−61.40	0.06
	TbTb	−65.49	−65.43	−0.06
	TbYb	−69.07	−69.15	0.08
Ho + Er statistical mixture	ErHo	−53.54	−53.56	0.02
	HoHo	−60.23	−60.17	−0.06
	ErEr	−60.87	−60.96	0.09
	HoEr	−67.55	−67.57	0.02
Tb + Er statistical mixture	ErTb	−58.25	−58.16	−0.09
	ErEr	−60.88	−60.96	0.08
	TbTb	−65.49	−65.43	−0.06
	TbEr	−68.33	−68.23	−0.10
Eu + Er statistical mixture	ErEu	−60.43	−60.34	−0.09
	ErEr	−60.89	−60.96	0.07
	EuEu	−63.46	−63.45	−0.01
	EuEr	−64.17	−64.08	−0.09
Dy + Er statistical mixture	ErDy	−48.08	−48.05	−0.03
	DyDy	−57.39	−57.32	−0.07
	ErEr	−60.87	−60.96	0.09
	DyEr	−70.17	−70.24	0.07
Pr + Er statistical mixture	ErPr	−59.01	−58.80	−0.21
	ErEr	−60.89	−60.96	0.07
	PrPr	−62.87	−62.98	0.11
	PrEr	−65.46	−65.15	−0.31
Pr + Y statistical mixture	YPr	−62.26	−62.23	−0.03
	PrPr	−62.85	−62.98	0.13
	YY	−63.15	−63.08	−0.07
	PrY	−63.76	−63.83	0.07
Sm + Ho statistical mixture	SmHo	−56.93	−56.96	0.03
	HoHo	−60.21	−60.17	−0.04
	SmSm	−63.16	−63.10	−0.06
	HoSm	−66.15	−66.31	0.16
Sm + Y statistical mixture	SmY	−63.13	−63.13	0.00
	YY	−63.15	−63.16	0.01
	SmSm	−63.17	−60.17	0.00
	YSm	−63.21	−63.21	0.00
Eu + Lu statistical mixture	EuLu	−62.76	−62.76	0.00
	LuLu	−63.10	−63.08	−0.02
	EuEu	−63.45	−63.45	0.00
	LuEu	−63.77	−63.77	0.00
Eu + Ho statistical mixture	EuHo	−56.56	−56.67	0.11
	HoHo	−60.19	−60.17	−0.02
	EuEu	−63.44	−63.45	0.01
	HoEu	−66.79	−66.95	0.16
Nd + Yb statistical mixture	YbNd	−58.60	−58.43	−0.17
	YbYb	−61.37	−61.40	0.03
	NdNd	−62.62	−62.58	−0.04
	NdYb	−65.72	−65.55	−0.17

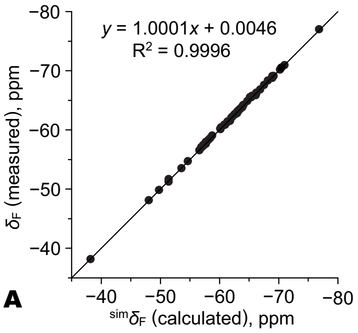

A

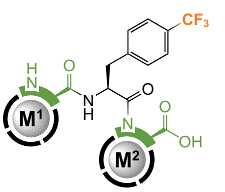

M^1M^2-TP1

B

^{19}F chemical shift contributions	Pr^{3+}	Nd^{3+}	*Sm^{3+}	Eu^{3+}	Tb^{3+}	Dy^{3+}	Ho^{3+}	Er^{3+}	Tm^{3+}	Yb^{3+}	Lu^{3+}/Y^{3+}
δ_p^1 (paramagnetic contribution of M^1)	−0.748	−0.241	0.030	0.322	−3.836	−5.838	−3.174	3.436	13.264	3.916	0
δ_p^2 (paramagnetic contribution of M^2)	0.851	0.742	−0.050	−0.689	1.488	11.601	6.089	−1.315	−7.917	−2.230	0
δ_d (const. diamagnetic contribution)						−63.083					

* Values for Sm^{3+} could not be obtained directly from the global fit due to extremely small paramagnetic effect of this ion, and were obtained from an independent fit of the Sm^{3+}/Y^{3+} statistical mixture and then used as fixed parameters in the global fit.

Supplementary Fig. 15. Fitting results (full dataset) of experimental ^{19}F NMR chemical shifts of M^1M^2-TP1 compounds. A, Complete list of experimental chemical shifts (δ_F; colored Dy/Ho obtained from spectra in Fig. 6A, rest obtained from Supplementary Fig. 9) and calculated chemical shifts ($^{sim}\delta_F$) and their differences resulting from the fit. Data plot (inset) demonstrates an excellent agreement between δ_F and $^{sim}\delta_F$. Charges of Ln^{3+} ions were omitted for clarity reasons. **B**, Fitted values of individual δ_p^1, δ_p^2 and δ_d parameters (in ppm) that make overall shift ($^{sim}\delta_F = \delta_p^1 + \delta_p^2 + \delta_d$).

A

	Pr^{3+}	Nd^{3+}	Sm^{3+}	Eu^{3+}	Tb^{3+}	Dy^{3+}	Ho^{3+}	Er^{3+}	Tm^{3+}	Yb^{3+}
δ_p^1 (contribution of M^1)	−0.748	−0.241	0.030	0.322	−3.836	−5.838	−3.174	3.436	13.264	3.916
δ_p^2 (contribution of M^2)	0.851	0.742	−0.050	−0.689	1.488	11.601	6.089	−1.315	−7.917	−2.230
C_j (Bleaney constant)[4]	−11	−4.2	−0.7	4	−86	−100	−39	33	53	22

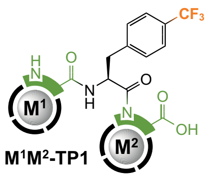

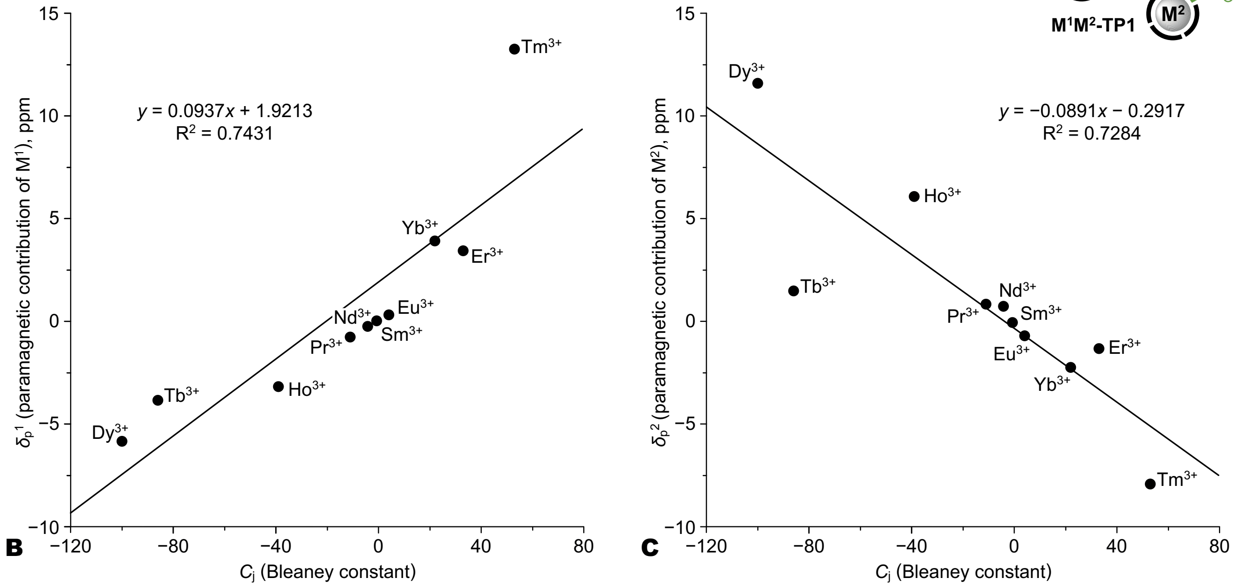

Supplementary Fig. 16. Comparison of pseudocontact paramagnetic shifts with Bleaney constants. **A**, Table of pseudocontact paramagnetic shift contributions δ_p^1, δ_p^2 obtained for metal ions in positions M^1 and M^2 in the **M¹M²-TP1** system (same as in Supplementary Fig. 15B) and corresponding Bleaney constants (C_j) for the metal ions.[4] **B**, Plot of δ_p^1 vs. C_j. **C**, Plot of δ_p^2 vs. C_j. The plots show reasonable agreement of the experimentally obtained shifts with theory.

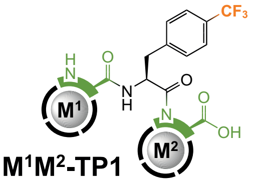

M² \ M¹	Pr	Nd	Sm	Eu	Tb	Dy	Ho	Er	Tm	Yb	Lu/Y
Pr	−62.98	−62.47	−62.20	−61.91	−66.07	−68.07	−65.41	−58.80	−48.97	−58.32	−62.23
Nd	−63.09	−62.58	−62.31	−62.02	−66.18	−68.18	−65.52	−58.91	−49.08	−58.43	−62.34
Sm	−63.88	−63.37	−63.10	−62.81	−66.97	−68.97	−66.31	−59.70	−49.87	−59.22	−63.13
Eu	−64.52	−64.01	−63.74	−63.45	−67.61	−69.61	−66.95	−60.34	−50.51	−59.86	−63.77
Tb	−62.34	−61.84	−61.57	−61.27	−65.43	−67.43	−64.77	−58.16	−48.33	−57.68	−61.60
Dy	−52.23	−51.72	−51.45	−51.16	−55.32	−57.32	−54.66	−48.05	−38.22	−47.57	−51.48
Ho	−57.74	−57.23	−56.96	−56.67	−60.83	−62.83	−60.17	−53.56	−43.73	−53.08	−56.99
Er	−65.15	−64.64	−64.37	−64.08	−68.23	−70.24	−67.57	−60.96	−51.13	−60.48	−64.40
Tm	−71.75	−71.24	−70.97	−70.68	−74.84	−76.84	−74.17	−67.56	−57.74	−67.08	−71.00
Yb	−66.06	−65.55	−65.28	−64.99	−69.15	−71.15	−68.49	−61.88	−52.05	−61.40	−65.31
Lu/Y	−63.83	−63.32	−63.05	−62.76	−66.92	−68.92	−66.26	−59.65	−49.80	−59.17	−63.08

Supplementary Fig. 17. Simulated ^{19}F NMR chemical shifts ($^{sim}\delta_F$) for M^1M^2-TP1 compounds. Values were calculated using $\delta_p{}^1$, $\delta_p{}^2$ and δ_d parameters from Supplementary Fig. 15B (charges of Ln^{3+} ions were omitted for clarity reasons). Subset of Ln^{3+} that provide overlap-free $^{sim}\delta_F$ is highlighted.

metals (M^1M^2-TP1 system)	M^1M^2	δ_F (^{19}F chemical shift, ppm)	$^{sim}\delta_F$	difference
Dy + Ho statistical mixture	HoDy	excluded		
	DyDy	excluded		
	HoHo	excluded		
	DyHo	excluded		
Dy + Y statistical mixture	YDy	−51.61	−51.46	−0.15
	DyDy	−57.40	−57.33	−0.07
	YY	−63.14	−63.08	−0.06
	DyY	−68.71	−68.95	0.24
Ho + Y statistical mixture	YHo	−57.04	−57.02	−0.02
	HoHo	−60.24	−60.16	−0.08
	YY	−63.15	−63.08	−0.07
	HoY	−66.27	−66.23	−0.04
Dy + Tm statistical mixture	TmDy	−38.16	−38.21	0.05
	DyDy	−57.28	−57.33	0.05
	TmTm	−57.88	−57.73	−0.15
	DyTm	−76.96	−76.85	−0.11
Dy + Lu statistical mixture	LuDy	−51.18	−51.46	0.28
	DyDy	−57.40	−57.33	−0.07
	LuLu	−63.07	−63.08	0.01
	DyLu	−69.03	−68.95	−0.08
Tm + Lu statistical mixture	TmLu	−49.86	−49.83	−0.03
	TmTm	−57.61	−57.73	0.12
	LuLu	−63.11	−63.08	−0.03
	LuTm	−70.86	−70.99	0.13
Tb + Ho statistical mixture	HoHo	excluded		
	TbHo	excluded		
	HoTb	excluded		
	TbTb	excluded		
Nd + Tb statistical mixture	NdTb	−61.50	−61.81	0.31
	NdNd	−62.60	−62.58	−0.02
	TbTb	−65.49	−65.40	−0.09
	TbNd	−65.88	−66.16	0.28
Nd + Eu statistical mixture	EuNd	−62.04	−62.00	−0.04
	NdNd	−62.63	−62.58	−0.05
	EuEu	−63.45	−63.45	0.00
	NdEu	−64.08	−64.02	−0.06

metals (M^1M^2-TP1 system)	M^1M^2	δ_F (^{19}F chemical shift, ppm)	$^{sim}\delta_F$	difference
Tb + Yb statistical mixture	YbTb	excluded		
	YbYb	excluded		
	TbTb	excluded		
	TbYb	excluded		
Ho + Er statistical mixture	ErHo	−53.54	−53.59	0.05
	HoHo	−60.23	−60.16	−0.07
	ErEr	−60.87	−60.96	0.09
	HoEr	−67.55	−67.54	−0.01
Tb + Er statistical mixture	ErTb	−58.25	−58.13	−0.12
	ErEr	−60.88	−60.96	0.08
	TbTb	−65.49	−65.40	−0.09
	TbEr	−68.33	−68.23	−0.10
Eu + Er statistical mixture	ErEu	−60.43	−60.34	−0.09
	ErEr	−60.89	−60.96	0.07
	EuEu	−63.46	−63.45	−0.01
	EuEr	−64.17	−64.07	−0.10
Dy + Er statistical mixture	ErDy	−48.08	−48.03	−0.05
	DyDy	−57.39	−57.33	−0.06
	ErEr	−60.87	−60.96	0.09
	DyEr	−70.17	−70.26	0.09
Pr + Er statistical mixture	ErPr	−59.01	−58.80	−0.21
	ErEr	−60.89	−60.96	0.07
	PrPr	−62.87	−62.98	0.11
	PrEr	−65.46	−65.15	−0.31
Pr + Y statistical mixture	YPr	−62.26	−62.23	−0.03
	PrPr	−62.85	−62.98	0.13
	YY	−63.15	−63.08	−0.07
	PrY	−63.76	−63.83	0.07
Sm + Ho statistical mixture	SmHo	−56.93	−56.99	0.06
	HoHo	−60.21	−60.16	−0.05
	SmSm	−63.16	−63.10	−0.06
	HoSm	−66.15	−66.28	0.13
Sm + Y statistical mixture	SmY	−63.13	−63.13	0.00
	YY	−63.15	−63.16	0.01
	SmSm	−63.17	−60.17	0.00
	YSm	−63.21	−63.21	0.00
Eu + Lu statistical mixture	EuLu	−62.76	−62.76	0.00
	LuLu	−63.10	−63.08	−0.02
	EuEu	−63.45	−63.45	0.00
	LuEu	−63.77	−63.78	0.01
Eu + Ho statistical mixture	EuHo	−56.56	−56.69	0.13
	HoHo	−60.19	−60.16	−0.03
	EuEu	−63.44	−63.45	0.01
	HoEu	−66.79	−66.92	0.13
Nd + Yb statistical mixture	YbNd	−58.60	−58.48	−0.12
	YbYb	−61.37	−61.50	0.13
	NdNd	−62.62	−62.58	−0.04
	NdYb	−65.72	−65.60	−0.12

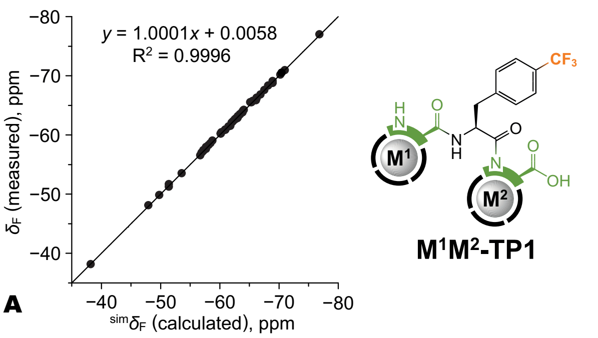

B

^{19}F chemical shift contributions	Pr^{3+}	Nd^{3+}	*Sm^{3+}	Eu^{3+}	Tb^{3+}	Dy^{3+}	Ho^{3+}	Er^{3+}	Tm^{3+}	Yb^{3+}	Lu^{3+}/Y^{3+}
δ_p^1 (paramagnetic contribution of M^1)	−0.753	−0.246	0.030	0.325	−3.834	−5.867	−3.147	3.432	13.253	3.852	0
δ_p^2 (paramagnetic contribution of M^2)	0.853	0.753	−0.050	−0.694	1.519	11.617	6.064	−1.315	−7.905	−2.267	0
δ_d (const. diamagnetic contribution)	−63.080										

* Values for Sm^{3+} could not be obtained directly from the global fit due to extremely small paramagnetic effect of this ion, and were obtained from an independent fit of the Sm^{3+}/Y^{3+} statistical mixture and then used as fixed parameters in the global fit.

Supplementary Fig. 18. Fitting results (reduced dataset) of experimental ^{19}F NMR chemical shifts of M^1M^2-TP1 compounds. A, Reduced list of experimental chemical shifts (δ_F; obtained from spectra in Supplementary Fig. 9) and calculated chemical shifts ($^{sim}\delta_F$) and their differences resulting from the fit. Data plot (inset) demonstrates an excellent agreement between δ_F and $^{sim}\delta_F$. Charges of Ln^{3+} ions were omitted for clarity reasons. **B**, Fitted values of individual δ_p^1, δ_p^2 and δ_d parameters (in ppm) that make overall shift ($^{sim}\delta_F = \delta_p^1 + \delta_p^2 + \delta_d$).

A

$^{sim}\delta_F$ matrix (from full dataset)

M² \ M¹	Tb	Dy	Ho	Yb
Tb	−65.43	−67.43	−64.77	−57.68
Dy	−55.32	−57.32	−54.66	−47.57
Ho	−60.83	−62.83	−60.17	−53.08
Yb	−69.15	−71.15	−68.49	−61.40

B

$^{sim}\delta_F$ matrix (from reduced dataset)

M² \ M¹	Tb	Dy	Ho	Yb
Tb	−65.40	−67.43	−64.71	−57.71
Dy	−55.30	−57.33	−54.61	−47.61
Ho	−60.85	−62.88	−60.16	−53.16
Yb	−69.18	−71.21	−68.49	−61.50

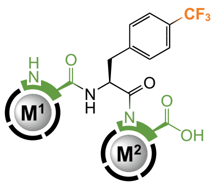

M¹M²-TP1

C

δ_F = −47.38 ppm → M¹M² = **YbDy**

M² \ M¹	Tb	Dy	Ho	Yb
Tb	18.02	20.05	17.33	10.33
Dy	7.92	9.95	7.23	**0.23**
Ho	13.47	15.50	12.78	5.78
Yb	21.80	23.83	21.11	14.12

δ_F = −53.02 ppm → M¹M² = **YbHo**

M² \ M¹	Tb	Dy	Ho	Yb
Tb	12.38	14.41	11.69	4.69
Dy	2.28	4.31	1.59	5.41
Ho	7.83	9.86	7.14	**0.14**
Yb	16.16	18.19	15.47	8.48

δ_F = −54.75 ppm → M¹M² = **HoDy**

M² \ M¹	Tb	Dy	Ho	Yb
Tb	10.65	12.68	9.96	2.96
Dy	0.55	2.58	**0.14**	7.14
Ho	6.10	8.13	5.41	1.59
Yb	14.43	16.46	13.74	6.75

δ_F = −55.55 ppm → M¹M² = **TbDy**

M² \ M¹	Tb	Dy	Ho	Yb
Tb	9.85	11.88	9.16	2.16
Dy	**0.25**	1.78	0.94	7.94
Ho	5.30	7.33	4.61	2.39
Yb	13.63	15.66	12.94	5.95

δ_F = −57.56 ppm → M¹M² = **YbTb**

M² \ M¹	Tb	Dy	Ho	Yb
Tb	7.84	9.87	7.15	**0.15**
Dy	2.26	0.23	2.95	9.95
Ho	3.29	5.32	2.60	4.40
Yb	11.62	13.65	10.93	3.94

δ_F = −60.91 ppm → M¹M² = **TbHo**

M² \ M¹	Tb	Dy	Ho	Yb
Tb	4.49	6.52	3.80	3.20
Dy	5.61	3.58	6.30	13.30
Ho	**0.06**	1.97	0.75	7.75
Yb	8.27	10.30	7.58	0.59

δ_F = −62.75 ppm → M¹M² = **DyHo**

M² \ M¹	Tb	Dy	Ho	Yb
Tb	2.65	4.68	1.96	5.04
Dy	7.45	5.42	8.14	15.14
Ho	1.90	**0.13**	2.59	9.59
Yb	6.43	8.46	5.74	1.25

δ_F = −64.81 ppm → M¹M² = **HoTb**

M² \ M¹	Tb	Dy	Ho	Yb
Tb	0.59	2.62	**0.10**	7.10
Dy	9.51	7.48	10.20	17.20
Ho	3.96	1.93	4.65	11.65
Yb	4.37	6.40	3.68	3.31

δ_F = −67.29 ppm → M¹M² = **DyTb**

M² \ M¹	Tb	Dy	Ho	Yb
Tb	1.89	**0.14**	2.58	9.58
Dy	11.99	9.96	12.68	19.68
Ho	6.44	4.41	7.13	14.13
Yb	1.89	3.92	1.20	5.79

δ_F = −68.39 ppm → M¹M² = **HoYb**

M² \ M¹	Tb	Dy	Ho	Yb
Tb	2.99	0.96	3.68	10.68
Dy	13.09	11.06	13.78	20.78
Ho	7.54	5.51	8.23	15.23
Yb	0.79	2.82	**0.10**	6.89

δ_F = −69.10 ppm → M¹M² = **TbYb**

M² \ M¹	Tb	Dy	Ho	Yb
Tb	3.70	1.67	4.39	11.39
Dy	13.80	11.77	14.49	21.49
Ho	9.25	6.22	8.94	15.94
Yb	**0.08**	2.11	0.61	7.60

δ_F = −71.04 ppm → M¹M² = **DyYb**

M² \ M¹	Tb	Dy	Ho	Yb
Tb	5.64	3.61	6.33	13.33
Dy	15.74	13.71	16.43	23.43
Ho	10.19	8.16	10.88	17.88
Yb	1.86	**0.17**	2.55	9.54

Comparision of experimental shifts δ_F against simulated shifts $^{sim}\delta_F$ from reduced dataset matrix:

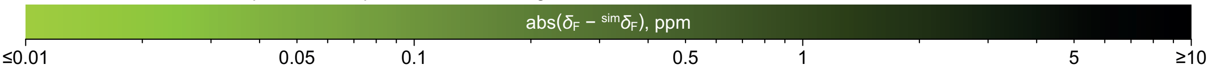

Supplementary Fig. 19. Decoding of M¹M² sequences from experimental chemical shifts (δ_F) of M¹M²-TP1 compounds containing Tb^{3+}, Dy^{3+}, Ho^{3+} and Yb^{3+} ions. **A**, Matrix of simulated ^{19}F NMR chemical shifts ($^{sim}\delta_F$) taken from Supplementary Fig. 17. **B**, Matrix of simulated ^{19}F NMR chemical shifts ($^{sim}\delta_F$) using unbiased δ_p^1, δ_p^2 and δ_d values from Supplementary Fig. 18B. **C**, Decoding matrices showing the differences between simulated shifts $^{sim}\delta_F$ (from reduced dataset matrix) and experimental shifts of the tested compound (δ_F) calculated as abs($^{sim}\delta_F - \delta_F$). The lowest difference reveals the identity of M¹ and M². Charges of Ln^{3+} ions were omitted for clarity reasons.

A

M¹ =	Yb	Yb	Ho	Tb	Dy	Yb	Ho	Tb	Yb	Dy	Ho	Tb	Dy	Ho	Tb	Dy
M² =	Dy	Ho	Dy	Dy	Dy	Tb	Ho	Ho	Yb	Ho	Tb	Tb	Tb	Yb	Yb	Yb
$^{sim}\delta_F$, ppm	−47.61	−53.16	−54.61	−55.30	−57.33	−57.71	−60.16	−60.85	−61.50	−62.88	−64.71	−65.40	−67.43	−68.49	−69.18	−71.21
δ_F, ppm	−47.38	−53.02	−54.75	−55.55	−57.33	−57.56	−60.19	−60.91	−61.37	−62.75	−64.81	−65.39	−67.29	−68.39	−69.10	−71.04
difference	−0.23	−0.14	0.14	0.25	0.00	−0.15	0.03	0.06	−0.13	−0.13	0.10	−0.01	−0.14	−0.10	−0.08	−0.17

B

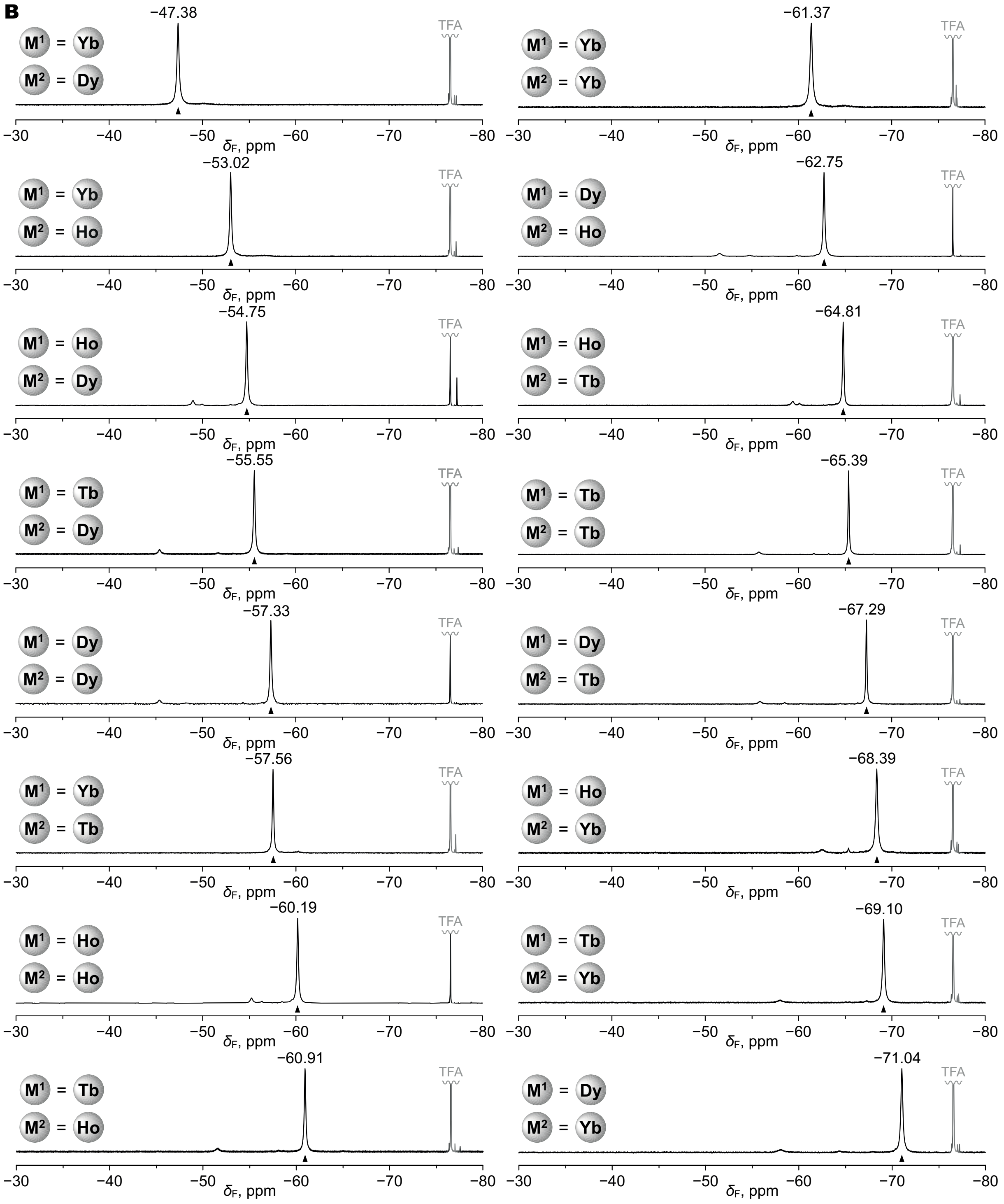

Supplementary Fig. 20. ^{19}F NMR spectroscopy (aq. MOPS/NaOH buffer, pH = 7, external D_2O for frequency lock, 470.4 MHz, *T* = 298.1 K) of all 16 possible M¹M²-TP1 compounds encoded with Tb^{3+}, Dy^{3+}, Ho^{3+} and Yb^{3+} ions. A, Table of simulated ($^{sim}\delta_F$, unbiased values from Supplementary Fig. 19B) and experimental (δ_F, from panel B) ^{19}F NMR chemical shifts, and their differences. **B**, Measured ^{19}F NMR spectra (referenced to TFA = −76.55 ppm) of **M¹M²-TP1** compounds prepared individually by controlled synthesis. Major products are marked with triangles. Charges of Ln^{3+} ions were omitted for clarity reasons.

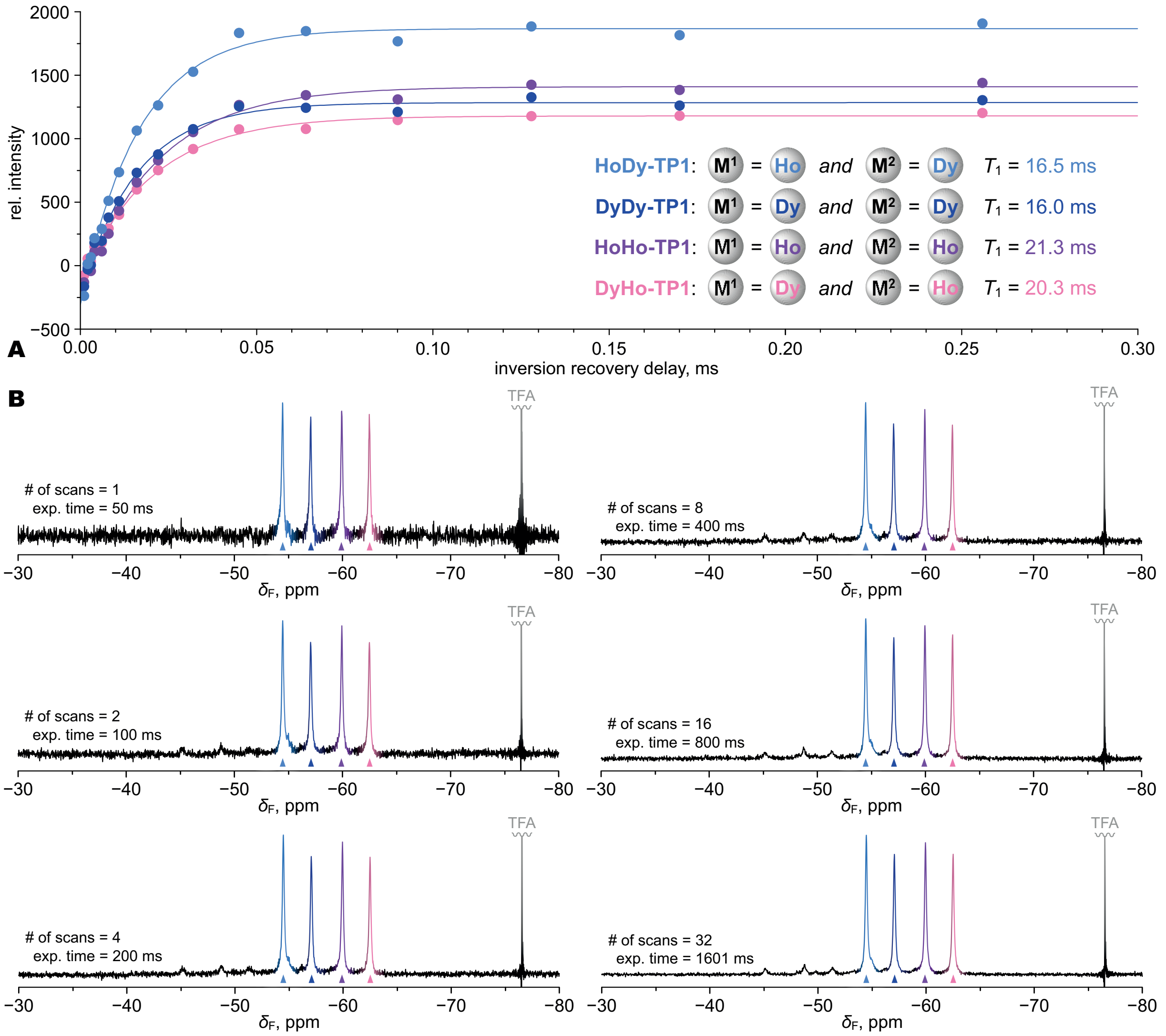

C

# of scans<br>exp. time, ms	1<br>50	2<br>100	4<br>200	8<br>400	16<br>800	32<br>1601
**HoDy-TP1**	SNR = 16.8	SNR = 26.7	SNR = 35.5	SNR = 55.2	SNR = 71.7	SNR = 107.0
**DyDy-TP1**	SNR = 15.0	SNR = 21.7	SNR = 29.8	SNR = 46.6	SNR = 62.6	SNR = 92.8
**HoHo-TP1**	SNR = 15.5	SNR = 24.1	SNR = 34.1	SNR = 52.2	SNR = 68.1	SNR = 101.5
**DyHo-TP1**	SNR = 14.7	SNR = 21.9	SNR = 29.5	SNR = 46.3	SNR = 63.3	SNR = 92.8

D

compound	exp. time, s	# of scans	amount, nmol	SNR	detection limit (SNR = 3), nmol
**DyHo-TP1**	1.6	32	180	92.8	5.819
	3600	72000	180	4401.9	0.123

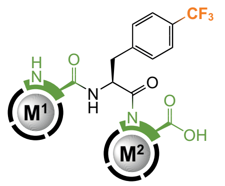

M1M2-TP1

Supplementary Fig. 21. Signal-to-noise (SNR) measurements for ^{19}F NMR signals of HoDy-TP1, DyDy-TP1, HoHo-TP1 and DyHo-TP1 (aq. MOPS/NaOH buffer, pH = 7, external D_2O for frequency lock, 470.4 MHz, *T* = 298.1 K). A, Fit of longitudinal relaxation (T_1) values from inversion recovery experiment of a mixture prepared from stock solutions of the individual compounds. The concentration (3.6 mM) and the observed volume (~50 µL) correspond to 0.18 µmol of each compound being measured in total. Charges of Ln^{3+} ions were omitted for clarity reasons. **B**, ^{19}F NMR spectra obtained with 1, 2, 4, 8, 16 and 32 scans (90° observation pulse, pre-acquisition delay = 0, no apodization used). Major products are colored and marked with triangles. **C**, Table of SNR values for the corresponding compounds and number of scans. **D**, Extrapolation of the detection limit at SNR = 3 (extrapolated numbers are highlighted green) for different number of scans and experiment times.

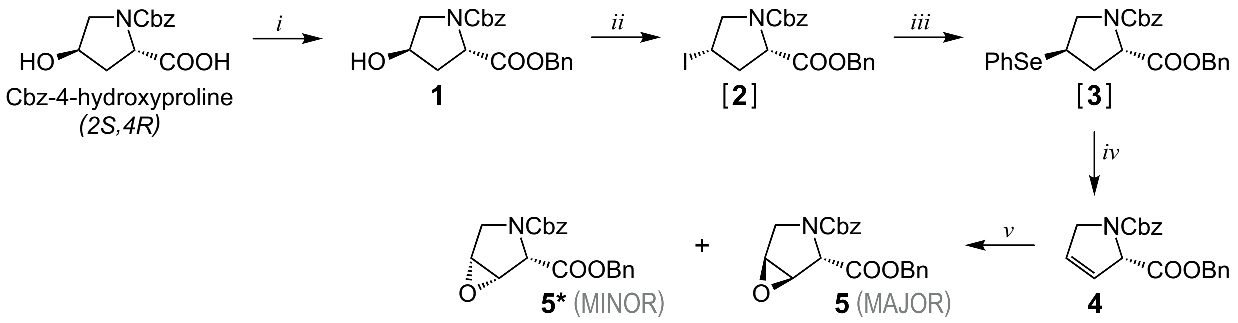

Supplementary Fig. 22. Synthesis of epoxide intermediates 5 and 5*. ***Conditions:*** (*i*) BnBr, TEA, THF; (*ii*) PPh_3, THF, 0 °C *followed by* DIAD, MeI, 0 °C → RT; (*iii*) $(PhSe)_2$, EtOH, $NaBH_4$, 0 °C → RT; (*iv*) H_2O_2, THF, 0 °C → RT; (*v*) MCPBA, $CHCl_3$, 85 °C. Intermediates in brackets were not isolated.

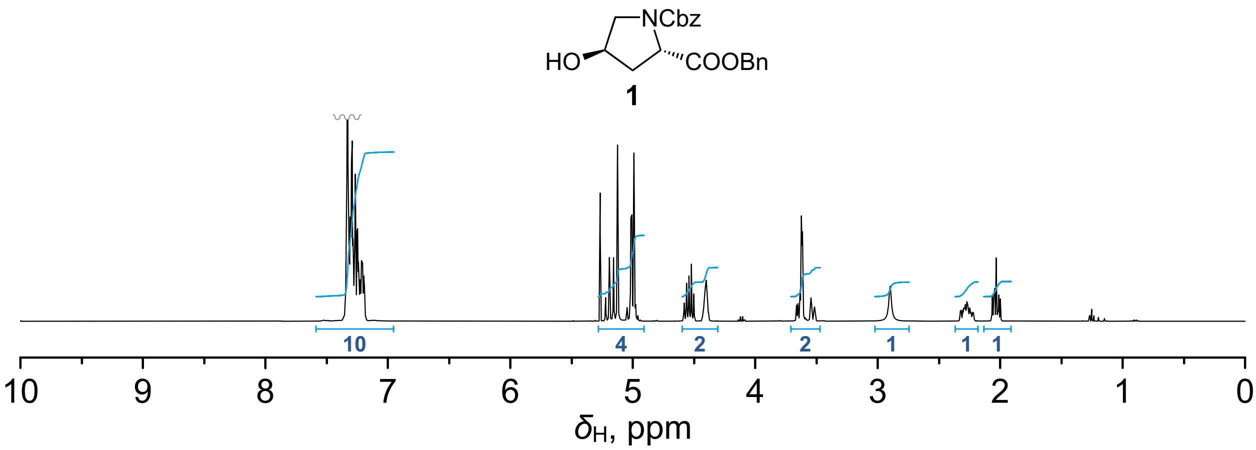

Supplementary Fig. 23. Synthesis and ^{1}H NMR spectrum ($CDCl_3$, 400.1 MHz, *T* = 300 K) of dibenzyl (*2S*,*4R*)-*4*-hydroxypyrrolidine-*1*,*2*-dicarboxylate (1). ***Synthesis:*** In a round-bottom glass flask (2 L), (*2S*,*4R*)-*1*-((benzyloxy)carbonyl)-*4*-hydroxypyrrolidine-*2*-carboxylic acid (108 g, 0.41 mol, 1.00 equiv.) and BnBr (51.9 mL, 0.43 mol, 1.05 equiv.) were dissolved in THF (760 mL) followed by dropwise addition of TEA (43.3 g, 0.43 mol, 1.05 equiv.) at 0 °C. The solution was stirred at RT for 28 h The mixture was evaporated to dryness and the residue was re-dissolved in DCM (600 mL), washed subsequently with 1M HCl (2 × 300 mL), sat. aq. $NaHCO_3$ (2 × 250 mL), H_2O (300 mL) and brine (300 mL) and dried with anh. Na_2SO_4. Filtrate was evaporated to dryness and the colourless residue was re-dissolved in DCM and adsorbed onto SiO_2. The solid was transferred to sintered funnel and was washed with 5% EtOAc in Hexane (1.5 L) to wash away impurities followed by 50% EtOAc in Hexane (2 L) which yielded fractions containing product. Removal of the solvent afforded product as a colorless oil. Yield: 93 g (64%; 1 step; based on (*2S*,*4R*)-1-((benzyloxy)carbonyl)-*4*-hydroxypyrrolidine-*2*-carboxylic acid). **NMR ($CDCl_3$, pair of rotamers):** **^{1}H** (400.1 MHz, *T* = 300 K) δ_H 2.01–2.11 (C*H*$_2$–CH–CO, m, 1H); 2.21–2.40 (C*H*$_2$–CH–CO, m, 1H); 2.93 (O*H*, s, 1H); 3.49–3.73 (C*H*$_2$–N, m, 2H); 4.37–4.48 (C*H*–O, m, 1H); 4.55;4.59 (C*H*–CO, 2 × t, 1H, $^3J_{HH}$ = 7.9); 4.96–5.31 (C*H*$_2$–arom., m, 4H); 7.00–7.60 (arom., m, 10H). **^{13}C{^{1}H}** (100.6 MHz, *T* = 300 K) δ_C 38.31;39.11 (*C*H$_2$–CH–CO, 2 × s); 54.64;55.24 (*C*H$_2$–N, 2 × s); 57.90;58.14 (*C*H–CO, 2 × s); 66.85;66.98 (*C*H$_2$–arom., 2 × s); 67.28;67.31 (*C*H$_2$–arom., 2 × s); 69.16; 69.89 (*C*H–O, 2 × s); 127.50–128.79 (*arom.*, m); 135.36;135.57 (*arom.*, 2 × s); 136.19;136.40 (*arom.*, 2 × s); 154.71;155.18 (N–*C*O, 2 × s); 172.48;172.68 (*C*O, 2 × s). **ESI-HRMS:** 378.1315 [M+Na]$^+$ (theor. [$C_{20}H_{21}O_5N_1Na_1$]$^+$ = 378.1312).

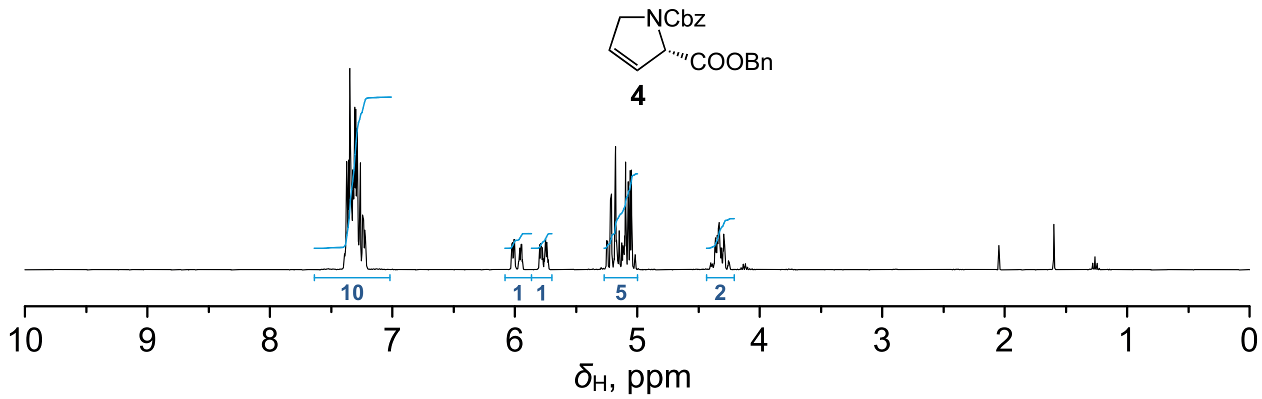

Supplementary Fig. 24. One-pot synthesis and ^{1}H NMR spectrum ($CDCl_3$, 400.1 MHz, *T* = 300 K) of dibenzyl (*S*)-2,5-dihydro-*1H*-pyrrole-1,2-dicarboxylate (4). ***Synthesis:*** In a round-bottom glass flask (500 mL), intermediate **1** (18.1 g, 51.0 mmol, 1.00 equiv.) and PPh_3 (14.1 g, 54.0 mmol, 1.05 equiv.) were dissolved in dry THF (120 mL) under Ar. Solution was cooled to 0 °C in an ice bath followed by dropwise addition of DIAD (98%, 10.6 mL, 53.8 mmol, 1.05 equiv.) and further followed by addition of MeI (3.36 mL, 54.0 mmol, 1.05 equiv.). The reaction mixture was then allowed to warm up to RT and it was further stirred at RT until disappearance of starting material that took approximately 3 h (controlled by TLC; SiO_2; Hexane–EtOAc 1:1). Reaction mixture containing intermediate **2** was then carefully evaporated to dryness (temperature of the bath was maintained at 30 °C). In a separate round-bottom glass flask (500 mL), $(PhSe)_2$ (7.96 g, 26.0 mmol, 0.5 equiv.) was suspended in dry EtOH (180 mL) and heated under condenser until dissolution under atmosphere of argon. Then, the reaction mixture was put on an ice bath and $NaBH_4$ (2.00 g, 53.0 mmol, 1.04 equiv.) was added in four portions under Ar. The mixture was then stirred until colourless solution was produced followed by addition of solution of intermediate **2** in dry EtOH (80 mL) through condenser. Reaction mixture was then heated to 70 °C. After 60 mins, TLC confirmed consumption of starting material. Reaction mixture containing intermediate **3** was cooled to RT and diluted with THF (150 mL). Mixture was further cooled to 0 °C followed by dropwise addition of H_2O_2 (30% aq., 51.2 mL, 671 mmol, 13 equiv.). The reaction mixture was then allowed to warm up to RT and further stirred for 19 h. The solution was diluted with H_2O (400 mL) and extracted with 150 mL of Et_2O. Organic phase was washed with dil. aq. $NaHCO_3$ (2 × 150 mL), aq. Na_2SO_3 (10% aq., 2 × 150 mL), brine (2 × 150 mL) and evaporated to dryness. The residue was purified by column chromatography (SiO_2, 100 % Hexane to 15% EtOAc in Hexane). Fractions containing product were pooled and evaporated to dryness to yield the product as colourless oil. **Yield:** 14.5 g (84%; 3 steps; based on **1**). **NMR ($CDCl_3$):** ^{1}**H** (400.1 MHz, *T* = 300 K) δ_H 4.22–4.45 (C*H*$_2$, m, 2H); 4.93–5.31 (C*H*–CO, C*H*$_2$–arom., m, 1H+4H); 5.69–5.81 (C*H*=CH, m, 1H); 5.89–6.07 (C*H*=CH, m, 1H); 7.04–7.60 (*arom.*, m, 10H). ^{13}**C{^{1}H}** (100.6 MHz, *T* = 300 K) δ_C 53.56;54.06 (*C*H$_2$, 2 × s); 66.55;66.86 (C*H*–CO, 2 × s); 67.07;67.20;67.28;67.34 (*C*H$_2$–arom., 4 × s); 124.75;124.79 (*C*H=CH); 127.84–128.78 (*arom.*, m); 129.37;129.49 (*C*H=CH); 135.54;135.70 (*arom.*, 2 × s); 136.54;136.69 (*arom.*, 2 × s); 154.09;154.55 (N–*C*O, 2 × s); 169.89;170.19 (*C*O, 2 × s). **ESI-HRMS:** 338.1383 $[M+H]^+$ (theor. $[C_{20}H_{20}O_4N]^+$ = 338.1387).

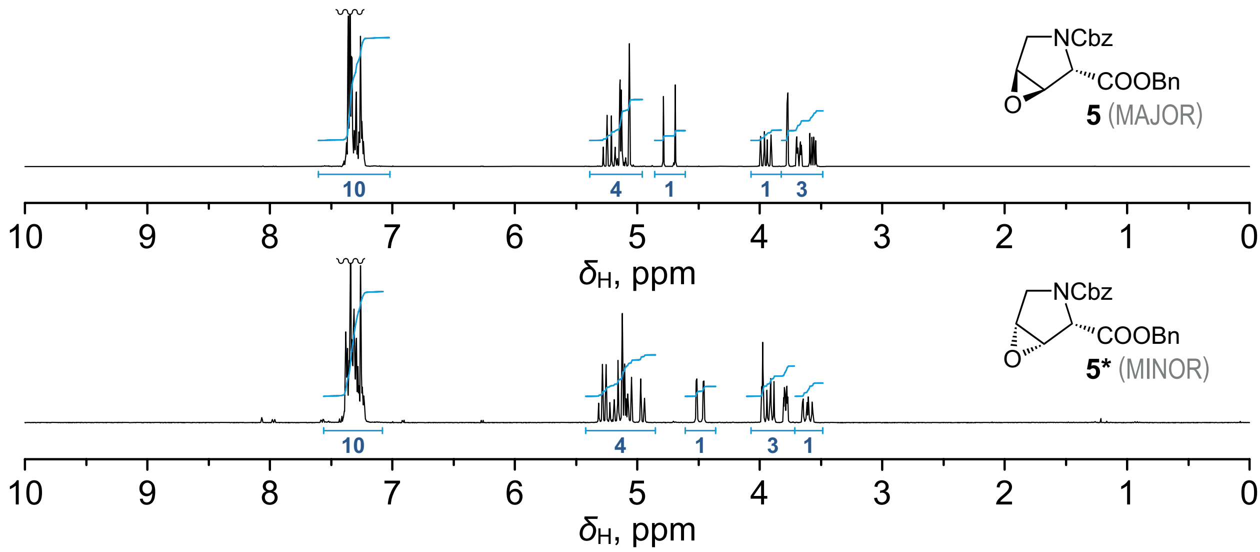

Supplementary Fig. 25. Synthesis and ^{1}H NMR spectra (CDCl$_3$, 400.1 MHz, *T* = 300 K) of dibenzyl (*1R*,*2S*,*5S*)-*6*-oxa-*3*-azabicyclo[*3.1.0*]hexane-*2*,*3*-dicarboxylate (5) and dibenzyl (*1S*,*2S*,*5R*)-*6*-oxa-*3*-azabicyclo[*3.1.0*]hexane-*2*,*3*-dicarboxylate (5*). ***Synthesis:*** In a round-bottom glass flask (250 mL), intermediate **4** (11.83 g, 35.1 mmol, 1.0 equiv.) was dissolved in $CHCl_3$ (175 mL) followed by addition of MCPBA (77%, 14.2 g, 63.4 mmol, 1.8 equiv.). The solution was stirred at 85 ºC for 20 h. Mixture was concentrated followed by addition of DCM (70 mL) to produce precipitate. Reaction mixture was put into the freezer for 24 h. White precipitate was removed by filtration. Filtrate was evaporated and the residue was purified by column chromatography (SiO_2, 100% P.E. to 40% EtOAc in P.E.). Fractions containing pure compounds were evaporated, yielding **5** as transparent oil and **5*** as white solid. **Characterization of 5** (MAJOR)**: Yield:** 6.86 g (55%; 1 step; based on **4**). **NMR (CDCl$_3$, pair of rotamers): ^{1}H** (400.1 MHz, *T* = 300 K) δ_H 3.58;3.60 (C*H*$_2$–N, 2 × dd, 1H, $^{2}J_{HH}$ = 12.5, $^{3}J_{HH}$ = 1.4); 3.69;3.72 (CH$_2$–C*H*–O, 2 × dd, 1H, $^{3}J_{HH}$ = 2.9, $^{3}J_{HH}$ = 1.4); 3.80 (O–C*H*–CH–CO, d, 1H, $^{3}J_{HH}$ = 2.9); 3.95;4.00 (C*H*$_2$–N, 2 × d, 1H, $^{2}J_{HH}$ = 12.5); 4.71;4.81 (CH–CO, 2 × s, 1H); 5.05–5.32 (C*H*$_2$–arom., m, 4H); 7.03–7.62 (*arom.*, m, 10H). **^{13}C{^{1}H}** (100.6 MHz, *T* = 300 K) δ_C 47.27;47.52 (*C*H$_2$–N, 2 × s); 54.35;54.89 (CH$_2$–*C*H–O, 2 × s); 56.59;57.31 (O–*C*H–CH–CO, 2 × s); 60.74;60.89 (*C*H–CO, 2 × s); 67.44;67.54 (*C*H$_2$–arom., 2 × s); 67.56;67.63 (*C*H$_2$–arom., 2 × s); 127.83–128.95 (*arom.*, m); 135.21;135.08 (*arom.*, 2 × s); 136.25;136.31 (*arom.*, 2 × s); 154.76;155.28 (N–*C*O, 2 × s); 168.85;168.91 (*C*O, 2 × s). **ESI-HRMS:** 376.1152 [M+Na]$^{+}$ (theor. [$C_{20}H_{19}O_5N_1Na_1$]$^{+}$ = 376.1155). **EA** ($C_{20}H_{19}NO_5\cdot0.2H_2O$, M_R = 357.0): C 67.3 (67.4); H 5.5 (5.3); N 3.9 (3.8). **Characterization of 5*** (MINOR)**: Yield:** 2.67 g (22%; 1 step; based on **4**). **NMR (CDCl$_3$, pair of rotamers): ^{1}H** (400.1 MHz, *T* = 300 K) δ_H 3.58;3.63 (C*H*$_2$–N, 2 × dd, 1H, $^{2}J_{HH}$ = 12.5, $^{3}J_{HH}$ = 2.0); 3.78;3.80 (CH$_2$–C*H*–O, 2 × dd, 1H, $^{3}J_{HH}$ = 3.0, $^{3}J_{HH}$ = 2.0); 3.90;3.93 (C*H*$_2$–N, 2 × d, 1H, $^{2}J_{HH}$ = 12.5); 3.96–4.00 (O–C*H*–CH–CO, m, 1H); 4.46;4.52 (CH–CO, 2 × d, 1H, $^{3}J_{HH}$ = 2.4); 4.92–5.33 (C*H*$_2$–arom., m, 4H); 7.03–7.58 (*arom.*, m, 10H). **^{13}C{^{1}H}** (100.6 MHz, *T* = 300 K) δ_C 47.99;48.42 (*C*H$_2$–N, 2 × s); 55.78;56.23 (CH$_2$–*C*H–O, 2 × s); 57.37;58.21 (O–*C*H–CH–CO, 2 × s); 60.18;60.43 (*C*H–CO, 2 × s); 67.33;67.46 (*C*H$_2$–arom., 2 × s); 67.51;67.62 (*C*H$_2$–arom., 2 × s); 127.96–128.88 (*arom.*, m); 135.42;135.57 (*arom.*, 2 × s); 136.15;136.33 (*arom.*, 2 × s); 154.51;155.05 (N–*C*O, 2 × s); 167.15;167.65 (*C*O, 2 × s). **ESI-HRMS:** 376.1151 [M+Na]$^{+}$ (theor. [$C_{20}H_{19}O_5N_1Na_1$]$^{+}$ = 376.1155). **EA** ($C_{20}H_{19}NO_5\cdot0.2H_2O$, M_R = 357.0): C 67.3 (67.2); H 5.5 (5.2); N 3.9 (3.7).

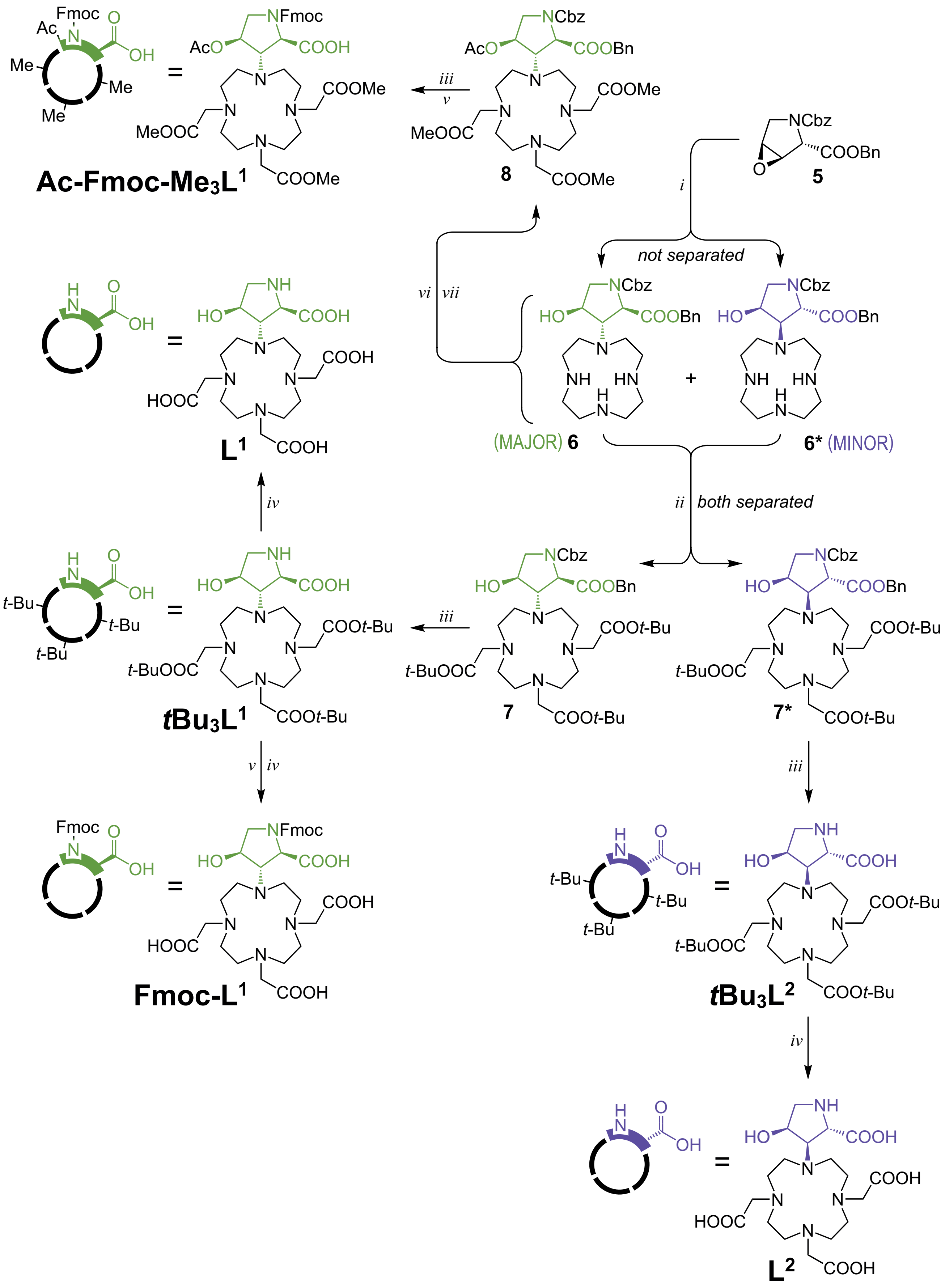

Supplementary Fig. 26. Synthesis of building blocks Ac-Fmoc-Me$_3$L^1, L^1, *t*Bu$_3$L^1, Fmoc-L^1 and *t*Bu$_3$L^2 and L^2. ***Conditions:*** (*i*) cyclen, *t*-BuOH, 105 °C; (*ii*) *t*-BuO$_2$CCH$_2$Br, K_2CO_3, MeCN; (*iii*) H_2, Pd@C, AcOH, MeOH; (*iv*) TFA; (*v*) FmocCl, borate buffer (pH 9.0), MeCN; (*vi*) MeO$_2$CCH$_2$Br, K_2CO_3, MeCN; (*vii*) Ac_2O, TEA, DMAP, MeCN.

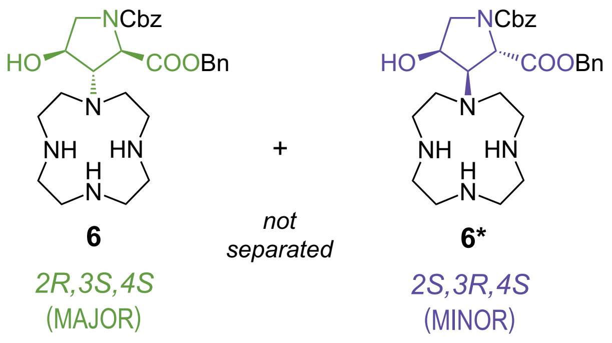

Supplementary Fig. 27. Synthesis of intermediate 6 and 6*. ***Synthesis:*** In a round-bottom glass flask (250 mL), intermediate **5** (2.0 g, 5.60 mmol, 1.0 equiv.) and cyclen (3.9 g, 22.6 mmol, 4.0 equiv.) were dissolved in dry *t*-BuOH (120 mL). The resulting solution was stirred at 105 °C for 24 h. After cooling to RT, reaction mixture was quenched with TFA (2.65 mL) and concentrated to dryness. Resulting oil was purified by flash chromatography (C18, H_2O/MeCN gradient with 0.1% TFA additive). Fractions with product were joined and lyophilized to give product (as a mixture of *2R,3S,4S–2S,3R,4S* regioisomers in ~9:1 ratio) in the form of TFA salt as faint brown solid (regioisomer separation was possible in the next steps – **7** and **8**). **Yield:** 2.30 g (54%; 1 step; based on **5**). **ESI-HRMS:** 526.3010 $[M+H]^+$ (theor. $[C_{28}H_{40}O_5N_5]^+$ = 526.3024). **EA** ($C_{28}H_{39}N_5O_5 \cdot 2.3TFA \cdot 0.3H_2O$, M_R = 793.3): C 49.4 (49.5); H 5.3 (5.1); N 8.8 (8.8); F 16.5 (16.3).

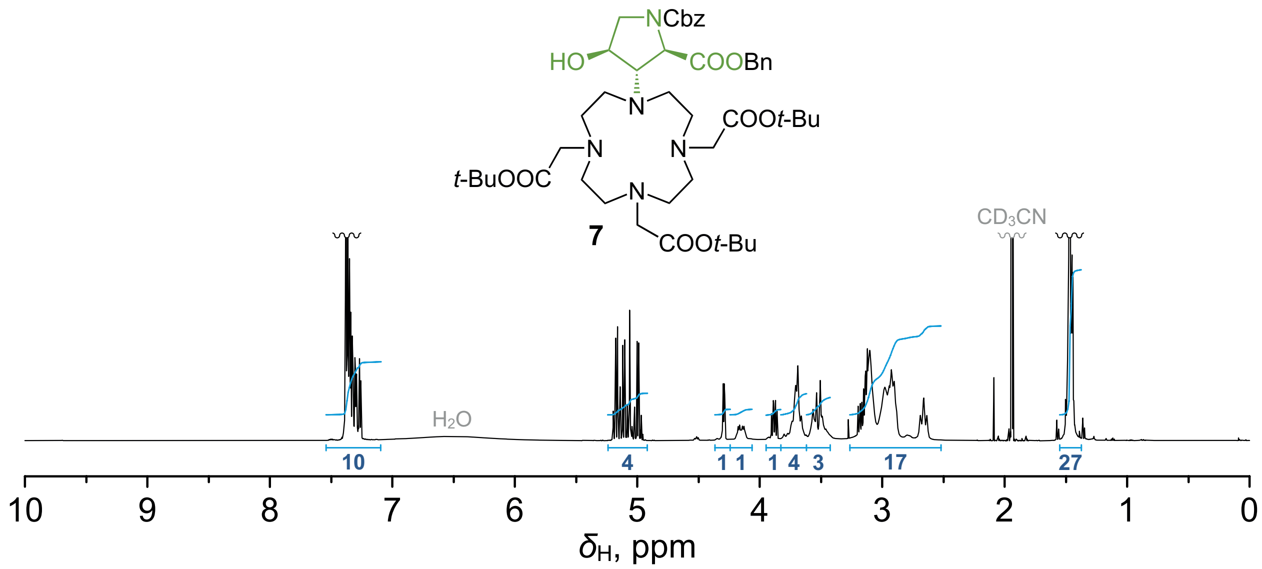

Supplementary Fig. 28. Synthesis and ^{1}H NMR spectrum (CD_3CN, 600.1 MHz, *T* = 298.1 K) of intermediate 7. ***Synthesis:*** In a pearl-shaped glass flask (50 mL), intermediate **6**·2.3TFA·0.3H_2O (mixture of *2R,3S,4S–2S,3R,4S* regioisomers in ~9:1 ratio, 788 mg, 0.99 mmol, 1.0 equiv.) and K_2CO_3 (863 mg, 6.24 mmol, 6.3 equiv.) were mixed in 20 mL of MeCN followed by addition of tert-butyl bromoacetate (630 µL, 4.23 mmol, 4.3 equiv.). The resulting suspension was stirred at RT for 16 h. Reaction mixture was filtered through syringe microfilter (PTFE), and the filtrate was evaporated to dryness. Residue was purified by preparative HPLC (C18, H_2O/MeCN gradient with 0.1% FA additive). Fractions with pure product (*2R,3S,4S*) were joined and lyophilized to give product in the form of mixed TFA/FA salt as white solid. **Yield:** 575 mg (62%; 1 step; based on **6**·2.3TFA·0.3H_2O). **NMR (CD_3CN, pair of rotamers): ^{1}H** (600.1 MHz, *T* = 298.1 K) δ_H 1.46–1.47 (C*H*$_3$, m, 27H); 2.58–3.23 (*mc*, C*H*$_2$, m, 16H+1H); 3.44–3.62 (C*H*$_2$–CO, C*H*–N, m, 2H+1H); 3.62–3.83 (C*H*$_2$–CO, m, 4H); 3.83–3.90 (C*H*$_2$, m, 1H); 4.06–4.24 (C*H*–O, m, 1H); 4.24–4.36 (C*H*–CO, m, 1H); 4.97–5.20 (C*H*$_2$–arom., m, 4H); 7.07–7.51 (*arom.*, m, 10H); **^{13}C{^{1}H}** (150.9 MHz, *T* = 298.1 K) δ_C 28.47 (CH_3, s); 28.50 (CH_3, s); 47.59 (*mc*, s); 50.58 (*mc*, s); 52.00 (CH_2, s); 53.10 (*mc*, s); 53.34 (*mc,* s); 56.05 (CH_2–CO, s); 56.44 (CH_2–CO, s); 58.21;58.67 (CH–CO, 2 × s); 68.00–68.22 (CH_2–arom., m); 70.54;71.13 (CH–O, 2 × s); 72.09;72.98 (CH–N, 2 × s); 83.5 (*C*–CH_3, s); 84.27 (*C*–CH_3, s); 128.77–129.80 (*arom.*, m); 136.64;136.84 (*arom.*, 2 × s); 137.80;138.06 (*arom.*, 2 × s); 155.01;155.65 (*C*O–N, 2 × s); 169.25 (*C*O–O, s); 170.54 (*C*O–O, s); 172.88;173.22 (CO–O, 2 × s). **ESI-HRMS:** 868.5063 $[M+H]^+$ (theor. $[C_{46}H_{70}O_{11}N_5]^+$ = 868.5066). **EA** ($C_{28}H_{39}N_5O_5$·0.3TFA·0.8FA, M_R = 939.1): C 60.6 (60.8); H 7.6 (7.5); N 7.5 (7.2); F 1.8 (2.0).

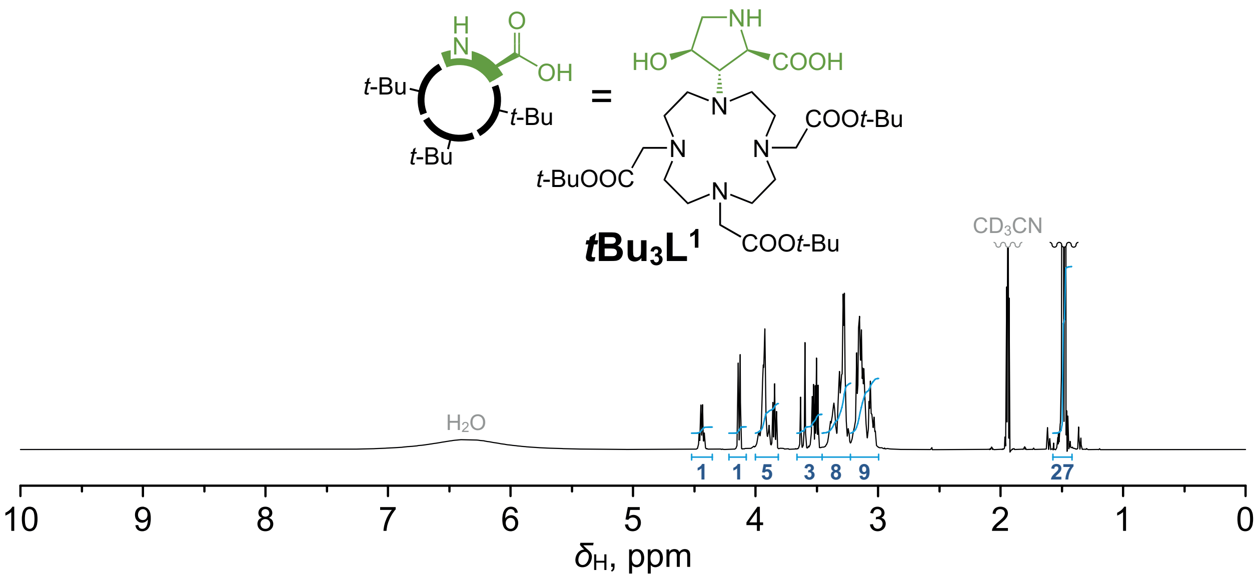

Supplementary Fig. 29. Synthesis and ^{1}H NMR spectrum (CD_3CN, 500.0 MHz, *T* = 330 K) of building block *t*Bu$_3$L^1. ***Synthesis:*** In a pear-shaped glass flask (50 mL), Pd@C (10%, 57 mg) was three-times secured with Ar followed by addition of solution of intermediate **7**·0.3TFA·0.8FA (522 mg, 556 μmol, 1.0 equiv.) in MeOH (20 mL) through septum. The mixture was then stirred at RT for 30 min with slow bubling of H_2 (from ballon) through the mixture. Reaction mixture was filtered through syringe microfilter (PTFE) and the filtrate was evaporated to dryness. Residue was purified by flash chromatography (C18, H_2O/MeCN gradient with 0.1% TFA additive). Fractions with product were joined and lyophilized to give product in the form of TFA salt as white solid. **Yield:** 313 mg (56%; 1 step; based on **7**·0.3TFA·0.8FA). **NMR (CD_3CN): ^{1}H** (500.0 MHz, *T* = 330 K) δ_H 1.47 (CH_3, s, 9H); 1.49 (CH_3, s, 18H); 2.99–3.22 (*mc*, CH_2, m, 8H+1H); 3.22–3.46 (*mc*, m, 8H); 3.46–3.56 (CH_2, CH_2–CO, m, 1H+1H); 3.61 (CH_2–CO, d, 1H, $^2J_{HH}$ = 17.8); 3.84 (*CH*–N, dd, 1H, $^3J_{HH}$ = 8.2, $^3J_{HH}$ = 6.9); 3.87–4.00 (CH_2–CO, m, 4H); 4.13 (*CH*–CO, d, 1H, $^3J_{HH}$ = 8.2); 4.44 (*CH*–O, q, 1H, $^3J_{HH}$ = 7.0). **^{13}C{^{1}H}** (125.7 MHz, *T* = 330 K) δ_C 28.43 (CH_3, s); 28.57 (CH_3, s); 47.16 (*mc*, s); 50.21 (CH_2, s); 50.36 (*mc,* s); 52.95 (*mc,* s); 52.98 (*mc*, s); 55.73 (CH_2–CO, s); 55.92 (CH_2–CO, s); 58.32 (CH–CO, s); 69.26 (CH–N, s); 71.04 (CH–O, s); 83.80 (*C*–CH_3, s); 85.28 (*C*–CH_3, s); 168.26 (*C*O, s); 170.80 (*C*O, s); 171.44 (*C*O, s). **ESI-HRMS:** 644.4227 [M+H]$^+$ (theor. $[C_{31}H_{58}O_9N_5]^+$ = 644.4229). **EA** ($C_{31}H_{57}N_5O_9$·3.0TFA·0.9H_2O, M_R = 1002.0): C 44.4 (44.4); H 6.2 (6.3); N 7.0 (7.0); F 17.1 (17.0).

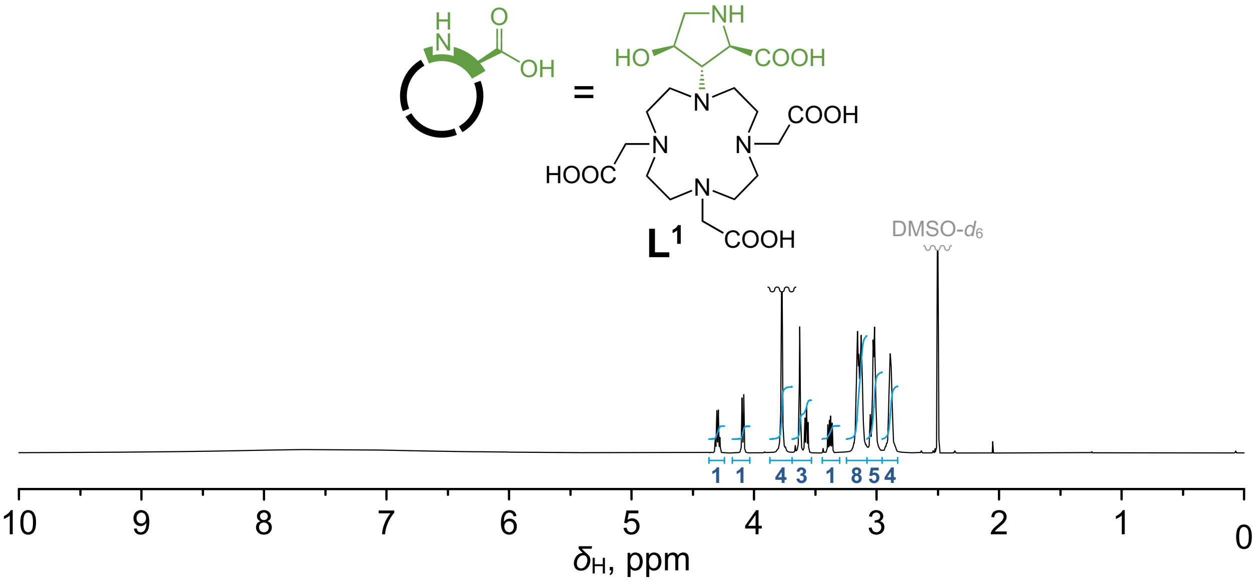

Supplementary Fig. 30. Synthesis and ^{1}H NMR spectrum (DMSO-d$_6$, 500.0 MHz, T = 330 K) of building block L^1. ***Synthesis:*** In a pear-shaped glass flask (25 mL), ***t*****-Bu$_3$L^1**·3.0TFA·0.9H_2O (278 mg, 0.28 mmol) was dissolved in TFA (4 mL). The resulting solution was stirred at RT for 5 h. The mixture was evaporated to dryness and once co-evaporated with MeOH. Residue was purified by preparative HPLC (C18, H_2O/MeCN gradient with 0.1% TFA additive). Fractions with product were joined and lyophilized to give product in the form of TFA salt as white solid. **Yield:** 156 mg (71%; 1 step; based on ***t*****-Bu$_3$L^1**·3.0TFA·0.9H_2O). **NMR (DMSO-d$_6$): ^{1}H** (500.0 MHz, T = 330 K) δ_H 2.87–2.91 (*mc*, m, 4H); 3.00–3.05 (C*H*$_2$, *mc*, m, 1H+4H); 3.10–3.18 (*mc*, m, 8H); 3.38 (C*H*$_2$, dd, 1H, $^{2}J_{HH}$ = 11.7, $^{3}J_{HH}$ = 6.4); 3.57 (C*H*–N, dd, 1H, $^{3}J_{HH}$ = 7.1, $^{3}J_{HH}$ = 5.9); 3.63 (C*H*$_2$–CO, s, 2H); 3.77 (C*H*$_2$–CO, s, 4H); 4.09 (C*H*–CO, d, 1H, $^{3}J_{HH}$ = 7.1); 4.30 (C*H*–O, q, 1H, $^{3}J_{HH}$ = 6.3). **^{13}C{^{1}H}** (125.7 MHz, T = 330 K) δ_C 46.22 (*mc*, s); 49.08 (*mc*, s); 49.60 (*C*H–CO, s); 51.41 (*mc,* s); 51.88 (*mc*, s); 53.81 (*C*H$_2$–CO, s); 54.52 (*C*H$_2$–CO, s); 57.42 (*C*H$_2$, s); 69.08 (*C*H–O, s); 70.01 (*C*H–N, s); 169.92 (*C*O, s); 170.11 (*C*O, s); 171.08 (*C*O, s). **ESI-HRMS:** 474.2209 [M−H]$^-$ (theor. $[C_{19}H_{32}O_9N_5]^-$ = 474.2206). **EA** ($C_{19}H_{33}N_5O_9$·2.4TFA·2.5H_2O, M_R = 794.1): C 36.0 (36.0); H 5.1 (4.8); N 8.8 (8.7); F 17.2 (17.0).

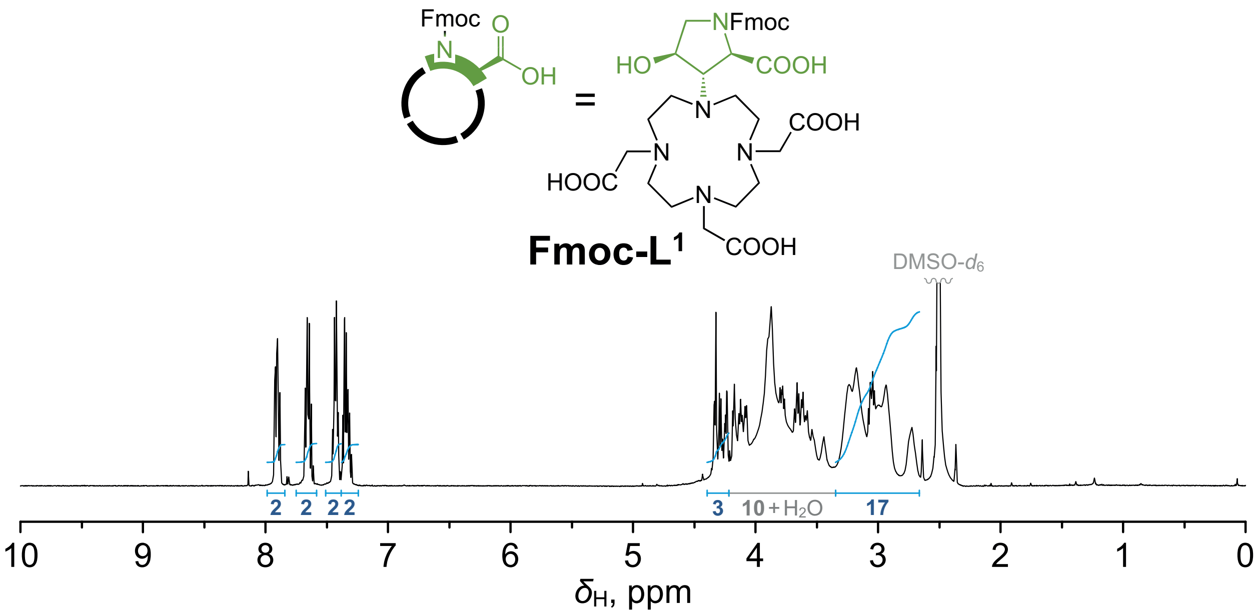

Supplementary Fig. 31. Synthesis and ^{1}H NMR spectrum (DMSO-d$_6$, 500.0 MHz, T = 298.1 K) of building block Fmoc-L^1. ***Synthesis:*** In a pear-shaped glass flask (25 mL), ***t*Bu$_3$L^1**·3.0TFA·0.9H_2O (69.0 mg, 68.9 µmol, 1.0 equiv.) was dissolved in a mixture of MeCN (4 mL) and aq. borate buffer (200 mM, pH 9.0, 3.45 mL, 690 µmol, 10 equiv.) followed by addition of FmocCl (17.8 mg, 68.8 µmol, 1.0 equiv.). The mixture was stirred at RT for 30 mins. Solution was purified by preparative HPLC (C18, H_2O/MeCN gradient with 0.1% TFA additive). Fractions with *tert*-butyl protected product were joined and evaporated to dryness. Residue was dissolved in TFA (2 mL) and stirred at RT for 5 h. The mixture was evaporated to dryness and once co-evaporated with MeOH. Residue was purified by preparative HPLC (C18, H_2O/MeCN gradient with 0.1% FA additive). Fractions with product were joined and lyophilized to give product in the form of mixed FA/TFA salt as white solid. **Yield:** 41 mg (60%; 2 steps; based on ***t*Bu$_3$L^1**·3.0TFA·0.9H_2O). **NMR (DMSO-d$_6$, pair of rotamers): ^{1}H** (500.0 MHz, T = 298.1 K) δ_H 2.67–3.29 (*mc*, CH_2, m, 16H+1H); 3.42–4.21 (CH_2, CH–N, CH–CO, CH–O, CH_2–CO, m, 1H+1H+1H+1H+6H, obscured by signal from H_2O); 4.21–4.33 (*Fmoc*, m, 3H); 7.29–7.37 (*Fmoc*, m, 2H); 7.40–7.45 (*Fmoc*, m, 2H); 7.62–7.67 (*Fmoc*, m, 2H); 7.87–7.92 (*Fmoc*, m, 2H). **^{13}C{^{1}H}** (125.7 MHz, T = 298.1 K) δ_C 45.68–54.03 (*mc*, CH_2–CO, m); 46.82;46.89 (*Fmoc*, 2 × s); 50.91;51.45 (CH_2, 2 × s); 55.84;57.01 (CH–CO, 2 × s); 66.89;67.33 (*Fmoc*, 2 × s); 69.13;69.24 (CH–O, 2 × s); 70.20;71.43 (CH–N, 2 × s); 120.33–120.44 (*Fmoc*, m); 125.30;125.38;125.45;125.54 (*Fmoc*, 4 × s); 127.35–127.43 (*Fmoc*, m); 127.86–128.08 (*Fmoc*, m); 140.86;140.92;140.99;141.02 (*Fmoc*, 4 × s); 143.85;143.88;143.95;143.99 (*Fmoc*, 4 × s); 153.84+154.05 (N–*C*O, 2 × s); 170.25 (*C*O, s); 172.11 (*C*O, s); 173.48;173.73 (*C*O, 2 × s). **ESI-HRMS:** 698.3033 $[M+H]^+$ (theor. $[C_{34}H_{44}O_{11}N_5]^+$ = 698.3032). **EA** ($C_{34}H_{43}N_5O_{11}$·2.0TFA·1.3FA, M_R = 985.5): C 47.9 (47.8); H 4.9 (4.8); N 7.1 (7.0); F 11.6 (11.7).

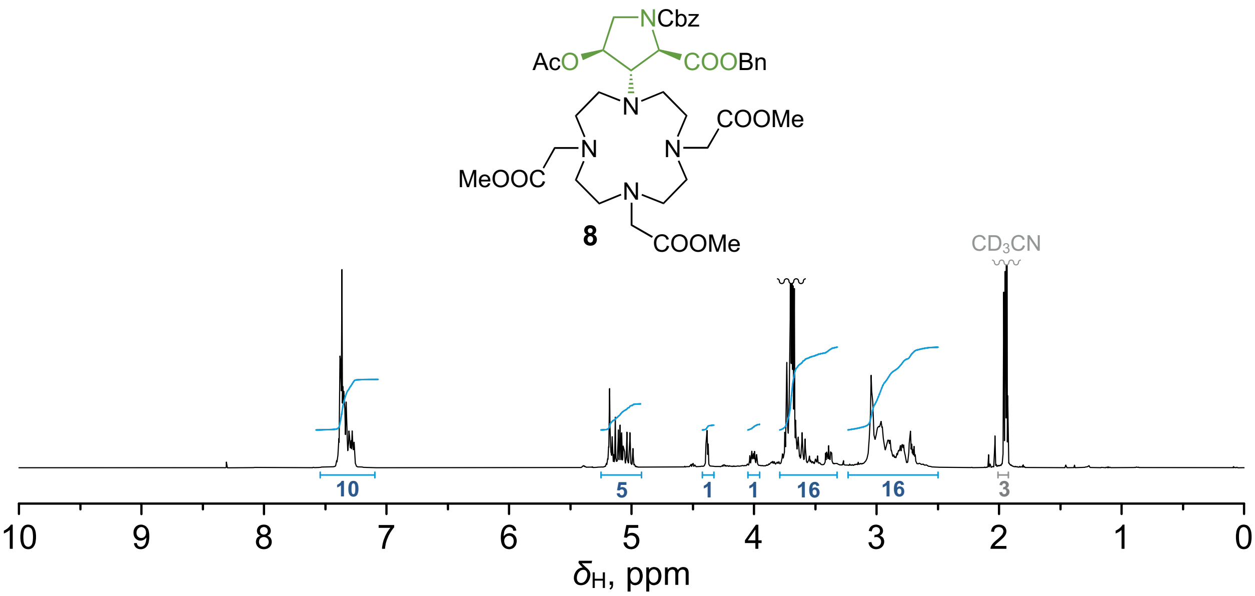

Supplementary Fig. 32. Synthesis and ^{1}H NMR spectrum (CD_3CN, 500.0 MHz, *T* = 298.1 K) of intermediate 8. ***Synthesis:*** In a pear-shaped glass flask (50 mL), intermediate **6**·2.3TFA·0.3H_2O (mixture of *2R,3S,4S–2S,3R,4S* regioisomers in ~9:1 ratio, 464 mg, 585 µmol, 1.0 equiv.) and K_2CO_3 (508 mg, 3.68 mmol, 6.3 equiv.) were mixed in 25 mL of MeCN followed by addition of methyl bromoacetate (209 µL, 2.21 mmol, 3.8 equiv.). The resulting suspension was stirred for at RT 16 h. Reaction mixture was filtered through syringe microfilter (PTFE), and the filtrate was evaporated to dryness. Residue was purified by preparative HPLC (C18, H_2O/MeCN gradient with 0.1% TFA additive). Fractions with fully alkylated intermediate were joined and lyophilized. The resulting white solid was dissolved in MeCN (8 mL) followed by addition of Ac_2O (102 µL, 1.08 mmol; 1.8 equiv.), TEA (362 µL, 2.60 mmol, 4.5 equiv.) and DMAP (3 mg, 22 µmol, ~4 mol %). The resulting solution was stirred at RT for 24 h. Reaction mixture was evaporated to dryness and the residue was purified by preparative HPLC (C18, H_2O/MeCN gradient with 0.1% TFA additive). Fractions with pure product (*2R,3S,4S*) were joined and lyophilized to give product in the form of TFA salt as white solid. **Yield:** 323 mg (54%; 2 steps; based on **6**·2.3TFA·0.3H_2O). **NMR (CD_3CN, pair of rotamers): ^{1}H** (500.0 MHz, *T* = 298.1 K) δ_H 1.95;1.96 (CH_3–C, 2 × s, 3H); 2.50–3.24 (*mc*, CH_2, m, 16H+1H); 3.32–3.79 (C*H*–N, CH_2–CO, CH_3–O, m, 1H+6H+9H); 3.93–4.07 (CH_2, m, 1H); 4.35–4.42 (C*H*–CO, m, 1H); 7.02–7.55 (*arom.*, m, 10H). **^{13}C{^{1}H}** (125.7 MHz, *T* = 298.1 K) δ_C 21.16 (CH_3–C, s); 46.95;47.15 (*mc*, s); 50.13;50.37 (CH_2, 2 × s); 52.50–52.96 (CH_3–O, m); 51.69–55.35 (mc, CH_2–CO, m); 60.18;60.60 (CH–CO, 2 × s); 67.95–68.13 (CH_2–arom., m); 70.17;71.41 (CH–N, 2 × s); 73.40;74.11 (CH–O, 2 × s); 128.66–129.62 (*arom.*, m); 136.55;136.71 (*arom.*, 2 × s); 137.56;137.72 (*arom.*, 2 × s); 155.41;155.61 (CO–N, 2 × s); 170.32–171.43 (CO–O, m); 171.73;172.02 (CO–O, 2 × s). **ESI-HRMS:** 784.3757 $[M+H]^+$ (theor. $[C_{39}H_{54}O_{12}N_5]^+$ = 784.3764). **EA** ($C_{39}H_{53}N_5O_{12}$·1.9TFA·0.8H_2O, M_R = 1014.9): C 50.7 (50.9); H 5.6 (5.6); N 6.9 (6.6); F 10.7 (10.6).

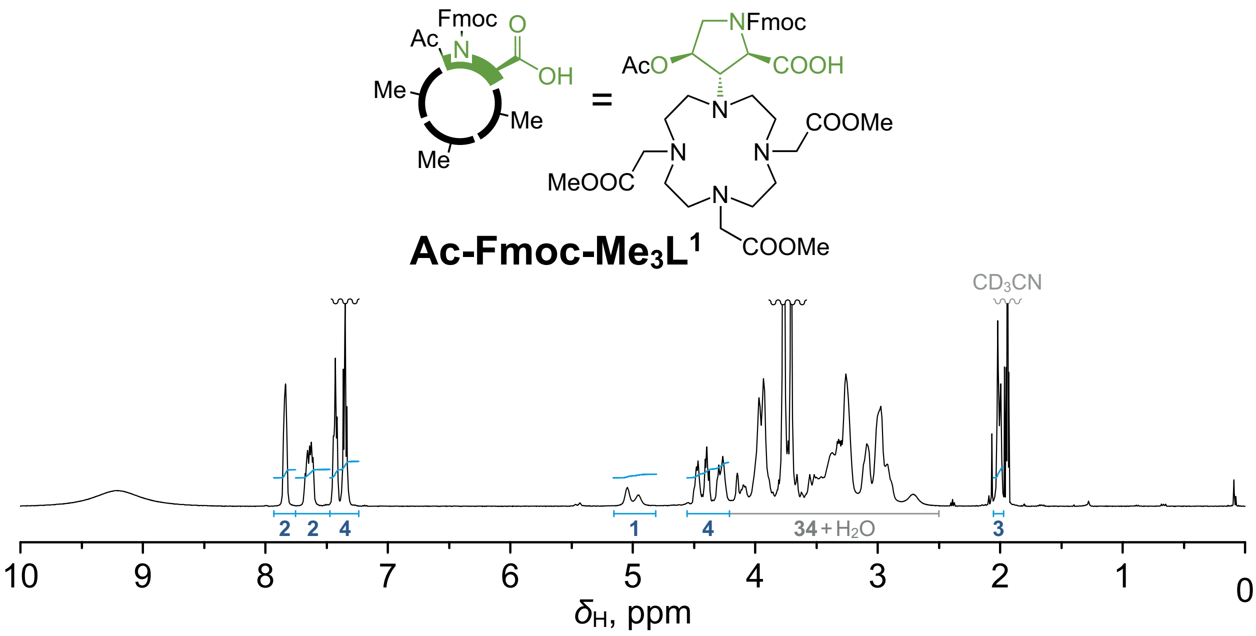

Supplementary Fig. 33. Synthesis and ^{1}H NMR spectrum (CD_3CN, 500.0 MHz, T = 323 K) of building block Ac-Fmoc-Me_3L^1. ***Synthesis:*** In a pear-shaped glass flask (50 mL), Pd@C (10%, 35 mg) was three-times secured with Ar followed by addition of solution of intermediate **8**·1.9TFA·0.8H_2O (346 mg, 341 µmol, 1.0 equiv.) in MeOH (30 mL) through septum. The mixture was then stirred at RT for 30 min with slow bubling of H_2 (from ballon) through the mixture. Reaction mixture was filtered through syringe microfilter (PTFE) and the filtrate was evaporated to dryness. Residue was purified by preparative HPLC (C18, H_2O/MeCN gradient with 0.1% TFA additive). Fractions with partially deprotected intermediate were joined and lyophilized. The resulting white solid (153 mg, assuming M·TFA, M_R = 674, 0.23 mmol) was dissolved in a mixture of MeCN (12 mL) and aq. borate buffer (200 mM, pH 9.0, 11.3 mL, 2.26 mmol, 10 equiv.) followed by addition of FmocCl (58 mg, 0.23 mmol, 1.0 equiv.). The mixture was stirred for at RT for 30 mins. Solution was then concentrated and purified by preparative HPLC (C18, H_2O/MeCN gradient with 0.1% TFA additive). Fractions with product were joined and lyophilized to give product in the form of TFA salt as white solid. **Yield:** 184 mg (52%; 2 steps; based on **8**·1.9TFA·0.8H_2O). **NMR (CD_3CN, pair of rotamers):** ^{1}**H** (500.0 MHz, T = 323 K) δ_H 1.99;2.02 (CH_3–C, 2 × s, 3H); 2.66–4.22 (*mc*, CH_2, CH–N, CH_3–O, CH_2–CO, m, 16H+2H+1H+9H+6H); 4.21–4.55 (*Fmoc*, CH–CO, m, 3H+1H); 4.80–5.16 (CH–O, m, 1H); 7.33–7.44 (*Fmoc*, m, 4H); 7.61–7.67 (*Fmoc*, m, 2H); 7.82–7.86 (*Fmoc*, m, 2H). ^{13}**C**{^{1}**H**} (125.7 MHz, T = 323 K) δ_C 21.31 (CH_3–C, s); 48.24 (*Fmoc*, s); 52.99 (CH_3–O, s); 53.62 (CH_3–O, s); 46.89–55.14 (*mc*, CH_2–CO, CH_2, m); 58.77;59.93 (CH–CO, 2 × s); 68.43 (*Fmoc*, s); 69.05;70.43 (CH–N, 2 × s); 74.03 (CH–O, s); 121.12 (*Fmoc*, s); 126.22 (*Fmoc*, s); 128.30 (*Fmoc*, s); 128.90 (*Fmoc*, s); 142.25–142.41 (*Fmoc*, m); 145.06–145.28 (*Fmoc*, m); 154.69;155.47 (N–CO, 2 × s); 169.24–172.59 (CO, CO, m). **ESI-HRMS:** 782.3600 $[M+H]^+$ (theor. $[C_{39}H_{52}O_{12}N_5]^+$ = 782.3607). **EA** ($C_{39}H_{51}N_5O_{12}$·1.9TFA·1.3H_2O, M_R = 1035.7): C 50.3 (50.6); H 5.5 (5.4); N 6.9 (6.5); F 10.6 (10.7).

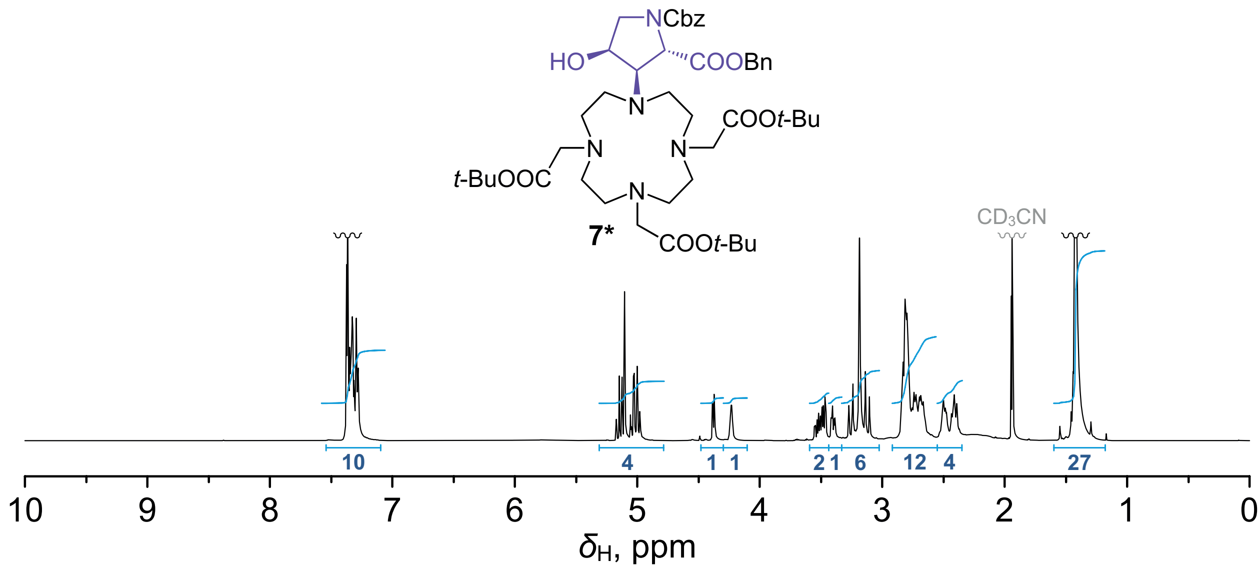

Supplementary Fig. 34. Synthesis and ^{1}H NMR spectrum (CD_3CN, 500.0 MHz, T = 298.1 K) of intermediate 7*. ***Synthesis:*** Obtained as a side product during synthesis of **7.** Fractions with pure product (*2S,3R,4S*) were joined and lyophilized to give product as white solid. **Yield:** 47 mg (5%; 1 step; based on **6**·2.3TFA·0.3H_2O assuming **7***·0.3TFA·0.8FA, M_R = 939). **NMR (CD_3CN, pair of rotamers):** **^{1}H** (500.0 MHz, T = 298.1 K) δ_H 1.42 (CH_3, s, 27H); 2.34–2.56 (*mc*, m, 4H); 2.56–2.90 (*mc*, m, 12H); 3.02–3.33 (CH_2–CO, m, 6H); 3.33–3.43 (CH_2, m, 1H); 3.43–3.59 (CH_2, C*H*–N m, 1H+1H); 4.10–4.30 (C*H*–O, m, 1H); 4.30–4.47 (C*H*–CO, m, 1H); 4.79–5.31 (CH_2–arom., m, 4H); 7.07–7.51 (*arom.*, m, 10H). **^{13}C{^{1}H}** (125.7 MHz, T = 298.1 K) δ_C 28.39 (CH_3, s); 52.49;52.88 (*mc*, 2 × s); 53.39;53.50 (CH_2, 2 × s); 53.84;53.88 (*mc*, 2 × s); 54.41;54.44 (*mc*, 2 × s); 56.64 (CH_2–CO, s); 57.51;57.54 (CH_2–CO, 2 × s); 57.77;58.39 (CH–CO, 2 × s); 67.50;67.55 (CH_2–arom., 2 × s); 67.64;67.71 (CH_2–arom., 2 × s); 68.29;69.34 (CH–N, 2 × s); 72.45;72.96 (CH–O, 2 × s); 81.16 (*C*–CH_3, s); 81.47 (*C*–CH_3, s); 128.44–129.79 (*arom.*, m); 136.70;136.90 (*arom.*, 2 × s); 137.83;138.08 (*arom.*, 2 × s); 155.36;155.73 (*C*O–N, 2 × s); 171.31 (*C*O–O, s); 171.81 (*C*O–O, s); 172.88;173.22 (*C*O–O, 2 × s). **ESI-HRMS:** 868.5068 $[M+H]^+$ (theor. $[C_{46}H_{70}O_{11}N_5]^+$ = 868.5066).

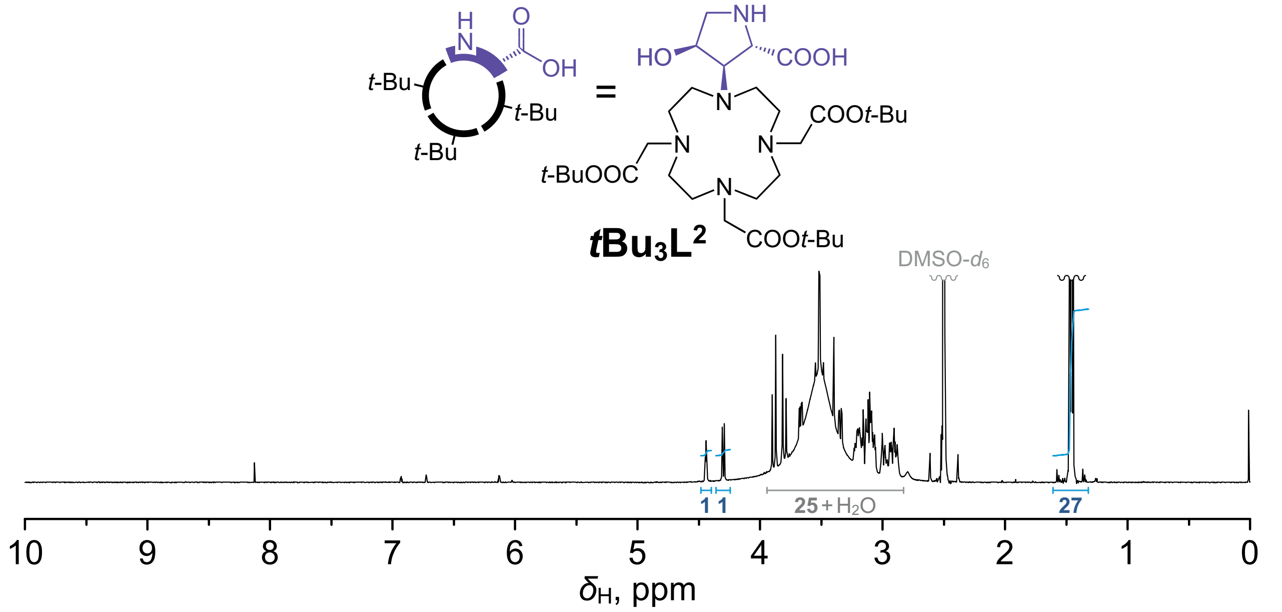

Supplementary Fig. 35. Synthesis and ^{1}H NMR spectrum (DMSO-d$_6$, 600.1 MHz, *T* = 345 K) of building block *t*Bu$_3$L^2. ***Synthesis:*** In a glass flask (4 mL), Pd@C (10%, 4 mg) was three-times secured with Ar followed by addition of solution of intermediate **7***·0.3TFA·0.8FA (47 mg, 50 μmol, 1.0 equiv.) in MeOH (2 mL) through septum. The mixture was then stirred at RT for 30 min with slow bubling of H_2 (from ballon) through the mixture. Reaction mixture was filtered through syringe microfilter (PTFE) and the filtrate was evaporated to dryness. Residue was purified by preparative HPLC (C18, H_2O/MeCN gradient with 0.1% TFA additive). Fractions with product were joined and lyophilized to give product in the form of TFA salt as white solid. **Yield:** 27 mg (56%; 1 step; based on **7***·0.3TFA·0.8FA). **NMR (DMSO-d$_6$): ^{1}H** (600.1 MHz, *T* = 345 K) δ_H 1.46 (C*H*$_3$, s, 9H); 1.47 (C*H*$_3$, s, 18H); 2.87–3.01 (*mc*, m, 6H); 3.05–3.23 (*mc*, C*H*$_2$, m, 10H+1H); 3.34 (C*H*$_2$, dd, 1H, $^2J_{HH}$ = 12.2, $^3J_{HH}$ = 3.6); 3.50 (C*H*$_2$–CO, d, 1H, $^2J_{HH}$ = 17.3); 3.53 (C*H*$_2$–CO, d, 1H, $^2J_{HH}$ = 17.3); 3.67 (C*H*–N, dd, 1H, $^3J_{HH}$ = 9.7, $^3J_{HH}$ = 4.4); 3.80 (C*H*$_2$–CO, d, 2H, $^2J_{HH}$ = 17.1); 3.89 (C*H*$_2$–CO, d, 2H, $^2J_{HH}$ = 17.1); 4.30 (C*H*–CO, d, 1H, $^3J_{HH}$ = 9.7); 4.41–4.47 (C*H*–O, m, 1H). **^{13}C{^{1}H}** (150.9 MHz, *T* = 345 K) δ_C 27.67 (CH$_3$, s); 27.70 (CH$_3$, s); 47.24 (*mc*, s); 48.59 (*mc*, s); 51.37 (CH$_2$, s); 52.19 (*mc*, s); 52.88 (*mc*, s); 54.07 (CH$_2$–CO, s); 58.32 (CH–CO, s); 55.68 (CH$_2$–CO, s); 65.68 (CH–N, s); 70.43 (CH–O, s); 81.12 (*C*–CH$_3$, s); 82.10 (*C*–CH$_3$, s); 167.60 (*C*O, s); 169.53 (*C*O, s); 169.86 (*C*O, s). **ESI-HRMS:** 644.4227 [M+H]$^+$ (theor. $[C_{31}H_{58}O_9N_5]^+$ = 644.4229). **EA** ($C_{31}H_{57}N_5O_9$·3.0TFA·3.1H_2O, M_R = 1041.6): C 42.7 (42.2); H 6.4 (5.9); N 6.7 (6.3); F 16.4 (16.0).

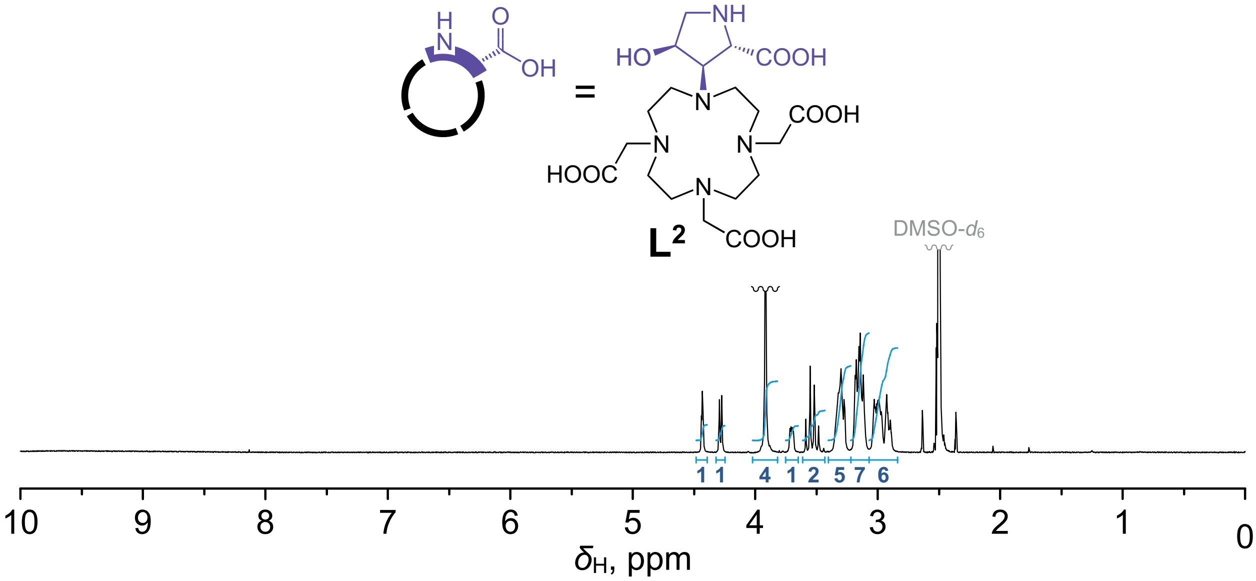

Supplementary Fig. 36. Synthesis and ^{1}H NMR spectrum (DMSO-d$_6$, 500.0 MHz, T = 330 K) of building block L^2. ***Synthesis:*** In a pear-shaped glass flask (25 mL), ***t*-Bu$_3$L^2**·3.0TFA·3.1H$_2$O (88 mg, 84 µmol) was dissolved in TFA (2 mL). The resulting solution was stirred at RT for 4 h. The mixture was evaporated to dryness and once co-evaporated with MeOH. Residue was purified by preparative HPLC (C18, H$_2$O/MeCN gradient with 0.1% TFA additive). Fractions with product were joined and lyophilized to give product in the form of TFA salt as white solid. **Yield:** 48 mg (72%; 1 step; based on ***t*-Bu$_3$L^2**·3.0TFA·3.1H$_2$O). **NMR (DMSO-d$_6$): ^{1}H** (500.0 MHz, T = 330 K) δ_H 2.84–3.07 (*mc*, m, 6H); 3.07–3.22 (C*H*$_2$, *mc*, m, 1H+6H); 3.22–3.40 (C*H*$_2$, *mc*, m, 1H+4H); 3.43–3.61 (C*H*$_2$–CO, m, 2H); 3.77 (C*H*–N, dd, 1H, $^3J_{HH}$ = 9.6, $^3J_{HH}$ = 4.4); 3.92 (C*H*$_2$–CO, s, 4H); 4.28 (C*H*–CO, d, 1H, $^3J_{HH}$ = 9.6); 4.39–4.48 (C*H*–O, m, 1H). **^{13}C{^{1}H}** (125.7 MHz, T = 330 K) δ_C 46.73 (*mc*, s); 48.17 (*mc*, s); 51.15 (*C*H$_2$, s); 51.95 (*mc*, s); 52.27 (*mc*, s); 53.51 (*C*H$_2$–CO, s); 54.29 (*C*H$_2$–CO, s); 54.90 (*C*H–CO, s); 64.42 (*C*H–N, s); 70.15 (*C*H–O, s); 169.15 (*C*O, s); 169.94 (*C*O, s); 171.79 (*C*O, s). **ESI-HRMS:** 476.2350 [M+H]$^+$ (theor. [C$_{19}$H$_{34}$O$_9$N$_5$]$^+$ = 476.2351). **EA** (C$_{19}$H$_{33}$N$_5$O$_9$·2.4TFA·2.5H$_2$O, M_R = 794.1): C 36.0 (36.7); H 5.1 (4.6); N 8.8 (8.1); F 17.2 (16.8).

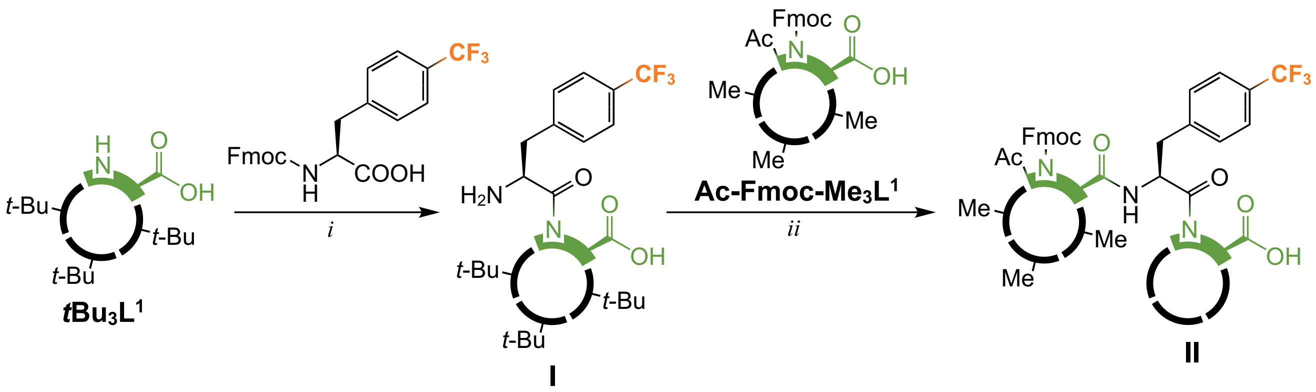

Supplementary Fig. 37. Synthesis of partially protected tripeptide intermediate II. ***Conditions:*** (*i*) Fmoc-Phe{*p*-CF_3}-OH, PyAOP, DIPEA, DMSO *followed by* DBU, DMF; (*ii*) **Ac-Fmoc-Me$_3$L^1**, PyAOP, DIPEA, DMSO *followed by* TFA.

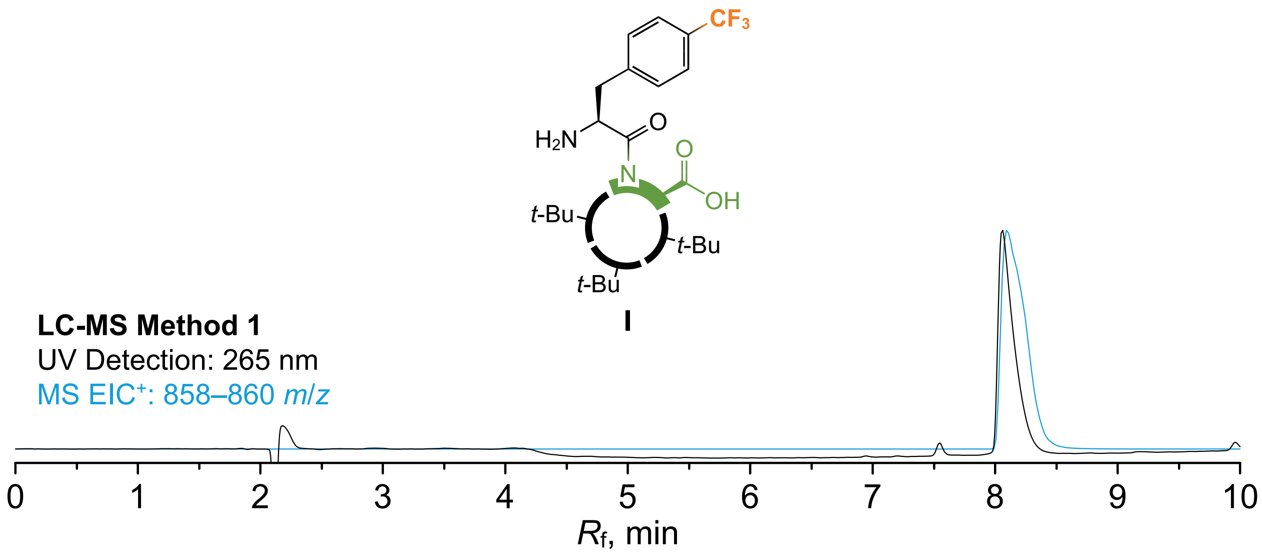

Supplementary Fig. 38. Synthesis and LC-MS chromatogram of intermediate I. ***Synthesis:*** In a glass vial (4 mL), Fmoc-Phe{p-CF_3}-OH (29 mg, 63.4 µmol, 1.1 equiv.), PyAOP (33 mg, 63.4 µmol, 1.1 equiv.) and DIPEA (55 µL, 314 µmol, 5.5 equiv.) were dissolved in dry DMSO (1.2 mL). After 2 mins, ***t*Bu₃L¹**·3.0TFA·0.9H_2O (57.4 mg, 57.3 µmol, 1.0 equiv.) was added and solution was stirred at RT for 30 mins. Solution was purified by preparative HPLC (C18, H_2O/MeCN gradient with 0.1% FA additive). Fractions with Fmoc protected product were joined and lyophilized. The resulting white solid was dissolved in dry DMF (2.4 mL) followed by addition of DBU (48 µL, 322 µmol, 5.6 equiv.). After 5 mins, the reaction was quenched with TFA (25 µL, 327 µmol, 5.7 equiv.) and diluted with H_2O (1 mL). Solution was then purified by preparative HPLC (C18, H_2O/MeCN gradient with 0.1% FA additive). Fractions with product were joined and lyophilized to give product as white solid. **Yield:** 26 mg (53%; 2 steps; based on ***t*Bu$_3$L^1**·3.0TFA·0.9H_2O assuming zwitterionic form of **I**, M_R = 859.0). **ESI-HRMS:** 859.4789 $[M+H]^+$ (theor. $[C_{41}H_{66}F_3O_{10}N_6]^+$ = 859.4787).

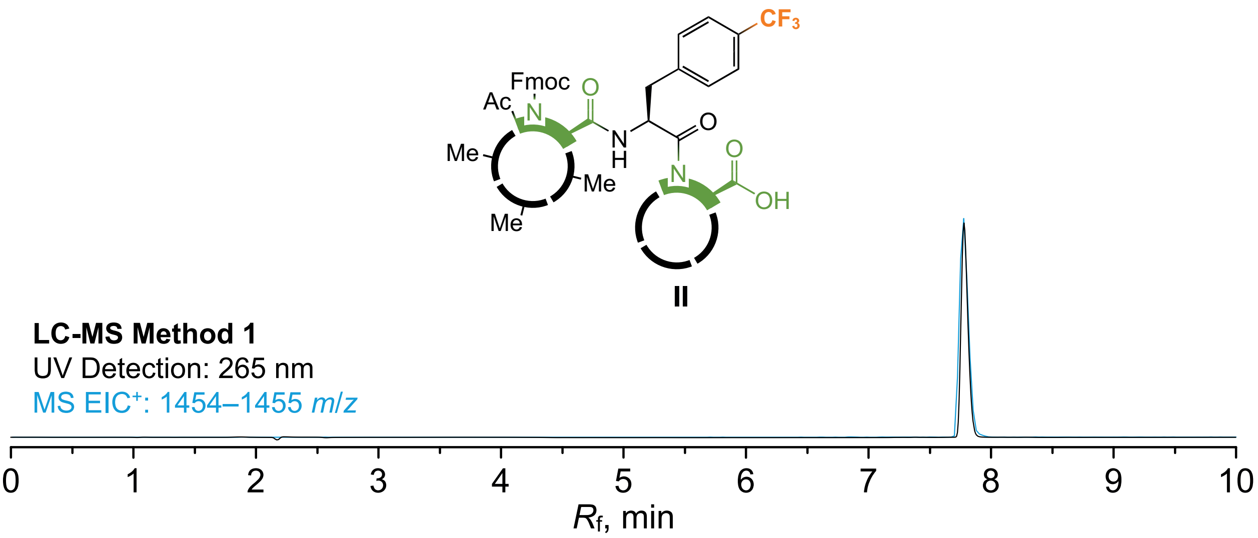

Supplementary Fig. 39. Synthesis and LC-MS chromatogram of intermediate II. ***Synthesis:*** In a glass vial (4 mL), **Ac-Fmoc-Me$_3$L^1**·1.9TFA·1.3H_2O (17.8 mg, 17.4 µmol, 1.0 equiv.) was dissolved in dry DMSO (850 µL) followed by addition of freshly prepared solution of PyOAP (100 mM in dry DMSO, 174 µL, 17.4 µmol, 1.0 equiv.) and of DIPEA (25 µL, 143 µmol, 8.2 equiv.). The resulting mixture was stirred at RT for 2 mins followed by addition of solution of **I** (22.9 mg, 26.7 µmol assuming zwitterionic form, 1.5 equiv.) in dry DMSO (1 mL) and the mixture was further stirred at RT for 30 mins. Solution was then purified by preparative HPLC (C18, H_2O/MeCN gradient with 0.1% FA additive). Fractions with *tert*-butyl product were joined and lyophilized. The resulting solid was dissolved in TFA (3 mL) and the resulting solution was stirred at RT for 16 h. Reaction mixture was evaporated to dryness and twice co-evaporated with MeOH. Residue was purified by preparative HPLC (C18, H_2O/MeCN gradient with 0.1% TFA additive). Fractions with product were joined and lyophilized. **Yield:** 19.0 mg (66%; 2 steps; based on **Ac-Fmoc-Me$_3$L^1**·1.9TFA·1.3H_2O assuming **II**·2TFA, M_R = 1682). **ESI-HRMS:** 1454.6357 [M+H]$^+$ (theor. [$C_{68}H_{91}F_3O_{21}N_{11}$]$^+$ = 1454.6338).

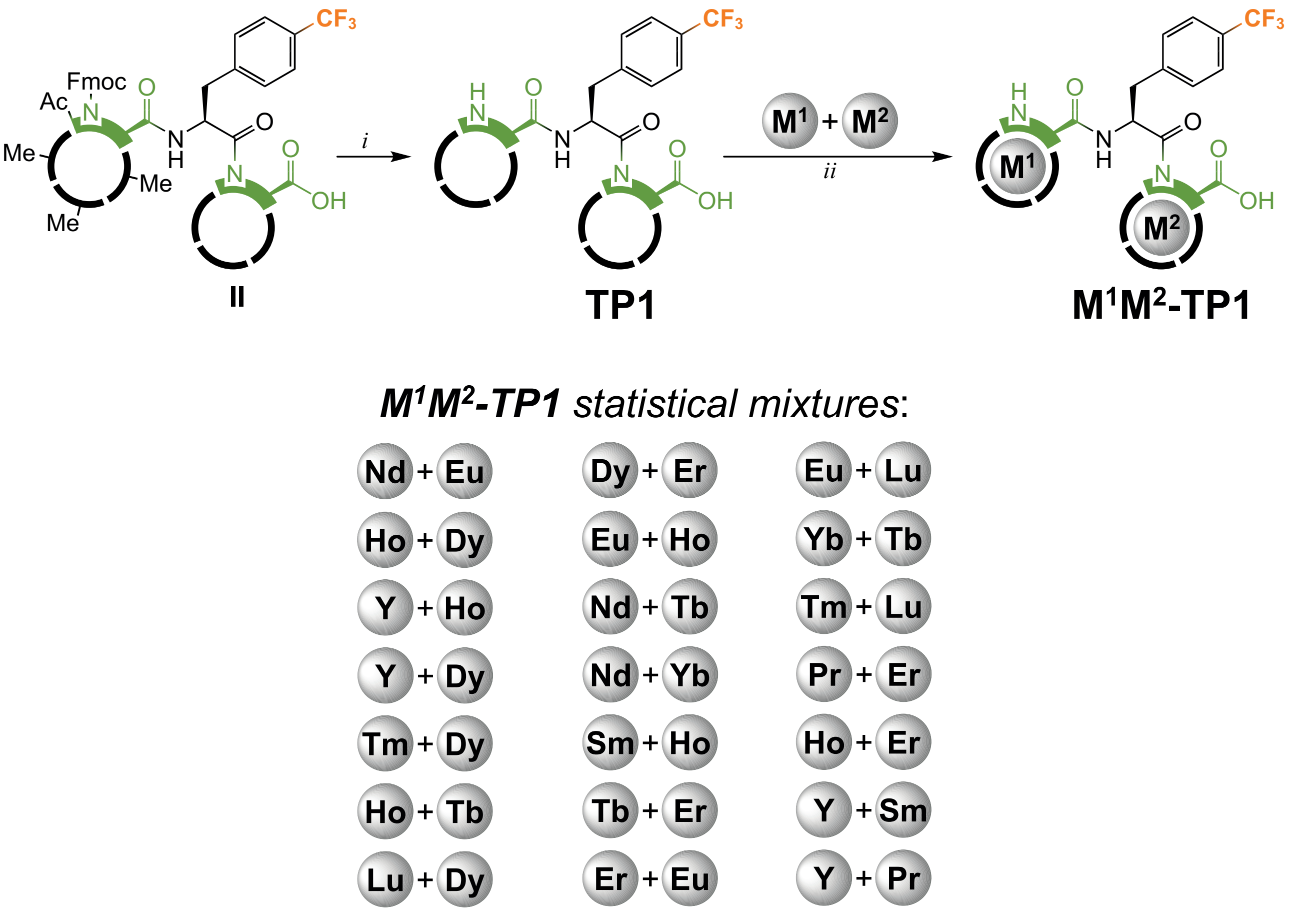

Supplementary Fig. 40. Synthesis of M¹M²-TP1 statistical mixture tripeptides using post-synthetic complexation. List of binary M^{3+} ion combinations (21) is included (charges were omitted for clarity reason). ***Conditions:*** (*i*) LiOH, H_2O, MeOH; (*ii*) M^1Cl_3, M^2Cl_3, aq. MOPS/NaOH buffer (pH 7.0).

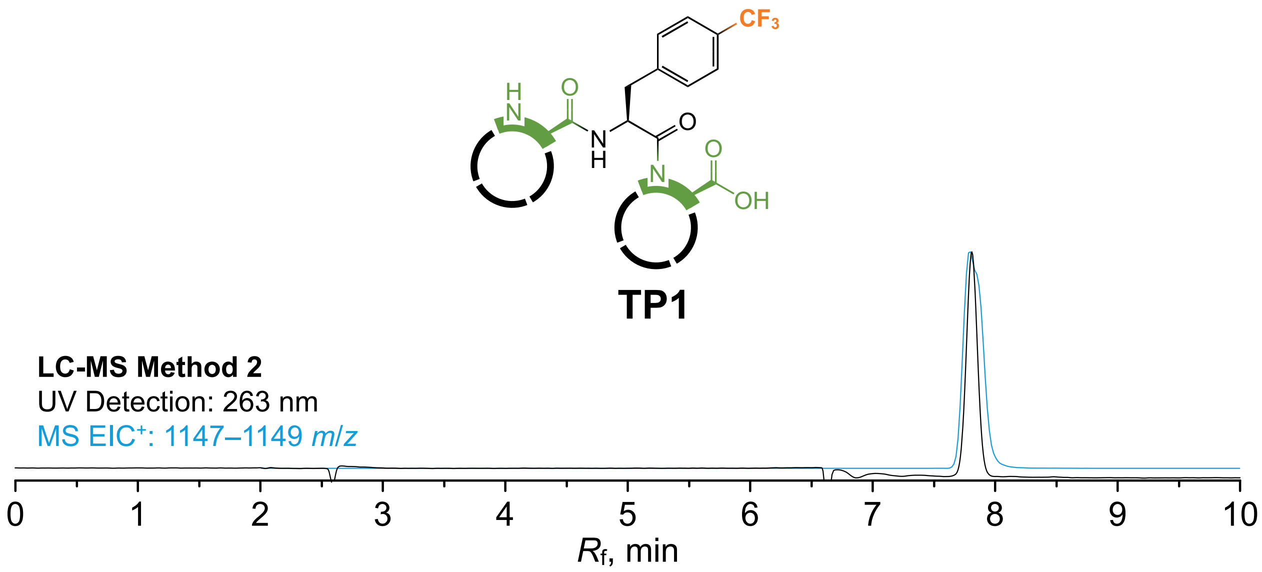

Supplementary Fig. 41. Synthesis and LC-MS chromatogram of TP1. ***Synthesis:*** In a glass vial (20 mL), **II** (19 mg, 11.3 µmol assuming **II**·2TFA, 1.0 equiv.) was dissolved in MeOH (3.6 mL) and H_2O (0.4 mL) followed by addition of aq. LiOH (1.0 M, 325 µL, 325 µmol, 29 equiv.). The resulting solution was stirred at RT for 2 d. The mixture was then purified by preparative HPLC (C18, H_2O/MeCN gradient with 0.1% TFA additive). Fractions with product were joined and lyophilized to give product as white solid. **Yield:** 9.7 mg (62%; 2 steps; based on **II**·2TFA assuming **TP1**·2TFA, M_R = 1376). **ESI-HRMS:** 1148.5084 $[M+H]^+$ (theor. $[C_{48}H_{73}F_3O_{18}N_{11}]^+$ = 1148.5082.

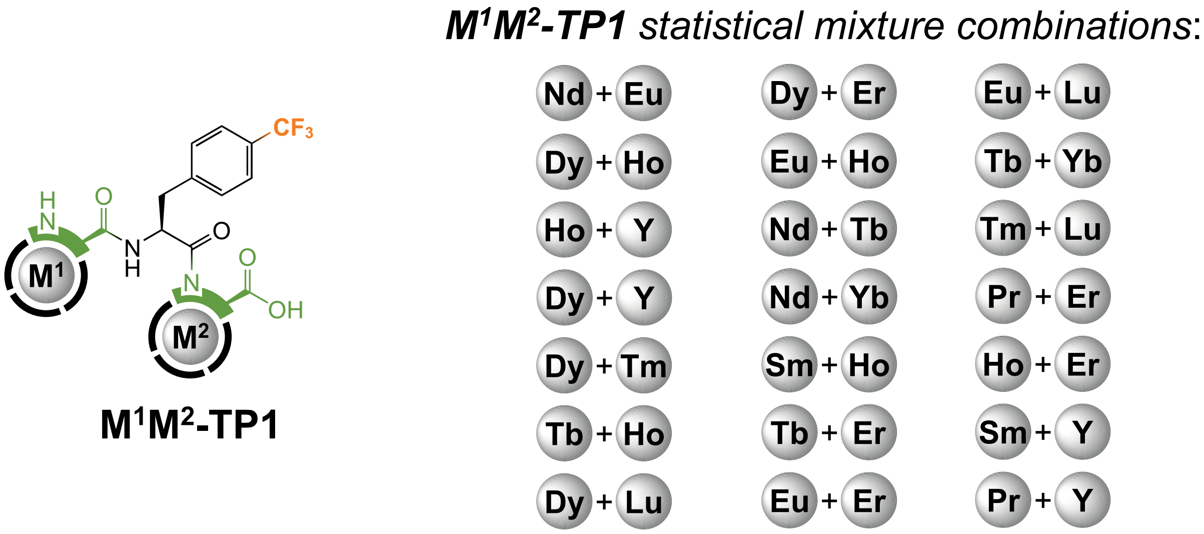

Supplementary Fig. 42. Post-synthesis of M¹M²-TP1 statistical mixtures. ***Synthesis:*** Solid **TP1** (assuming **TP1**·2TFA, M_R = 1376) was dissolved in aq. MOPS/NaOH buffer (pH 7.0) to a final concentration of 2.5 mM **TP1** in 500 mM MOPS/NaOH buffer. In a plastic Eppendorf tube (0.5 mL), buffered stock solution of **TP1** (95 µL, 240 nmol, 1.0 equiv.) was added to a mixture of aq. M^1Cl_3 (100 mM, 2.5 µL, 250 nmol, 1.05 equiv.) and of aq. M^2Cl_3 (100 mM, 2.5 µL, 250 nmol, 1.05 equiv.). The resulting mixture was briefly vortexed and transferred into an NMR insert tube, which was then put into 5 mm NMR tube filled with D_2O and directly used for ^{19}F NMR measurements (21 combinations of M^{3+} cations are listed below; charges were ommited for clarity reasons).

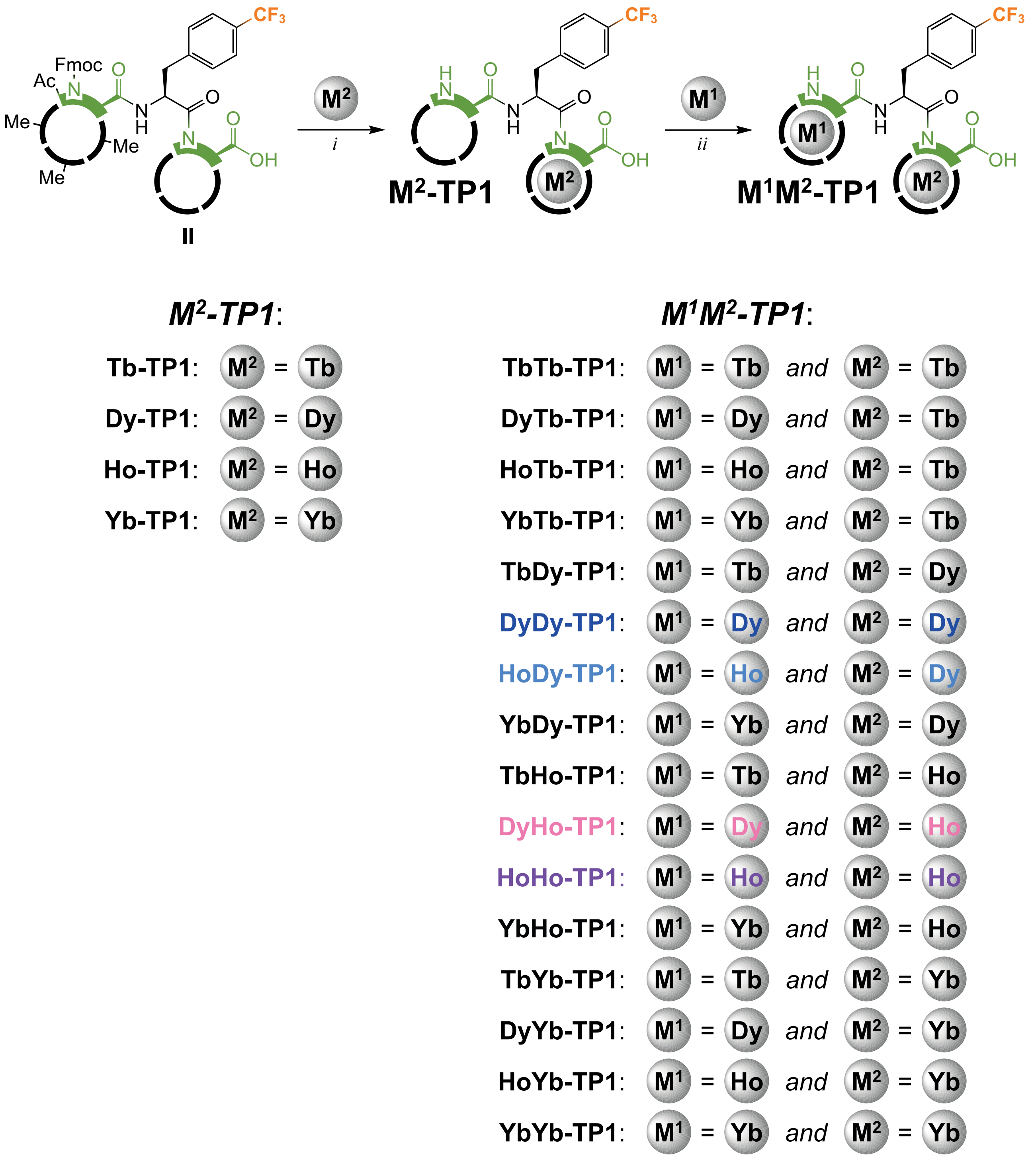

Supplementary Fig. 43. Synthesis of M^1M^2-TP1 tripeptides with Tb^{3+}, Dy^{3+}, Ho^{3+} and Yb^{3+} cations. Charges were omitted for clarity reason. ***Conditions:*** (*i*) M^2Cl_3, aq. MOPS/NaOH buffer (pH 7.0) *followed by* LiOH, H_2O, MeOH; (*ii*) M^1Cl_3, aq. MOPS/NaOH buffer (pH 7.0).

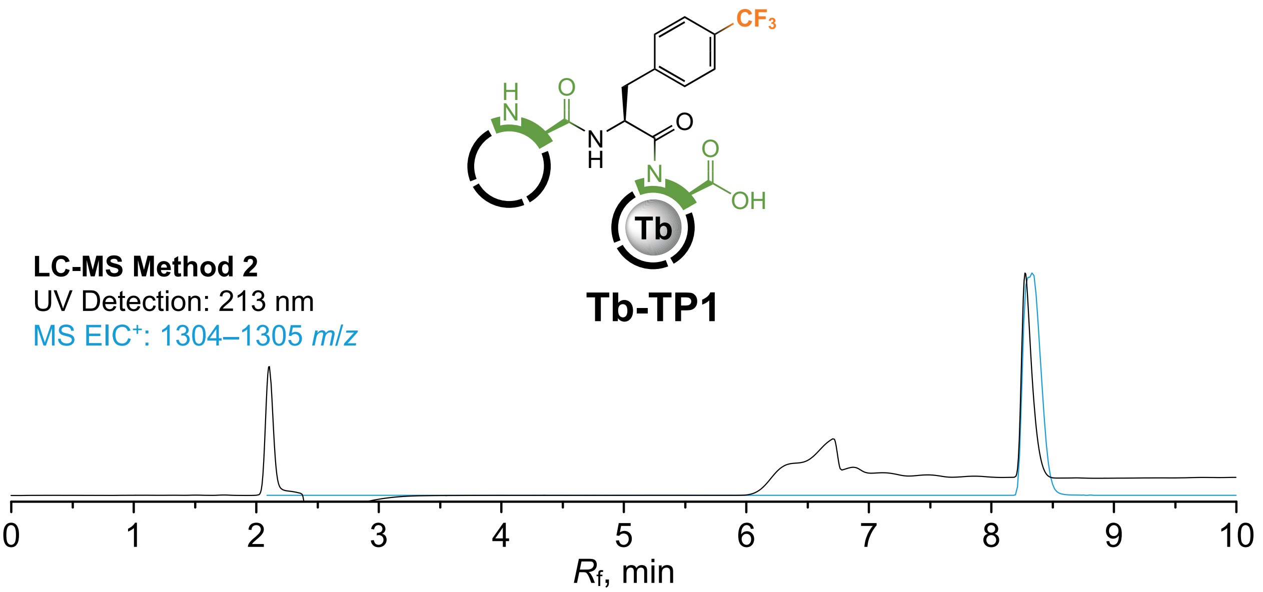

Supplementary Fig. 44. Synthesis and LC-MS chromatogram of intermediate Tb-TP1. ***Synthesis:*** In a glass vial (4 mL), **II** (2.9 mg, 1.7 µmol assuming **II**·2TFA, 1.0 equiv.) was dissolved in aq. MOPS/NaOH buffer (500 mM, pH 7.0, 580 µL, 290 µmol, 170 equiv) followed by addition of aq. $TbCl_3$ (100 mM, 24 µL, 2.4 µmol, 1.4 equiv.). The resulting solution was stirred at RT for 15 mins. The mixture was then purified by preparative HPLC (C18, H_2O/MeCN gradient with 0.1% FA additive). Fractions with product were joined and lyophilized to give product as white solid. The resulting white solid was dissolved in a mixture of MeOH (2.0 mL) and H_2O (150 µL) followed by addition of aq. *LiOH* (1 M, 106 µL, 106 µmol, 62 equiv.) and the mixture was stirred at RT for 5 d. Reaction was then quenched by FA (5.0 µL, 110 µmol, 65 equiv.) and the mixture was evaporated to dryness. The residue was purified by preparative HPLC (C18, H_2O/MeCN gradient with 0.1% FA additive). Fractions with product were joined and lyophilized to give product as white solid. **Yield:** 2.2 mg (90%; 2 steps; based on **II**·2TFA assuming **Tb-TP1**·2.5TFA, M_R = 1419). **ESI-HRMS:** 1304.4091 $[M+H]^+$ (theor. $[C_{48}H_{70}O_{18}N_{11}F_3Tb_1]^+$ = 1304.4100).

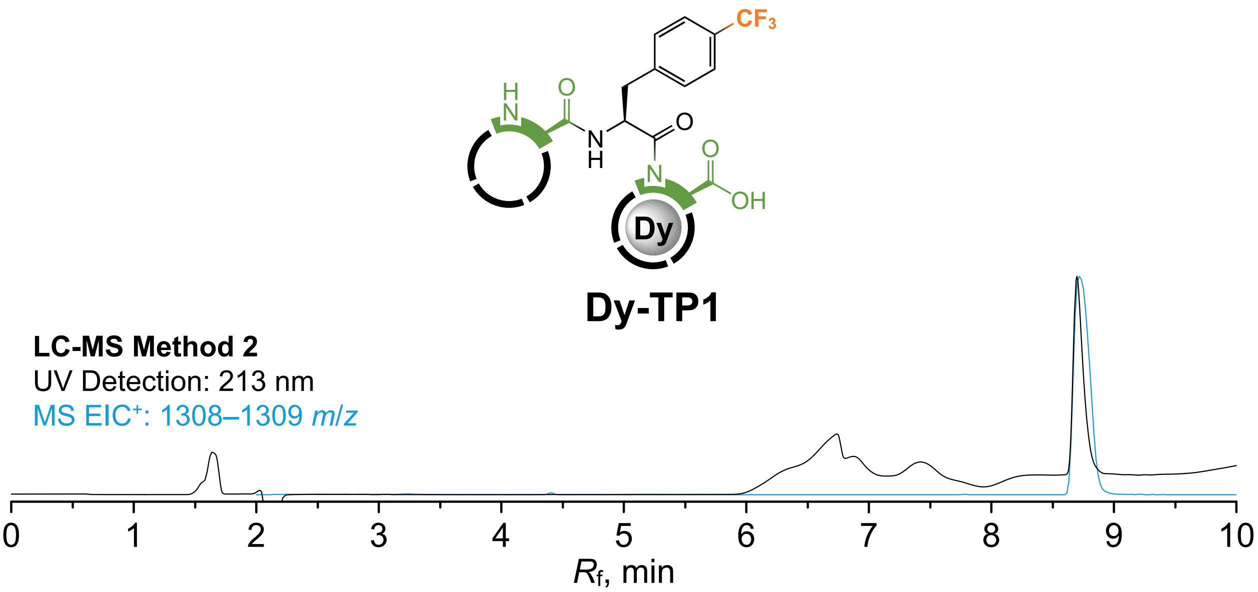

Supplementary Fig. 45. Synthesis and LC-MS chromatogram of intermediate Dy-TP1. ***Synthesis:*** In a glass vial (4 mL), **II** (5.0 mg, 3.0 µmol assuming **II**·2TFA, 1.0 equiv.) was dissolved in aq. MOPS/NaOH buffer (500 mM, pH 7.0, 1.0 mL, 500 µmol, 170 equiv) followed by addition of aq. $DyCl_3$ (100 mM, 41 µL, 4.1 µmol, 1.4 equiv.). The resulting solution was stirred at RT for 15 mins. The mixture was then purified by preparative HPLC (C18, H_2O/MeCN gradient with 0.1% FA additive). Fractions with product were joined and lyophilized to give product as white solid. The resulting white solid was dissolved in a mixture of MeOH (2.4 mL) and H_2O (180 µL) followed by addition of aq. LiOH (1 M, 113 µL, 113 µmol, 38 equiv.) and the mixture was stirred at RT for 5 d. Reaction was then quenched by FA (5.0 µL, 110 µmol, 37 equiv.) and the mixture was evaporated to dryness. The residue was purified by preparative HPLC (C18, H_2O/MeCN gradient with 0.1% FA additive). Fractions with product were joined and lyophilized to give product as white solid. **Yield:** 2.7 mg (64%; 2 steps; based on **II**·2TFA assuming **Dy-TP1**·2.5TFA, M_R = 1423). **ESI-HRMS:** 1309.4130 $[M+H]^+$ (theor. $[C_{48}H_{70}O_{18}N_{11}F_3Dy_1]^+$ = 1309.4139).

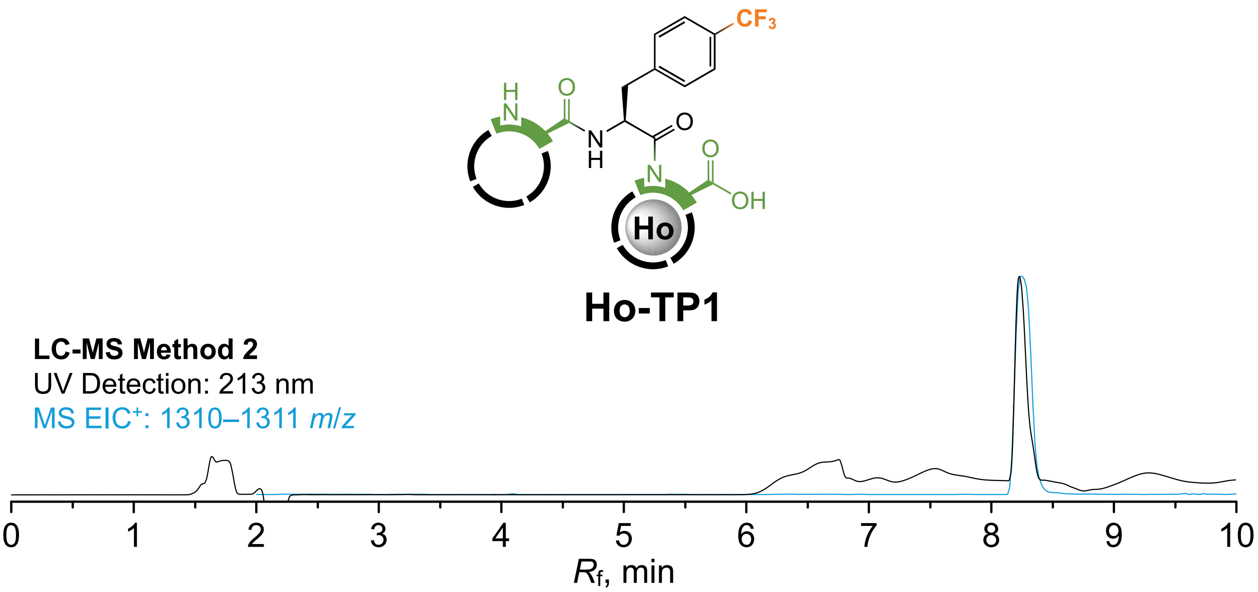

Supplementary Fig. 46. Synthesis and LC-MS chromatogram of intermediate Ho-TP1. ***Synthesis:*** In a glass vial (4 mL), **II** (4.0 mg, 2.4 µmol assuming **II**·2TFA, 1.0 equiv.) was dissolved in aq. MOPS/NaOH buffer (500 mM, pH 7.0, 0.8 mL, 430 µmol, 170 equiv) followed by addition of aq. $HoCl_3$ (100 mM, 33 µL, 3.3 µmol, 1.4 equiv.). The resulting solution was stirred at RT for 15 mins. The mixture was then purified by preparative HPLC (C18, H_2O/MeCN gradient with 0.1% FA additive). Fractions with product were joined and lyophilized to give product as white solid. The resulting white solid was dissolved in a mixture of MeOH (2.0 mL) and H_2O (150 µL) followed by addition of aq. LiOH (1 M, 102 µL, 102 µmol, 43 equiv.) and the mixture was stirred at RT for 5 d. Reaction was then quenched by FA (5.0 µL, 110 µmol, 46 equiv.) and the mixture was evaporated to dryness. The residue was purified by preparative HPLC (C18, H_2O/MeCN gradient with 0.1% FA additive). Fractions with product were joined and lyophilized to give product as white solid. **Yield:** 2.1 mg (61%; 2 steps; based on **II**·2TFA assuming **Ho-TP1**·2.5TFA, M_R = 1425). **ESI-HRMS:** 1310.4146 $[M+H]^+$ (theor. $[C_{48}H_{70}O_{18}N_{11}F_3Ho_1]^+$ = 1310.4150).

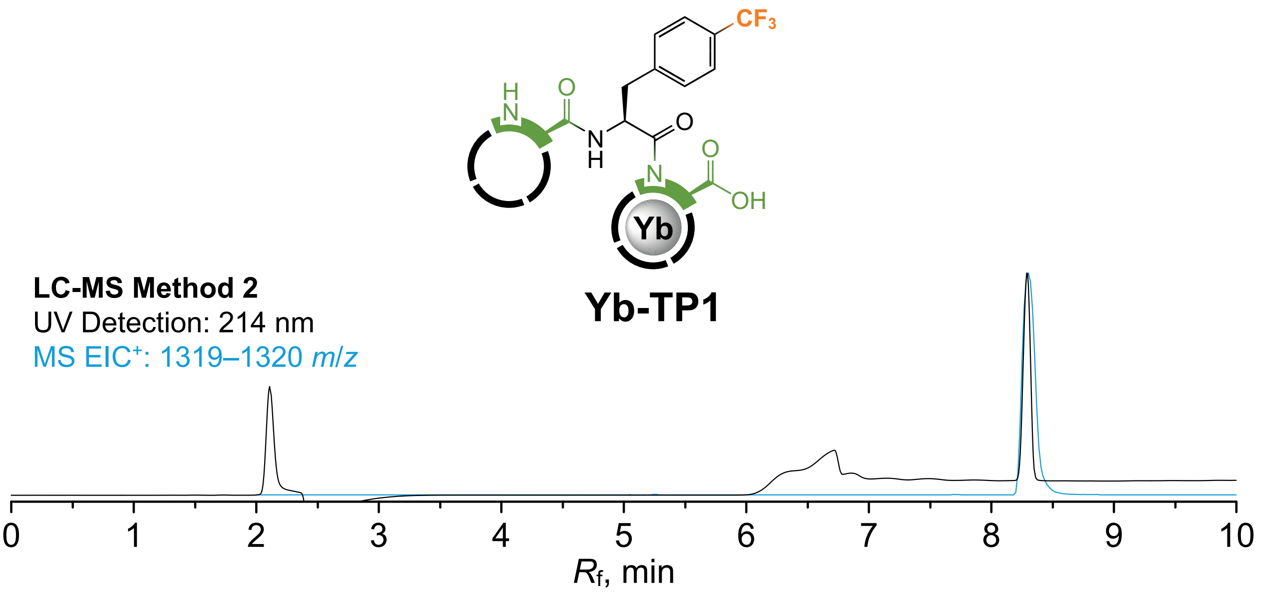

Supplementary Fig. 47. Synthesis and LC-MS chromatogram of intermediate Yb-TP1. ***Synthesis:*** In a glass vial (4 mL), **II** (2.9 mg, 1.7 µmol assuming **II**·2TFA, 1.0 equiv.) was dissolved in aq. MOPS/NaOH buffer (500 mM, pH 7.0, 580 µL, 500 µmol, 170 equiv) followed by addition of aq. $YbCl_3$ (100 mM, 24 µL, 2.4 µmol, 1.4 equiv.). The resulting solution was stirred at RT for 15 mins. The mixture was then purified by preparative HPLC (C18, H_2O/MeCN gradient with 0.1% FA additive). Fractions with product were joined and lyophilized to give product as white solid. The resulting white solid was dissolved in a mixture of MeOH (2.0 mL) and H_2O (150 µL) followed by addition of aq. LiOH (1 M, 104 µL, 104 µmol, 61 equiv.) and the mixture was stirred at RT for 5 d. Reaction was then quenched by FA (5.0 µL, 110 µmol, 65 equiv.) and the mixture was evaporated to dryness. The residue was purified by preparative HPLC (C18, H_2O/MeCN gradient with 0.1% FA additive). Fractions with product were joined and lyophilized to give product as white solid. **Yield:** 1.6 mg (65%; 2 steps; based on **II**·2TFA assuming **Yb-TP1**·2.5TFA, M_R = 1433). **ESI-HRMS:** 1319.4240 $[M+H]^+$ (theor. $[C_{48}H_{70}O_{18}N_{11}F_3Yb_1]^+$ = 1319.4236).

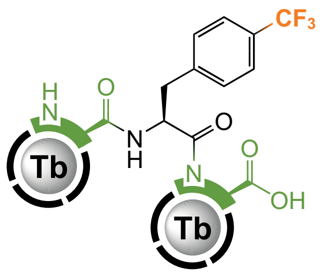

TbTb-TP1

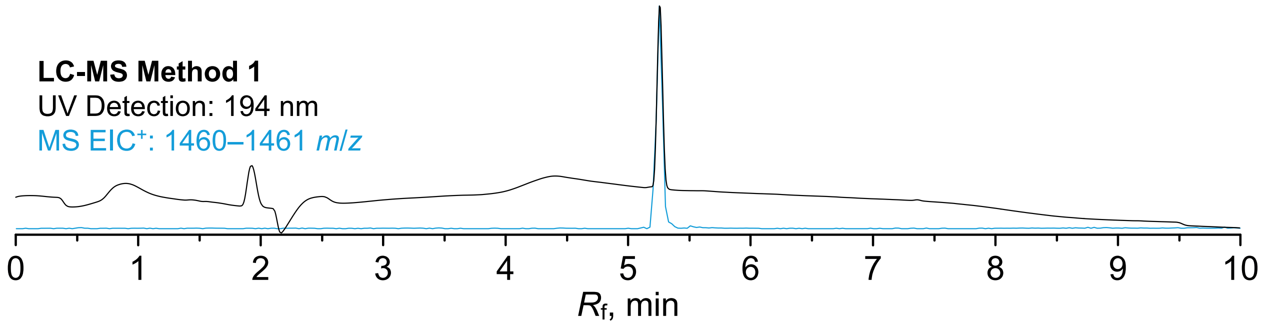

Supplementary Fig. 48. Synthesis and LC-MS chromatogram of TbTb-TP1. ***Synthesis:*** In a glass vial (4 mL), **Tb-TP1** (0.6 mg, ~0.4 µmol assuming **Tb-TP1**·2.5FA, 1.0 equiv.) was dissolved in aq. MOPS/NaOH buffer (500 mM, pH 7.0, 500 µL, 250 µmol, ~600 equiv.) followed by addition of aq. $TbCl_3$ (100 mM, 5 µL, 0.5 µmol, ~1.2 equiv.) and the resulting solution was stirred at RT for 15 mins. The mixture was then purified by preparative HPLC (C18, H_2O/MeCN gradient with 0.1% TFA additive). Fractions with product were joined and lyophilized to give product as white solid. **Yield:** ~0.3 mg. **NMR (aq. MOPS pH = 7.0, external D_2O):** ^{19}**F** (470.4 MHz, T = 298.2 K) δ_F –55.76 (CF_3, s). **ESI-HRMS:** 730.6594 $[M+2H]^{2+}$ (theor. $[C_{48}H_{68}O_{18}N_{11}F_3Tb_2]^{2+}$ = 730.6596).

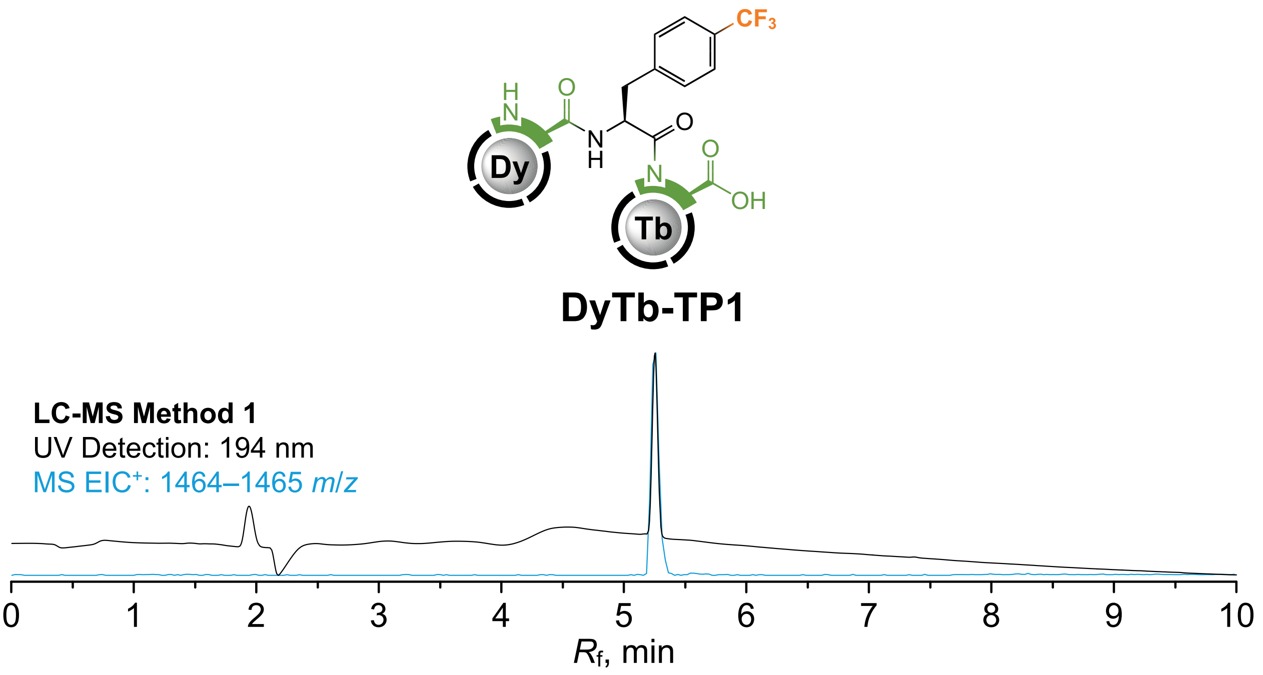

Supplementary Fig. 49. Synthesis and LC-MS chromatogram of DyTb-TP1. ***Synthesis:*** In a glass vial (4 mL), **Tb-TP1** (0.6 mg, ~0.4 µmol assuming **Tb-TP1**·2.5FA, 1.0 equiv.) was dissolved in aq. MOPS/NaOH buffer (500 mM, pH 7.0, 500 µL, 250 µmol, ~600 equiv.) followed by addition of aq. $DyCl_3$ (100 mM, 5 µL, 0.5 µmol, ~1.2 equiv.) and the resulting solution was stirred at RT for 15 mins. The mixture was then purified by preparative HPLC (C18, H_2O/MeCN gradient with 0.1% TFA additive). Fractions with product were joined and lyophilized to give product as white solid. **Yield:** ~0.3 mg. **NMR (aq. MOPS pH = 7.0, external D_2O):** ^{19}**F** (470.4 MHz, $T = 298.2$ K) δ_F –67.29 (C*F*$_3$, s). **ESI-HRMS:** 731.6605 $[M+2H]^{2+}$ (theor. $[C_{48}H_{68}O_{18}N_{11}F_3Dy_1Tb_1]^{2+}$ = 731.6604).

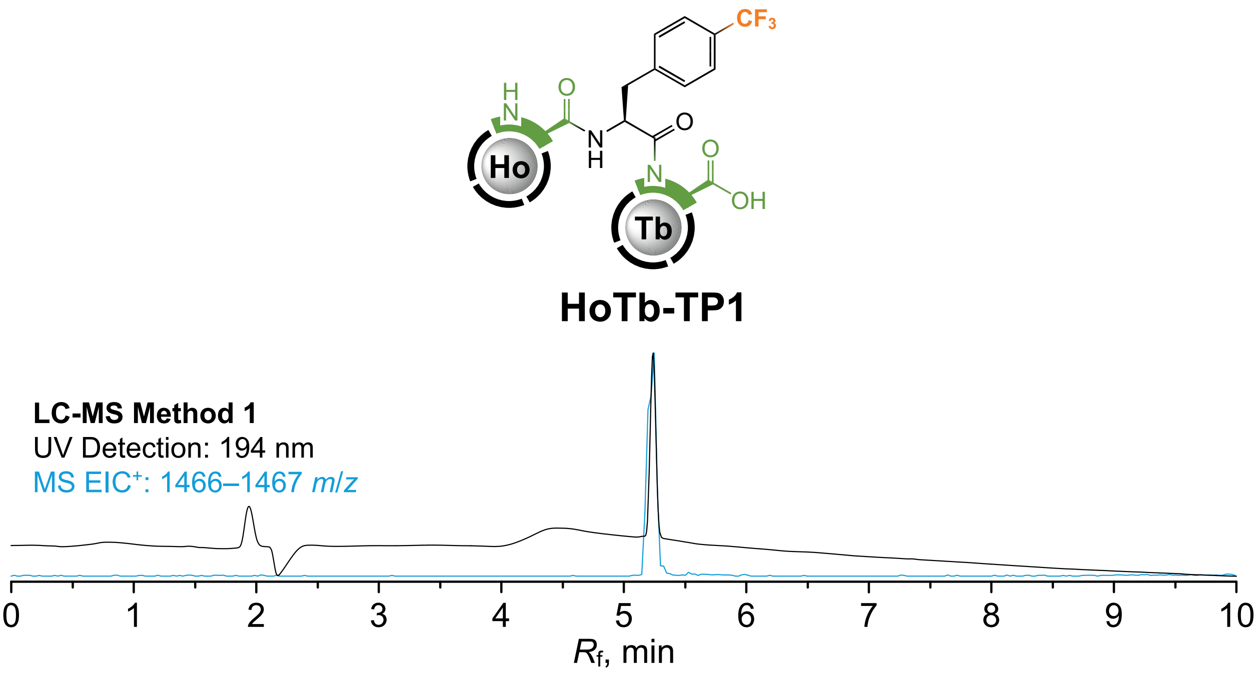

Supplementary Fig. 50. Synthesis and LC-MS chromatogram of HoTb-TP1. ***Synthesis:*** In a glass vial (4 mL), **Tb-TP1** (0.5 mg, ~0.4 µmol assuming **Tb-TP1**·2.5FA, 1.0 equiv.) was dissolved in aq. *MOPS/NaOH buffer* (500 mM, pH 7.0, 500 µL, 250 µmol, ~700 equiv.) followed by addition of aq. $HoCl_3$ (100 mM, 5 µL, 0.5 µmol, ~1.4 equiv.) and the resulting solution was stirred at RT for 15 mins. The mixture was then purified by preparative HPLC (C18, H_2O/MeCN gradient with 0.1% TFA additive). Fractions with product were joined and lyophilized to give product as white solid. **Yield:** ~0.3 mg. **NMR (aq. MOPS pH = 7.0, external D_2O):** ^{19}**F** (470.4 MHz, T = 298.2 K) δ_F –64.81 (CF_3, s). **ESI-HRMS:** 733.6620 $[M+2H]^{2+}$ (theor. $[C_{48}H_{68}O_{18}N_{11}F_3Ho_1Tb_1]^{2+}$ = 733.6621).

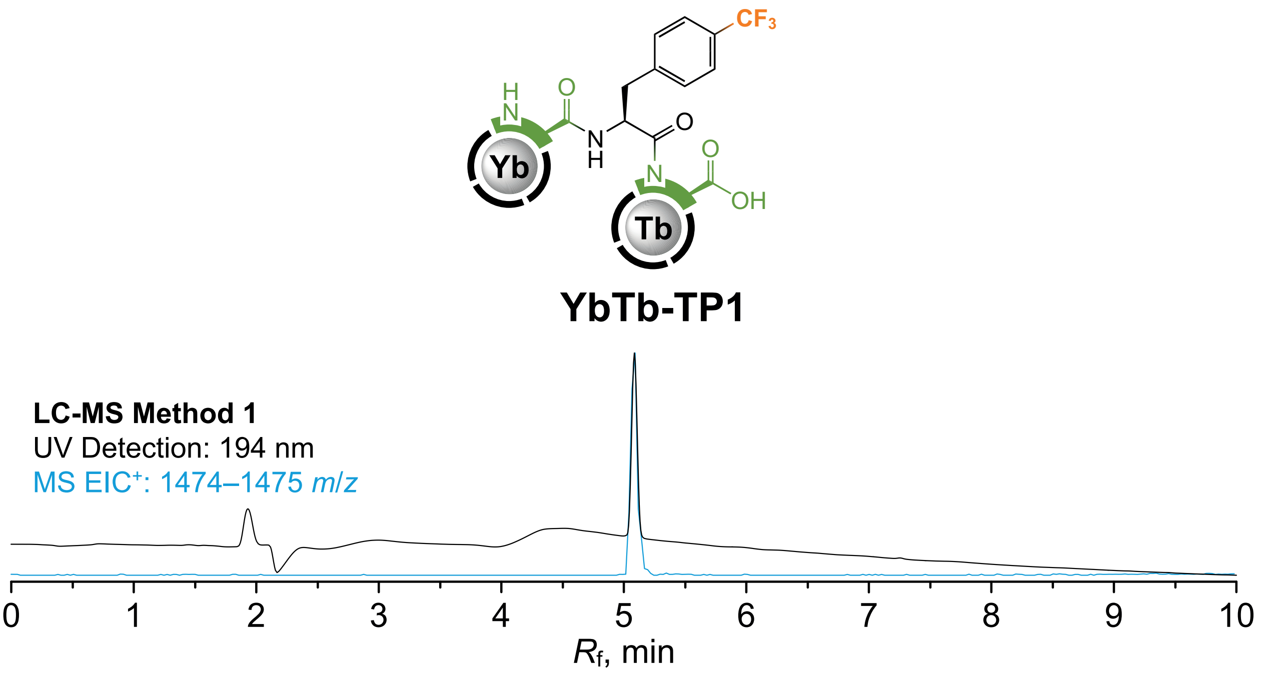

Supplementary Fig. 51. Synthesis and LC-MS chromatogram of YbTb-TP1. ***Synthesis:*** In a glass vial (4 mL), **Tb-TP1** (0.5 mg, ~0.4 µmol assuming **Tb-TP1**·2.5FA, 1.0 equiv.) was dissolved in aq. MOPS/NaOH buffer (500 mM, pH 7.0, 500 µL, 250 µmol, ~700 equiv.) followed by addition of aq. $YbCl_3$ (100 mM, 5 µL, 0.5 µmol, ~1.4 equiv.) and the resulting solution was stirred at RT for 15 mins. The mixture was then purified by preparative HPLC (C18, H_2O/MeCN gradient with 0.1% TFA additive). Fractions with product were joined and lyophilized to give product as white solid. **Yield:** ~0.3 mg. **NMR (aq. MOPS pH = 7.0, external D_2O):** ^{19}**F** (470.4 MHz, T = 298.2 K) δ_F –57.56 (CF_3, s). **ESI-HRMS:** 738.1665 $[M+2H]^{2+}$ (theor. $[C_{48}H_{68}O_{18}N_{11}F_3Yb_1Tb_1]^{2+}$ = 738.1664).

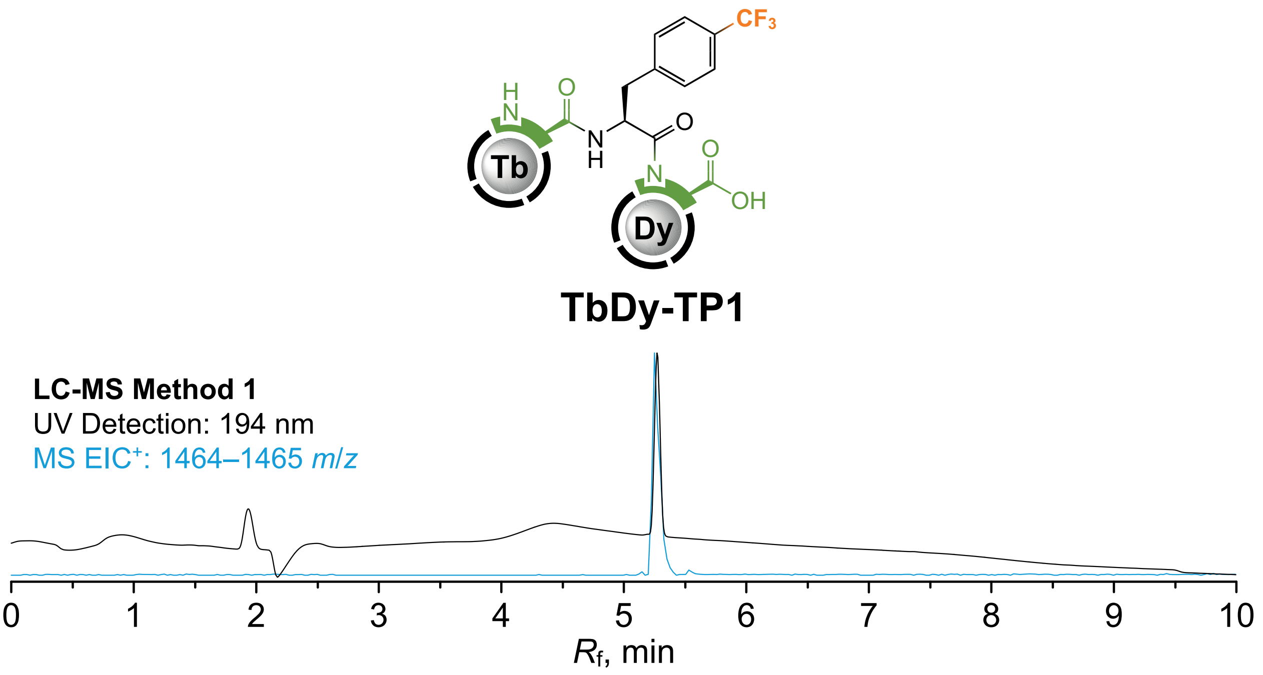

Supplementary Fig. 52. Synthesis and LC-MS chromatogram of TbDy-TP1. ***Synthesis:*** In a glass vial (4 mL), **Dy-TP1** (0.5 mg, ~0.4 µmol assuming **Dy-TP1**·2.5FA, 1.0 equiv.) was dissolved in aq. MOPS/NaOH buffer (500 mM, pH 7.0, 250 µL, 125 µmol, ~350 equiv.) followed by addition of aq. $TbCl_3$ (100 mM, 5 µL, 0.5 µmol, ~1.4 equiv.) and the resulting solution was stirred at RT for 15 mins. The mixture was then purified by preparative HPLC (C18, H_2O/MeCN gradient with 0.1% TFA additive). Fractions with product were joined and lyophilized to give product as white solid. **Yield:** ~0.4 mg. **NMR (aq. MOPS pH = 7.0, external D_2O):** ^{19}**F** (470.4 MHz, T = 298.2 K) δ_F –55.55 (CF_3, s). **ESI-HRMS:** 733.1610 $[M+2H]^{2+}$ (theor. $[C_{48}H_{68}O_{18}N_{11}F_3Tb_1Dy_1]^{2+}$ = 733.1615).

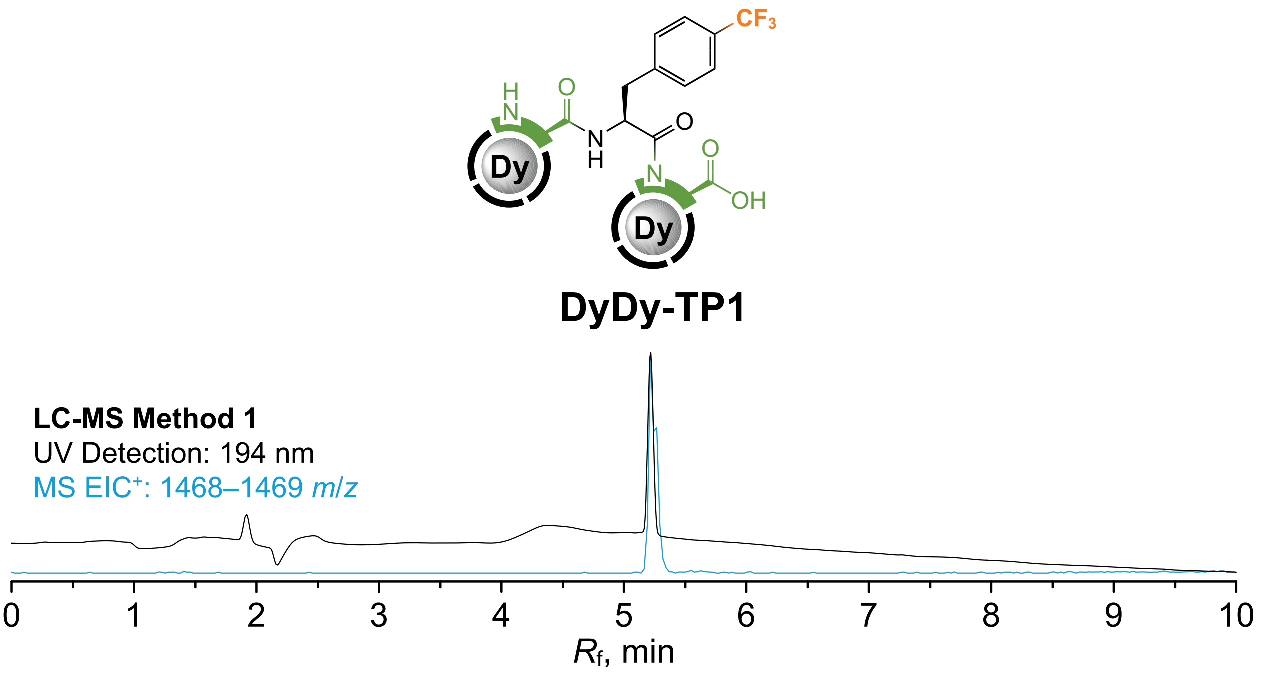

Supplementary Fig. 53. Synthesis and LC-MS chromatogram of DyDy-TP1. ***Synthesis:*** In a glass vial (4 mL), **Dy-TP1** (1.0 mg, ~0.7 µmol assuming **Dy-TP1**·2.5FA, 1.0 equiv.) was dissolved in aq. MOPS/NaOH buffer (500 mM, pH 7.0, 500 µL, 250 µmol, ~350 equiv.) followed by addition of aq. $DyCl_3$ (100 mM, 9 µL, 0.9 µmol, ~1.3 equiv.) and the resulting solution was stirred at RT for 15 mins. The mixture was then purified by preparative HPLC (C18, H_2O/MeCN gradient with 0.1% TFA additive). Fractions with product were joined and lyophilized to give product as white solid. **Yield:** ~1.0 mg. **NMR (aq. MOPS pH = 7.0, external D_2O):** ^{19}**F** (470.4 MHz, T = 298.2 K) δ_F –57.02 (CF_3, s). **ESI-HRMS:** 735.6632 $[M+2H]^{2+}$ (theor. $[C_{48}H_{68}O_{18}N_{11}F_3Dy_2]^{2+}$ = 735.6634).

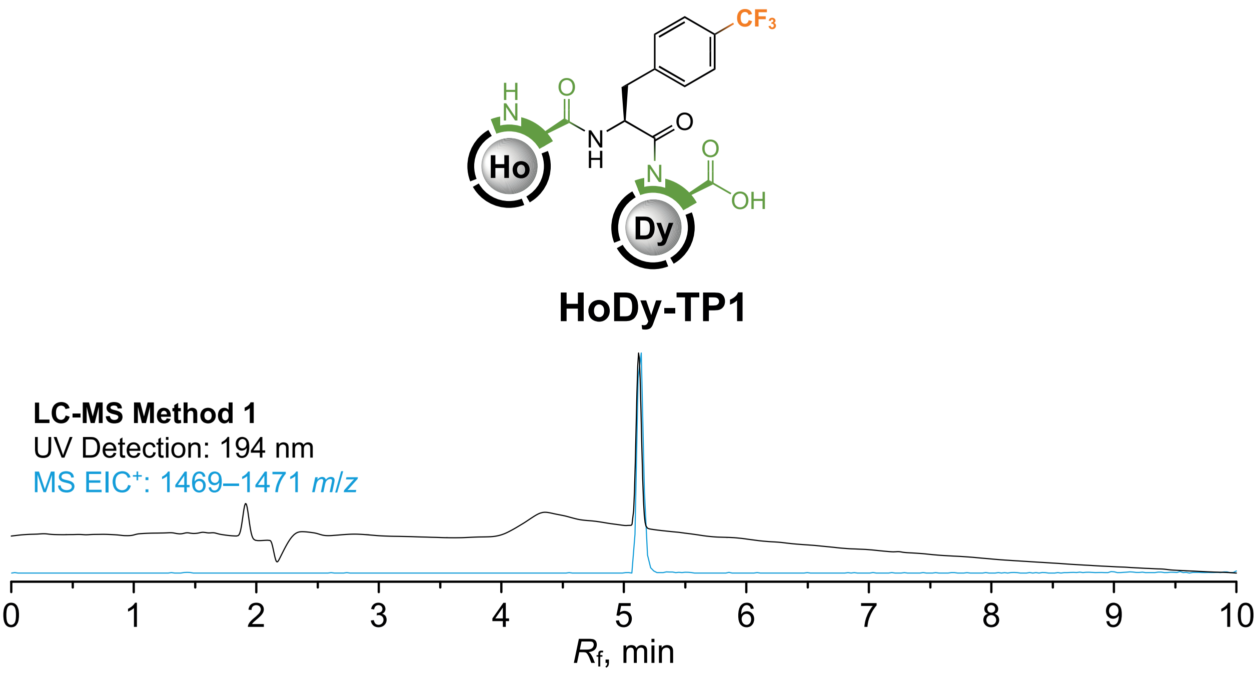

Supplementary Fig. 54. Synthesis and LC-MS chromatogram of HoDy-TP1. ***Synthesis:*** In a glass vial (4 mL), **Dy-TP1** (0.5 mg, ~0.4 µmol assuming **Dy-TP1**·2.5FA, 1.0 equiv.) was dissolved in aq. MOPS/NaOH buffer (500 mM, pH 7.0, 250 µL, 125 µmol, ~350 equiv.) followed by addition of aq. $HoCl_3$ (100 mM, 5 µL, 0.5 µmol, ~1.4 equiv.) and the resulting solution was stirred at RT for 15 mins. The mixture was then purified by preparative HPLC (C18, H_2O/MeCN gradient with 0.1% TFA additive). Fractions with product were joined and lyophilized to give product as white solid. **Yield:** ~0.5 mg. **NMR (aq. MOPS pH = 7.0, external D_2O):** ^{19}**F** (470.4 MHz, T = 298.2 K) δ_F –54.61 (CF_3, s). **ESI-HRMS:** 736.1641 $[M+2H]^{2+}$ (theor. $[C_{48}H_{68}O_{18}N_{11}F_3Ho_1Dy_1]^{2+}$ = 736.1640).

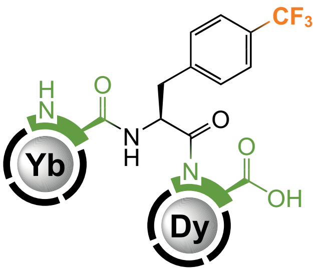

YbDy-TP1

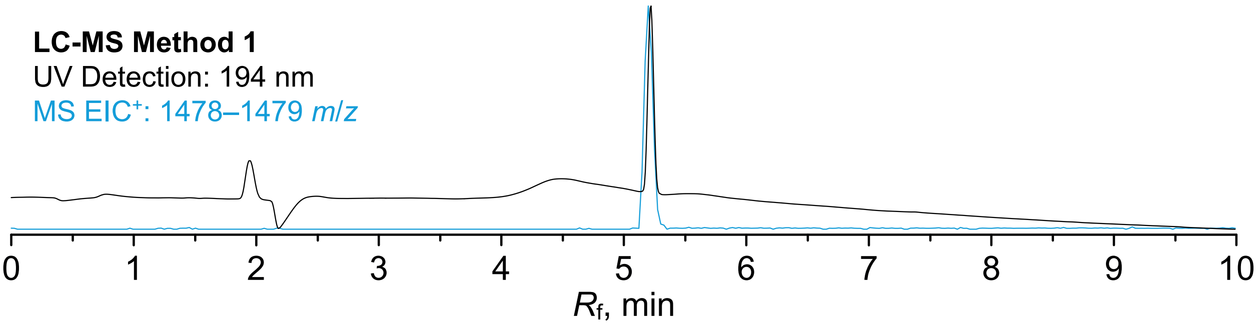

Supplementary Fig. 55. Synthesis and LC-MS chromatogram of YbDy-TP1. ***Synthesis:*** In a glass vial (4 mL), **Dy-TP1** (0.5 mg, ~0.4 µmol assuming **Dy-TP1**·2.5FA, 1.0 equiv.) was dissolved in aq. MOPS/NaOH buffer (500 mM, pH 7.0, 250 µL, 125 µmol, ~350 equiv.) followed by addition of aq. $YbCl_3$ (100 mM, 5 µL, 0.5 µmol, ~1.4 equiv.) and the resulting solution was stirred at RT for 15 mins. The mixture was then purified by preparative HPLC (C18, H_2O/MeCN gradient with 0.1% TFA additive). Fractions with product were joined and lyophilized to give product as white solid. **Yield:** ~0.4 mg. **NMR (aq. MOPS pH = 7.0, external D_2O):** ^{19}**F** (470.4 MHz, T = 298.2 K) δ_F –47.38 (CF_3, s). **ESI-HRMS:** 740.6698 $[M+2H]^{2+}$ (theor. $[C_{48}H_{68}O_{18}N_{11}F_3Yb_1Dy_1]^{2+}$ = 740.6683).

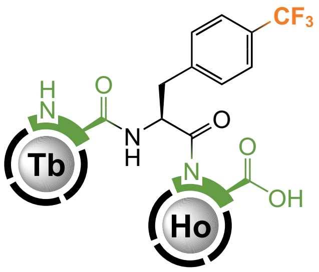

TbHo-TP1

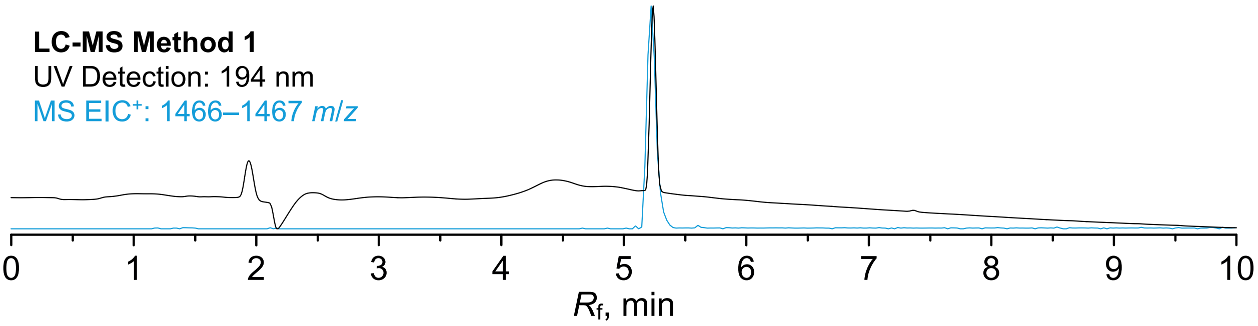

Supplementary Fig. 56. Synthesis and LC-MS chromatogram of TbHo-TP1. ***Synthesis:*** In a glass vial (4 mL), **Ho-TP1** (0.5 mg, ~0.4 µmol assuming **Ho-TP1**·2.5FA, 1.0 equiv.) was dissolved in aq. MOPS/NaOH buffer (500 mM, pH 7.0, 250 µL, 125 µmol, ~350 equiv.) followed by addition of aq. $TbCl_3$ (100 mM, 5 µL, 0.5 µmol, ~1.4 equiv.) and the resulting solution was stirred at RT for 15 mins. The mixture was then purified by preparative HPLC (C18, H_2O/MeCN gradient with 0.1% TFA additive). Fractions with product were joined and lyophilized to give product as white solid. **Yield:** ~0.4 mg. **NMR (aq. MOPS pH = 7.0, external D_2O):** ^{19}**F** (470.4 MHz, T = 298.2 K) δ_F –60.91 (CF_3, s). **ESI-HRMS:** 744.6528 $[M+2H]^{2+}$ (theor. $[C_{48}H_{68}O_{18}N_{11}F_3Tb_1Ho_1]^{2+}$ = 744.6531).

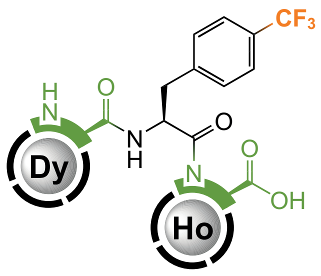

DyHo-TP1

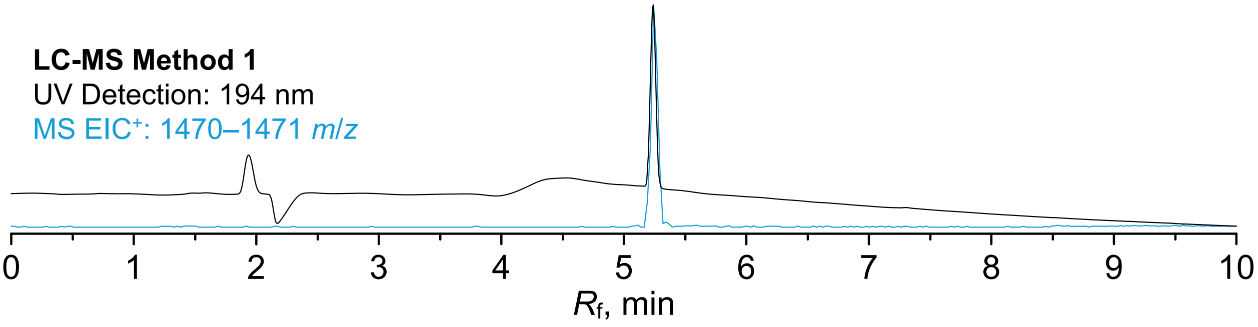

Supplementary Fig. 57. Synthesis and LC-MS chromatogram of DyHo-TP1. ***Synthesis:*** In a glass vial (4 mL), **Ho-TP1** (0.4 mg, ~0.3 µmol assuming **Ho-TP1**·2.5FA, 1.0 equiv.) was dissolved in aq. MOPS/NaOH buffer (500 mM, pH 7.0, 250 µL, 125 µmol, ~900 equiv.) followed by addition of aq. $DyCl_3$ (100 mM, 3 µL, 0.3 µmol, ~1.1 equiv.) and the resulting solution was stirred at RT for 15 mins. The mixture was then purified by preparative HPLC (C18, H_2O/MeCN gradient with 0.1% TFA additive). Fractions with product were joined and lyophilized to give product as white solid. **Yield:** ~0.4 mg. **NMR (aq. MOPS pH = 7.0, external D_2O):** ^{19}**F** (470.4 MHz, T = 298.2 K) δ_F –62.75 (CF_3, s). **ESI-HRMS:** 736.1643 $[M+2H]^{2+}$ (theor. $[C_{48}H_{68}O_{18}N_{11}F_3Dy_1Ho_1]^{2+}$ = 736.1640).

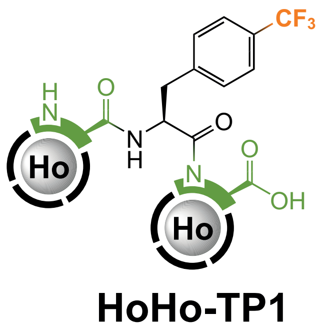

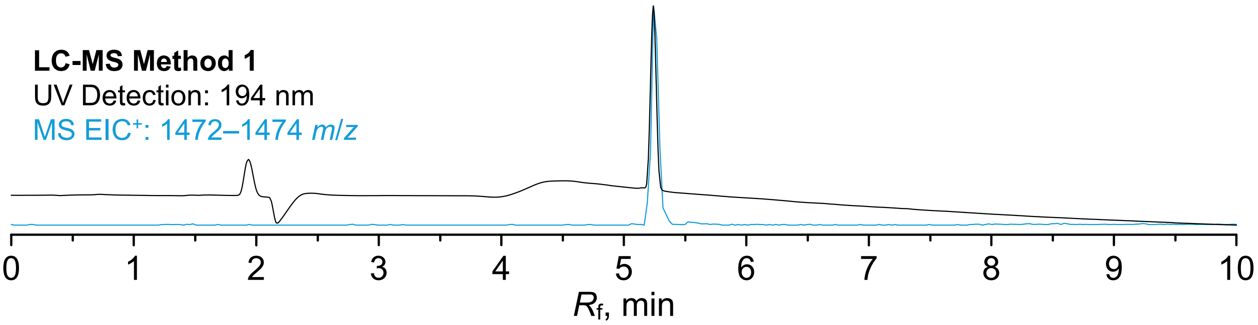

Supplementary Fig. 58. Synthesis and LC-MS chromatogram of HoHo-TP1. ***Synthesis:*** In a glass vial (4 mL), **Ho-TP1** (0.4 mg, ~0.3 µmol assuming **Ho-TP1**·2.5FA, 1.0 equiv.) was dissolved in aq. MOPS/NaOH buffer (500 mM, pH 7.0, 250 µL, 125 µmol, ~900 equiv.) followed by addition of aq. $HoCl_3$ (100 mM, 3 µL, 0.3 µmol, ~1.1 equiv.) and the resulting solution was stirred at RT for 15 mins. The mixture was then purified by preparative HPLC (C18, H_2O/MeCN gradient with 0.1% TFA additive). Fractions with product were joined and lyophilized to give product as white solid. **Yield:** ~0.4 mg. **NMR (aq. MOPS pH = 7.0, external D_2O):** ^{19}**F** (470.4 MHz, T = 298.2 K) δ_F –60.20 (CF_3, s). **ESI-HRMS:** 736.6644 $[M+2H]^{2+}$ (theor. $[C_{48}H_{68}O_{18}N_{11}F_3Ho_2]^{2+}$ = 736.6646).

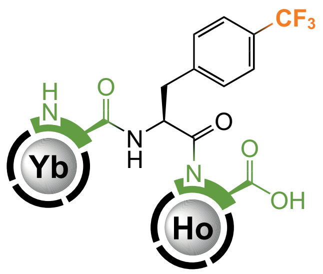

YbHo-TP1

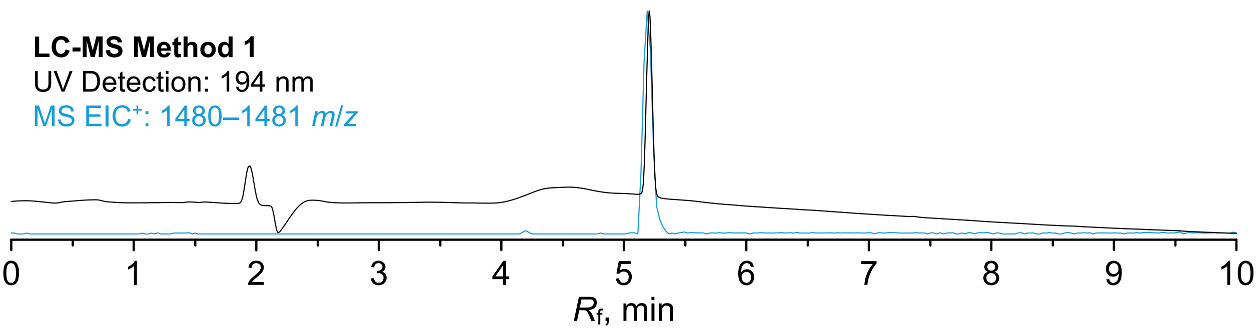

Supplementary Fig. 59. Synthesis and LC-MS chromatogram of YbHo-TP1. ***Synthesis:*** In a glass vial (4 mL), **Ho-TP1** (0.5 mg, ~0.4 µmol assuming **Ho-TP1**·2.5FA, 1.0 equiv.) was dissolved in aq. MOPS/NaOH buffer (500 mM, pH 7.0, 250 µL, 125 µmol, ~350 equiv.) followed by addition of aq. $YbCl_3$ (100 mM, 5 µL, 0.5 µmol, ~1.4 equiv.) and the resulting solution was stirred at RT for 15 mins. The mixture was then purified by preparative HPLC (C18, H_2O/MeCN gradient with 0.1% TFA additive). Fractions with product were joined and lyophilized to give product as white solid. **Yield:** ~0.3 mg. **NMR (aq. MOPS pH = 7.0, external D_2O):** ^{19}**F** (470.4 MHz, T = 298.2 K) δ_F –53.02 (CF_3, s). **ESI-HRMS:** 741.1700 $[M+2H]^{2+}$ (theor. $[C_{48}H_{68}O_{18}N_{11}F_3Yb_1Ho_1]^{2+}$ = 741.1688).

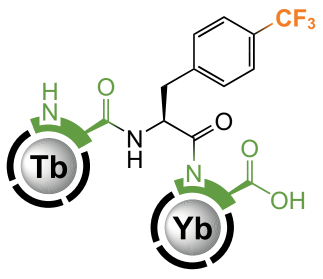

TbYb-TP1

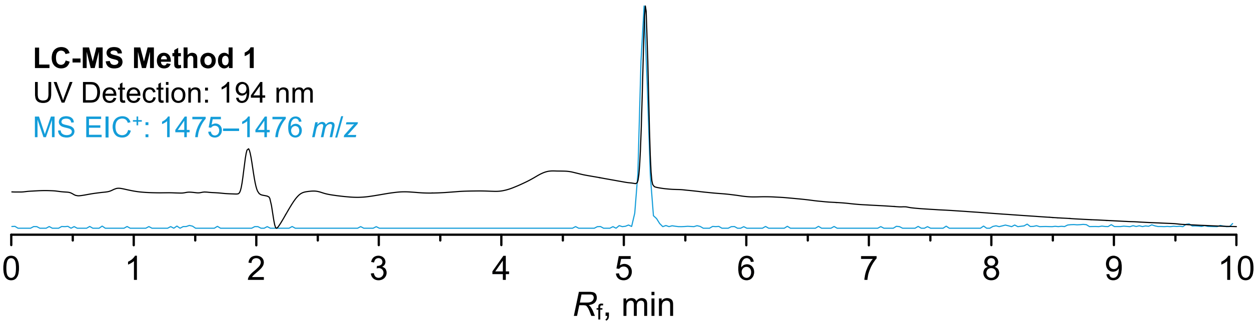

Supplementary Fig. 60. Synthesis and LC-MS chromatogram of TbYb-TP1. ***Synthesis:*** In a glass vial (4 mL), **Yb-TP1** (0.4 mg, ~0.3 µmol assuming **Yb-TP1**·2.5FA, 1.0 equiv.) was dissolved in aq. MOPS/NaOH buffer (500 mM, pH 7.0, 250 µL, 125 µmol, ~900 equiv.) followed by addition of aq. $TbCl_3$ (100 mM, 4 µL, 0.4 µmol, ~1.4 equiv.) and the resulting solution was stirred at RT for 15 mins. The mixture was then purified by preparative HPLC (C18, H_2O/MeCN gradient with 0.1% TFA additive). Fractions with product were joined and lyophilized to give product as white solid. **Yield:** ~0.3 mg. **NMR (aq. MOPS pH = 7.0, external D_2O):** ^{19}**F** (470.4 MHz, T = 298.2 K) δ_F –69.10 (CF_3, s). **ESI-HRMS:** 738.1662 $[M+2H]^{2+}$ (theor. $[C_{48}H_{68}O_{18}N_{11}F_3Tb_1Yb_1]^{2+}$ = 738.1664).

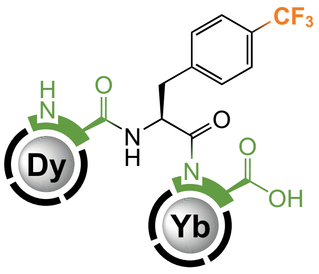

DyYb-TP1

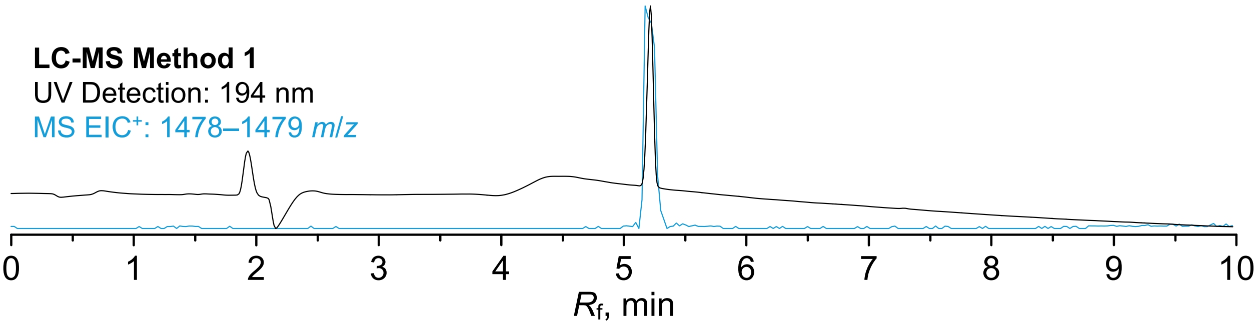

Supplementary Fig. 61. Synthesis and LC-MS chromatogram of DyYb-TP1. ***Synthesis:*** In a glass vial (4 mL), **Yb-TP1** (0.4 mg, ~0.3 µmol assuming **Yb-TP1**·2.5FA, 1.0 equiv.) was dissolved in aq. MOPS/NaOH buffer (500 mM, pH 7.0, 250 µL, 125 µmol, ~900 equiv.) followed by addition of aq. $DyCl_3$ (100 mM, 4 µL, 0.4 µmol, ~1.4 equiv.) and the resulting solution was stirred at RT for 15 mins. The mixture was then purified by preparative HPLC (C18, H_2O/MeCN gradient with 0.1% TFA additive). Fractions with product were joined and lyophilized to give product as white solid. **Yield:** ~0.4 mg. **NMR (aq. MOPS pH = 7.0, external D_2O):** ^{19}**F** (470.4 MHz, T = 298.2 K) δ_F –71.04 (CF_3, s). **ESI-HRMS:** 740.6689 $[M+2H]^{2+}$ (theor. $[C_{48}H_{68}O_{18}N_{11}F_3Dy_1Yb_1]^{2+}$ = 740.6683).

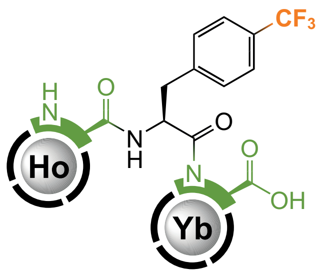

HoYb-TP1

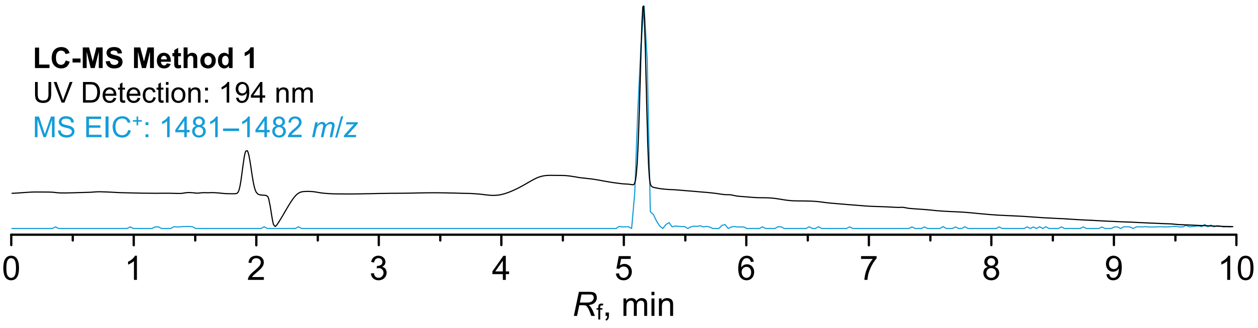

Supplementary Fig. 62. Synthesis and LC-MS chromatogram of HoYb-TP1. ***Synthesis:*** In a glass vial (4 mL), **Yb-TP1** (0.4 mg, ~0.3 µmol assuming **Yb-TP1**·2.5FA, 1.0 equiv.) was dissolved in aq. MOPS/NaOH buffer (500 mM, pH 7.0, 250 µL, 125 µmol, ~900 equiv.) followed by addition of aq. $HoCl_3$ (100 mM, 4 µL, 0.4 µmol, ~1.4 equiv.) and the resulting solution was stirred at RT for 15 mins. The mixture was then purified by preparative HPLC (C18, H_2O/MeCN gradient with 0.1% TFA additive). Fractions with product were joined and lyophilized to give product as white solid. **Yield:** ~0.3 mg. **NMR (aq. MOPS pH = 7.0, external D_2O):** ^{19}**F** (470.4 MHz, T = 298.2 K) δ_F –68.39 (CF_3, s). **ESI-HRMS:** 741.1687 $[M+2H]^{2+}$ (theor. $[C_{48}H_{68}O_{18}N_{11}F_3Ho_1Yb_1]^{2+}$ = 741.1688).

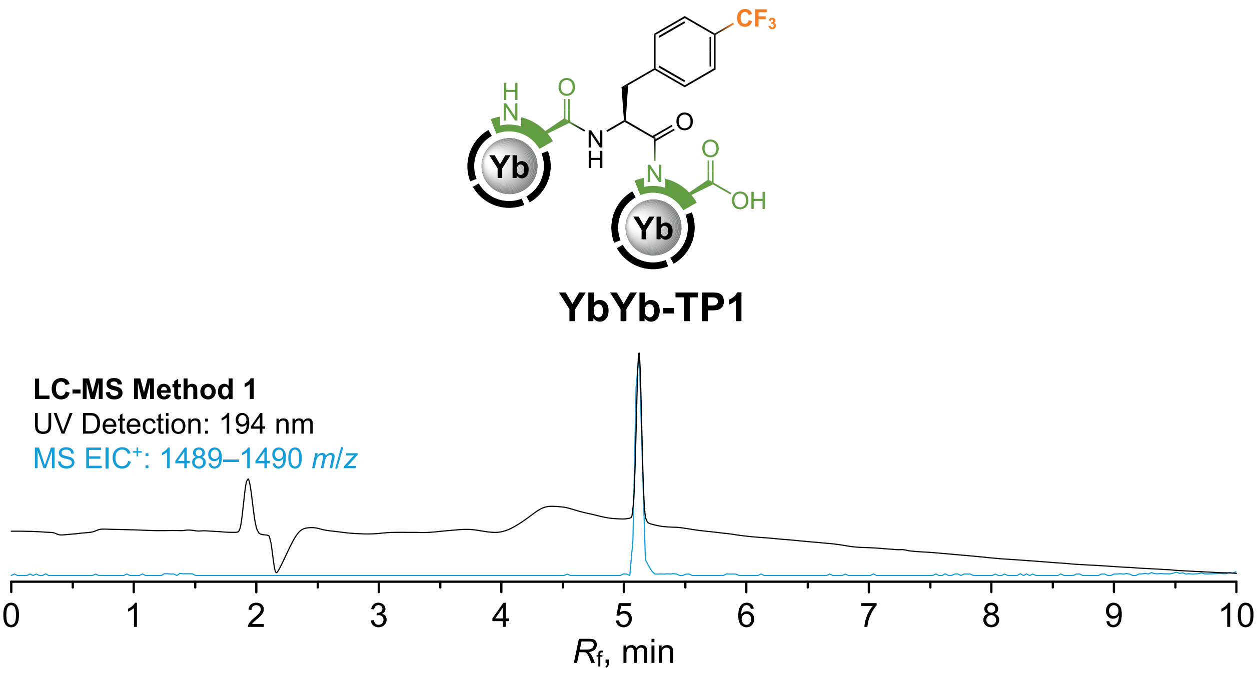

Supplementary Fig. 63. Synthesis and LC-MS chromatogram of YbYb-TP1. ***Synthesis:*** In a glass vial (4 mL), **Yb-TP1** (0.4 mg, ~0.3 µmol assuming **Dy-TP1**·2.5FA, 1.0 equiv.) was dissolved in aq. MOPS/NaOH buffer (500 mM, pH 7.0, 250 µL, 125 µmol, ~900 equiv.) followed by addition of aq. $YbCl_3$ (100 mM, 4 µL, 0.4 µmol, ~1.4 equiv.) and the resulting solution was stirred at RT for 15 mins. The mixture was then purified by preparative HPLC (C18, H_2O/MeCN gradient with 0.1% TFA additive). Fractions with product were joined and lyophilized to give product as white solid. **Yield:** ~0.4 mg. **NMR (aq. MOPS pH = 7.0, external D_2O):** ^{19}**F** (470.4 MHz, T = 298.2 K) δ_F –61.37 (CF_3, s). **ESI-HRMS:** 745.6728 $[M+2H]^{2+}$ (theor. $[C_{48}H_{68}O_{18}N_{11}F_3Yb_2]^{2+}$ = 745.6731).

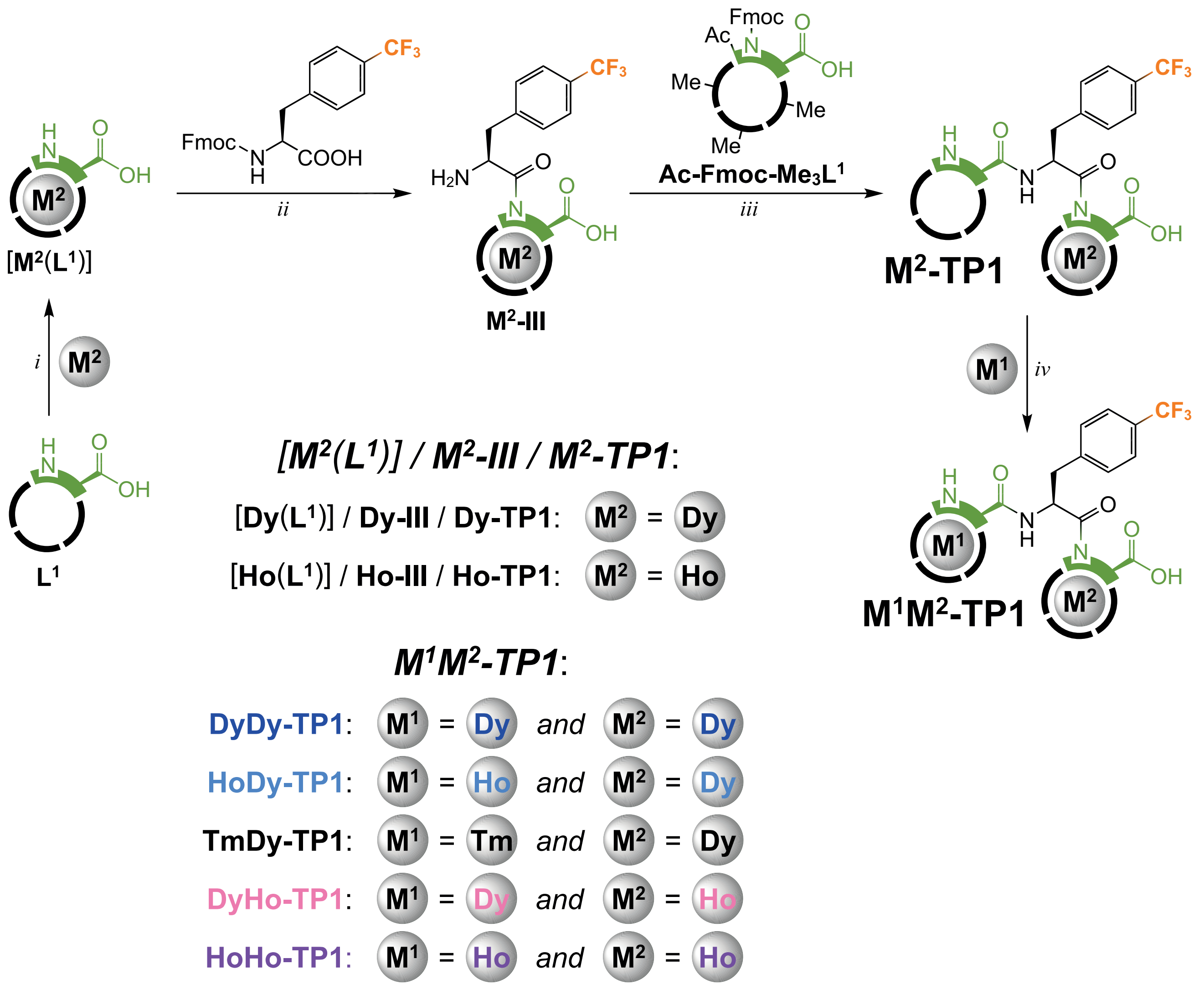

Supplementary Fig. 64. Alternative synthesis of M¹M²-TP1 tripeptides with Dy^{3+}, Ho^{3+} and Tm^{3+} cations. Charges were omitted for clarity reason. ***Conditions:*** (*i*) M^2Cl_3, aq. MOPS/NaOH buffer (pH 7.0); (*ii*) Fmoc-Phe{*p*-CF_3}-OH, PyAOP, DIPEA, DMSO *followed by* DBU, DMF; (*iii*) **Ac-Fmoc-Me₃L¹**, PyAOP, DIPEA, DMSO *followed by* LiOH, H_2O, MeOH; (*vi*) M^1Cl_3, aq. MOPS/NaOH buffer (pH 7.0).

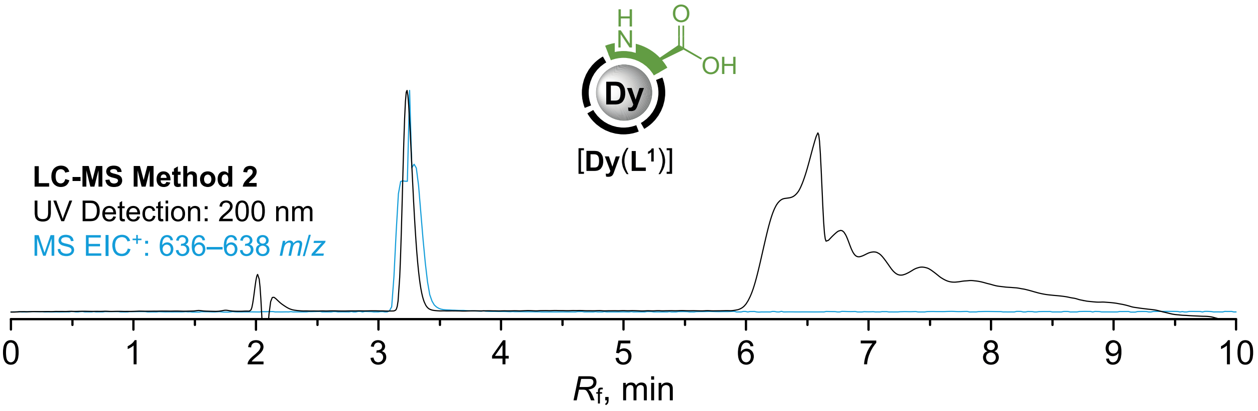

Supplementary Fig. 65. Synthesis and LC-MS chromatogram of building block [Dy(L[1])]. ***Synthesis:*** In a glass vial (4 mL), **L[1]**·2.4TFA·2.5H_2O (76.8 mg, 96.8 µmol, 1.0 equiv.) was dissolved in aq. MOPS/NaOH buffer (3.0 M, pH 7.0, 1.30 mL, 3.90 mmol, 40 equiv.) followed by addition of aq. $DyCl_3$ (100 mM, 1.03 mL, 103 µmol, 1.1 equiv.) and the resulting solution was stirred at RT for 15 mins. The mixture was then purified by preparative HPLC (C18, H_2O/MeCN gradient with 0.1% TFA additive). Fractions with product were joined and lyophilized to give product as white solid. **Yield:** 61.0 mg (76%; 1 step; based on **L[1]**·2.4TFA·2.5H_2O). **ESI-HRMS:** 637.1413 $[M+H]^+$ (theor. $[C_{19}H_{31}O_9N_5Dy_1]^+$ = 637.1408). **EA** ($C_{19}H_{30}N_5O_9Dy_1$·1.2TFA·3.0H_2O, M_R = 825.8): C 31.1 (31.3); H 4.5 (4.3); N 8.5 (8.4); F 8.3 (8.1); Dy 19.7 (19.3). ***Preparation of single crystals:*** In a glass vial (4 mL), aq. solution of [**Dy**(**L[1]**)] (repurified on HPLC with 0.1% FA additive – assuming zwitterionic form; ~20 mM, 62 µL, ~1.24 µmol, 1.0 equiv.; filtered through syringe microfilter) was mixed with aq. solution of HBr (~1.0 M, 1.24 µL, 1.24 µmol, ~1.0 equiv.) and the resulting solution was briefly vortexed. Then, *1,4*-dioxane (~160 µL) was slowly added dropwise until opalescence occured. The mixture was then sealed with a cap and gently heated using heatgun until clear solution was produced. The mixture was then left standing at RT for 3 weeks, producing single crystals of [**Dy**(**L[1]**)]·3.5H_2O suitable for X-Ray analysis. Simillar crystallization experiment with $HClO_4$ instead of HBr yielded identical crystals.

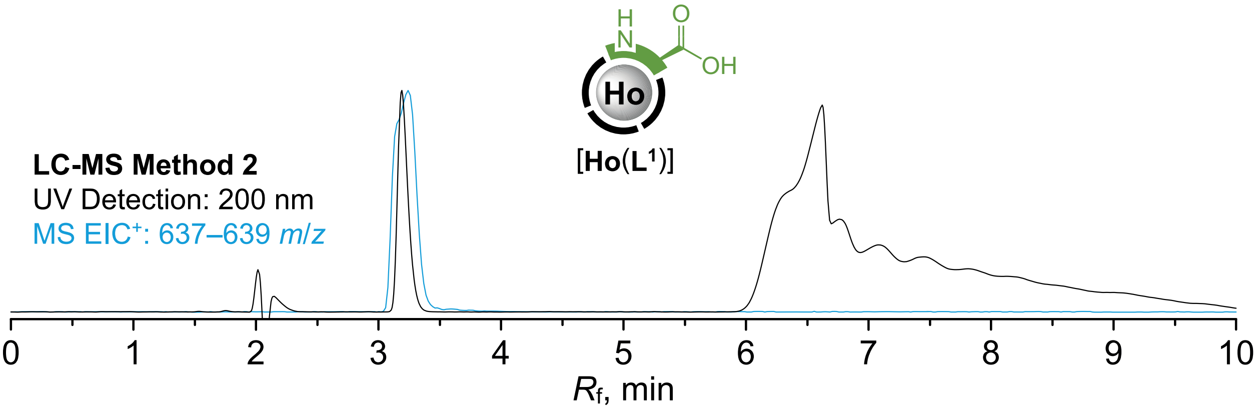

Supplementary Fig. 66. Synthesis and LC-MS chromatogram of building block [Ho(L¹)]. ***Synthesis:*** In a glass vial (4 mL), **L¹**·2.4TFA·2.5H_2O (76.8 mg, 96.8 µmol, 1.0 equiv.) was dissolved in aq. MOPS/NaOH buffer (3.0 M, pH 7.0, 1.30 mL, 3.90 mmol, 40 equiv.) followed by addition of aq. $HoCl_3$ (100 mM, 1.03 mL, 103 µmol, 1.1 equiv.) and the resulting solution was stirred at RT for 15 mins. The mixture was then purified by preparative HPLC (C18, H_2O/MeCN gradient with 0.1% TFA additive). Fractions with product were joined and lyophilized to give product as pinkish solid. **Yield:** 61.7 mg (78%; 1 step; based on **L¹**·2.4TFA·2.5H_2O). **ESI-HRMS:** 638.1424 $[M+H]^+$ (theor. $[C_{19}H_{31}O_9N_5Ho_1]^+$ = 638.1420). **EA** ($C_{19}H_{30}N_5O_9Ho_1$·1.1TFA·3.0H_2O, M_R = 816.8): C 31.2 (31.4); H 4.6 (4.3); N 8.6 (8.3); F 7.7 (7.8); Ho 20.2 (19.8).

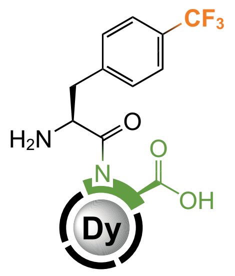

Dy-III

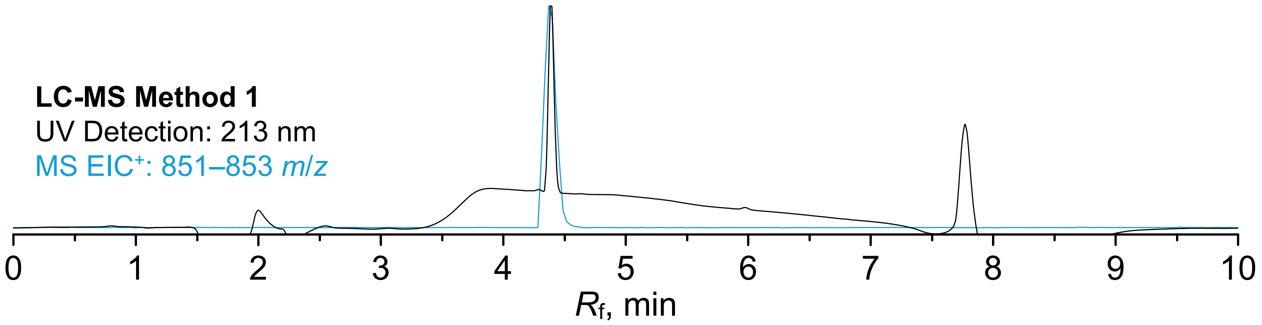

Supplementary Fig. 67. Synthesis and LC-MS chromatogram of intermediate Dy-III. ***Synthesis:*** In a glass vial (4 mL), [**Dy**(**L**[1])]·1.2TFA·3.0H_2O (12.8 mg, 15.5 µmol, 1.0 equiv.) was dissolved in dry DMSO (764 µL), followed by addition of Fmoc-Phe{*p*-CF_3}-OH (100 mM in dry DMSO, 389 µL, 38.9 µmol, 2.5 equiv.), PyAOP (100 mM in dry DMSO, 389 µL, 38.9 µmol, 2.5 equiv.) and DIPEA (13.5 µL, 77.1 µmol, 5.0 equiv.). The resulting solution was sonicated at RT for 2 mins and then further stirred at RT for 30 mins. The mixture was then purified by preparative HPLC (C18, H_2O/MeCN gradient with 0.1% FA additive). Fractions with Fmoc protected product were joined and lyophilized. The resulting white solid was dissolved in 2% DBU in DMF (891 µL, 119 µmol, 7.7 equiv.) and the solution was stirred at RT for 5 mins. Reaction was quenched with TFA (8 µL, 105 µmol, 6.8 equiv.) followed by addition of H_2O (100 µL). The mixture was then purified by preparative HPLC (C18, H_2O/MeCN gradient with 0.1% TFA additive). Fractions with product were joined and lyophilized to give product as white solid. **Yield:** 8.5 mg (51%; 2 steps; based on [**Dy**(**L**[1])]·1.2TFA·3.0H_2O). **ESI-HRMS:** 852.1981 $[M+H]^+$ (theor. $[C_{29}H_{39}O_{10}N_6F_3Dy_1]^+$ = 852.1966). **EA** ($C_{29}H_{38}N_6O_{10}F_3Dy_1$·1.1TFA·5.0$H_2O$, M_R = 1065.6): C 35.2 (34.8); H 4.6 (4.4); N 7.9 (7.7); F 11.2 (10.9).

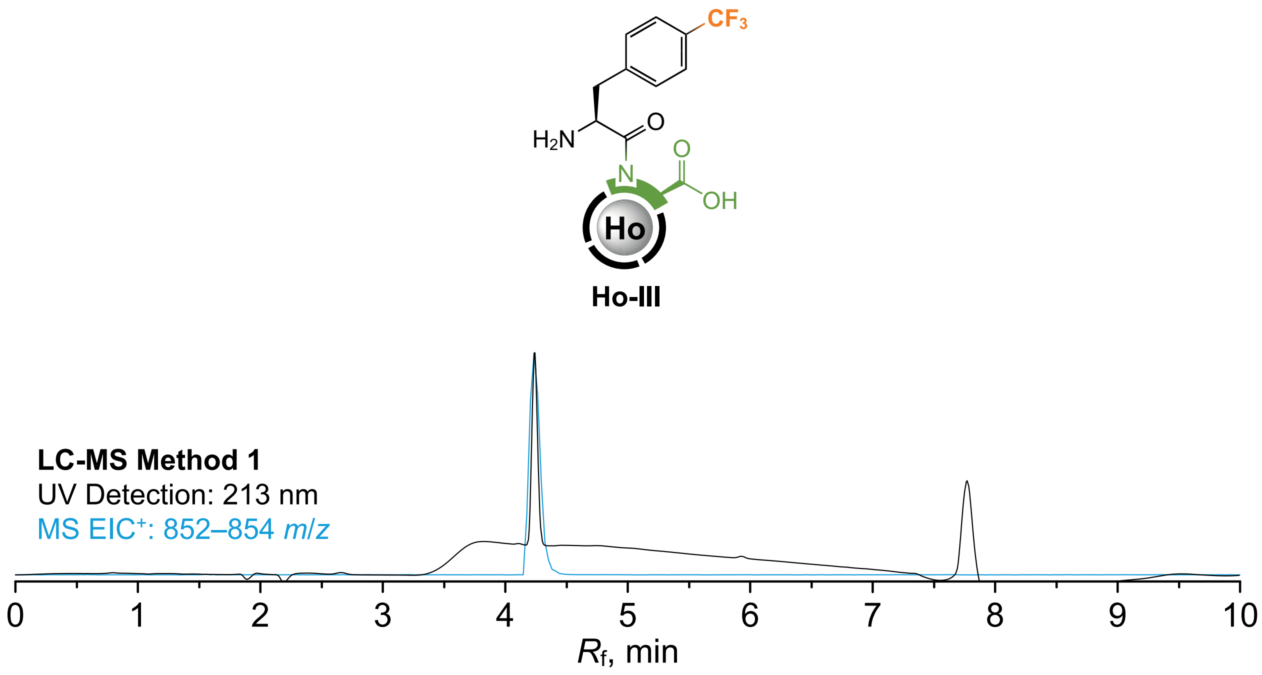

Supplementary Fig. 68. Synthesis and LC-MS chromatogram of intermediate Ho-III. ***Synthesis:*** In a glass vial (4 mL), [**Ho(L[1])**]·1.1TFA·3.0H_2O (13.8 mg, 16.9 µmol, 1.0 equiv.) was dissolved in dry DMSO (826 µL), followed by addition of Fmoc-Phe{*p*-CF_3}-OH (100 mM in dry DMSO, 420 µL, 42.0 µmol, 2.5 equiv.), PyAOP (100 mM in dry DMSO, 420 µL, 42.0 µmol, 2.5 equiv.) and DIPEA (14.6 µL, 84.0 µmol, 5.0 equiv.). The resulting solution was sonicated at RT for 2 mins and then further stirred at RT for 30 mins. The mixture was then purified by preparative HPLC (C18, H_2O/MeCN gradient with 0.1% FA additive). Fractions with Fmoc protected product were joined and lyophilized. The resulting pinkish solid was dissolved in 2% DBU in DMF (906 µL, 121 µmol, 7.2 equiv.) and the solution was stirred at RT for 5 mins. Reaction was quenched with TFA (9 µL, 118 µmol, 7.0 equiv.) followed by addition of H_2O (100 µL). The mixture was then purified by preparative HPLC (C18, H_2O/MeCN gradient with 0.1% TFA additive). Fractions with product were joined and lyophilized to give product as pinkish solid. **Yield:** 8.7 mg (49%; 2 steps; based on [**Ho(L[1])**]·1.1TFA·3.0H_2O assuming **Ho-III**·1.0TFA·5.0H_2O according to **Dy-IV** analogy, M_R = 1057). **ESI-HRMS:** 853.1985 $[M+H]^+$ (theor. $[C_{29}H_{39}O_{10}N_6F_3Ho_1]^+$ = 853.1978).

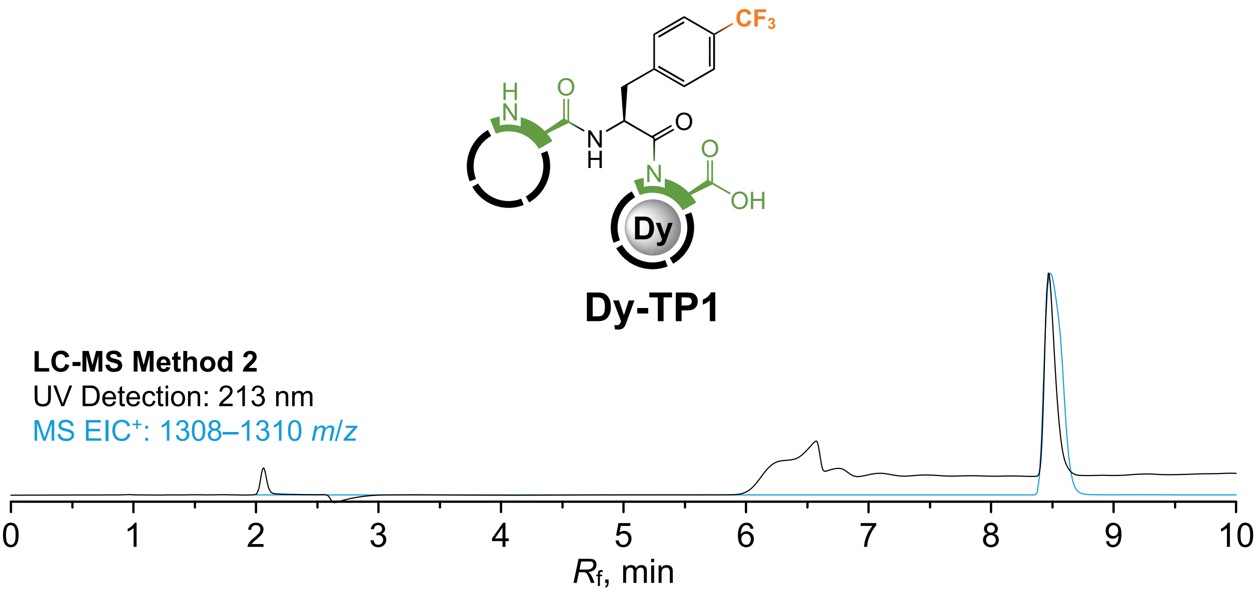

Supplementary Fig. 69. Synthesis and LC-MS chromatogram of intermediate Dy-TP1. ***Synthesis:*** In a glass vial (4 mL), **Dy-III**·1.1TFA·5.0H_2O (25.4 mg, 23.8 µmol, 1.0 equiv.) was dissolved in dry DMSO (1.28 mL), followed by addition of **Ac-Fmoc-Me$_3$L^1**·1.9TFA·1.3H_2O (100 mM in dry DMSO, 238 µL, 23.8 µmol, 1.0 equiv.), PyAOP (100 mM in dry DMSO, 596 µL, 59.6 µmol, 2.5 equiv.) and DIPEA (29.0 µL, 166 µmol, 7.0 equiv.). The resulting solution was stirred at RT for 30 mins. The mixture was then purified by preparative HPLC (C18, H_2O/MeCN gradient with 0.1% FA additive). Fractions with protected product were joined and lyophilized. The resulting white solid was dissolved in a mixture of MeOH (3.68 mL) and H_2O (0.41 mL) followed by addition of aq. LiOH (100 mM, 364 µL, 364 µmol, 15 equiv.) and the mixture was stirred at RT for 43 h. Reaction was then quenched by FA (12.5 µL, 331 µmol, 14 equiv.) and the mixture was evaporated to dryness. The residue was purified by preparative HPLC (C18, H_2O/MeCN gradient with 0.1% FA additive). Fractions with product were joined and lyophilized to give product as white solid. **Yield:** 15.5 mg (46%; 2 steps; based on **Dy-III**·1.1TFA·5.0H_2O assuming **Dy-TP1**·2.5FA, M_R = 1423). **ESI-HRMS:** 1309.4130 $[M+H]^+$ (theor. $[C_{48}H_{70}O_{18}N_{11}F_3Dy_1]^+$ = 1309.4139).

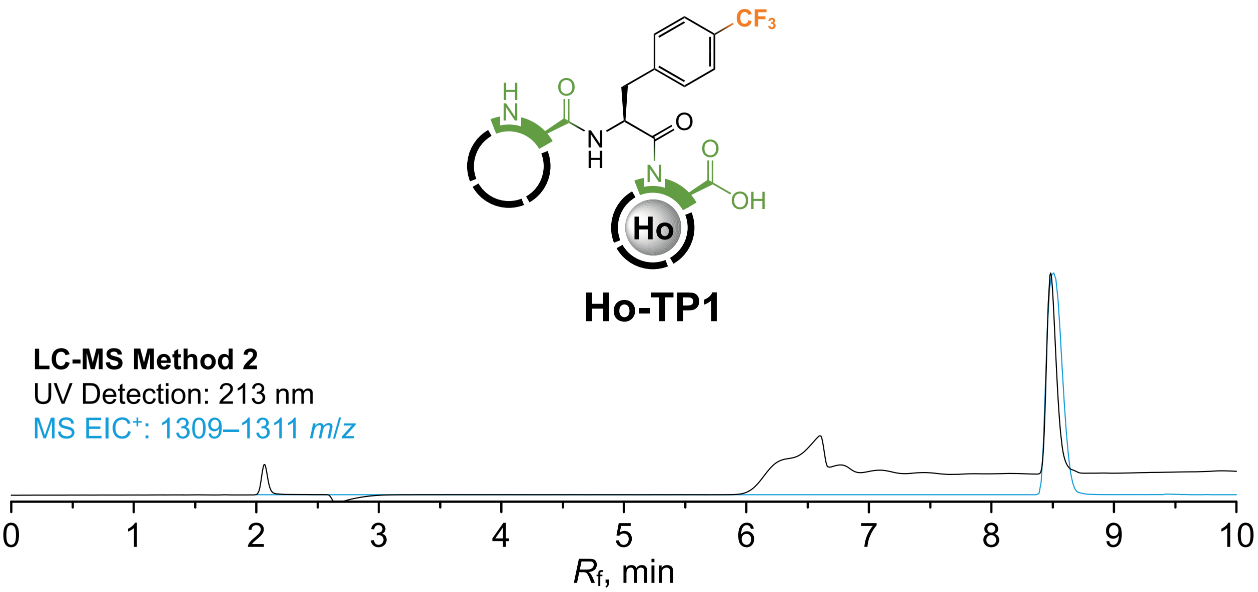

Supplementary Fig. 70. Synthesis and LC-MS chromatogram of intermediate Ho-TP1. ***Synthesis:*** In a glass vial (4 mL), **Ho-III** (26.3 mg, 24.9 µmol assuming **Ho-III**·1.0TFA·5.0H_2O, 1.0 equiv.) was dissolved in dry DMSO (1.33 mL), followed by addition of **Ac-Fmoc-Me$_3$L^1**·1.9TFA·1.3H_2O (100 mM in dry DMSO, 247 µL, 24.7 µmol, 1.0 equiv.), PyAOP (100 mM in dry DMSO, 617 µL, 61.7 µmol, 2.5 equiv.) and DIPEA (30.0 µL, 171 µmol, 6.9 equiv.). The resulting solution was stirred at RT for 30 mins. The mixture was then purified by preparative HPLC (C18, H_2O/MeCN gradient with 0.1% FA additive). Fractions with protected product were joined and lyophilized. The resulting pinkish solid was dissolved in a mixture of MeOH (4.11 mL) and H_2O (0.46 mL) followed by addition of aq. LiOH (100 mM, 373 µL, 373 µmol, 15 equiv.) and the mixture was stirred at RT for 43 h. Reaction was then quenched by FA (14.1 µL, 374 µmol, 15 equiv.) and the mixture was evaporated to dryness. The residue was purified by preparative HPLC (C18, H_2O/MeCN gradient with 0.1% FA additive). Fractions with product were joined and lyophilized to give product as pinkish solid. **Yield:** 19.1 mg (54%; 2 steps; based on **Ho-III**·1.0TFA·5.0H_2O assuming **Ho-TP1**·2.5FA, M_R = 1425). **ESI-HRMS:** 1310.4146 $[M+H]^+$ (theor. $[C_{48}H_{70}O_{18}N_{11}F_3Ho_1]^+$ = 1310.4150).

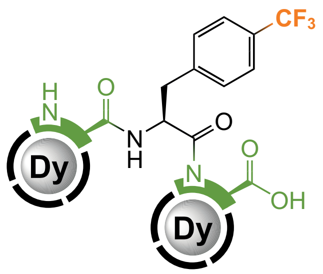

DyDy-TP1

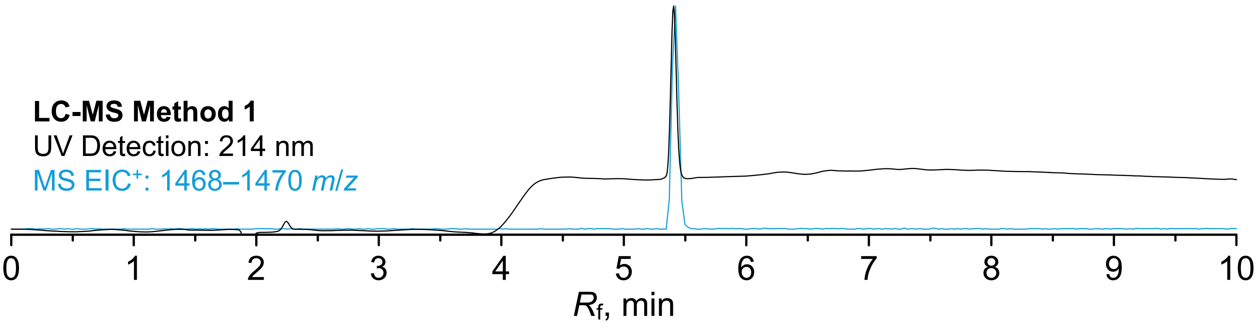

Supplementary Fig. 71. Synthesis and LC-MS chromatogram of DyDy-TP1. ***Synthesis:*** In a glass vial (4 mL), **Dy-TP1** (7.0 mg, 4.9 µmol assuming **Dy-TP1**·2.5FA, 1.0 equiv.) was dissolved in aq. MOPS/NaOH buffer (500 mM, pH 7.0, 2.0 mL, 1.0 mmol, 200 equiv.) followed by addition of aq. $DyCl_3$ (100 mM, 59 µL, 5.9 µmol, 1.2 equiv.) and the resulting solution was stirred at RT for 15 mins. The mixture was then purified by preparative HPLC (C18, H_2O/MeCN gradient with 0.1% TFA additive). Fractions with product were joined and lyophilized to give product as white solid. **Yield:** 7.3 mg (94%; 1 step; based on **Dy-TP1**·2.5FA assuming **DyDy-TP1**·1.0TFA, M_R = 1581). **NMR (aq. MOPS pH = 7.0, external D_2O):** ^{19}**F** (470.4 MHz, T = 298.2 K) δ_F –57.33 (CF_3, s). **ESI-HRMS:** 735.6657 $[M+2H]^{2+}$ (theor. $[C_{48}H_{68}O_{18}N_{11}F_3Dy_2]^{2+}$ = 735.6634).

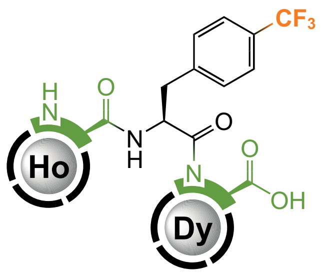

HoDy-TP1

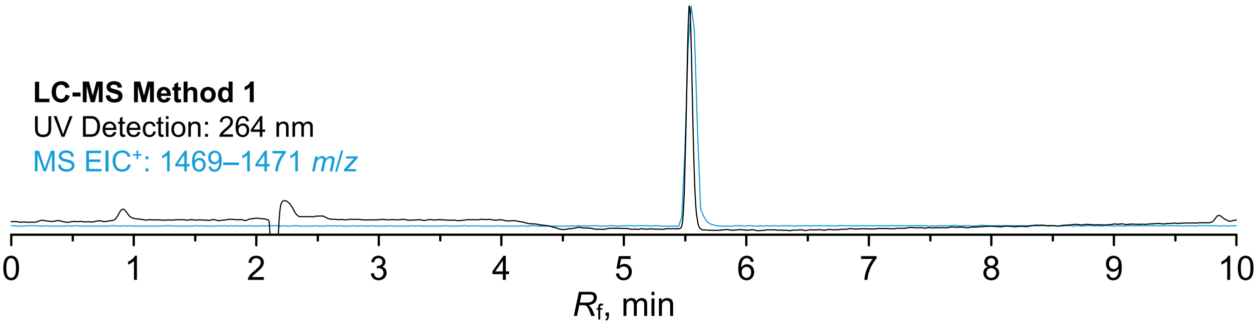

Supplementary Fig. 72. Synthesis and LC-MS chromatogram of HoDy-TP1. ***Synthesis:*** In a glass vial (4 mL), **Dy-TP1** (7.0 mg, 4.9 µmol assuming **Dy-TP1**·2.5FA, 1.0 equiv.) was dissolved in aq. MOPS/NaOH buffer (500 mM, pH 7.0, 2.0 mL, 1.0 mmol, 200 equiv.) followed by addition of aq. $HoCl_3$ (100 mM, 59 µL, 5.9 µmol, 1.2 equiv.) and the resulting solution was stirred at RT for 15 mins. The mixture was then purified by preparative HPLC (C18, H_2O/MeCN gradient with 0.1% TFA additive). Fractions with product were joined and lyophilized to give product as pinkish solid. **Yield:** 7.4 mg (95%; 1 step; based on **Dy-TP1**·2.5FA assuming **HoDy-TP1**·1.0TFA, M_R = 1584). **NMR (aq. MOPS pH = 7.0, external D_2O):** ^{19}**F** (470.4 MHz, T = 298.2 K) δ_F –54.75 (CF_3, s). **ESI-HRMS:** 736.1659 $[M+2H]^{2+}$ (theor. $[C_{48}H_{68}O_{18}N_{11}F_3Dy_1Ho_1]^{2+}$ = 736.1640).

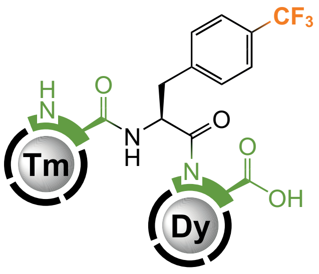

TmDy-TP1

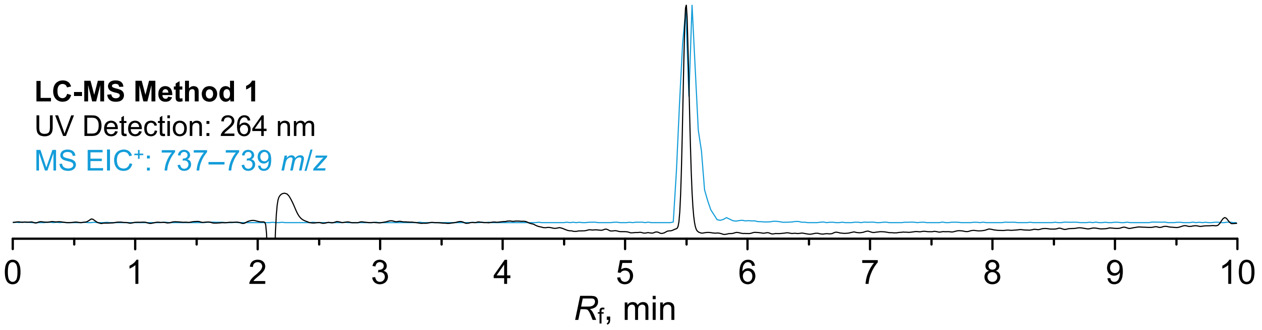

Supplementary Fig. 73. Synthesis and LC-MS chromatogram of TmDy-TP1. ***Synthesis:*** In a glass vial (2 mL), **Dy-TP1** (0.8 mg, 0.56 µmol assuming **Dy-TP1**·2.5FA, 1.0 equiv.) was dissolved in aq. MOPS/NaOH buffer (500 mM, pH 7.0, 100 µL, 50 µmol, 90 equiv.)followed by addition of aq. $TmCl_3$ (100 mM, 12 µL, 1.2 µmol, 2.1 equiv.) and the resulting solution was stirred at RT for 15 mins. The mixture was then purified by preparative HPLC (C18, H_2O/MeCN gradient with 0.1% TFA additive). Fractions with product were joined and lyophilized to give product as white solid. **Yield:** 0.7 mg (79%; 1 step; based on **Dy-TP1**·2.5FA assuming **TmDy-TP1**·1.0TFA, M_R = 1588). **NMR (aq. MOPS pH = 7.0, external D_2O):** ^{19}**F** (470.4 MHz, T = 298.2 K) δ_F –38.16 (CF_3, s). **ESI-HRMS:** 738.1666 $[M+2H]^{2+}$ (theor. $[C_{48}H_{68}O_{18}N_{11}F_3Dy_1Tm_1]^{2+}$ = 738.1659).

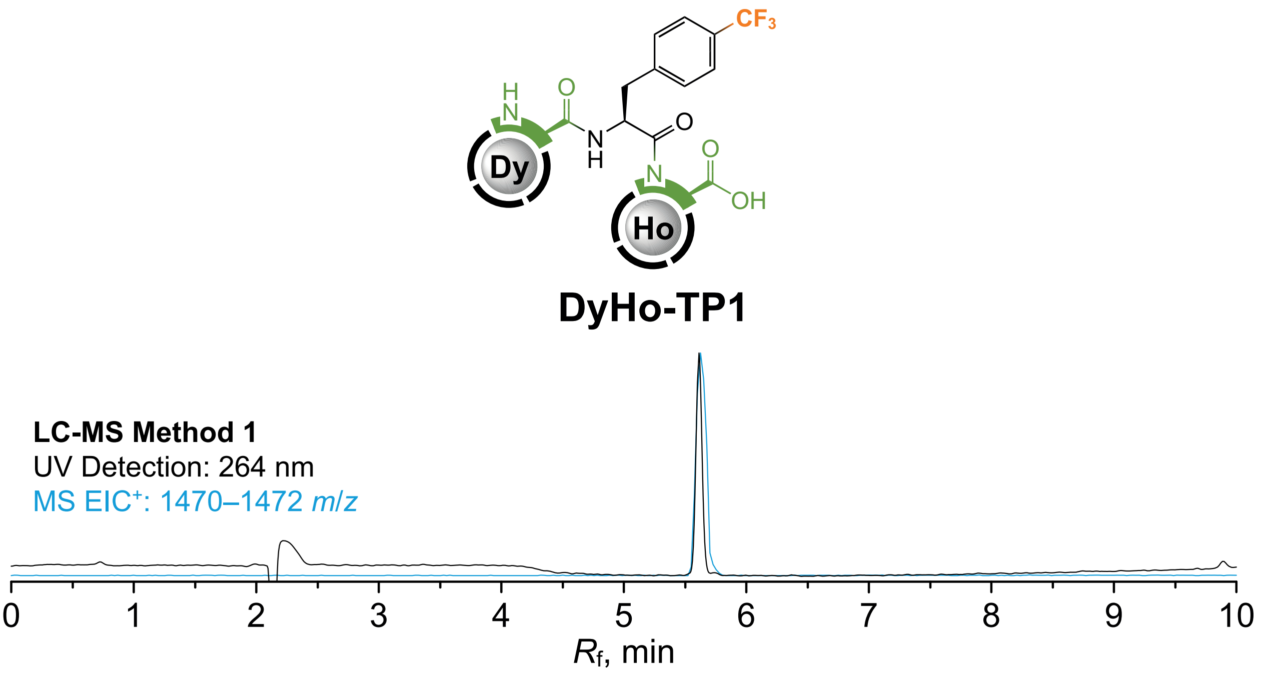

Supplementary Fig. 74. Synthesis and LC-MS chromatogram of DyHo-TP1. ***Synthesis:*** In a glass vial (4 mL), **Ho-TP1** (9.0 mg, 6.3 µmol assuming **Ho-TP1**·2.5FA, 1.0 equiv.) was dissolved in aq. MOPS/NaOH buffer (500 mM, pH 7.0, 2.0 mL, 1.0 mmol, 160 equiv.) followed by addition of aq. $DyCl_3$ (100 mM, 76 µL, 7.6 µmol, 1.2 equiv.) and the resulting solution was stirred at RT for 15 mins. The mixture was then purified by preparative HPLC (C18, H_2O/MeCN gradient with 0.1% TFA additive). Fractions with product were joined and lyophilized to give product as pinkish solid. **Yield:** 8.1 mg (81%; 1 step; based on **Ho-TP1**·2.5FA assuming **HoHo-TP1**·1.0TFA, M_R = 1584). **NMR (aq. MOPS pH = 7.0, external D_2O):** ^{19}**F** (470.4 MHz, T = 298.2 K) δ_F –62.75 (CF_3, s). **ESI-HRMS:** 736.1651 $[M+2H]^{2+}$ (theor. $[C_{48}H_{68}O_{18}N_{11}F_3Ho_1Dy_1]^{2+}$ =736.1640).

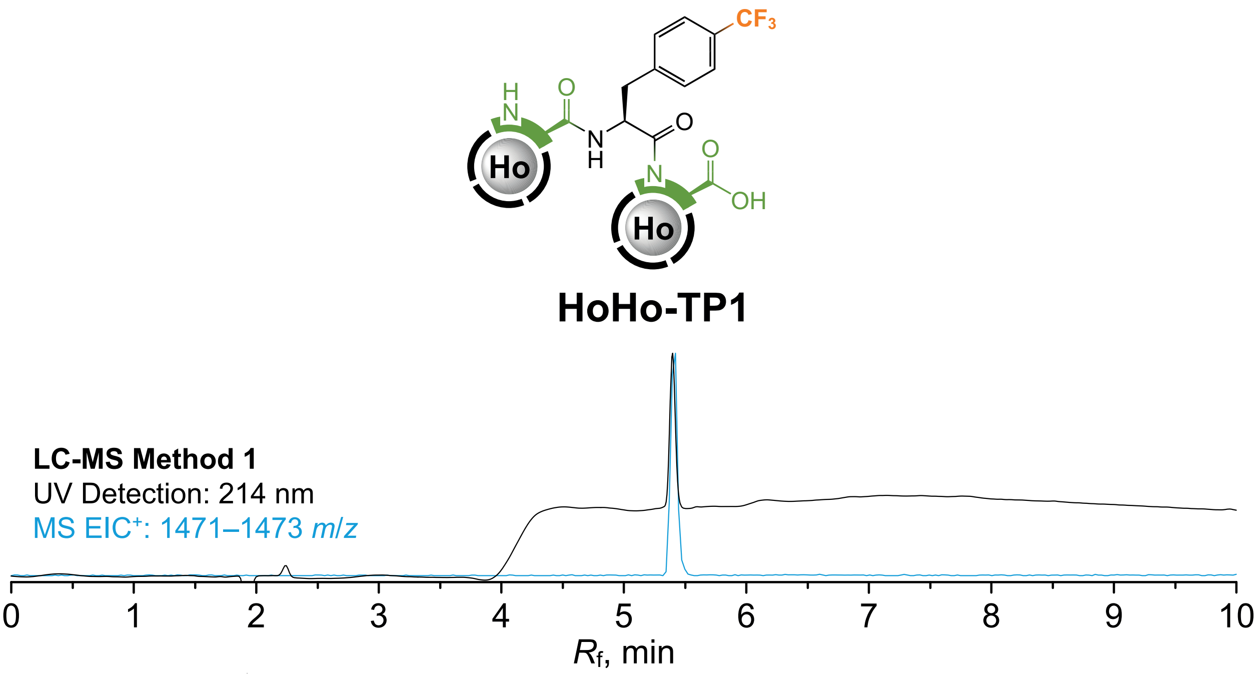

Supplementary Fig. 75. Synthesis and LC-MS chromatogram of HoHo-TP1. ***Synthesis:*** In a glass vial (4 mL), **Ho-TP1** (9.0 mg, 6.3 µmol assuming **Ho-TP1**·2.5FA, 1.0 equiv.) was dissolved in aq. MOPS/NaOH buffer (500 mM, pH 7.0, 2.0 mL, 1.0 mmol, 160 equiv.) followed by addition of aq. $HoCl_3$ (100 mM, 76 µL, 7.6 µmol, 1.2 equiv.) and the resulting solution was stirred at RT for 15 mins. The mixture was then purified by preparative HPLC (C18, H_2O/MeCN gradient with 0.1% TFA additive). Fractions with product were joined and lyophilized to give product as pinkish solid. **Yield:** 8.7 mg (87%; 1 step; based on **Ho-TP1**·2.5FA assuming **HoHo-TP1**·1.0TFA, M_R = 1586). **NMR (aq. MOPS pH = 7.0, external D_2O):** ^{19}**F** (470.4 MHz, T = 298.2 K) δ_F –60.19 (CF_3, s). **ESI-HRMS:** 1472.3229 $[M+H]^+$ (theor. $[C_{48}H_{67}O_{18}N_{11}F_3Ho_2]^+$ =1472.3219).

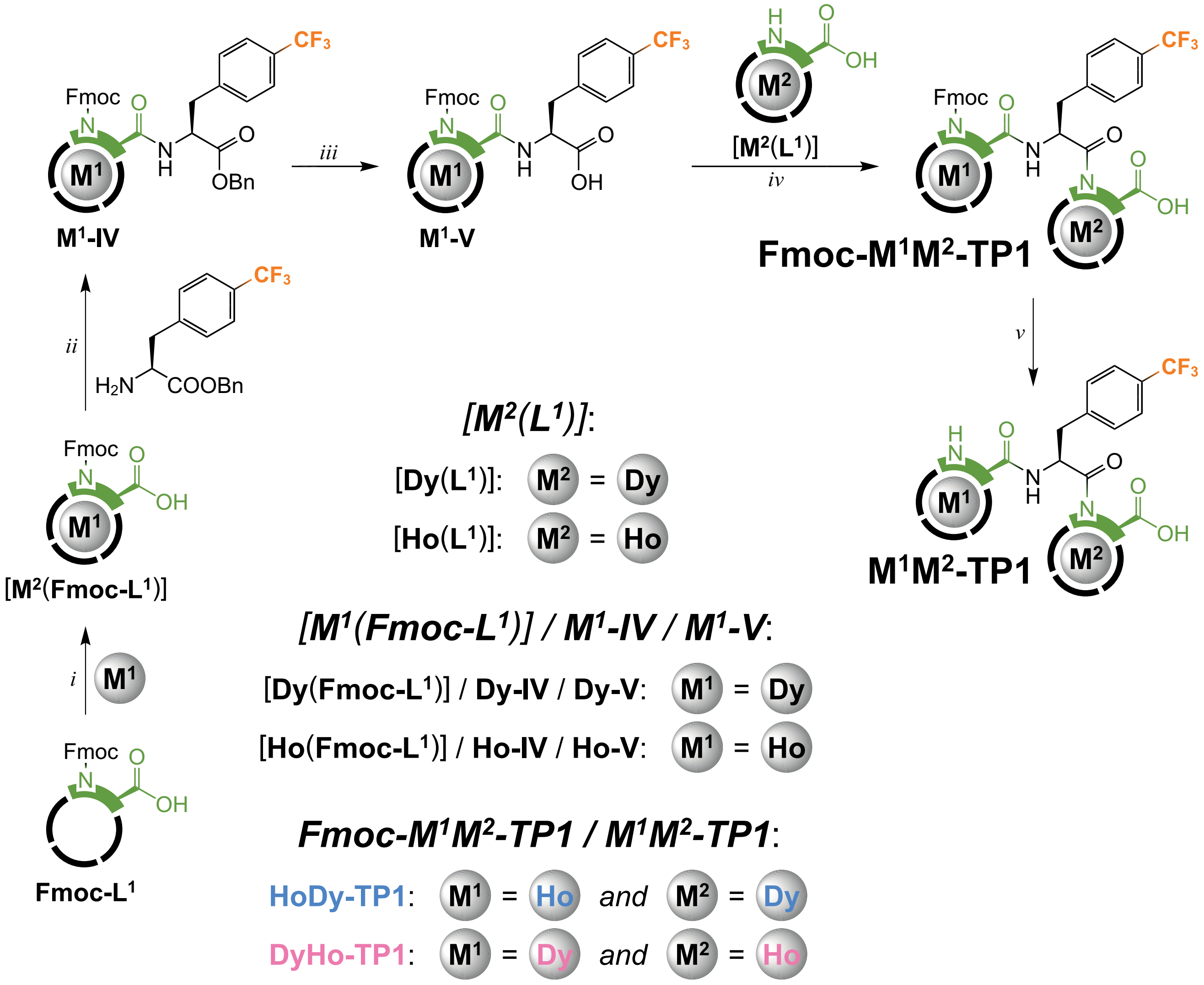

Supplementary Fig. 76. Direct synthesis of DyHo-TP1 and HoDy-TP1. ***Conditions:*** (*i*) M^1Cl_3, aq. MOPS/NaOH (pH 7.0); (*ii*) H-Phe{*p*-CF_3}-OBn, PyAOP, DIPEA, DMSO, 80 °C; (*iii*) H_2, Pd@C, MeOH; (*iv*) [**M²(L¹)**], PyAOP, DIPEA, DMSO; (*v*) DBU, DMF.

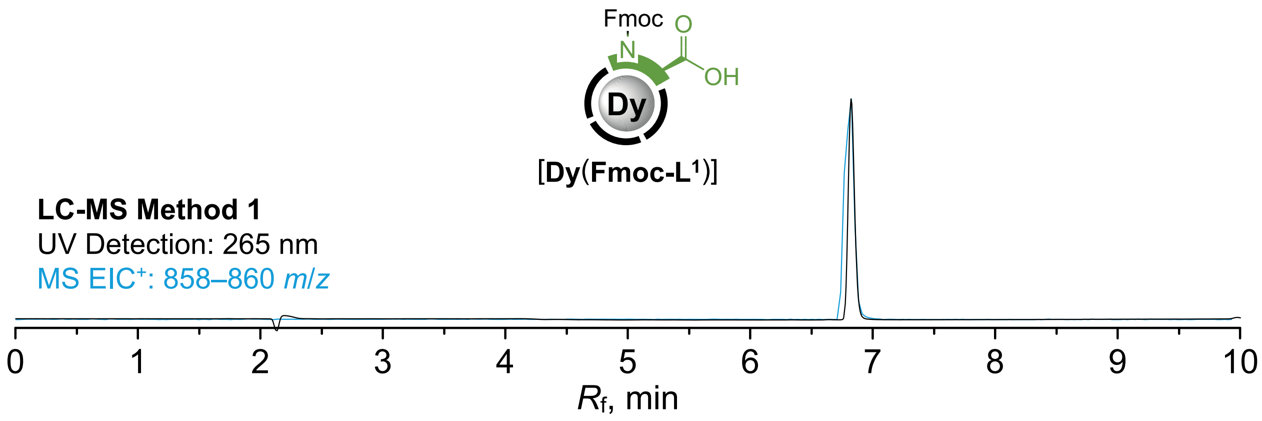

Supplementary Fig. 77. Synthesis and LC-MS chromatogram of building block [Dy(Fmoc-L[1]]. ***Synthesis:*** In a glass vial (4 mL), **Fmoc-L[1]**·2.0TFA·1.3FA (58.0 mg, 58.9 µmol, 1.0 equiv.) was dissolved in H_2O (1 mL) followed by addition of aq. MOPS/NaOH buffer (3.0 M, pH 7.0, 787 µL, 2.36 mmol, 40 equiv.) followed by addition of aq. $DyCl_3$ (100 mM, 700 µL, 70 µmol, 1.2 equiv.) and the resulting solution was stirred at RT for 15 mins. The mixture was then purified by preparative HPLC (C18, H_2O/MeCN gradient with 0.1% TFA additive). Fractions with product were joined and lyophilized to give product as white solid. **Yield:** 42.0 mg (68%; 1 step; based on **Fmoc-L[1]**·2.0TFA·1.3FA). **ESI-HRMS:** 881.1928 $[M+Na]^+$ (theor. $[C_{34}H_{40}O_{11}N_5Dy_1Na_1]^+$ = 881.1908). **EA** ($C_{34}H_{40}N_5O_{11}Dy_1$·1.1TFA·3.5$H_2O$, M_R = 1045.7): C 41.6 (41.9); H 4.6 (4.4); N 6.7 (6.4); F 6.0 (6.0); Dy 15.5 (12.5).

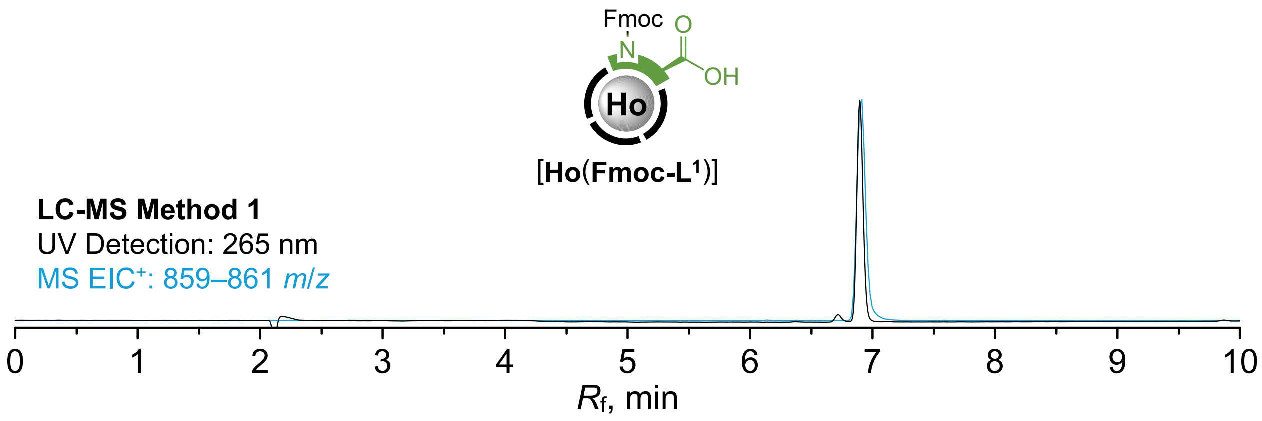

Supplementary Fig. 78. Synthesis and LC-MS chromatogram of building block [Ho(Fmoc-L^1]. ***Synthesis:*** In a glass vial (4 mL), **Fmoc-L^1**·2.0TFA·1.3FA (58.0 mg, 58.9 µmol, 1.0 equiv.) was dissolved in H_2O (1 mL) followed by addition of aq. MOPS/NaOH buffer (3.0 M, pH 7.0, 787 µL, 2.36 mmol, 40 equiv.) followed by addition of aq. $HoCl_3$ (100 mM, 700 µL, 70 µmol, 1.2 equiv.) and the resulting solution was stirred at RT for 15 mins. The mixture was then purified by preparative HPLC (C18, H_2O/MeCN gradient with 0.1% TFA additive). Fractions with product were joined and lyophilized to give product as pinkish solid. **Yield:** 38.5 mg (62%; 1 step; based on **Fmoc-L^1**·2.0TFA·1.3FA). **ESI-HRMS:** 860.2111 $[M+H]^+$ (theor. $[C_{34}H_{41}O_{11}N_5Ho_1]^+$ = 860.2100). **EA** ($C_{34}H_{40}N_5O_{11}Ho_1$·1.1TFA·3.5$H_2O$, M_R = 1048.1): C 41.5 (41.9); H 4.6 (4.5); N 6.7 (6.2); F 6.0 (5.8); Dy 15.7 (13.5).

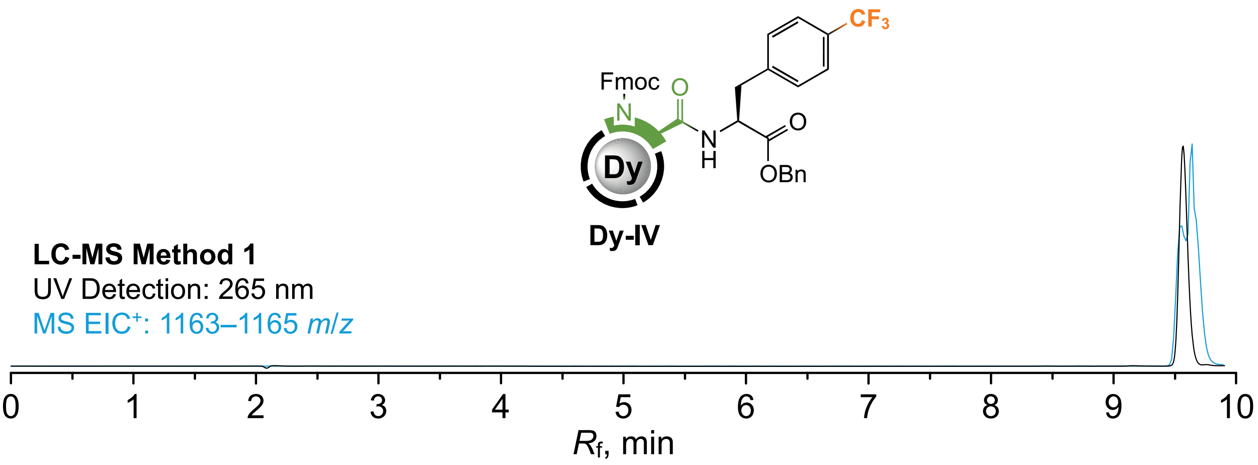

Supplementary Fig. 79. Synthesis and LC-MS chromatogram of intermediate Dy-IV. ***Synthesis:*** In a glass vial (4 mL), [**Dy**(**Fmoc-L**[1])]·1.1TFA·3.5H_2O (15.5 mg, 14.8 µmol, 1.0 equiv.) was dissolved in dry DMSO (460 µL), followed by addition of H-Phe{*p*-CF_3}-OBn (100 mM in dry DMSO, 204 µL, 20.4 µmol, 1.4 equiv.), PyAOP (100 mM in dry DMSO, 850 µL, 85.0 µmol, 5.7 equiv.) and DIPEA (14.6 µL, 84.0 µmol, 5.7 equiv.). The resulting solution was stirred at 80 °C for 5 mins. The mixture was then purified by preparative HPLC (C18, H_2O/MeCN gradient with 0.1% FA additive). Fractions with product were joined and lyophilized to give product as white solid. **Yield:** 13.7 mg (76%; 1 step; based on [**Dy**(**Fmoc-L**[1])]·1.1TFA·3.5H_2O assuming **Dy-IV**·1.0FA, M_R = 1209). **ESI-HRMS:** 1164.3113 $[M+H]^+$ (theor. $[C_{51}H_{55}O_{12}N_6F_3Dy_1]^+$ = 1164.3116).

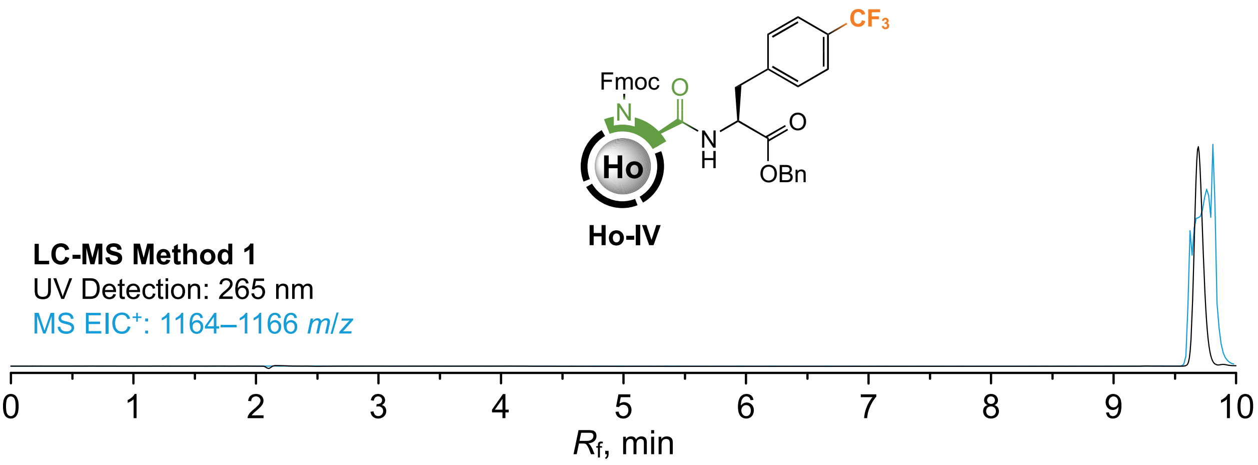

Supplementary Fig. 80. Synthesis and LC-MS chromatogram of intermediate Ho-IV. ***Synthesis:*** In a glass vial (4 mL), [**Ho**(**Fmoc-L**[1])]·1.1TFA·3.5H_2O (15.5 mg, 14.8 µmol, 1.0 equiv.) was dissolved in dry DMSO (460 µL), followed by addition of H-Phe{*p*-CF_3}-OBn (100 mM in dry DMSO, 204 µL, 20.4 µmol, 1.4 equiv.), PyAOP (100 mM in dry DMSO, 850 µL, 85.0 µmol, 5.7 equiv.) and DIPEA (14.6 µL, 84.0 µmol, 5.7 equiv.). The resulting solution was stirred at 80 °C for 5 mins. The mixture was then purified by preparative HPLC (C18, H_2O/MeCN gradient with 0.1% FA additive). Fractions with product were joined and lyophilized to give product as pinkish solid. **Yield:** 10.5 mg (59%; 1 step; based on [**Ho**(**Fmoc-L**[1])]·1.1TFA·3.5H_2O assuming **Ho-IV**·1.0FA, M_R = 1211). **ESI-HRMS:** 1165.3122 $[M+H]^+$ (theor. $[C_{51}H_{55}O_{12}N_6F_3Ho_1]^+$ = 1165.3128).

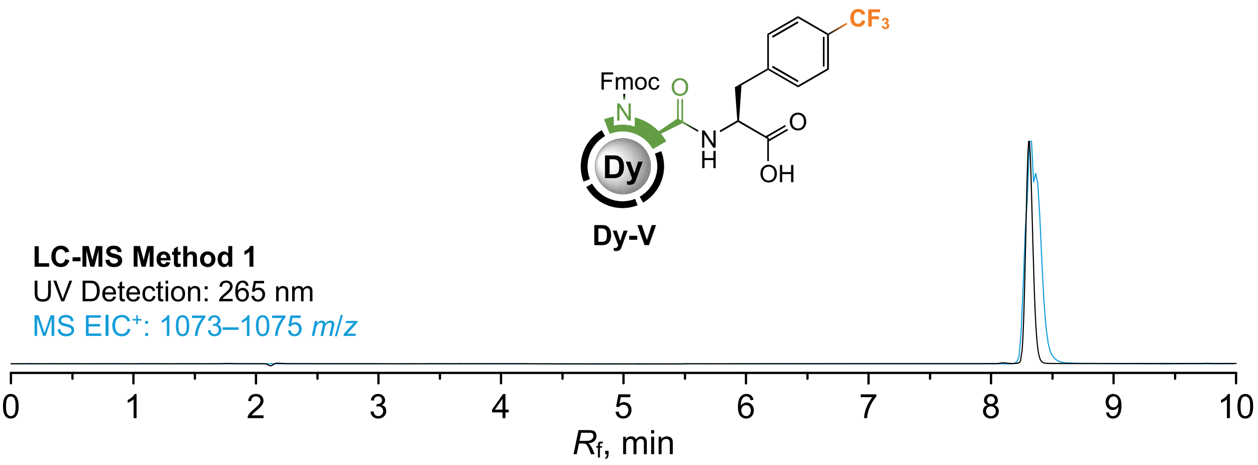

Supplementary Fig. 81. Synthesis and LC-MS chromatogram of intermediate Dy-V. ***Synthesis:*** In a pear-shaped glass flask (25 mL), Pd@C (10%, 5 mg) was three-times secured with Ar followed by addition of solution of intermediate **Dy-IV** (12.4 mg, 10.3 µmol assuming **Dy-IV**·1.0FA, 1.0 equiv.) in MeOH (9 mL) through septum. The mixture was then stirred at RT for 30 min with slow bubling of H_2 (from ballon) through the mixture. Reaction mixture was filtered through syringe microfilter (PTFE) and the filtrate was evaporated to dryness. Residue was purified by preparative HPLC (C18, H_2O/MeCN gradient with 0.1% TFA additive). Fractions with product were joined and lyophilized to give product as white solid. **Yield:** 7.0 mg (58%; 1 step; based on **Dy-IV**·1.0FA assuming **Dy-V**·1.0TFA, M_R = 1186). **ESI-HRMS:** 1074.2655 $[M+H]^+$ (theor. $[C_{44}H_{49}O_{12}N_6F_3Dy_1]^+$ = 1074.2647).

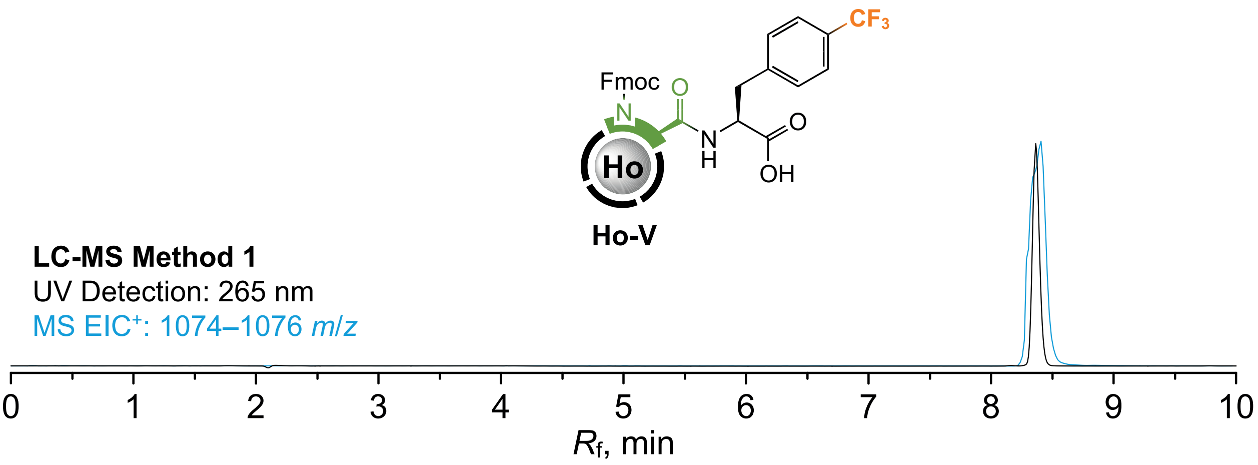

Supplementary Fig. 82. Synthesis and LC-MS chromatogram of intermediate Ho-V. ***Synthesis:*** In a pear-shaped glass flask (25 mL), Pd@C (10%, 3 mg) was three-times secured with Ar followed by addition of solution of intermediate **Ho-IV** (10.3 mg, 8.5 µmol assuming **Ho-IV**·1.0FA, 1.0 equiv.) in MeOH (5 mL) through septum. The mixture was then stirred at RT for 30 min with slow bubling of H_2 (from ballon) through the mixture. Reaction mixture was filtered through syringe microfilter (PTFE) and the filtrate was evaporated to dryness. Residue was purified by preparative HPLC (C18, H_2O/MeCN gradient with 0.1% TFA additive). Fractions with product were joined and lyophilized to give product as pinkish solid. **Yield:** 7.6 mg (75%; 1 step; based on **Ho-IV**·1.0FA assuming **Ho-V**·1.0TFA, M_R = 1189). **ESI-HRMS:** 1075.2663 $[M+H]^+$ (theor. $[C_{44}H_{49}O_{12}N_6F_3Ho_1]^+$ = 1075.2658).

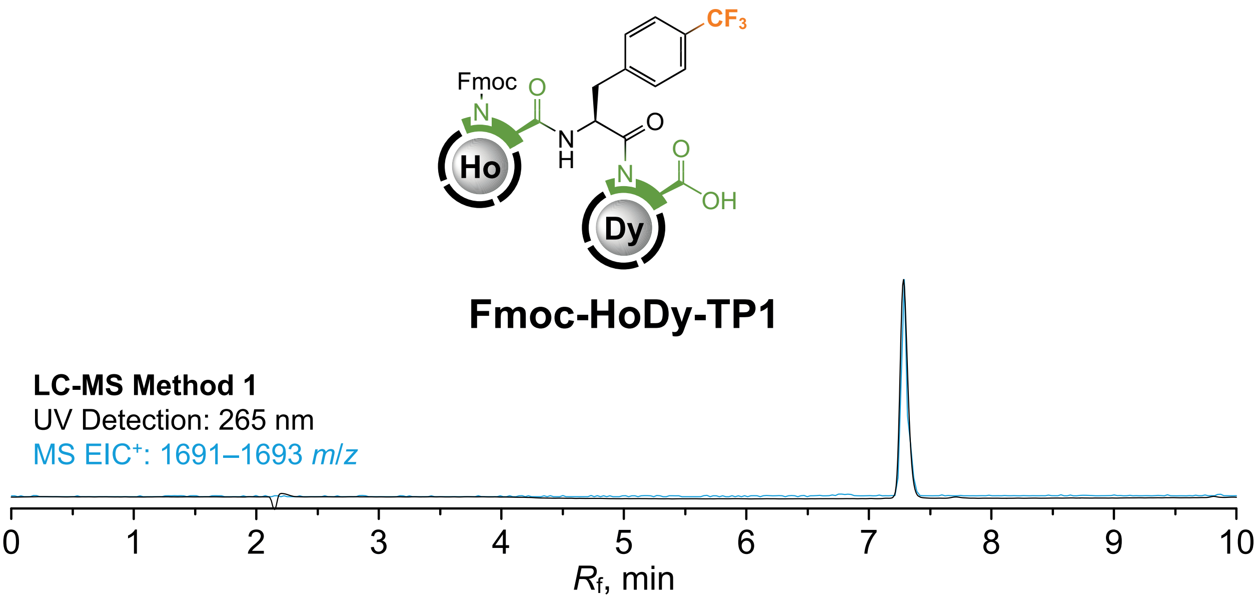

Supplementary Fig. 83. Synthesis and LC-MS chromatogram of intermediate Fmoc-HoDy-TP1. ***Synthesis:*** In a glass vial (4 mL), **Ho-V** (7.6 mg, 6.4 µmol assuming **Ho-V**·1.0TFA, 1.0 equiv.) and [**Dy**(**L**[1])]·1.2TFA·3.0H_2O (6.3 mg, 7.6 µmol, 1.2 equiv.) were dissolved in dry DMSO (550 µL) followed by addition of PyAOP (100 mM in dry DMSO, 64 µL, 6.4 µmol, 1.0 equiv.) and DIPEA (5.7 µL, 32.5 µmol, 5 equiv.). The resulting solution was stirred at RT for 15 mins. The mixture was then purified by preparative HPLC (C18, H_2O/MeCN gradient with 0.1% FA additive). Fractions with product were joined and lyophilized to give product as pinkish solid. **Yield:** 3.6 mg (33%; 1 step; based on **Ho-V**·1.0TFA assuming **Fmoc-HoDy-TP1**, M_R = 1692). **ESI-HRMS:** 847.1983 $[M+2H]^{2+}$ (theor. $[C_{63}H_{78}O_{20}N_{11}F_3Ho_1Dy_1]^{2+}$ = 847.1980).

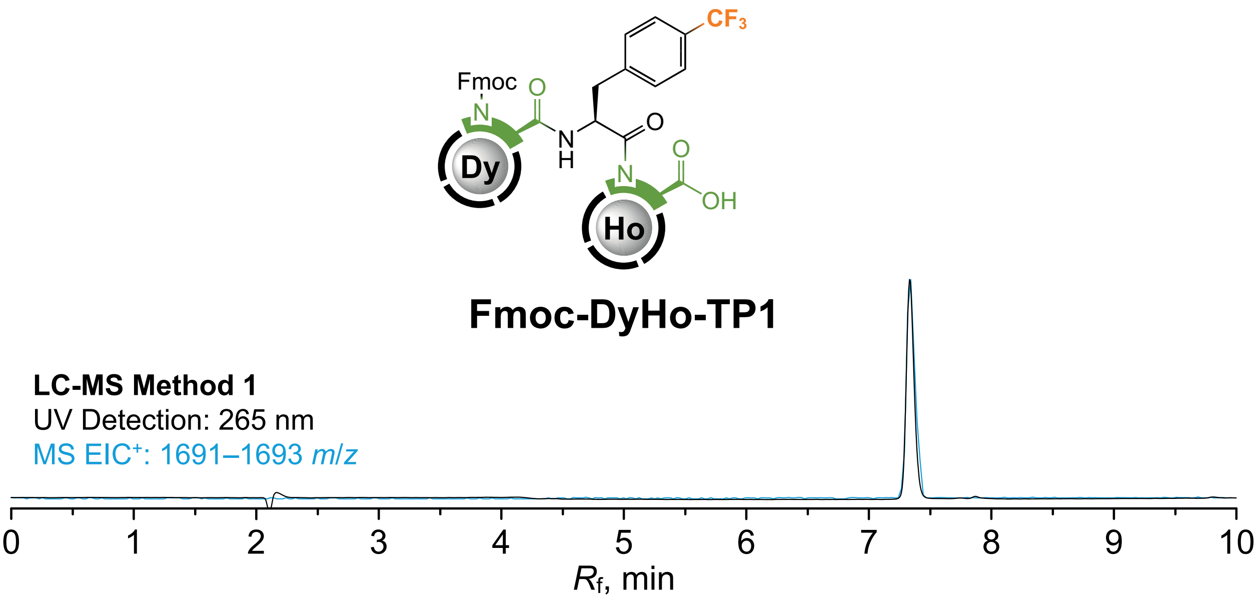

Supplementary Fig. 84. Synthesis and LC-MS chromatogram of intermediate Fmoc-DyHo-TP1. ***Synthesis:*** In a glass vial (4 mL), **Dy-V** (7.0 mg, 5.9 µmol assuming **Dy-V**·1.0TFA, 1.0 equiv.) and [**Ho**(**L**^{1})]·1.1TFA·3.0H_2O (6.4 mg, 7.8 µmol, 1.3 equiv.) were dissolved in dry DMSO (550 µL) followed by addition of PyAOP (100 mM in dry DMSO, 86 µL, 8.6 µmol, 1.5 equiv.) and DIPEA (13.6 µL, 78.0 µmol, 13 equiv.). The resulting solution was stirred at RT for 15 mins. The mixture was then purified by preparative HPLC (C18, H_2O/MeCN gradient with 0.1% FA additive). Fractions with product were joined and lyophilized to give product as pinkish solid.**Yield:** 3.6 mg (36%; 1 step; based on **Dy-V**·1.0TFA assuming **Fmoc-DyHo-TP1**, M_R = 1692). **ESI-HRMS:** 847.1985 $[M+2H]^{2+}$ (theor. $[C_{63}H_{78}O_{20}N_{11}F_3Ho_1Dy_1]^{2+}$ = 847.1980).

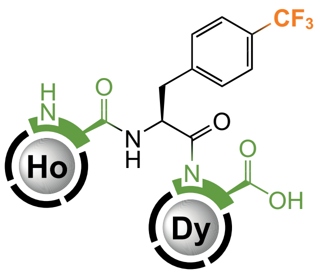

HoDy-TP1

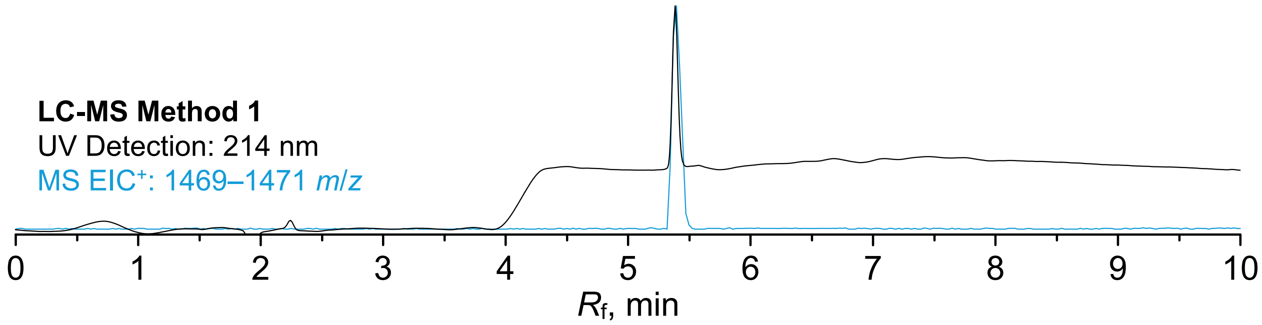

Supplementary Fig. 85. Synthesis and LC-MS chromatogram of HoDy-TP1. ***Synthesis:*** In a glass vial (4 mL), **Fmoc-DyHo-TP1** (3.6 mg, 2.1 µmol, 1.0 equiv.) was dissolved in 2% DBU in DMF (213 µL, 29 µmol, 14 equiv.) and the solution was stirred at RT for 5 mins. Reaction was quenched with FA (1.1 µL, 29 µmol, 14 equiv.). The mixture was then purified by preparative HPLC (C18, H_2O/ MeCN gradient with 0.1% FA additive). Fractions with product were joined and lyophilized to give product as pinkish solid. **Yield:** 2.8 mg (75%; 1 step; based on **Fmoc-DyHo-TP1**). **NMR (aq. MOPS pH = 7.0, external D_2O):** ^{19}**F** (470.4 MHz, T = 298.2 K) δ_F –54.75 (CF_3, s). **ESI-HRMS:** 736.1645 $[M+2H]^{2+}$ (theor. $[C_{48}H_{68}O_{18}N_{11}F_3Ho_1Dy_1]^{2+}$ = 736.1640). **EA** ($C_{48}H_{66}N_{11}O_{18}Ho_1Dy_1 \cdot 3.6FA \cdot 6.3H_2O$, M_R = 1750): C 35.4 (35.2); H 5.0 (4.7); N 8.8 (9.0); F 3.3 (3.1).

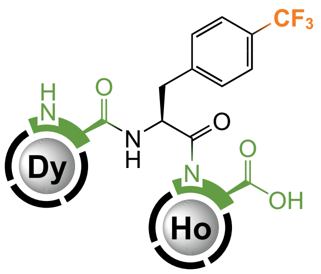

DyHo-TP1

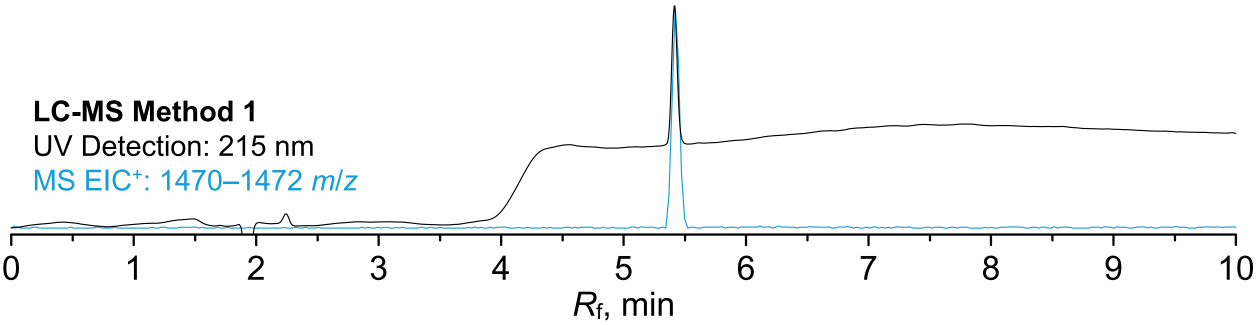

Supplementary Fig. 86. Synthesis and LC-MS chromatogram of DyHo-TP1. ***Synthesis:*** In a glass vial (4 mL), **Fmoc-DyHo-TP1** (3.6 mg, 2.1 µmol, 1.0 equiv.) was dissolved in 2% DBU in DMF (213 µL, 29 µmol, 14 equiv.) and the solution was stirred at RT for 5 mins. Reaction was quenched with FA (1.1 µL, 29 µmol, 14 equiv.). The mixture was then purified by preparative HPLC (C18, H_2O/ MeCN gradient with 0.1% FA additive). Fractions with product were joined and lyophilized to give product as pinkish solid. **Yield:** 2.2 mg (59%; 1 step; based on **Fmoc-DyHo-TP1** assuming **DyHo-TP1**·3.6FA·6.3H_2O according to **HoDy-TP1** analogy, M_R = 1750). **NMR (aq. MOPS pH = 7.0, external D_2O):** ^{19}**F** (470.4 MHz, T = 298.2 K) δ_F –62.74 (CF_3, s). **ESI-HRMS:** 736.1645 $[M+2H]^{2+}$ (theor. $[C_{48}H_{68}O_{18}N_{11}F_3Dy_1Ho_1]^{2+}$ = 736.1640).

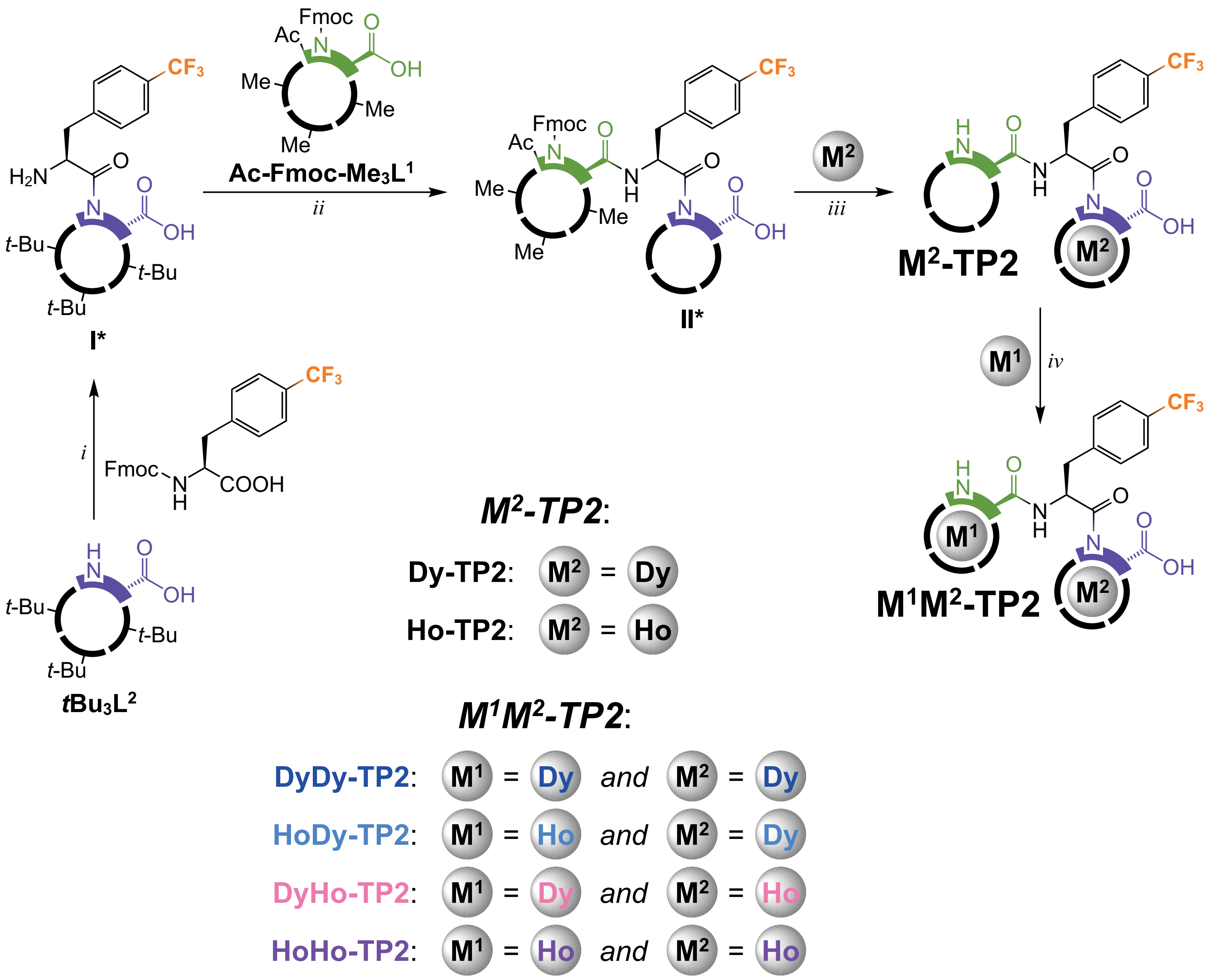

Supplementary Fig. 87. Synthesis of M^1M^2-TP2 tripeptides with Dy^{3+} and Ho^{3+} cations. ***Conditions:*** (*i*) Fmoc-Phe{*p*-CF_3}-OH, PyAOP, DIPEA, DMSO *followed by* DBU, DMF; (*ii*) **Ac-Fmoc-Me_3L^1**, PyAOP, DIPEA, DMSO *followed by* TFA; (*iii*) M^2Cl_3, aq. MOPS/NaOH buffer (pH 7.0) *followed by* LiOH, H_2O, MeOH; (*iv*) M^1Cl_3, aq. MOPS/NaOH buffer (pH 7.0).

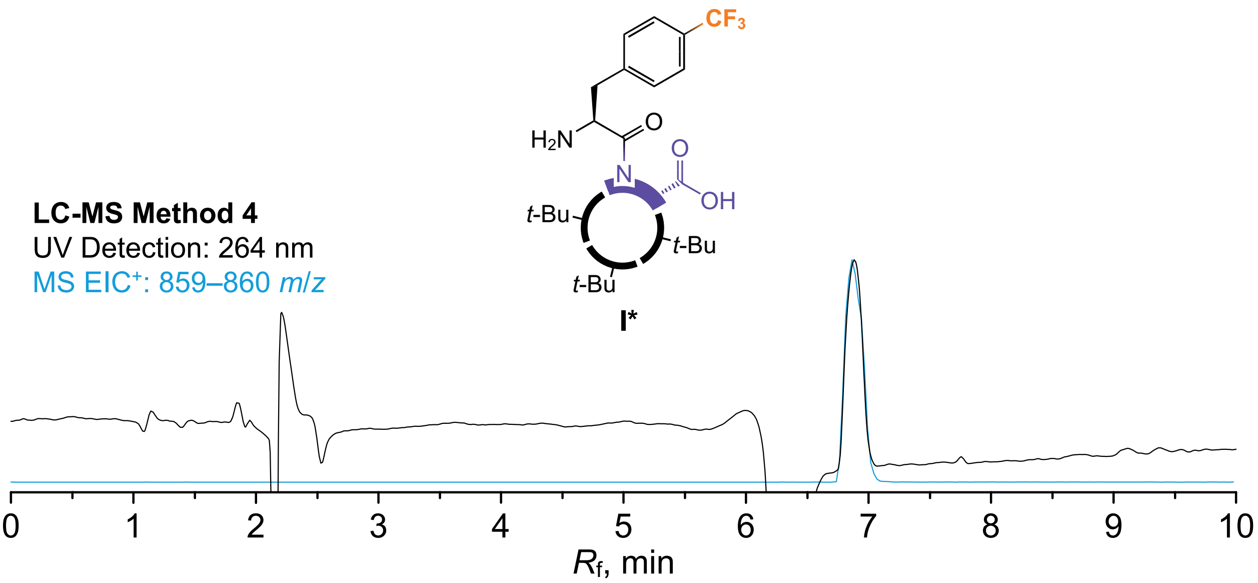

Supplementary Fig. 88. Synthesis and LC-MS chromatogram of intermediate I*. ***Synthesis:*** In a glass vial (4 mL), Fmoc-Phe{*p*-CF_3}-OH (9.7 mg, 21.3 µmol, 0.9 equiv.), PyAOP (11.1 mg, 21.3 µmol, 0.9 equiv.) and DIPEA (19 µL, 3106 µmol, 4.7 equiv.) were dissolved in dry DMSO (1.0 mL). After 1 min, ***t*Bu$_3$L^2**·3.0TFA·3.1H_2O (23.6 mg, 22.7 µmol, 1.0 equiv.) was added and solution was stirred at RT for 5 mins. Solution was purified by preparative HPLC (C18, H_2O/MeCN gradient with 0.1% FA additive). Fractions with Fmoc protected product were joined and lyophilized. The resulting white solid was dissolved in dry DMF (1.5 mL) followed by addition of DBU (30 µL, 202 µmol, 8.9 equiv.). After 5 mins, the reaction was quenched with TFA (15 µL, 202 µmol, 8.9 equiv.) and diluted with H_2O (1 mL). Solution was then purified by preparative HPLC (C18, H_2O/MeCN gradient with 0.1% FA additive). Fractions with product were joined and lyophilized to give product as white solid. **Yield:** 17 mg (87%; 2 steps; based on ***t*Bu$_3$L^2**·3.0TFA·3.1H_2O assuming zwitterionic form of **I**, M_R = 859.0). **ESI-HRMS:** 859.4786 $[M+H]^+$ (theor. $[C_{41}H_{66}F_3O_{10}N_6]^+$ = 859.4787).

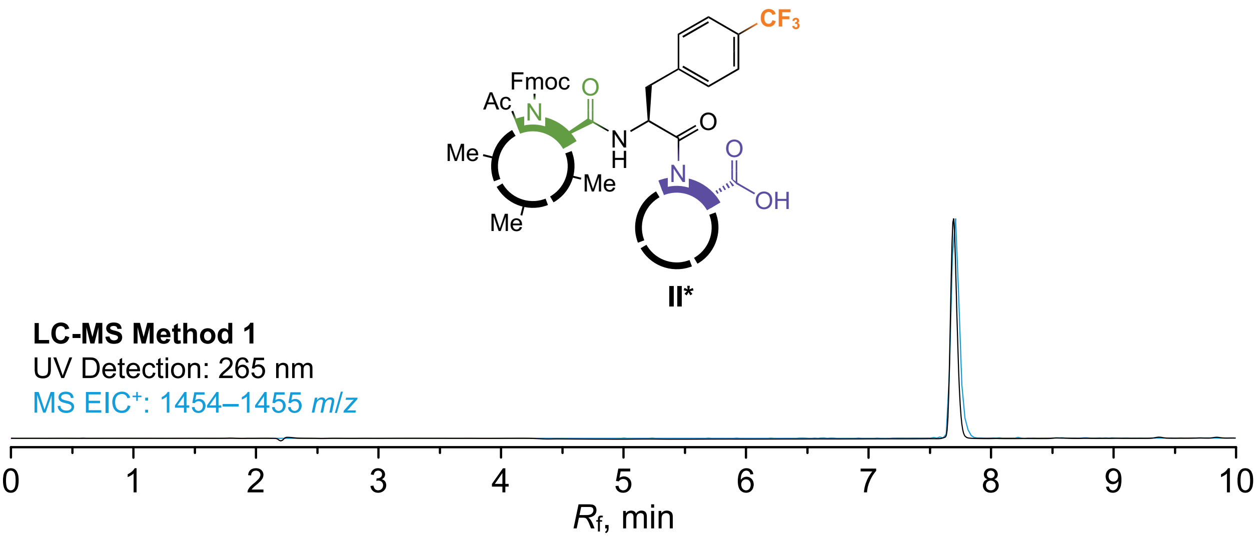

Supplementary Fig. 89. Synthesis and LC-MS chromatogram of intermediate II*. ***Synthesis:*** In a glass vial (4 mL), **Ac-Fmoc-Me$_3$L^1**·1.9TFA·1.3H_2O (7.4 mg, 7.1 µmol, 1.0 equiv.), PyOAP (3.7 mg, 7.1 µmol, 1.0 equiv.) and DIPEA (6 µL, 35 µmol, 5.0 equiv.) was dissolved in dry DMSO (300 µL). The resulting mixture was stirred at RT for 1 min followed by addition of solution of **I*** (9.0 mg, 10.5 µmol assuming zwitterionic form, 1.5 equiv.) in dry DMSO (100 µL) and the mixture was further stirred at RT for 5 mins. Solution was then purified by preparative HPLC (C18, H_2O/MeCN gradient with 0.1% FA additive). Fractions with *tert*-butyl product were joined and lyophilized. The resulting solid was dissolved in TFA (0.7 mL) and the resulting solution was stirred at RT for 16 h. Reaction mixture was evaporated to dryness and twice co-evaporated with MeOH. Residue was purified by preparative HPLC (C18, H_2O/MeCN gradient with 0.1% TFA additive). Fractions with product were joined and lyophilized to give product as white solid. **Yield:** 6.3 mg (52%; 2 steps; based on **Ac-Fmoc-Me$_3$L^1**·1.9TFA·1.3H_2O assuming **II***·2TFA, M_R = 1682). **ESI-HRMS:** 1454.6335 $[M+H]^+$ (theor. $[C_{68}H_{91}F_3O_{21}N_{11}]^+$ = 1454.6338).

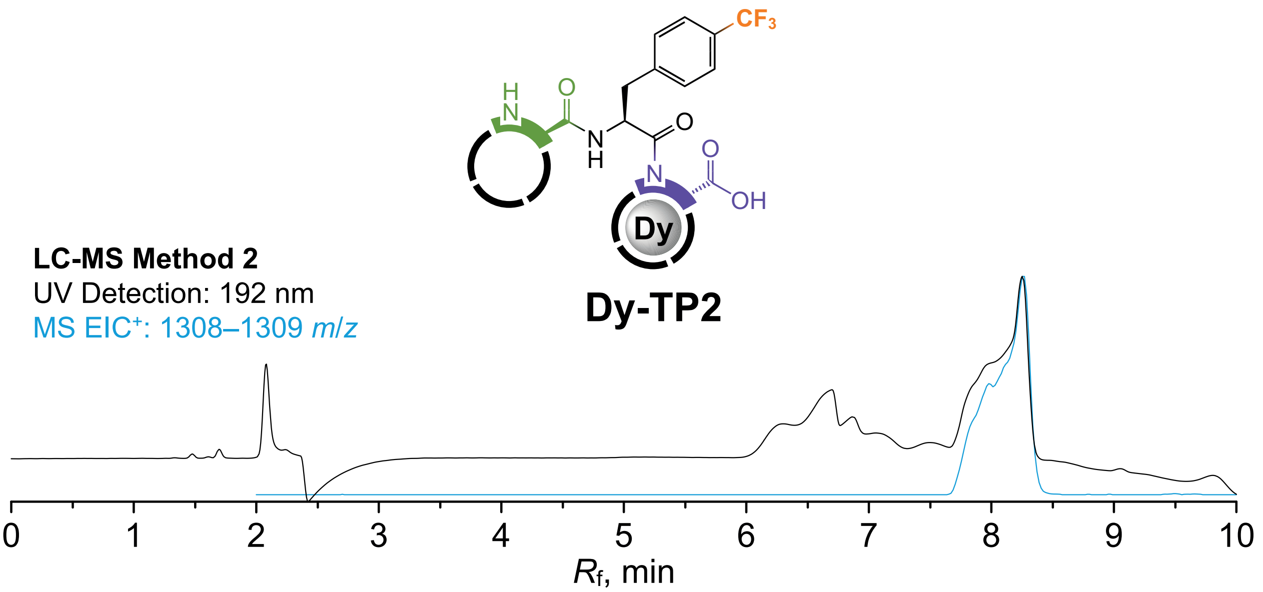

Supplementary Fig. 90. Synthesis and LC-MS chromatogram of intermediate Dy-TP2. ***Synthesis:*** In a glass vial (4 mL), **II*** (2.0 mg, 1.2 µmol assuming **II***·2TFA, 1.0 equiv.) was dissolved in aq. MOPS/NaOH buffer (500 mM, pH 7.0, 400 µL, 200 µmol, 170 equiv) followed by addition of aq. $DyCl_3$ (100 mM, 17 µL, 1.7 µmol, 1.4 equiv.). The resulting solution was stirred at RT for 16 h. The mixture was then purified by preparative HPLC (C18, H_2O/MeCN gradient with 0.1% FA additive). Fractions with product were joined and lyophilized to give product as white solid. The resulting white solid was dissolved in a mixture of MeOH (600 µL) and H_2O (50 µL) followed by addition of aq. LiOH (1 M, 40 µL, 40 µmol, 34 equiv.) and the mixture was stirred at RT for 2 d. Reaction was then quenched by FA (2.0 µL, 40 µmol, 34 equiv.) and the mixture was evaporated to dryness. The residue was purified by preparative HPLC (C18, H_2O/MeCN gradient with 0.1% FA additive). Fractions with product were joined and lyophilized to give product as white solid. **Yield:** 0.7 mg (41%; 2 steps; based on **II***·2TFA assuming **Dy-TP2**·2.5TFA, M_R = 1423). **ESI-HRMS:** 655.2113 $[M+2H]^{2+}$ (theor. $[C_{48}H_{71}O_{18}N_{11}F_3Dy_1]^{2+}$ = 655.2106).

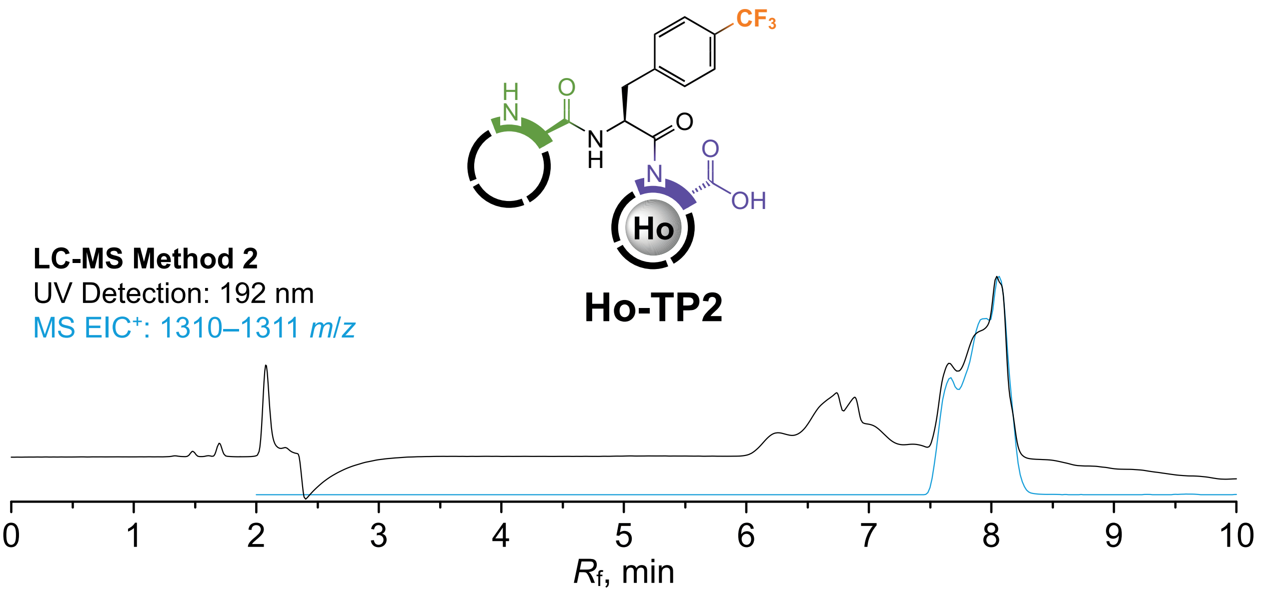

Supplementary Fig. 91. Synthesis and LC-MS chromatogram of intermediate Ho-TP2. ***Synthesis:*** In a glass vial (4 mL), **II*** (2.0 mg, 1.2 µmol assuming **II***·2TFA, 1.0 equiv.) was dissolved in aq. MOPS/NaOH buffer (500 mM, pH 7.0, 400 µL, 200 µmol, 170 equiv) followed by addition of aq. $HoCl_3$ (100 mM, 17 µL, 1.7 µmol, 1.4 equiv.). The resulting solution was stirred at RT for 16 h. The mixture was then purified by preparative HPLC (C18, H_2O/MeCN gradient with 0.1% FA additive). Fractions with product were joined and lyophilized to give product as white solid. The resulting white solid was dissolved in a mixture of MeOH (600 µL) and H_2O (50 µL) followed by addition of aq. LiOH (1 M, 40 µL, 40 µmol, 34 equiv.) and the mixture was stirred at RT fot 2 d. Reaction was then quenched by FA (2.0 µL, 40 µmol, 34 equiv.) and the mixture was evaporated to dryness. The residue was purified by preparative HPLC (C18, H_2O/MeCN gradient with 0.1% FA additive). Fractions with product were joined and lyophilized to give product as white solid. **Yield:** 0.8 mg (47%; 2 steps; based on **II***·2TFA assuming **Ho-TP2**·2.5TFA, M_R = 1425). **ESI-HRMS:** 655.7117 $[M+2H]^{2+}$ (theor. $[C_{48}H_{71}O_{18}N_{11}F_3Ho_1]^{2+}$ = 655.7111).

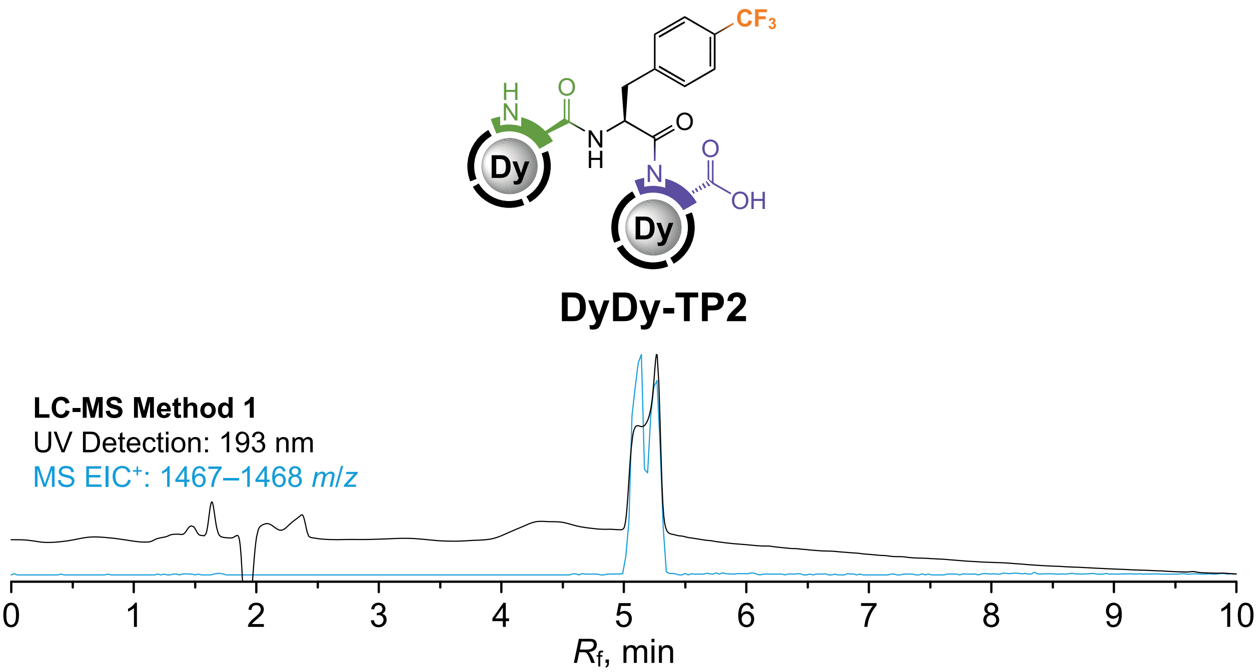

Supplementary Fig. 92. Synthesis and LC-MS chromatogram of DyDy-TP2. ***Synthesis:*** In a glass vial (4 mL), **Dy-TP2** (0.3 mg, ~0.2 µmol assuming **Dy-TP2**·2.5FA, 1.0 equiv.) was dissolved in aq. MOPS/NaOH buffer (500 mM, pH 7.0, 500 µL, 250 µmol, ~1200 equiv.) followed by addition of aq. $DyCl_3$ (100 mM, 4 µL, 0.4 µmol, ~1.9 equiv.) and the resulting solution was stirred at RT for 15 mins. The mixture was then purified by preparative HPLC (C18, H_2O/MeCN gradient with 0.1% TFA additive). Fractions with product were joined and lyophilized to give product as white solid. **Yield:** ~0.2 mg. **NMR (aq. MOPS pH = 7.0, external D_2O):** ^{19}**F** (470.4 MHz, T = 298.2 K) δ_F –90.53 (CF_3, s). **ESI-HRMS:** 735.6636 $[M+2H]^{2+}$ (theor. $[C_{48}H_{68}O_{18}N_{11}F_3Dy_2]^{2+}$ = 735.6634).

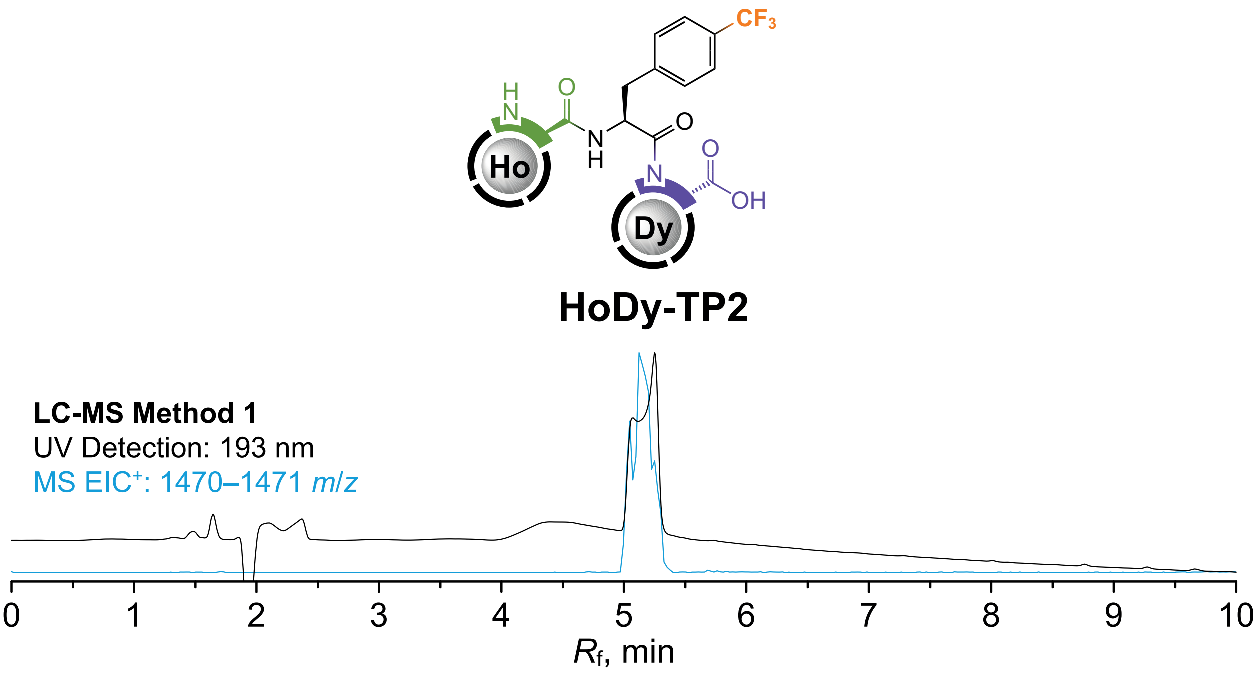

Supplementary Fig. 93. Synthesis and LC-MS chromatogram of HoDy-TP2. ***Synthesis:*** In a glass vial (4 mL), **Dy-TP2** (0.3 mg, ~0.2 µmol assuming **Dy-TP2**·2.5FA, 1.0 equiv.) was dissolved in aq. MOPS/NaOH buffer (500 mM, pH 7.0, 500 µL, 250 µmol, ~1200 equiv.) followed by addition of aq. $HoCl_3$ (100 mM, 4 µL, 0.4 µmol, ~1.9 equiv.) and the resulting solution was stirred at RT for 15 mins. The mixture was then purified by preparative HPLC (C18, H_2O/MeCN gradient with 0.1% TFA additive). Fractions with product were joined and lyophilized to give product as white solid. **Yield:** ~0.3 mg. **NMR (aq. MOPS pH = 7.0, external D_2O):** ^{19}**F** (470.4 MHz, T = 298.2 K) δ_F –91.95 (CF_3, s). **ESI-HRMS:** 736.1637 $[M+2H]^{2+}$ (theor. $[C_{48}H_{68}O_{18}N_{11}F_3Ho_1Dy_1]^{2+}$ = 736.1640).

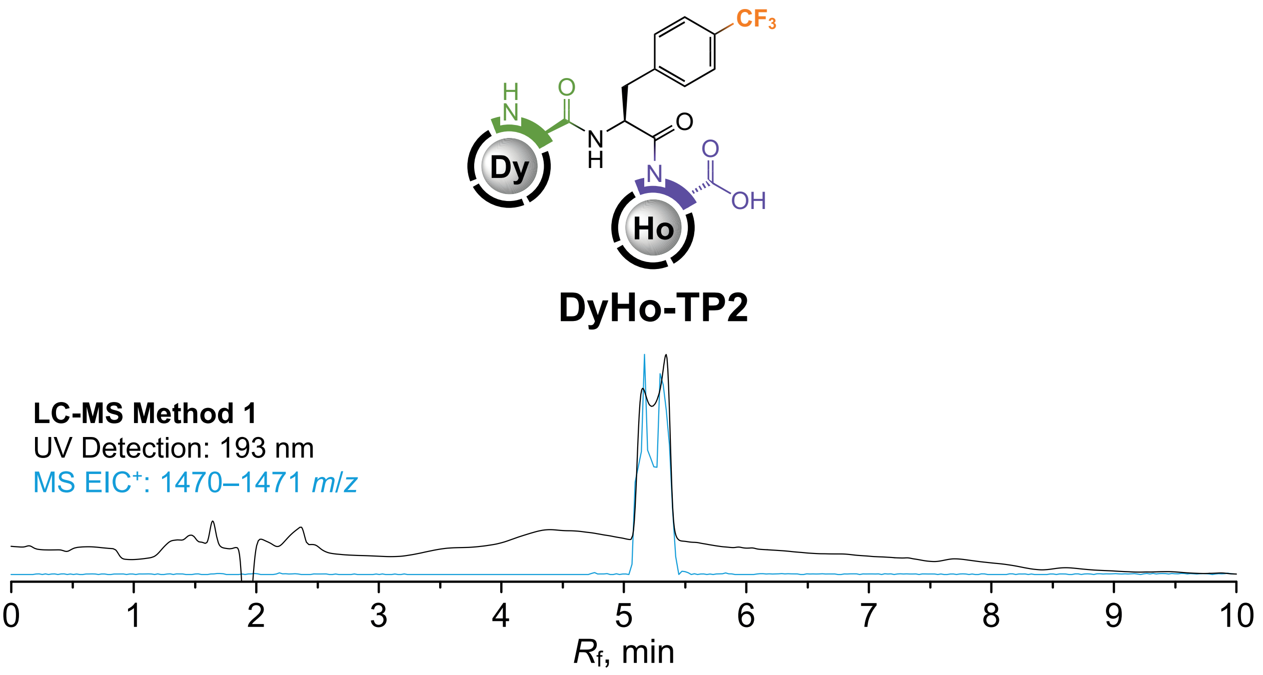

Supplementary Fig. 94. Synthesis and LC-MS chromatogram of DyHo-TP2. ***Synthesis:*** In a glass vial (4 mL), **Ho-TP2** (0.4 mg, ~0.3 µmol assuming **Ho-TP2**·2.5FA, 1.0 equiv.) was dissolved in aq. MOPS/NaOH buffer (500 mM, pH 7.0, 500 µL, 250 µmol, ~900 equiv.) followed by addition of aq. $DyCl_3$ (100 mM, 4 µL, 0.4 µmol, ~1.4 equiv.) and the resulting solution was stirred at RT for 15 mins. The mixture was then purified by preparative HPLC (C18, H_2O/MeCN gradient with 0.1% TFA additive). Fractions with product were joined and lyophilized to give product as white solid. **Yield:** ~0.3 mg. **NMR (aq. MOPS pH = 7.0, external D_2O):** ^{19}**F** (470.4 MHz, T = 298.2 K) δ_F –75.75 (CF_3, s). **ESI-HRMS:** 736.1645 $[M+2H]^{2+}$ (theor. $[C_{48}H_{68}O_{18}N_{11}F_3Dy_1Ho_1]^{2+}$ = 736.1640).

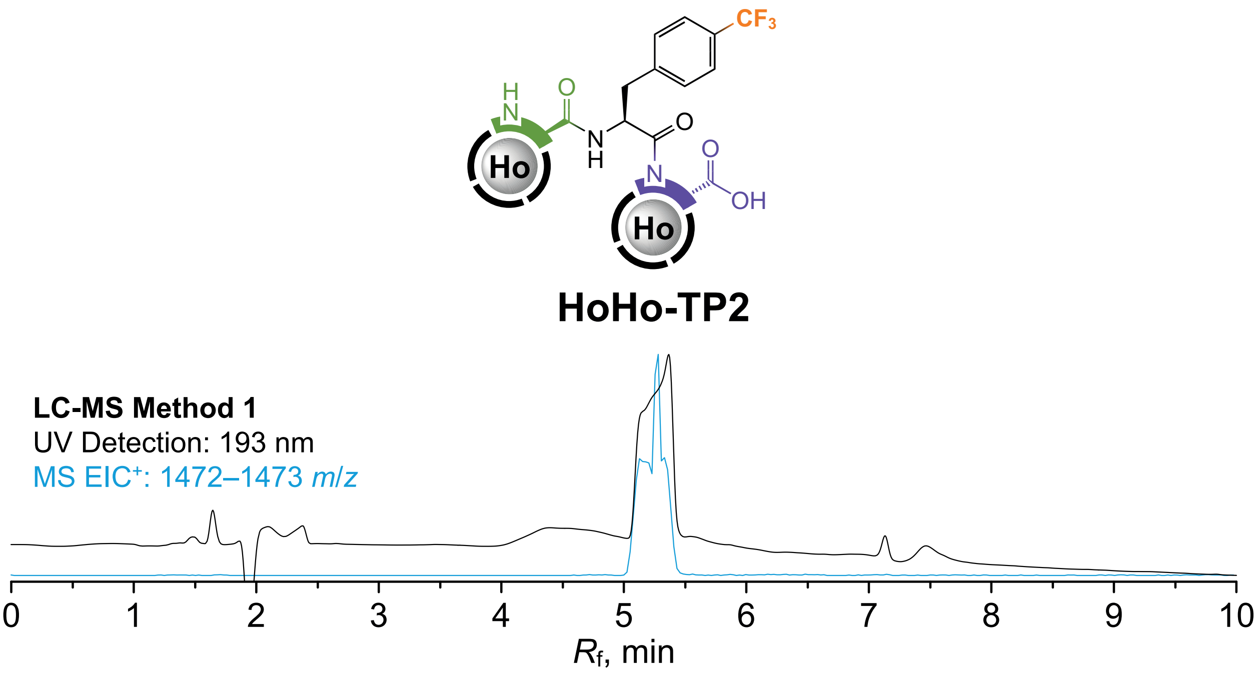

Supplementary Fig. 95. Synthesis and LC-MS chromatogram of HoHo-TP2. ***Synthesis:*** In a glass vial (4 mL), **Ho-TP2** (0.4 mg, ~0.3 µmol assuming **Ho-TP2**·2.5FA, 1.0 equiv.) was dissolved in aq. MOPS/NaOH buffer (500 mM, pH 7.0, 500 µL, 250 µmol, ~900 equiv.) followed by addition of aq. $HoCl_3$ (100 mM, 4 µL, 0.4 µmol, ~1.4 equiv.) and the resulting solution was stirred at RT for 15 mins. The mixture was then purified by preparative HPLC (C18, H_2O/MeCN gradient with 0.1% TFA additive). Fractions with product were joined and lyophilized to give product as white solid. **Yield:** ~0.4 mg. **NMR (aq. MOPS pH = 7.0, external D_2O):** ^{19}**F** (470.4 MHz, T = 298.2 K) δ_F –77.42 (CF_3, s). **ESI-HRMS:** 736.6644 $[M+2H]^{2+}$ (theor. $[C_{48}H_{68}O_{18}N_{11}F_3Ho_2]^{2+}$ = 736.6646).

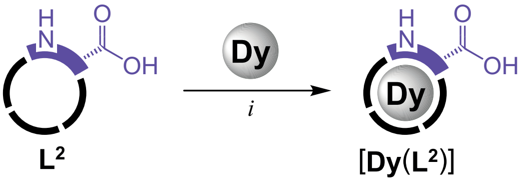

Supplementary Fig. 96. Synthesis of [Dy(L²)] building block for the purpose of crystalization experiments. ***Conditions:*** (*i*) $DyCl_3$, aq. MOPS/NaOH buffer (pH 7.0).

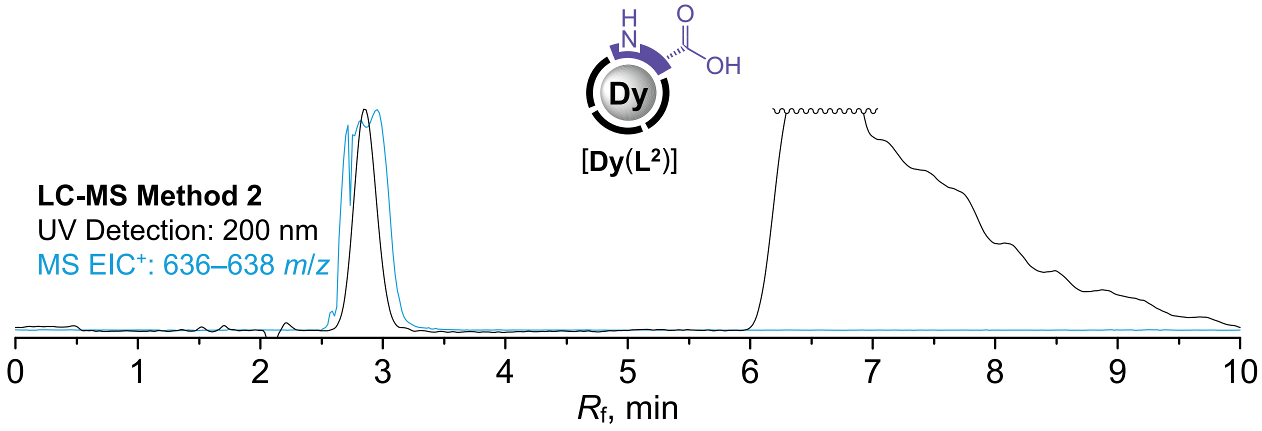

Supplementary Fig. 97. Synthesis and LC-MS chromatogram of building block [Dy(L²)]. ***Synthesis:*** In a glass vial (4 mL), **L²**·2.4TFA·2.5H_2O (30.0 mg, 37.8 µmol, 1.0 equiv.) was dissolved in aq. MOPS/NaOH buffer (0.5 M, pH 7.0, 759 µL, 380 µmol, 10 equiv.) followed by addition of aq. $DyCl_3$ (100 mM, 778 µL, 77.8 µmol, 2.1 equiv.) and the resulting solution was stirred at RT for 15 mins. The mixture was then purified by preparative HPLC (C18, H_2O/MeCN gradient with 0.1% TFA additive). Fractions with product were joined and re-purified by preparative HPLC (C18, H_2O/MeCN gradient with 0.1% FA additive) lyophilized to give product as white solid. **Yield:** 15.2 mg (58%; 1 step; based on **L²**·2.4TFA·2.5H_2O). **ESI-HRMS:** 637.1410 $[M+H]^+$ (theor. $[C_{19}H_{31}O_9N_5Dy_1]^+$ = 637.1408). **EA** ($C_{19}H_{30}N_5O_9Dy_1$·0.2TFA·1.7H_2O, M_R = 688.4): C 33.8 (34.4); H 4.9 (5.0); N 10.2 (9.6); F 1.7 (1.6); Dy 23.6 (20.6). ***Preparation of single crystals:*** In a glass vial (4 mL), aq. solution of [**Dy**(**L²**)] (~20 mM, 60 µL, ~1.20 µmol, 1.0 equiv.; filtered through syringe microfilter) was mixed with aq. solution of $HClO_4$ (~1.0 M, 1.22 µL, 1.22 µmol, ~1.0 equiv.) and the resulting solution was briefly vortexed. Then, THF (291 µL), *1,4*-dioxane (100 µL) and H_2O (100 µL) was added and the resulting mixture was filtered through syringe microfilter to a new glass vial (40 mL). Then *1,4*-dioxane (~100 µL) was slowly added dropwise until opalescence occured. The mixture was then sealed with a cap and gently heated using heatgun until clear solution was produced. The mixture was then left standing at RT for 1 week, producing single crystals of [**Dy**(**L²**)]·3H_2O suitable for X-Ray analysis.

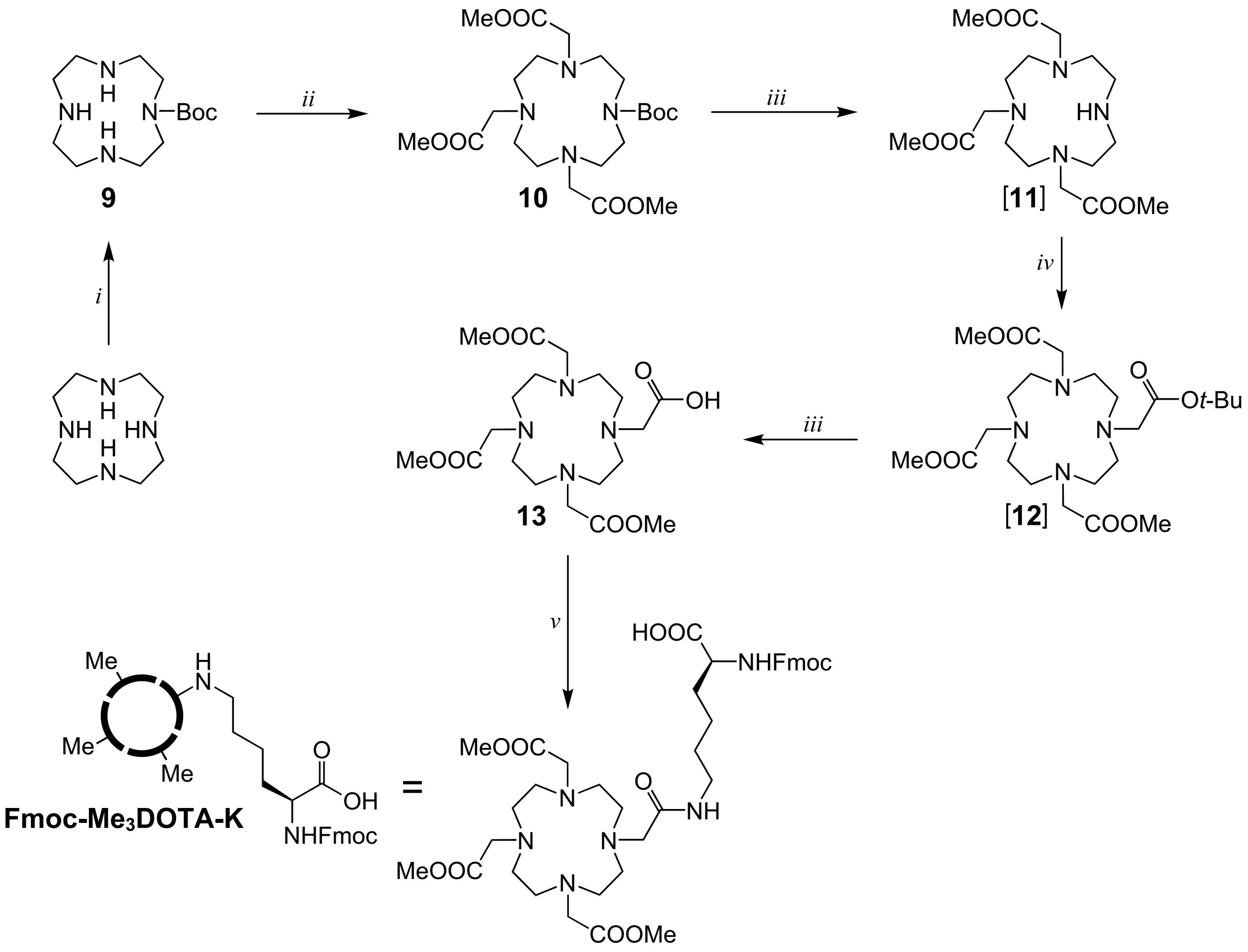

Supplementary Fig. 98. Synthesis of building block Fmoc-Me$_3$DOTA-K. ***Conditions:*** (*i*) *tert*-butyl (4-nitrophenyl) carbonate, DCM; (*ii*) MeO_2CCH_2Br, K_2CO_3, MeCN; (*iii*) TFA; (*iv*) *t*-BuO_2CCH_2Br, K_2CO_3, MeCN; (*v*) Fmoc-lysine-OH, PyAOP, DIPEA, DMSO. Intermediates in brackets were not isolated.

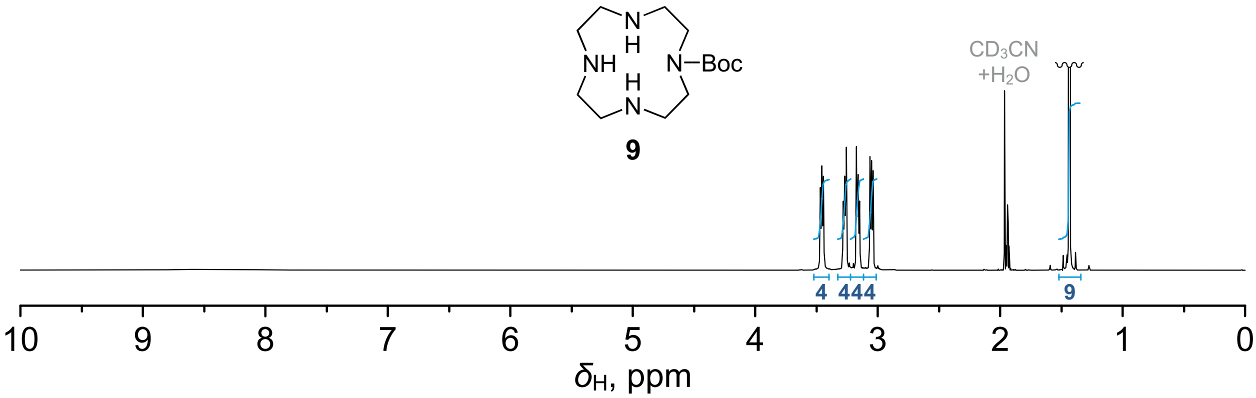

Supplementary Fig. 99. Synthesis and ^{1}H NMR spectrum ($CDCl_3$, 400.1 MHz, *T* = 300 K) of intermediate 9. ***Synthesis:*** In a round-bottom glass flask (50 mL), cyclen (172 mg, 1.00 mmol, 1.0 equiv.) was dissolved in DCM (10 mL) and tert-butyl (4-nitrophenyl) carbonate (239 mg, 1.00 mmol, 1.0 equiv.) in DCM (10 mL) was added dropwise. Reaction mixture was stirred at RT for 2 h. Mixture was then evaporated to dryness and residue was purified by preparative HPLC (C18, H_2O/MeCN gradient with 0.1% TFA additive). Fractions with product were joined and immediately lyophilized to give product as white solid. **Yield:** 300 mg (57%; 1 step; based on **cyclen**); **NMR (CD_3CN):** **^{1}H** (400.1 MHz, *T* = 300 K) δ_H 1.43 (CH_3, s, 9H); 3.01–3.09 (*mc*, m, 4H); 3.12–3.19 (*mc*, m, 4H); 3.24–3.30 (*mc*, m, 4H); 3.42–3.48 (*mc*, m, 4H). **^{13}C{^{1}H}** (100.6 MHz, *T* = 300 K) δ_C 28.49 (CH_3, s); 45.14 (*mc*, s); 45.73 (*mc*, s); 48.64 (*mc*, s); 48.91 (*mc*, s); 81.57 (*C*–CH_3, s); 156.71 (*C*O, s). **ESI-HRMS:** 273.2288 $[M+H]^+$ (theor. $[C_{13}H_{29}O_2N_4]^+$ = 273.2285). **EA** ($C_{13}H_{28}N_4O_2$·2.1TFA·0.8H_2O, M_R = 526.2)**:** C 38.3 (38.9); H 6.1 (5.2); N 10.7 (10.2); F 22.7 (21.9).

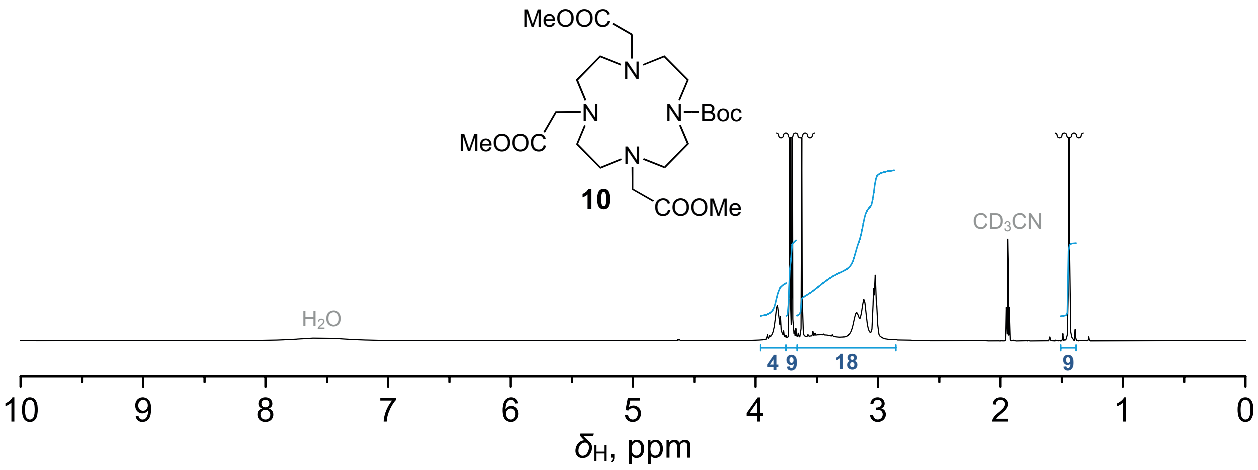

Supplementary Fig. 100. Synthesis and ^{1}H NMR spectrum ($CDCl_3$, 400.1 MHz, T = 300 K) of intermediate 10. ***Synthesis:*** In a glass vial (20 mL), **9**·2.1TFA·0.8H_2O (79 mg, 0.15 mmol, 1.0 equiv.) was dissolved in MeCN (5 mL) followed by addition of K_2CO_3 (76 mg, 0.55 mmol, 3.8 equiv.) and methyl bromoacetate (52 µL, 0.55 mmol, 3.8 equiv.). The resulting suspension was stirred at RT for 16 h. Mixture was then filtered through syringe microfilter (PTFE) and the filtrate was evaporated to dryness. Residue was purified by preparative HPLC (C18, H_2O/MeCN gradient with 0.1% TFA additive). Fractions with product were joined and immediately lyophilized to give product as white solid. **Yield:** 73 mg (70%; 1 step; based on **9**·2.1TFA·0.8H_2O assuming **10**·2.0TFA, M_R = 716.6). **NMR ($CDCl_3$): ^{1}H** (400.1 MHz, T = 300 K) δ_H 1.44 (CH_3–C, s, 9H); 2.86–3.65 (*mc*, CH_2–CO, m, 16H+2H); 3.70 (CH_3–O, s, 3H); 3.72 (CH_3–O, s, 6H); 3.85 (CH_2–CO, bs, 4H). **^{13}C{^{1}H}** (100.6 MHz, T = 300 K) δ_C 28.50 (CH_3–C, s); 46.64 (*mc*, s); 50.09 (*mc*, s); 52.46–56.11 (*mc*, CH_2–CO, CH_3–O, m); 81.87 (*C*–CH_3, s); 156.99 (*C*O–N, s); 170.30 (*C*O–O, s); 171.89 (*C*O–O, s).

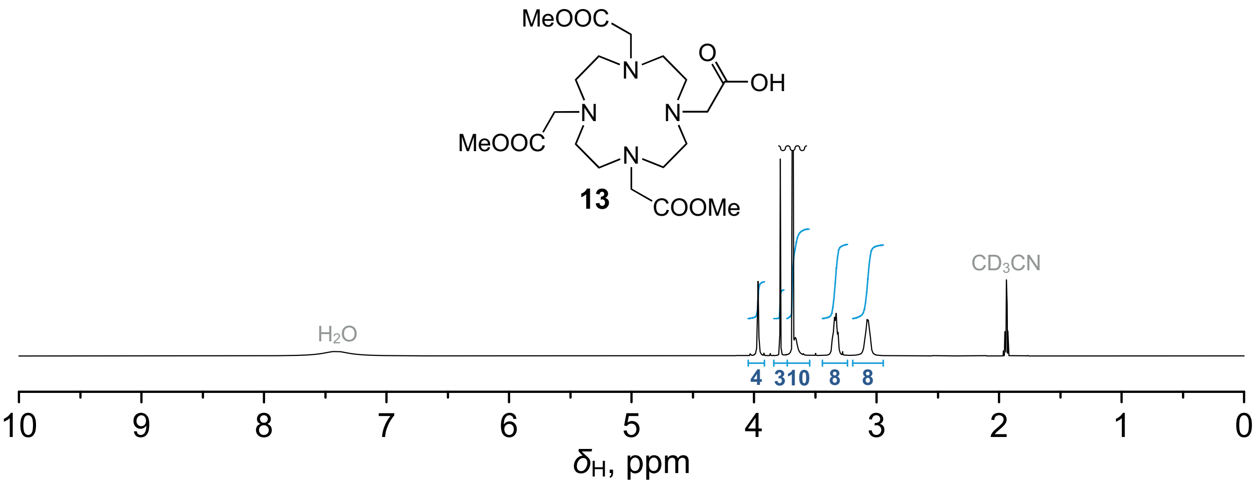

Supplementary Fig. 101. One-pot synthesis and ^{1}H NMR spectrum ($CDCl_3$, 400.1 MHz, *T* = 300 K) of intermediate 13. ***Synthesis:*** In a glass vial (20 mL), **10** (73 mg, 102 µmol assuming **10**·2.0TFA, 1.0 equiv.) was dissolved in TFA (3 mL, 39.2 mmol, 385 equiv.) and the resulting solution was stirred at RT for 2 h. Mixture was evaporated to dryness and twice co-evaporated with MeOH. Residue was purified by preparative HPLC (C18, H_2O/MeCN gradient with 0.1% TFA additive). Fractions with intermediate **11** were joined and immediately lyophilized. The resulting solid was dissolved in MeCN (5 mL) followed by addition of K_2CO_3 (40 mg, 291 µmol, 2.9 equiv.) and tert-butyl bromoacetate (21 mg, 107 µmol, 1.05 equiv.). The resulting suspension was stirred at RT for 2 h. Mixture was then filtered through syringe microfilter (PTFE) and the filtrate was evaporated to dryness. Residue containing intermediate **12** was dissolved in TFA (3 mL, 39.2 mmol, 385 equiv.) and the resulting solution was stirred at RT for 16 h. Mixture was evaporated to dryness and twice co-evaporated with MeOH. Residue was purified by preparative HPLC (C18, H_2O/MeCN gradient with 0.1% TFA additive). Fractions with product were joined and immediately lyophilized to give product as white solid. **Yield:** 27.2 mg (40%; 3 steps; based on **10**·2.0TFA assuming **13**·2.0TFA, M_R = 674.5). **NMR ($CDCl_3$):** ^{1}**H** (400.1 MHz, *T* = 300 K) δ_H 2.95–3.19 (*mc*, m, 8H); 3.24–3.45 (*mc*, m, 8H); 3.56–3.73 (CH_2–CO, CH_3–O, m, 4H+6H); 3.79 (CH_3–O, s, 3H); 3.97 (CH_2–CO, bs, 4H). ^{13}**C{^{1}H}** (100.6 MHz, *T* = 300 K) δ_C 49.65 (*mc*, s); 49.83 (*mc*, s); 52.16 (*mc*, s); 52.37 (*mc*, s); 52.86–55.81 (*mc*, CH_2–CO, CH_3–O, m); 168.81 (*C*O, s); 169.49 (*C*O–O, s); 172.20 (*C*O–O, s). **ESI-HRMS:** 447.2445 $[M+H]^+$ (theor. $[C_{19}H_{35}O_8N_4]^+$ = 447.2449).

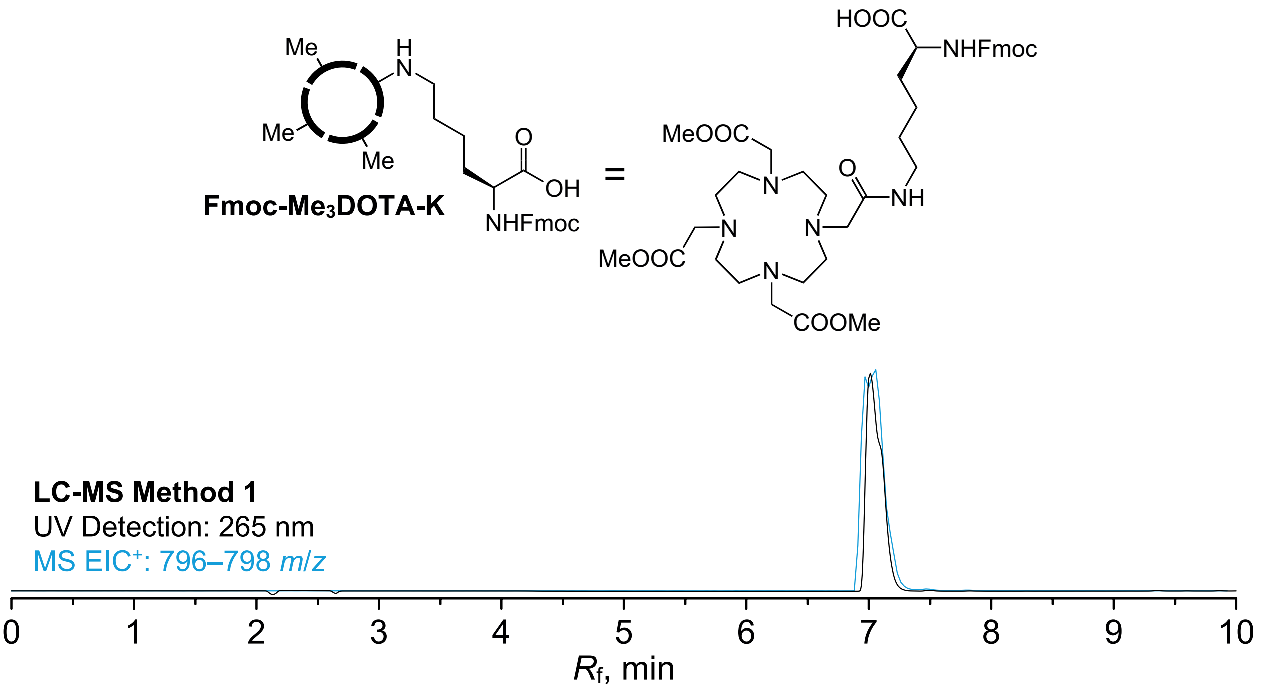

Supplementary Fig. 102. Synthesis and LC-MS chromatogram of building block Fmoc-Me$_3$DOTA-K. ***Synthesis:*** In a glass vial (4 mL), **10** (22 mg, 32.6 µmol assuming **10**·2.0TFA, 1.0 equiv.), PyAOP (17 mg, 32.6 µmol, 1.0 equiv.) and DIPEA (29 µL, 166 µmol, 5.1 equiv.) were dissolved in dry DMSO (620 µL). After 1 min of stirring, Fmoc-lysine-OH (16 mg, 32.6 µmol, 1.0 equiv.) was added and the resulting solution was stirred at RT for 5 mins. Mixture was then purified by preparative HPLC (C18, H_2O/MeCN gradient with 0.1% TFA additive). Fractions with product were joined and immediately lyophilized to give product as white solid. **Yield:** 11.1 mg (33%; 1 step; based on **10**·2.0TFA). **ESI-HRMS:** 797.4083 $[M+H]^+$ (theor. $[C_{40}H_{57}O_{11}N_6]^+$ = 797.4080). **EA** ($C_{40}H_{56}N_6O_{11}$·1.8TFA·1.3H_2O, M_R = 1025.5)**:** C 51.1 (51.8); H 5.9 (5.4); N 8.2 (7.4); F 10.0 (9.2).

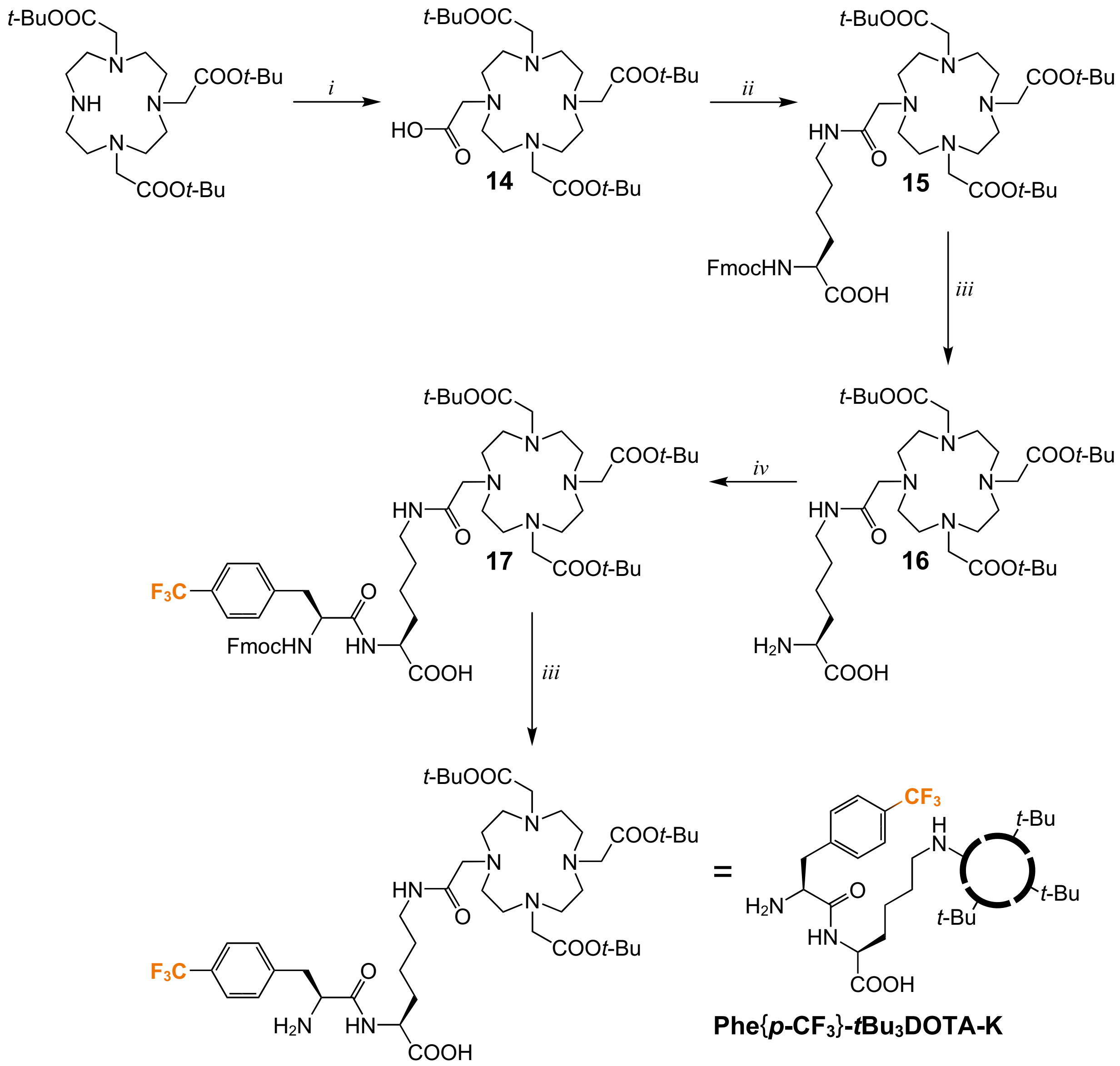

Supplementary Fig. 103. Synthesis of building block Phe{*p*-CF_3}-*t*Bu_3DOTA-K. ***Conditions:*** (*i*) MeO_2CCH_2Br, K_2CO_3, MeCN *followed by* LiOH, MeOH, H_2O; (*ii*) Fmoc-lysine-OH, PyAOP, DIPEA, DMSO; (*iii*) DBU, DMF; (*iv*) Fmoc-Phe{*p*-CF_3}-OH, PyAOP, DIPEA, DMSO.

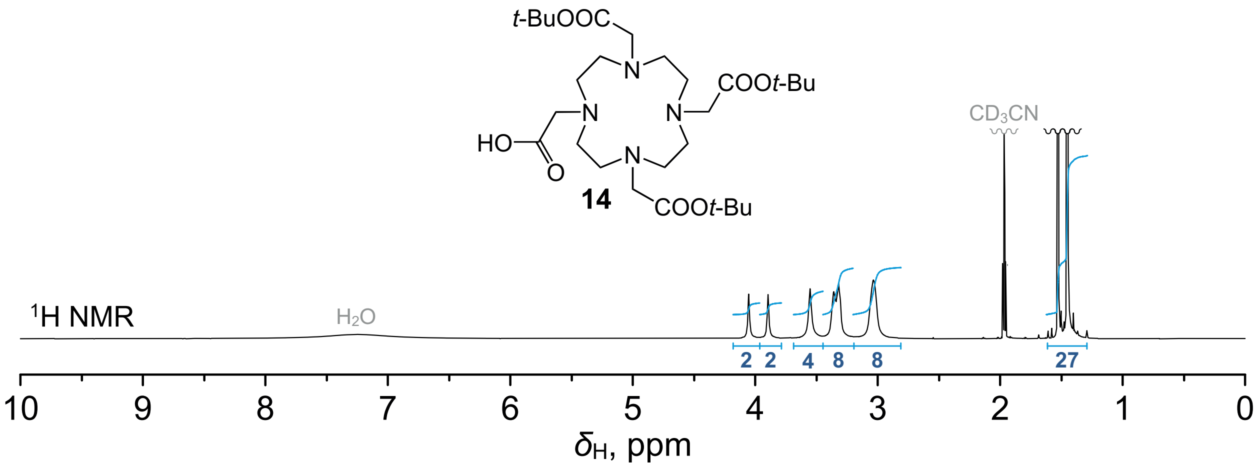

Supplementary Fig. 104. Synthesis and ^{1}H NMR spectrum ($CDCl_3$, 400.1 MHz, *T* = 300 K) of intermediate 14. ***Synthesis:*** In a pear-shaped glass flask (50 mL), **tBu$_3$DO3A**·HBr (242 mg, 0.41 mmol, 1.0 equiv), methyl bromoacetate (65 mg, 0.43 mmol, 1.05 equiv.) and K_2CO_3 (112 mg, 0.81 mmol, 2.0 equiv.) were mixed in 16 mL of MeCN. The mixture was stirred at RT for 20 h, followed by addition of H_2O (12 mL) and aq. LiOH (1.0 M, 2.03 mL, 2.03 mmol, 5.0 equiv.). The mixture was further stirred at RT for 18 h and then it was neutralized by TFA. Mixture was evaporated to dryness and the residue was purified by preparative HPLC (C18, H_2O/MeCN gradient with 0.1% TFA additive). Fractions with product were joined and lyophilized to give product as white solid. **Yield:** 225 mg (62%; 1 step; based on **tBu$_3$DO3A**·HBr). **NMR (CD_3CN): ^{1}H** (400.1 MHz, *T* = 300 K) δ_H 1.45 (C*H*$_3$, s, 18H); 1.53 (C*H*$_3$, s, 9H); 3.03 (*mc*, bs, 8H); 3.32 (*mc*, bs, 4H); 3.36 (*mc*, bs, 4H); 3.55 (C*H*$_2$–CO, s, 4H); 3.90 (C*H*$_2$–CO, s, 2H); 4.05 (C*H*$_2$–CO, s, 2H). **^{13}C{^{1}H}** (100.6 MHz, *T* = 300 K) δ_C 28.35 (*C*H$_3$, s); 28.38 (*C*H$_3$, s); 49.54 (*mc*, s); 49.78 (*mc*, s); 52.26 (2 × s); 54.90 (*C*H$_2$–CO, s); 55.73 (*C*H$_2$–CO, s); 55.83 (*C*H$_2$–CO, s); 83.35 (*C*–CH$_3$, s); 85.24 (*C*–CH$_3$, s); 168.78–171.14 (*C*O, m). **ESI-HRMS:** 573.3854 [M+H]$^+$ (theor. $[C_{28}H_{53}O_8N_4]^+$ = 573.3858). **EA** ($C_{28}H_{52}N_4O_8$·2.6TFA·1.1H_2O, M_R = 888.9): C 44.9 (45.1); H 6.4 (6.2); N 6.3 (6.1); F 16.7 (16.4).

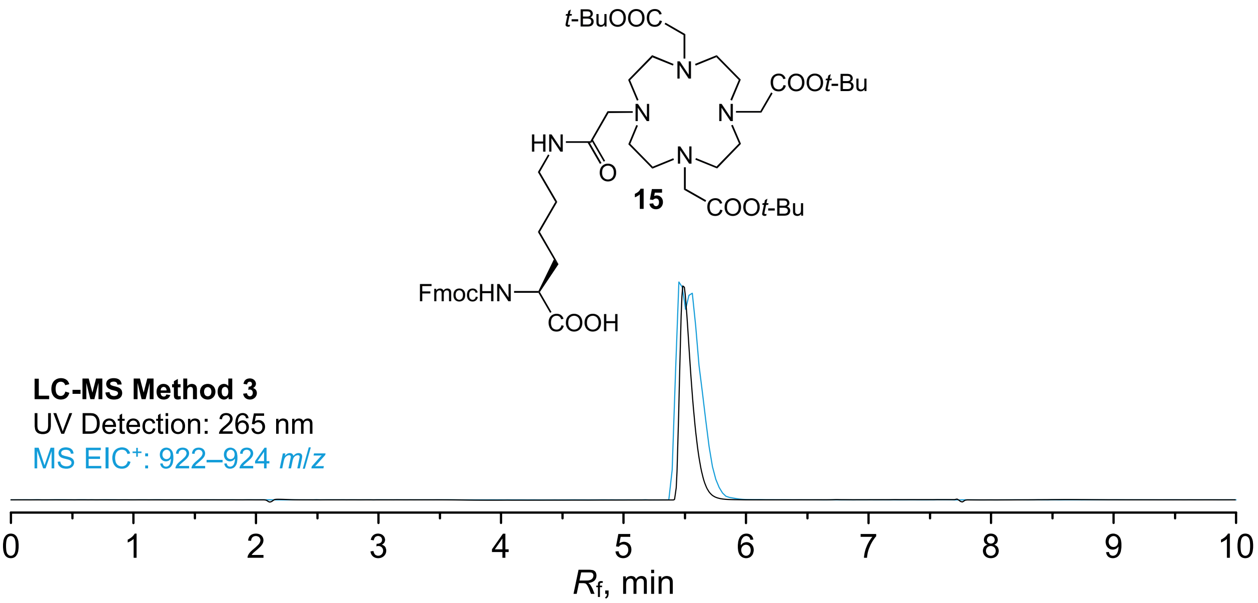

Supplementary Fig. 105. Synthesis and LC-MS chromatogram of intermediate 15. ***Synthesis:*** In a glass vial (4 mL), **14**·2.6TFA·1.1H_2O (66 mg, 74.3 µmol, 1.0 equiv.), PyAOP (39 mg, 71.0 µmol, 0.95 equiv.) and DIPEA (66 µL, 376 µmol, 5.1 equiv.) were dissolved in dry DMSO (1.4 mL). After 1 min of stirring, Fmoc-lysine-OH (37 mg, 75.1 µmol, 1.0 equiv.) was added and the resulting solution was stirred at RT for 5 mins. Mixture was then purified by preparative HPLC (C18, H_2O/MeCN gradient with 0.1% TFA additive). Fractions with product were joined and lyophilized to give product as white solid. **Yield:** 42.5 mg (50%; 1 step; based on **14**·2.6TFA·1.1H_2O assuming **15**·2.0TFA, M_R = 1151). **ESI-HRMS:** 923.5488 $[M+H]^+$ (theor. $[C_{49}H_{75}O_{11}N_6]^+$ = 923.5488).

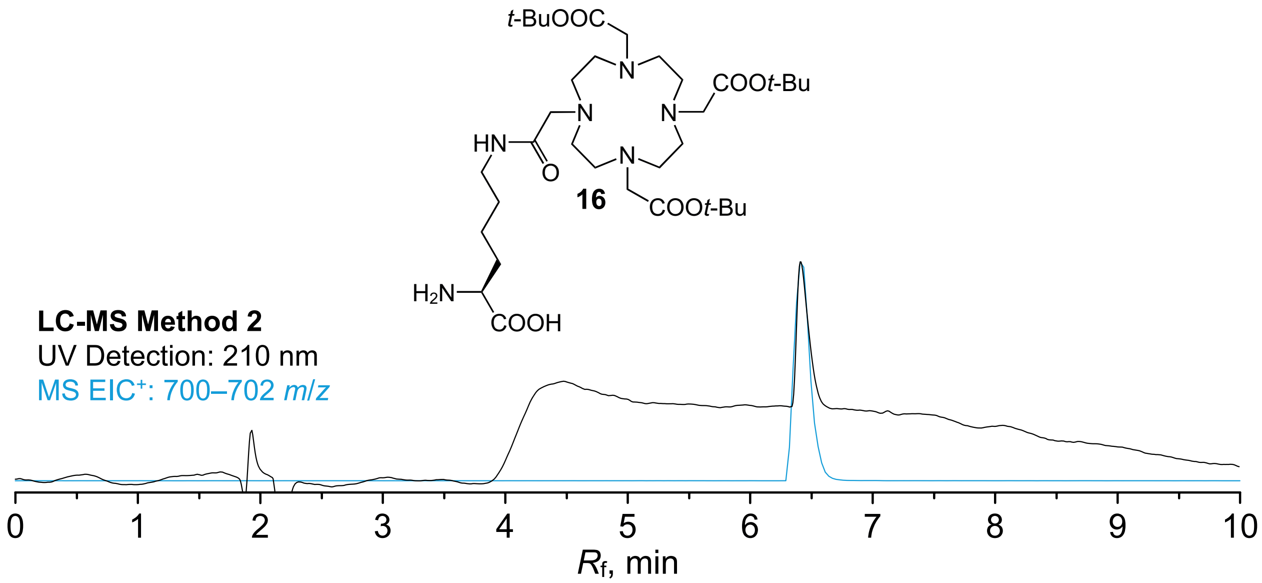

Supplementary Fig. 106. Synthesis and LC-MS chromatogram of intermediate 16. ***Synthesis:*** In a glass vial (4 mL), **15** (36.3 mg, 31.5 µmol assuming **15**·2.0TFA, 1.0 equiv.) was dissolved in DMF (2.5 mL) followed by addition of DBU (55 µL, 368 µmol, 11.7 equiv.). After 5 mins, the reaction was quenched with TFA (28 µL, 366 µmol, 11.7 equiv.) and diluted with H_2O (1 mL). Mixture was then purified by preparative HPLC (C18, H_2O/MeCN gradient with 0.1% TFA additive). Fractions with product were joined and lyophilized to give product as white solid. **Yield:** 22.4 mg (69%; 1 step; based on **15**·2.0TFA). **ESI-HRMS:** 701.4805 $[M+H]^+$ (theor. $[C_{34}H_{65}O_9N_6]^+$ = 701.4808). **EA** ($C_{34}H_{64}N_6O_9$·2.5TFA·2.3H_2O, M_R = 1027.3): C 45.6 (44.9); H 7.0 (6.2); N 8.2 (7.7); F 13.9 (13.3).

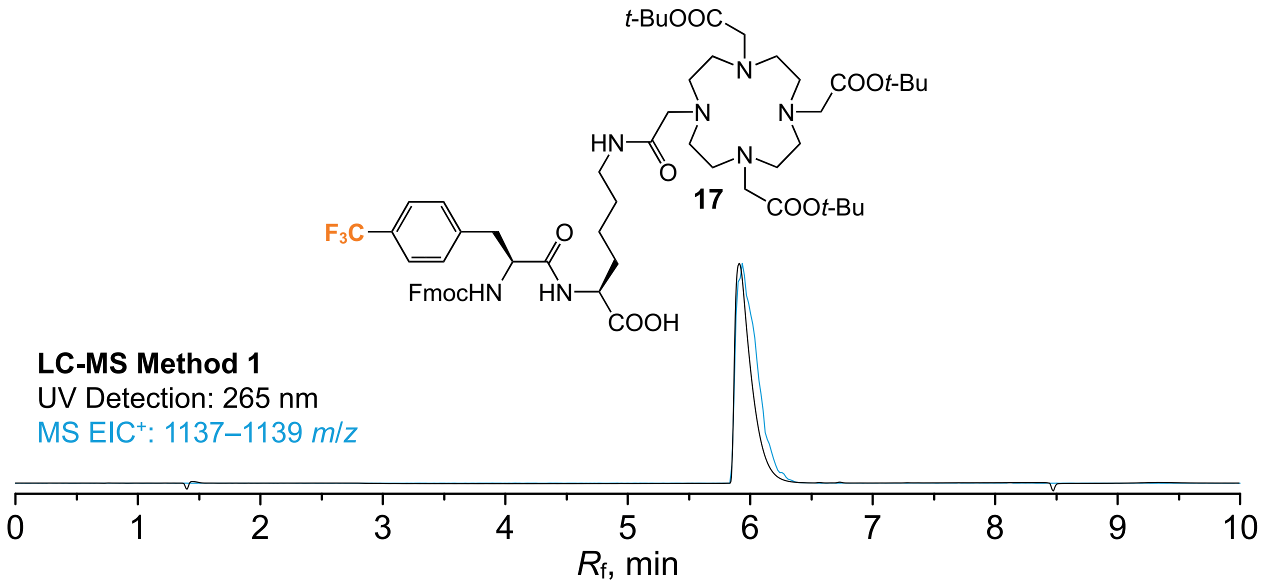

Supplementary Fig. 107. Synthesis and LC-MS chromatogram of intermediate 17. ***Synthesis:*** In a glass vial (4 mL), Fmoc-Phe{*p*-CF_3}-OH (100 mM in dry DMSO, 221 µL, 22.1 µmol, 1.0 equiv.), PyAOP (100 mM in dry DMSO, 221 µL, 22.1 µmol, 1.0 equiv.) and DIPEA (100 mM in dry DMSO, 221 µL, 22.1 µmol, 1.0 equiv.) were mixed together. After 1 min, **16** (100 mM in dry DMSO, 221 µL, 22.1 µmol, 1.0 equiv.) was added and the mixture was stirred at RT for 5 mins. Mixture was then purified by preparative HPLC (C18, H_2O/MeCN gradient with 0.1% TFA additive). Fractions with product were joined and lyophilized to give product as white solid. **Yield:** 20.0 mg 66%; 1 step; based on **16**·2.5TFA·2.3H_2O assuming **17**·2.0TFA, M_R = 1367). **ESI-HRMS:** 1138.6048 $[M+H]^+$ (theor. $[C_{59}H_{83}O_{12}N_7F_3]^+$ = 1138.6046).

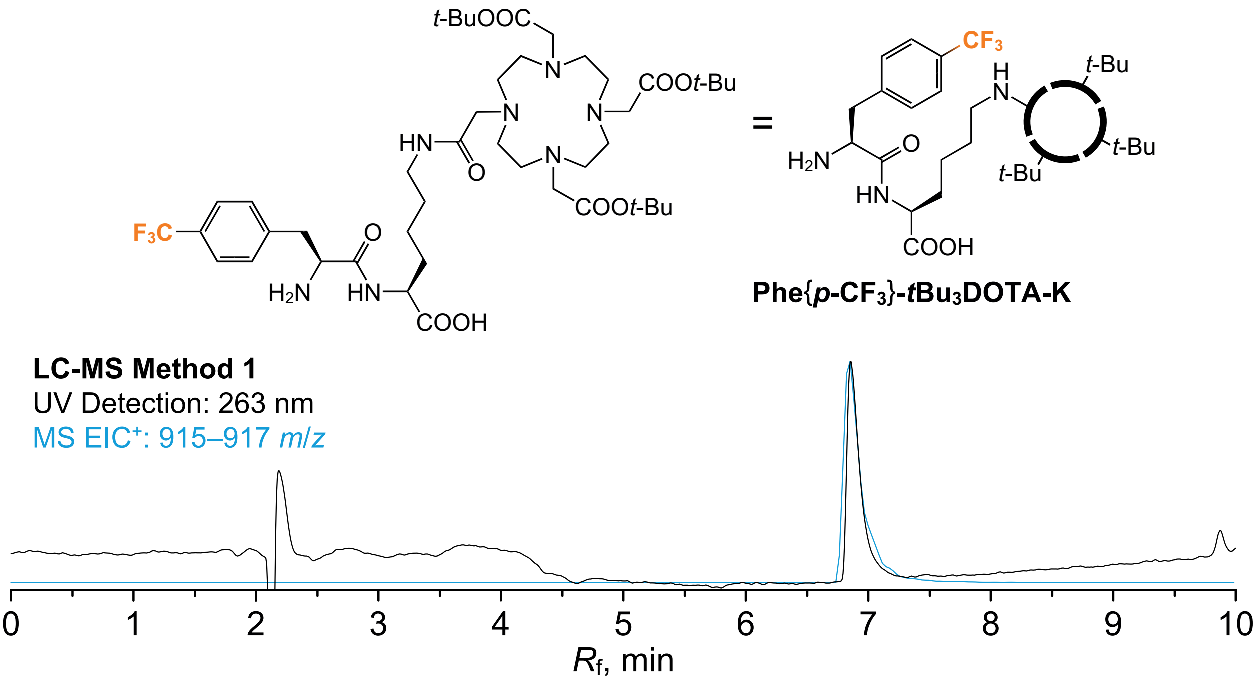

Supplementary Fig. 108. Synthesis and LC-MS chromatogram of building block Phe{*p*-CF_3}-*t*Bu_3DOTA-K. ***Synthesis:*** In a glass vial (4 mL), **17** (19 mg, 13.9 µmol assuming **19**·2.0TFA, 1.0 equiv.) was dissolved in DMF (1.4 mL) followed by addition of DBU (27 µL, 181 µmol, 13.0 equiv.). After 5 mins, the reaction was quenched with TFA (14 µL, 183 µmol, 13.1 equiv.) and diluted with H_2O (1 mL). Mixture was then purified by preparative HPLC (C18, H_2O/MeCN gradient with 0.1% TFA additive). Fractions with product were joined and lyophilized to give product as white solid. **Yield:** 11 mg (66%; 1 step; based on **19**·2.0TFA assuming **Phe{*p*-CF_3}-*t*Bu_3DOTA-K**·2.5TFA, M_R = 1201). **ESI-HRMS:** 916.5364 $[M+H]^+$ (theor. $[C_{44}H_{73}O_{10}N_7F_3]^+$ = 916.5366).

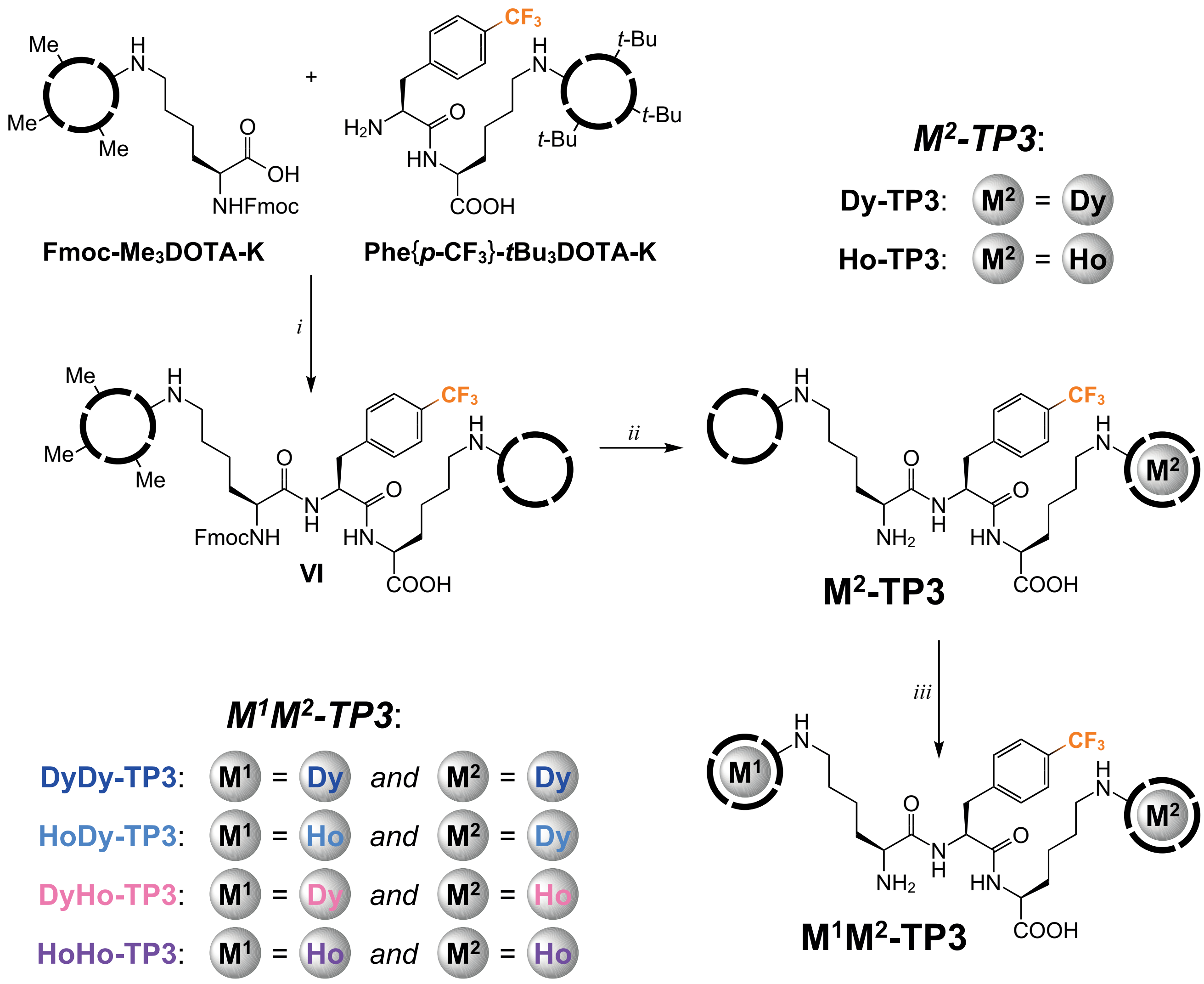

Supplementary Fig. 109. Synthesis of M^1M^2-TP3 tripeptides with Dy^{3+} and Ho^{3+} cations. ***Conditions:*** (*i*) PyAOP, DIPEA, DMSO *followed by* TFA; (*ii*) M^2Cl_3, aq. MOPS/NaOH (pH 7.0) *followed by* LiOH, MeOH, H_2O; (*iii*) M^1Cl_3, aq. MOPS/NaOH (pH 7.0).

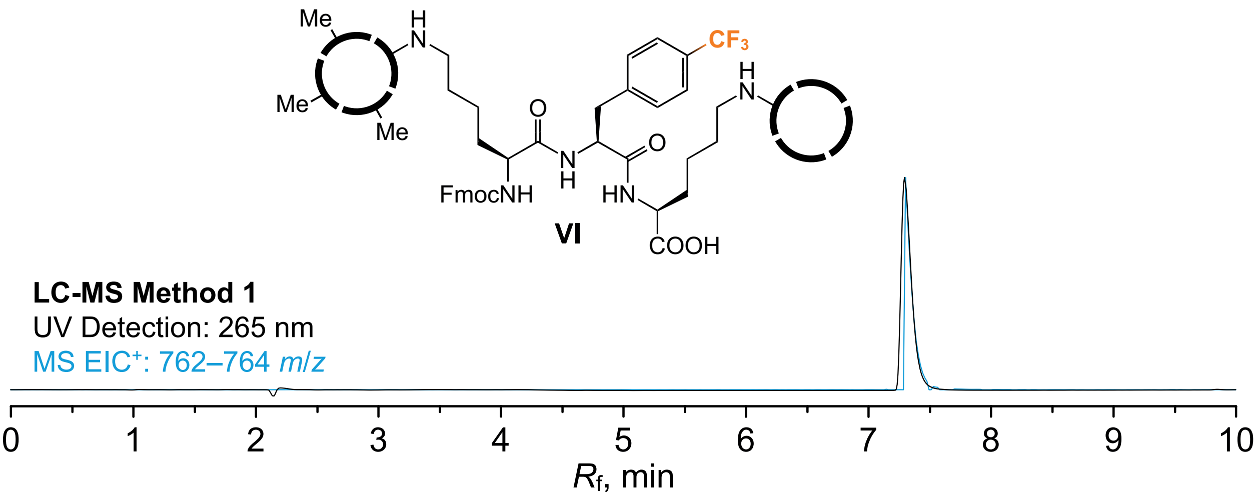

Supplementary Fig. 110. Synthesis and LC-MS chromatogram of intermediate VI. ***Synthesis:*** In a glass vial (4 mL), **Fmoc-Me$_3$DOTA-K**·1.8TFA·1.3H_2O (10 mg, 9.8 µmol, 1.3 equiv.) was dissolved in dry DMSO (650 µL) followed by adition of PyAOP (100 mM in dry DMSO, 95 µL, 9.5 µmol, 1.3 equiv.) and DIPEA (17 µL, 97 µmol, 13 equiv.). After 1 min, **Phe{*p*-CF$_3$}-*t*Bu$_3$DOTA-K** (9.1 mg, 7.6 µmol assuming **Phe{*p*-CF$_3$}-*t*Bu$_3$DOTA-K**·2.5TFA, 1.0 equiv.) was added and the mixture was stirred at RT for 5 mins. Mixture was then purified by preparative HPLC (C18, H_2O/MeCN gradient with 0.1% TFA additive). Fractions with *tert*-butyl protected product were joined and lyophilized. The resulting white solid was dissolved in TFA (3 mL). The resulting solution was stirred at RT for 24 h. Mixture was evaporated to dryness and the residue was purified by preparative HPLC (C18, H_2O/ MeCN gradient with 0.1% TFA additive). Fractions with product were joined and lyophilized to give product as white solid. **Yield:** 6.6 mg (44%; 2 steps; based on **Phe{*p*-CF$_3$}-*t*Bu$_3$DOTA-K**·2.5TFA assuming **VI**·4.0TFA, M_R = 1983). **ESI-HRMS:** 763.8729 $[M+2H]^{2+}$ (theor. $[C_{72}H_{104}O_{20}N_{13}F_3]^{2+}$ = 763.8731).

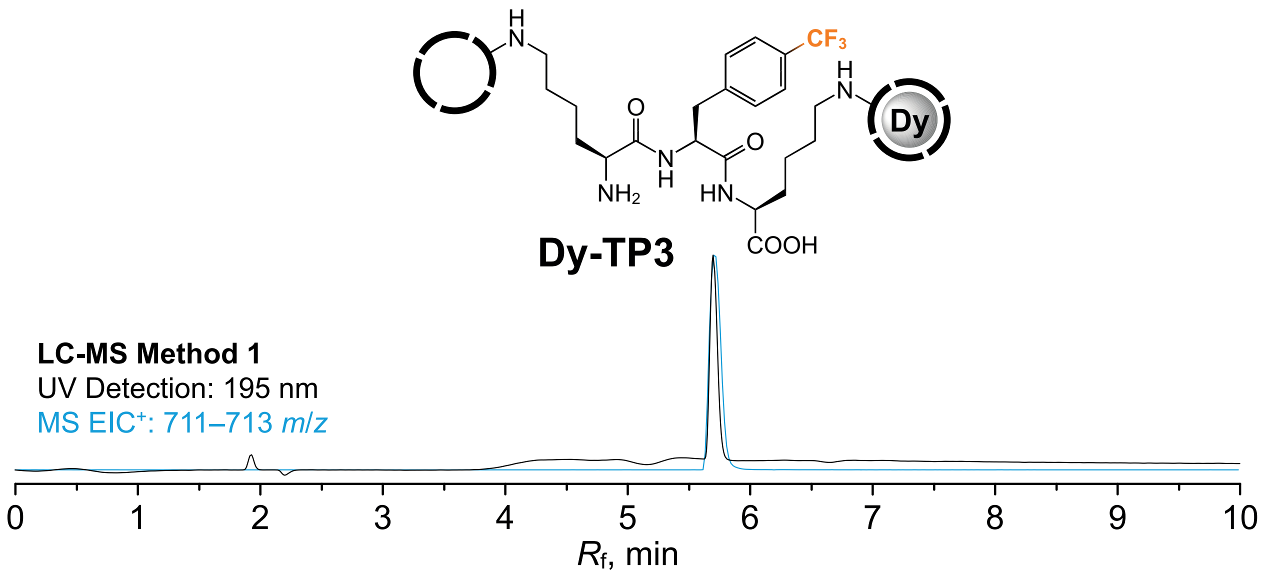

Supplementary Fig. 111. Synthesis and LC-MS chromatogram of intermediate Dy-TP3. ***Synthesis:*** In a glass vial (4 mL), **VI** (3.3 mg, 1.7 µmol assuming **VI**·4.0TFA) was dissolved in aq. MOPS/NaOH buffer (500 mM, pH 7.0, 500 µL, 250 µmol, 150 equiv.) followed by addition of aq. $DyCl_3$ (100 mM, 19 µL, 1.9 µmol, 1.1 equiv.) and the resulting solution was stirred at RT for 15 mins. The mixture was then purified by preparative HPLC (C18, H_2O/MeCN gradient with 0.1% TFA additive). Fractions with Fmoc/methyl ester protected product were joined and lyophilized. The resulting white solid was dissolved in MeOH (460 µL) and H_2O (40 µL) followed by addition of aq. LiOH (1.0 M, 42 µL, 42 µmol, 25 equiv.) and the mixture was stirred at RT for 24 h. Reaction was then quenched by FA (16 µL, 42 µmol, 25 equiv.) and the mixture was evaporated to dryness. The residue was purified by preparative HPLC (C18, H_2O/MeCN gradient with 0.1% FA additive). Fractions with product were joined and lyophilized to give product as white solid. **Yield:** 2.0 mg (78%; 2 steps; based on **VI**·4.0TFA assuming **Dy-TP3**·2.5FA, M_R = 1537). **ESI-HRMS:** 712.2681 $[M+2H]^{2+}$ (theor. $[C_{54}H_{85}O_{18}N_{13}F_3Dy_1]^{2+}$ = 712.2684).

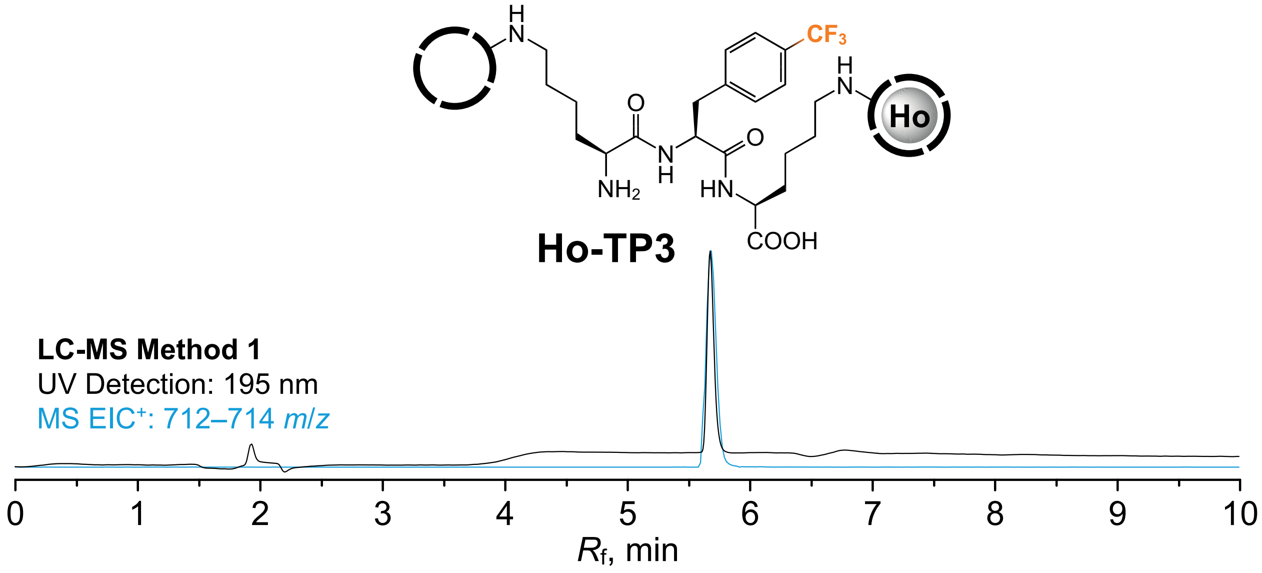

Supplementary Fig. 112. Synthesis and LC-MS chromatogram of intermediate Ho-TP3. ***Synthesis:*** In a glass vial (4 mL), **VI** (3.3 mg, 1.7 µmol assuming **VI**·4.0TFA) was dissolved in aq. MOPS/NaOH buffer (500 mM, pH 7.0, 500 µL, 250 µmol, 150 equiv.) followed by addition of aq. $HoCl_3$ (100 mM, 19 µL, 1.9 µmol, 1.1 equiv.) and the resulting solution was stirred at RT for 15 mins. The mixture was then purified by preparative HPLC (C18, H_2O/MeCN gradient with 0.1% TFA additive). Fractions with Fmoc/methyl ester protected product were joined and lyophilized. The resulting white solid was dissolved in MeOH (460 µL) and H_2O (40 µL) followed by addition of aq. LiOH (1.0 M, 42 µL, 42 µmol, 25 equiv.) and the mixture was stirred at RT for 24 h. Reaction was then quenched by FA (16 µL, 42 µmol, 25 equiv.) and the mixture was evaporated to dryness. The residue was purified by preparative HPLC (C18, H_2O/MeCN gradient with 0.1% FA additive). Fractions with product were joined and lyophilized to give product as pinkish solid. **Yield:** 2.0 mg (78%; 2 steps; based on **VI**·4.0TFA assuming **Ho-TP3**·2.5FA, M_R = 1539). **ESI-HRMS:** 712.7693 $[M+2H]^{2+}$ (theor. $[C_{54}H_{85}O_{18}N_{13}F_3Ho_1]^{2+}$ = 712.7690).

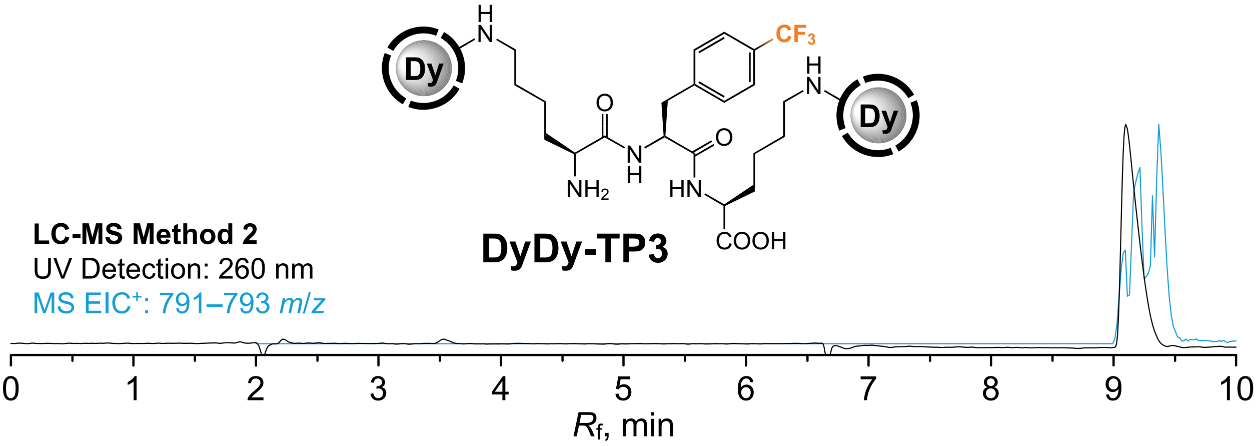

Supplementary Fig. 113. Synthesis and LC-MS chromatogram of DyDy-TP3. ***Synthesis:*** In a glass vial (4 mL), **Dy-TP3** (1.0 mg, 0.6 µmol assuming **Dy-TP3**·2.5FA, 1.0 equiv.) was dissolved in aq. MOPS/NaOH buffer (500 mM, pH 7.0, 500 µL, 250 µmol, 420 equiv.) followed by addition of aq. $DyCl_3$ (100 mM, 7 µL, 0.7 µmol, 1.1 equiv.) and the resulting solution was stirred at RT for 16 h. The mixture was then purified by preparative HPLC (C18, H_2O/MeCN gradient with 0.1% FA additive). Fractions with product were joined and lyophilized to give product as white solid. **Yield:** 0.7 mg (66%; 1 step; based on **Dy-TP3**·2.5FA assuming **DyDy-TP3**·1.0FA, M_R = 1627). **NMR (aq. MOPS pH = 7.0, external D_2O): ^{19}F** (470.4 MHz, T = 298.2 K) δ_F –62.71 (CF_3, m). **ESI-HRMS:** 792.7215 $[M+2H]^{2+}$ (theor. $[C_{54}H_{82}O_{18}N_{13}F_3Dy_2]^{2+}$ = 792.7213).

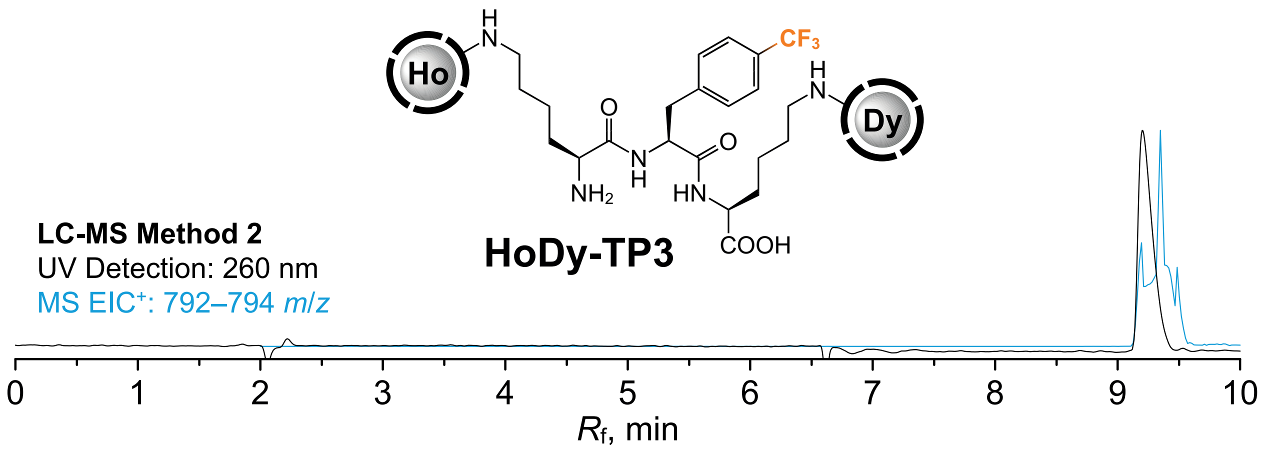

Supplementary Fig. 114. Synthesis and LC-MS chromatogram of HoDy-TP3. ***Synthesis:*** In a glass vial (4 mL), **Dy-TP3** (1.0 mg, 0.6 µmol assuming **Dy-TP3**·2.5FA, 1.0 equiv.) was dissolved in aq. MOPS/NaOH buffer (500 mM, pH 7.0, 500 µL, 250 µmol, 420 equiv.) followed by addition of aq. $HoCl_3$ (100 mM, 7 µL, 0.7 µmol, 1.1 equiv.) and the resulting solution was stirred at RT for 16 h. The mixture was then purified by preparative HPLC (C18, H_2O/MeCN gradient with 0.1% FA additive). Fractions with product were joined and lyophilized to give product as pinkish solid. **Yield:** 1.0 mg (94%; 1 step; based on **Dy-TP3**·2.5FA assuming **HoDy-TP3**·1.0FA, M_R = 1630). **NMR (aq. MOPS pH = 7.0, external D_2O):** ^{19}**F** (470.4 MHz, T = 298.2 K) δ_F –62.79 (CF_3, m). **ESI-HRMS:** 793.2217 $[M+2H]^{2+}$ (theor. $[C_{54}H_{82}O_{18}N_{13}F_3Ho_1Dy_1]^{2+}$ = 793.2218).

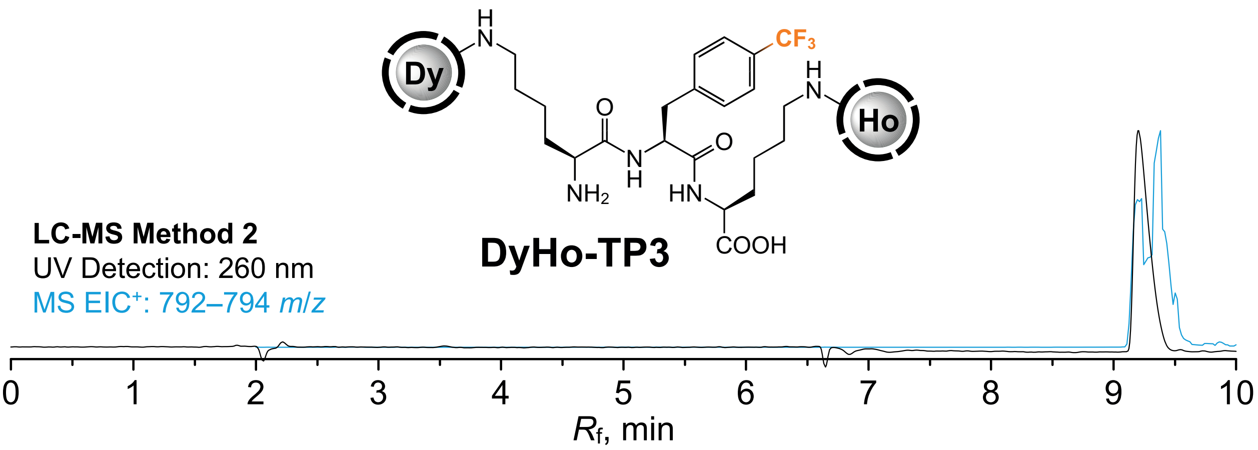

Supplementary Fig. 115. Synthesis and LC-MS chromatogram of DyHo-TP3. ***Synthesis:*** In a glass vial (4 mL), **Ho-TP3** (1.0 mg, 0.6 µmol assuming **Ho-TP3**·2.5FA, 1.0 equiv.) was dissolved in aq. MOPS/NaOH buffer (500 mM, pH 7.0, 500 µL, 250 µmol, 420 equiv.) followed by addition of aq. $DyCl_3$ (100 mM, 7 µL, 0.7 µmol, 1.1 equiv.) and the resulting solution was stirred at RT for 16 h. The mixture was then purified by preparative HPLC (C18, H_2O/MeCN gradient with 0.1% FA additive). Fractions with product were joined and lyophilized to give product as pinkish solid. **Yield:** 0.8 mg (76%; 1 step; based on **Ho-TP3**·2.5FA assuming **DyHo-TP3**·1.0FA, M_R = 1630). **NMR (aq. MOPS pH = 7.0, external D_2O): ^{19}F** (470.4 MHz, T = 298.2 K) δ_F –62.77 (CF_3, m). **ESI-HRMS:** 793.2222 $[M+2H]^{2+}$ (theor. $[C_{54}H_{82}O_{18}N_{13}F_3Dy_1Ho_1]^{2+}$ = 793.2218).

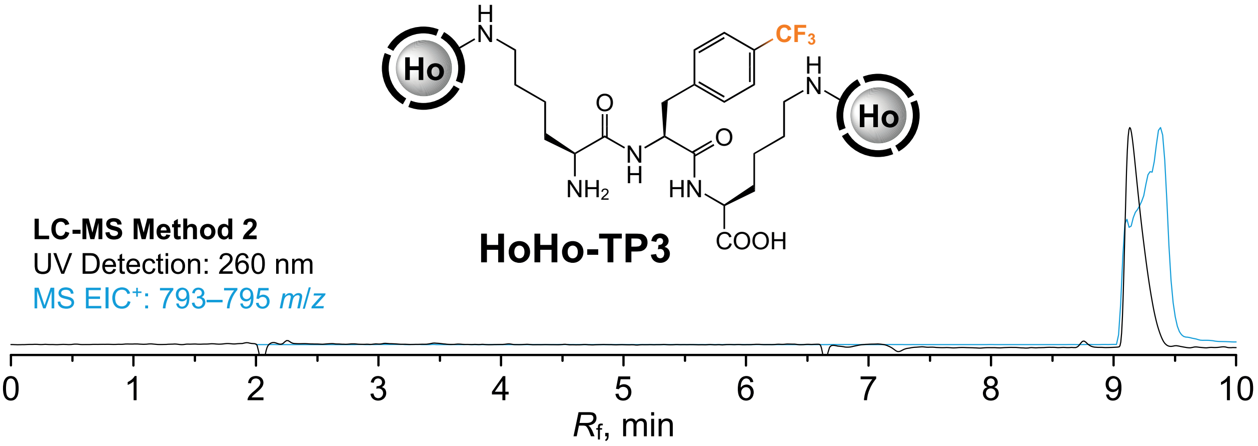

Supplementary Fig. 116. Synthesis and LC-MS chromatogram of HoHo-TP3. ***Synthesis:*** In a glass vial (4 mL), **Ho-TP3** (1.0 mg, 0.6 µmol assuming **Ho-TP3**·2.5FA, 1.0 equiv.) was dissolved in aq. MOPS/NaOH buffer (500 mM, pH 7.0, 500 µL, 250 µmol, 420 equiv.) followed by addition of aq. $HoCl_3$ (100 mM, 7 µL, 0.7 µmol, 1.1 equiv.) and the resulting solution was stirred at RT for 16 h. The mixture was then purified by preparative HPLC (C18, H_2O/MeCN gradient with 0.1% FA additive). Fractions with product were joined and lyophilized to give product as pinkish solid. **Yield:** 1.0 mg (94%; 1 step; based on **Ho-TP3**·2.5FA assuming **HoHo-TP3**·1.0FA, M_R = 1632). **NMR (aq. MOPS pH = 7.0, external D_2O):** ^{19}**F** (470.4 MHz, T = 298.2 K) δ_F –62.72 (CF_3, m). **ESI-HRMS:** 793.7226 $[M+2H]^{2+}$ (theor. $[C_{54}H_{82}O_{18}N_{13}F_3Ho_2]^{2+}$ = 793.7224).

Supplementary References

[1] SHELXT: Sheldrick, G.M. (2015). *Acta Cryst.* A71, 3–8.

[2] SHELXL: Sheldrick, G.M. (2015). *Acta Cryst.* C71, 3–8.

[3] Parsons, S., Flack, H.D. and Wagner, T. (2013). *Acta Cryst.* B69, 249–259.

[4] Bleaney, B. et al., Origin of lanthanide nuclear magnetic resonance shifts and their uses. *J. Chem. Soc. Chem. Commun.* 791–793 (1972).
