# Supplementary material for: Paramagnetic encoding of molecules
Source: Nat Commun. 2022 Jun 8;13:3179. doi: 10.1038/s41467-022-30811-9 (PMC9177614; doi:10.1038/s41467-022-30811-9)
Supplement: Supplementary file 3 — Description of Additional Supplementary Files [file 41467_2022_30811_MOESM3_ESM.pdf]

Description of Additional Supplementary Files

Supplementary Movie 1: This video file shows a fly-through view of the CSI MRI data from Fig. 9.
